# New peptide architectures through C–H activation stapling between tryptophan–phenylalanine/tyrosine residues

**DOI:** 10.1038/ncomms8160

**Published:** 2015-05-21

**Authors:** Lorena Mendive-Tapia, Sara Preciado, Jesús García, Rosario Ramón, Nicola Kielland, Fernando Albericio, Rodolfo Lavilla

**Affiliations:** 1 Institute for Research in Biomedicine, Barcelona Science Park, Baldiri Reixac 10-12, 08028 Barcelona, Spain; 2 Department of Organic Chemistry, University of Barcelona, Martí i Franqués 1-11, 08028 Barcelona, Spain; 3 CIBER-BBN, Networking Centre on Bioengineering, Biomaterials and Nanomedicine; 4 Barcelona Science Park, Baldiri Reixac 10-12, 08028 Barcelona, Spain; 5 School of Chemistry, Yachay Tech, Yachay City of Knowledge, 100119 Urcuqui, Ecuador; 6 Laboratory of Organic Chemistry, Faculty of Pharmacy, University of Barcelona, Avda. Joan XXII s.n., 08028 Barcelona, Spain

## Abstract

Natural peptides show high degrees of specificity in their biological action. However, their therapeutical profile is severely limited by their conformational freedom and metabolic instability. Stapled peptides constitute a solution to these problems and access to these structures lies on a limited number of reactions involving the use of non-natural amino acids. Here, we describe a synthetic strategy for the preparation of unique constrained peptides featuring a covalent bond between tryptophan and phenylalanine or tyrosine residues. The preparation of such peptides is achieved in solution and on solid phase directly from the corresponding sequences having an iodo-aryl amino acid through an intramolecular palladium-catalysed C–H activation process. Moreover, complex topologies arise from the internal stapling of cyclopeptides and double intramolecular arylations within a linear peptide. Finally, as a proof of principle, we report the application to this new stapling method to relevant biologically active compounds.

## Abstract (web summary)

Macrocyclic, constrained peptides show promise in therapeutic applications due to the stable and defined conformations that can be produced. Here, the authors report a method to form macrocyclic peptides through C–H activation on tryptophan and coupling with iodo-substituted aryl amino acids

## Graphical abstract

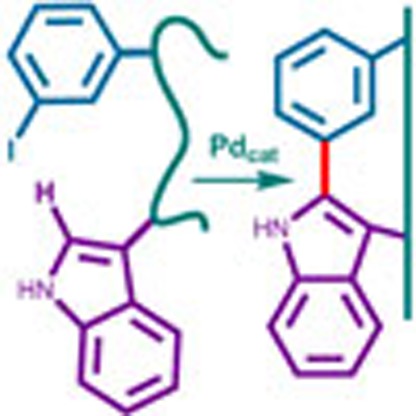

Peptides are attracting great attention as therapeutics since they combine the high selectivity, potency and low toxicity of biologics with advantages such as the conformational restrictions and the reduced costs characteristic of small molecular entities^1^,^2^. In addition to the large number of commercialized peptides, many others are also found in clinical phases, thereby demonstrating their validity and application as active pharmaceutical ingredients^3^. However, the general use of peptides as drugs is severely hampered by their poor pharmacokinetic features. In this regard, it is widely believed that the characterization of protein–protein interactions, a fundamental issue in deciphering biological pathways, is better tackled through structurally defined small peptides with specific sequences. Therefore, there is a need for peptides with new topological architectures; however, these are difficult to obtain even using modern synthetic procedures^4^,^5^. To address these problems, general strategies for peptide macrocyclization have been developed to improve the properties (cell penetration, stability, selectivity and so on) and enhance the potential of peptides as therapeutics and bioprobes, with a special focus on poorly tractable targets^6^,^7^,^8^. In this context, peptides constrained via a non-amide sidechain-to-sidechain linkage (stapled peptides) provide a new structural paradigm because the conformational stabilized species display remarkably stronger biological activity. In this regard, the pioneering work of Verdine *et al*.^9^,^10^ on the basis of all-hydrocarbon staples through olefin metathesis represents a breakthrough in the field.

So far, stapled peptides are generated through a variety of strategies^11^, the main being the use of cysteine side chains to form disulfide bridges^12^ and thioether formation (crosslinking with α,α'-dibromo-*m*-xylene^13^ or aromatic nucleophilic substitutions with perfluoroaromatic reactants^14^); or with functionalized non-natural amino acids by means of biaryl linkages involving the borylated phenylalanine derivatives^15^,^16^,^17^,^18^, ring-closing metathesis^19^ and azide–alkyne cycloadditions (click chemistry)^20^, and so on, usually in expensive and long stepwise syntheses. A comparative table summarizes the major strengths and weaknesses of the main conventional stapling strategies (Fig. 1a). Owing to the increasing interest in stapled peptides and other conformationally restricted structures, many efforts have been made to develop new practical and general preparative methods for their generation.

There is a considerable concern regarding the efficiency of synthetic aspects with respect to the preparation of complex bioactive compounds. In this context, the function-oriented synthesis approach to target simplified, although biologically meaningful, fragments of complex chemical entities is relevant, as it leads to practical syntheses of new specific drugs and probes, also in the field of peptides^21^.

Tryptophan (Trp) has a low relative abundance in peptide and protein sequences (≈1% of the amino acids); however, its presence is critical for the activity of these biomolecules. Therefore, the development of new synthetic methods for the selective and straightforward chemical modification of Trp is highly significant^22^.

Recently, metal-catalysed C–C coupling through direct C–H activation^23^,^24^,^25^,^26^,^27^,^28^,^29^ has become a fundamental process in modern organic syntheses, allowing the straightforward preparation of a plethora of new structural types. In this respect, the functionalization of indoles using this approach has been extensively examined^30^,^31^, including studies described in (refs 28, 32, 33, 34, 35) among others. Particularly relevant to our research was the methodology of Larrosa for indole arylation using Pd(OAc)_2_ in acidic media^36^. In particular, although the Pd-mediated C-2 arylation of Trp has been reported^37^,^38^,^39^, it has not yet been applied to staple true peptides. We recently disclosed the direct C-2 arylation of indoles in Trp-containing peptidic sequences in an intermolecular manner, without additional requierements^37^. Although the alternative *N*-arylation processes are conceivable, such Ullmann-type reactions take place normally with Cu and Pd catalysts, in the latter case usually requiring strong bases^40^. No experimental evidence of these transformations have been recorded in our systems. The main restriction is that lower conversions are obtained for sequences that comprise methionine, cysteine or histidine residues, presumably because of selective hydrolysis of the peptide bond catalysed by bidentate palladium coordination; on-going experiments along this line show that working in nonaqueous solvents allows reasonable arylations (unpublished results). Later we reported on the arylation of Trp-diketopiperazines^41^ and related transformations of Trp derivatives, leading to the generation of Fmoc-protected arylated Trps that are amenable to direct incorporation in solid-phase peptide synthesis (SPPS)^42^. These processes work well with *N*-protected peptides in dimethylformamide (DMF) or in aqueous environments.

In this work, we present a new stapling methodology involving Trp and Phe(Tyr) through a Pd-catalysed C–H activation process. The method is versatile, allowing the formation of constrained peptides of different ring sizes, amenable to solution- and solid-phase synthesis and can lead to the direct preparation of biologically meaningful peptide derivatives.

## Results

### Preliminary studies

**Figure 1:**
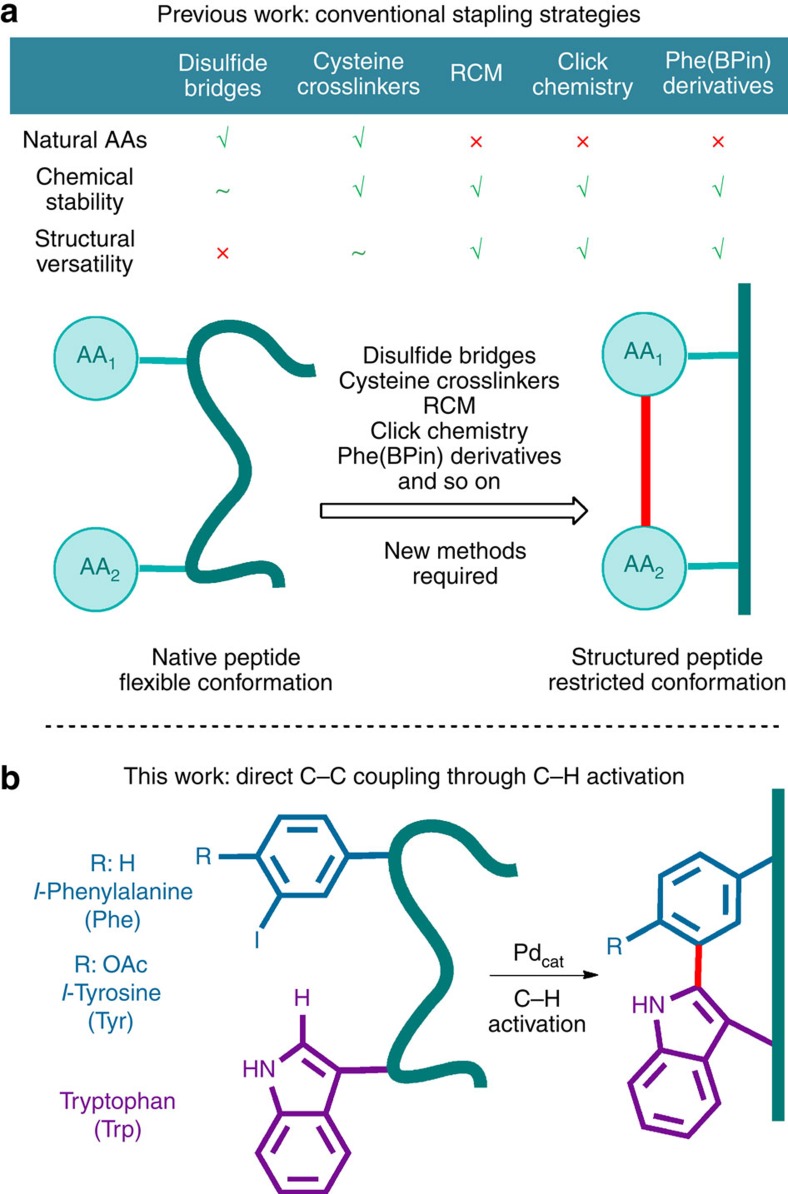
Formation of stapled peptides. (**a**) Conventional stapling methods. (**b**) Phe/Tyr–Trp stapling via a selective Pd-catalysed C–H arylation process.

Here we present a one-step process for the synthesis of Trp–Phe(Tyr)-stapled peptides directly from commercially available precursors. The method is based on an intramolecular Pd-catalysed C–H activation reaction between a Trp residue and an iodo-phenylalanine (or tyrosine) unit (Fig. 1b).

After a preliminary modelling study, we established the structural features for the intramolecular C–H arylation, placing the iodo-substituent for Phe or Tyr amino acids in the *meta* position. The preferential distances between these latter residues and Trp ranged from one to three amino acids in a series of linear *N*-terminal-acetylated sequences.

**Figure 2:**
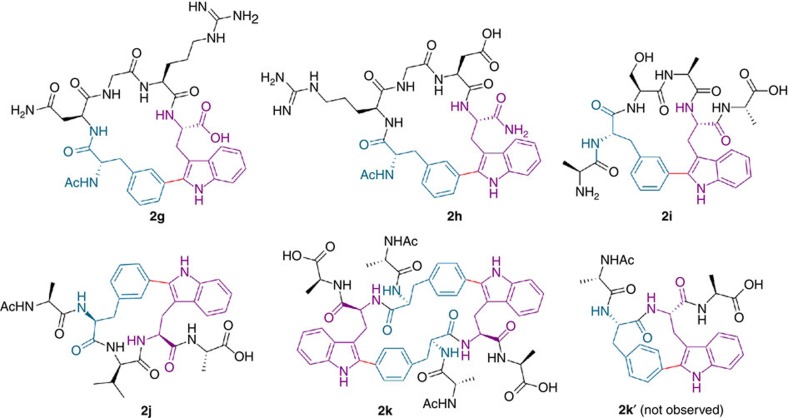
Structure of isolated locked peptides **2g–2k**. Schematic representation: Phe (blue), Trp (purple) and staple bond (red).

The initial experiments were carried out using the conditions previously applied in the arylation of the Trp diketopiperazine:^41^ Overall, 5 mol% Pd(OAc)_2_, AgBF_4_ (1.0 eq.) and *o*-nitrobenzoic acid (2-NO_2_BzOH) (1.5 eq.) in PBS:DMF (1:1) under microwave (MW) irradiation at 80 °C for 15 min (Table 1, entries 1–6). These preliminary observations suggested that these arrangements are suitable for stapled peptide formation, affording good to excellent conversions for Trp–Phe(Tyr) staples located at i−i+2, i−i+3 and i−i+4 positions (Table 1, entries 1–6). Furthermore, a series of representative sequences displaying from one to three amino acids between Trp and *m*-iodinated Phe were constrained in useful yields (Table 1, entries 7–10; Fig. 2; see Supplementary Fig. 1), in an anhydrous medium using trifluoroacetic acid (TFA) as the acid, applying the optimized conditions previously reported for the preparation of Fmoc-protected arylated Trps^42^. Although these transformations may lead to atropoisomeric diastereomers, only one stereochemically defined structure was observed in each case. Of note is the successful application of this technique to stapled peptides containing the Asn-Gly-Arg (-NGR-) and Arg-Gly-Asp (-RGD-) tumour-homing signalling sequences (**2g** and **2h**, Table 1, entries 7 and 8, respectively).

Preliminary studies on the capacity of the RGD-containing compound **2h** and its linear precursor **1h** to inhibit cellular adhesion showed that these substances act selectively as antagonists for the ανβ3 in front of ανβ5 integrin receptors; compound **2h** showing a moderate EC_50_ (6 μM) is more active than its linear precursor (**1h**, 26 μM, see Biochemical and Cellular Studies in Supplementary Methods). Compound **2h** is considerably less potent than cilengitide, not surprisingly as this drug has been thoroughly optimized. However, it has to be considered that even linear analogues display a much lower potency (10–10^3^ times, depending on the targeted integrin) than the parent cilengitide^43^,^44^. These preliminary results clearly show that this stapling technique preserves the activity of the natural sequence, while improving its potency, in line with pioneering experiments in macrocyclization techniques for medchem peptide development^45^,^46^.

We next tackled the preparation of the more challenging locked peptide i−i+1. Three sequences with adjacent Trp—(*ortho*-, *meta*- or *para*)-I-Phe were synthesized. A constrained C–C linked structure was obtained for the *meta*-derivative (60%), although it could not be properly characterized. With respect to the *ortho* analogue, only the reduced peptide was detected. Interestingly, the *para*-I-Phe peptide **1k** was successfully reacted under the usual conditions to yield cyclodimer **2k** (60%, Table 1, entry 11; see Supplementary Figs 2 and 3). Presumably, the putative monomeric structure **2k′** would be highly strained (molecular models display a nonplanar phenyl ring, see Supplementary Fig. 4) and the process evolves through a ditopic pathway, in a remarkable demonstration of the process versatility.

A detailed nuclear magnetic resonance (NMR) study was performed on dimethylsulphoxide to analyse the conformational behaviour of the peptides synthesized. The NMR spectra of linear sequences **1g–1j** were indicative of flexible, unstructured peptides. H_α_ and ^13^C_α_ chemical shifts displayed values typical for random coil, and the NH temperature coefficients and ^3^*J*(H_α_NH) couplings were within the range expected for unstructured peptides^47^.

**Figure 3:**
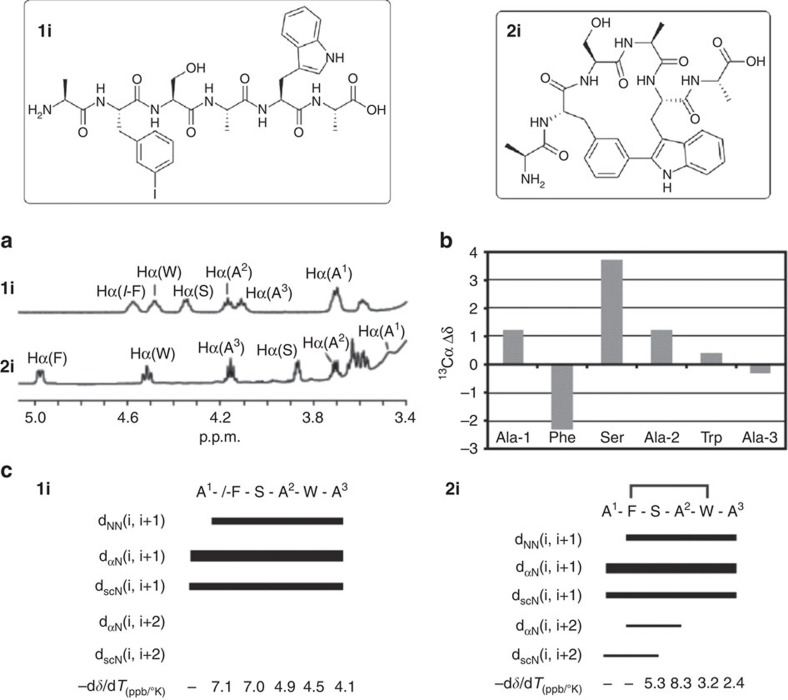
Peptide NMR spectra comparison between stapled peptide **2i** and its linear counterpart **1i**. (**a**) NMR H_α_ region of peptide **2i** and its linear precursor **1i**. (**b**) Plot of the ^13^C_α_ chemical shift differences (^13^C_α_ Δ*δ*_cyclic–linear_) between stapled peptide **2i** and its linear counterpart **1i**. (**c**) Summary of NOE connectivity and temperature coefficients of the NH amide protons (Δ*δ*/Δ*T*) of peptide **1i** (bottom left) and **2i** (bottom right). The thickness of the bars reflects the intensity of the NOEs, that is, weak (
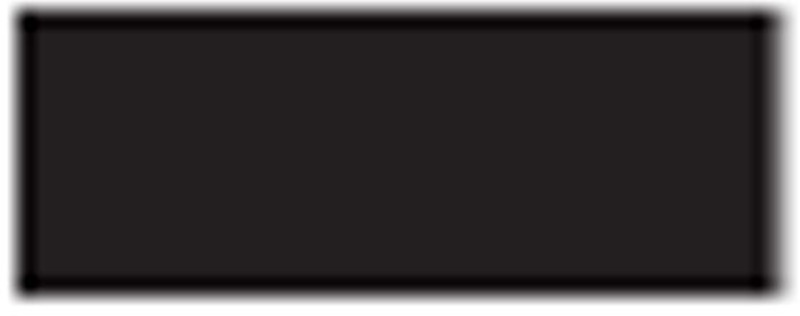
), medium (
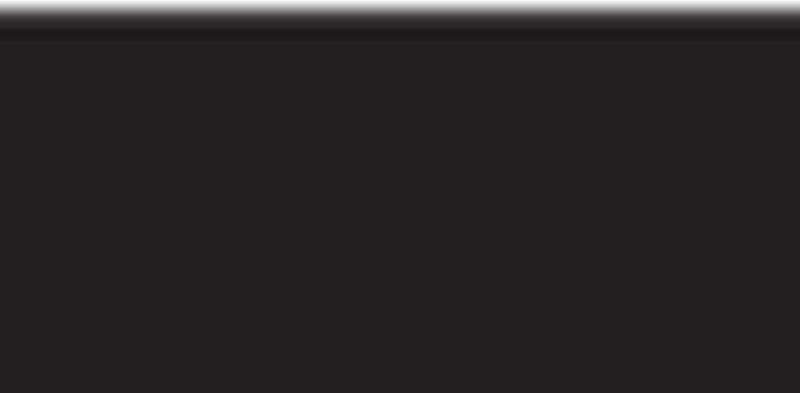
) and strong (▪). *I*-F: *m*-iodophenylalanine.

Staple bond formation caused a substantial modification of the peptide NMR spectra. Compared with their linear precursors, peptides **2g–2j** showed larger H_α_ chemical shift dispersion (Fig. 3a and Supplementary Figs 5–7), indicating less conformational flexibility. However, broad resonances were observed at 25 °C for some backbone NH protons, suggesting that peptides **2g–2j** show some flexibility. Significant differences in ^13^C_α_ chemical shifts were observed between stapled peptides **2g–2j** and their linear counterparts **1g–1j** (Fig. 3b and Supplementary Figs 5–7). The temperature dependence of the amide NH groups was also significantly affected by staple formation (Fig. 3c and Supplementary Figs 5–7). Stapled peptides showed a wider distribution of Δ*δ*/Δ*T* values within the amino-acid sequence and also featured values characteristic of solvent-shielded NH groups (lower than −3 ppb/K). NOE connectivity was also affected by staple formation. In the case of peptides **2h**, **2i** and **2j**, several nonsequential NOEs indicated that the peptides were constrained by intramolecular cyclization (Fig. 3c and Supplementary Figs 6 and 7). Incidentally, we detect all N_indole_–H atoms, and also we find the corresponding indole Cα positions consequently substituted, therefore ruling out any interference by *N*-arylation processes. The aryl–aryl moieties show NOE correlations consistent with a defined single configuration in each case, which matches with the arrangement predicted from molecular modelling.

Moreover, circular dichroism measurements of stapled peptides **2g** and **2h** were performed in order to detect evidence of secondary structure and were then compared with their linear precursors **1g** and **1h**, respectively (See Supplementary Figs 8–11). Stapled peptides **2** show a positive maximum at ∼190 nm and minimum peaks at 206 nm, which indicate some levels of structuring. In contrast, linear peptides exhibit a more flattened profile typical of a flexible unfolded structure.

### Extension to biologically relevant peptides

**Figure 4:**
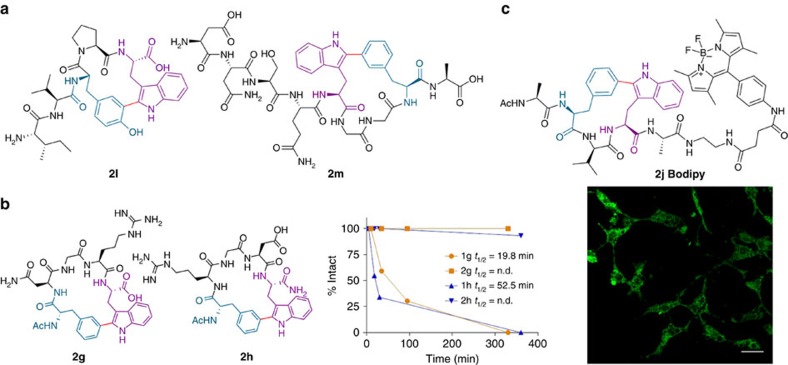
Bioactive stapled peptides, biochemical and cellular studies. (**a**) Structure of stapled peptides **2l** (valorphin analogue) and **2m** (baratin analogue). (**b**) Proteolytic degradation assay of stapled peptides **2g** and **2h** and their linear precursors **1g** and **1h**. (**c**) Structure of labelled-stapled peptide **2j Bodipy** (left) and the corresponding confocal microscopy image of SH-SY5Y cells treated with compound **2j Bodipy** (750 nM). Scale bar, 25 μm (right).

To further explore the potential of the methodology, we planned to make the stapled analogues of known linear bioactive peptides, and at the same time test the influence of new features, such as the presence of proline (Pro) in the sequence, the overall length of the chain (up to nine amino acids) and the possibility of performing the C–H arylation on the solid phase, which enables the transformation of unprotected *N*-terminal peptides. In this way, we prepared the stapled version of an active valorphin analogue^48^, which is a potent dipeptidyl peptidase III inhibitor, displaying Pro in a five amino-acid sequence containing also Trp and Tyr (see Supplementary Fig. 12). Interestingly, this peptide is closely related to spinorphin, an endogenous antinociceptive peptide, a potent and noncompetitive antagonist at the ATP-activated human P2X3 receptor^49^. The SPPS method was used to synthesize the Fmoc-protected sequence, which was intramolecularly arylated on resin. Subsequent *N*-terminal deprotection and cleavage, successfully afforded the stapled peptide **2l** (Fig. 4a). This solid-phase protocol is fully compatible with the Pd chemistry involved and, interestingly, allows the preparation of arylated sequences having unprotected terminal amino groups. Futhermore, baratin^50^, a neurostimulating peptide, was stapled following a related procedure. In this way, the standard SPPS protocol was interrupted to perform the on-resin C–H activation step, to be continued with the incorporation of the final four amino acids. Afterwards, deprotection and cleavage afforded the desired derivative **2m** (Fig. 4a, see Supplementary Methods and Supplementary Fig. 12).

Next, we determined the proteolytic stability of a couple of meaningful stapled peptides, in comparison with their respective linear counterparts. We followed a chymotrypsin-based protocol, previously used to evaluate stapled peptides^51^. Plotting the HPLC-MS profiles of the couples **2g**/**1g** and **2h**/**1h** (Fig. 4b, also Supplementary Figs 13–15) clearly shows an almost total protection of the stapled peptides towards enzymatic degradation, after 5–6 h, whereas the linear precursors suffered a rapid hydrolytic cleavage to remove the *N*-terminal amino acid, and completely disappeared after this time lapse.

Finally, we studied the controlled labelling of the stapled peptides. The goal was to selectively attach a fluorophore to the *C*-terminal amino acid to trace cellular permeabilization and localization. Thus, we linked a Bodipy residue to cyclopeptide **2j** through an amino spacer (the novel Bodipy construct was designed and prepared for this purpose, see Supplementary Fig. 16 and Supplementary Methods), to get the desired labelled-stapled peptide **2j Bodipy** (Fig. 4c) in a convenient manner. This compound exhibits low cytotoxicity at 750 nM after 24 h of incubation in SH-SY5Y cells (MTT assay; see Supplementary Fig. 17), and this concentration was used for the cell penetration studies. In a preliminary flow cytometry experiment (fluorescence-activated cell sorting assay), SH-SY5Y cells resulted in brightly stained sections on incubation for 30 min (see Supplementary Fig. 18). The cells treated in this manner were analysed using confocal microscopy. The stapled peptide **2j Bodipy** was localized both in the membrane and in the cytoplasmatic region (Fig. 4c). Incidentally, the Bodipy-labelled linear precursor **1j Bodipy** also penetrates into cells in a comparable extent; however, there are appreciable differences in toxicity and cell permeability with respect to the stapled analogue (see Supplementary Figs 19 and 20). Overall, these results enable the performance of systematic bioimaging studies on these compounds.

### Intermolecular peptide conjugation through C–H activation

Chemical conjugation can effectively link drugs to carriers, and this technique is routinely used to modify biologics, especially antibodies with therapeutic indications^52^. We next explored peptide–peptide conjugation via C–H activation to achieve bismacrocyclic peptide constructs linked through a nonhydrolysable bond.

**Figure 5:**
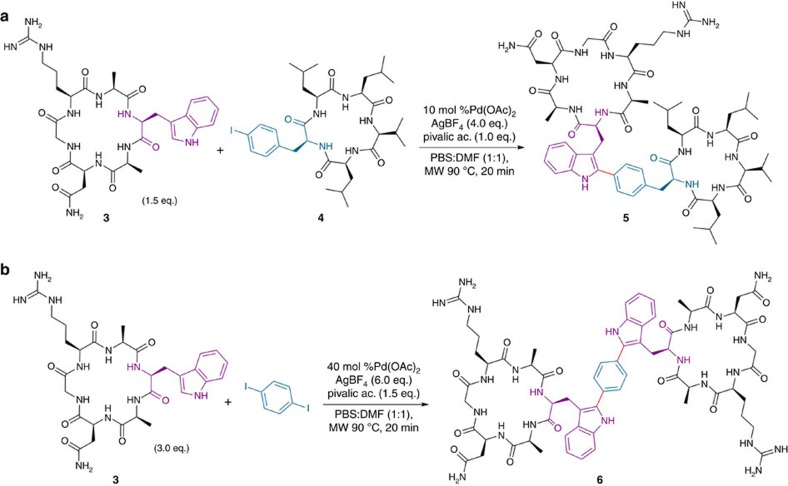
Macrocyclic conjugation via C–H activation. (**a**) Intermolecular conjugation of NGR cyclopeptide **3** with the sansalvamide derivative **4** via C–H activation. (**b**) Double conjugation of the NGR cyclopeptide **3** with 1,4-diodobenzene.

Peptides that inhibit the new blood vessel growth (angiogenesis) have become a promising tool for treating cancer. In this context, it has been reported that cyclic forms of NGR-containing sequences (tumour-homing peptides) lead to an improvement of the anticancer activity of an associated drug^53^. We selected the previously studied Asn-Gly-Arg (NGR) array and synthesized the corresponding Trp-containing macrocycle **3** to be conjugated to a synthetic *p*-I-Phe-cyclopeptide derivative of the cyclic depsipeptide sansalvamide A (**4,** Fig. 5a), a natural product whose synthetic analogues have demonstrated significant anticancer activity^54^. Using a standard Pd-catalysed reaction in an aqueous medium of PBS–DMF, the two partners were successfully coupled to yield the C–C conjugate **5** (Fig. 5a).

In a second example, the conjugation of two units of the NGR Trp-containing cyclopeptide **3** with a 1,4-diiodobenzene connector via a double C–H arylation was achieved. Again, the process yielded the expected bis-NGR-adduct **6** in a single step (Fig. 5b).

To evaluate the effects of macrocyclic conjugation, the NMR spectra of conjugated peptide **5** and its cyclic precursors **3** and **4** were compared (see Supplementary Fig. 21). After conjugation, small shifts were observed in the H_α_ resonances of the sansalvamide A analogue **4**. In contrast, the H_α_ chemical shifts of the NGR-containing cycle were almost identical in peptides **5** and **3**. Comparison of ^13^C_α_ chemical shifts showed that in both cycles the larger ^13^C_α_ chemical shift change corresponded to the residue involved in the intermolecular bond formation: Trp (1.2 p.p.m.) in the NGR-containing cycle and Phe (0.6 p.p.m.) in the sansalvamide A derivative. More modest changes were observed in the ^13^C_α_ of the remaining amino acids. The resemblance of the H_α_ and ^13^C_α_ chemical shifts, which are highly sensitive to conformational changes, suggests that conjugation provided a minimal perturbation of the overall structures of peptides **3** and **4**. The NMR analysis was extended to peptide **6** and its precursor **3**. The effects of conjugation in the H_α_ and C_α_ resonances are shown in Supplementary Fig. 22.

Unfortunately, in preliminary biological studies evaluating the conjugated peptide **5** and its macrocycle precursors **3** and **4** against several cancer cell lines, only the cyclopeptide **4** showed activity (IC_50_<10 μM). Nevertheless, the protocol seems general and offers new possibilities for peptide-based conjugation.

### Stapled cyclopeptides

**Figure 6:**
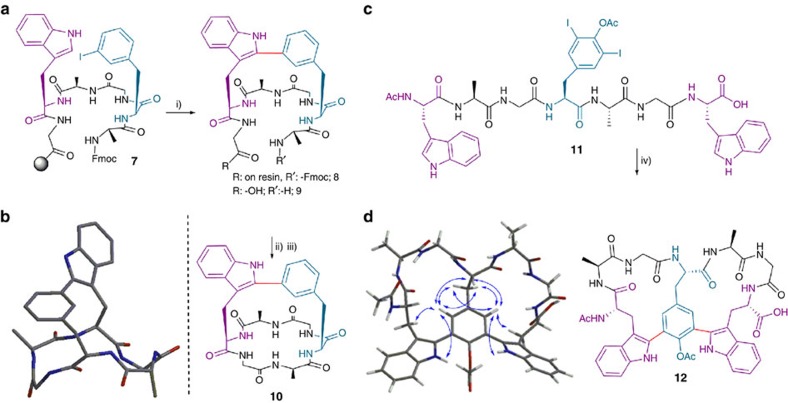
Synthesis of macrobicyclic peptides 10 and 12. (**a**) Synthesis of macrobicyclic peptide **10** through solid-phase stapling. Reaction conditions: (i) Pd(OAc)_2_ (0.05 eq.), AgBF_4_ (1.0 eq.), 2-NO_2_BzOH (1.5 eq.), DMF, MW 90 °C, 20 min; (ii) (1) 1% sodium diethyldithiocarbamate (DDC) in DMF. (2) Piperidine–DMF (1:4; 1 × 1 min, 2 × 5 min). (3) TFA-TIS-H_2_O (95:2.5:2.5), r.t, 1 h; (iii) PyAOP (2.0 eq.), DIEA (6.0 eq.), DMF, r.t, 1.5 h. (**b**) Minimized geometry of compound **10** generated by the Spartan '14 suite. Hydrogens omitted for clarity. (**c**) Double C–H arylation to cyclic biaryl **12** (25% conversion, estimated by HPLC). Reaction conditions: (iv) 40 mol %Pd(OAc)_2_, AgBF_4_ (6.0 eq.), pivalic ac. (1.5 eq.), DMF, MW 90 °C, 20 min. (**d**) Minimized geometry of compound **12** generated by Spartan ‘14 suite showing the diagnostic NOE correlations (blue arrows).

We then explored access to complex topologies, focusing on the synthesis of bicyclic peptide chemotypes. In a first approach, the synthesis of a stapled cyclopeptide was attempted by promoting the usual C–H arylation through an intramolecular Trp-I–Phe interaction. Preliminary studies showed that alanine (Ala) hexapeptides containing a *m*-I-Phe and Trp units, respectively, placed at positions i−i+2 and i−i+3 afforded the corresponding macrocycles after routine amide coupling in quantitative yields; however, the subsequent intramolecular C–H arylations did not take place under the standard conditions, probably because of a highly restricted conformation. Remarkably, when the stapling on the linear sequence anchored to the resin **7** was carried out first on the solid phase, we obtained the corresponding NH-free amino terminal-stapled peptide **9** after cleavage from resin. Finally, we isolated the desired bicyclic compound **10** by amide cyclization (Fig. 6a,b). Furthermore, in this way, we overcame the relative limitation of irreversible protection of the *N*-terminal amino group, as previously reported^37^. Incidentally, preliminary results showed that increasing the Pd(OAc)_2_ amount up to 0.2 eq. gave acceptable intermolecular arylations for NH-free amino terminal sequences (data not shown). The stapled cyclopeptide **10** was prepared in a suitable manner, involving a solid-phase arylation, thus facilitating the purification step, followed by a routine peptide coupling. This protocol may enable the synthesis of further derivatives of this attractive and unexplored structural class.

The ^1^H NMR spectrum of the linear stapled peptide **9** was characterized by a large chemical shift dispersion of the NH and H_α_ resonances. Bicyclopeptide **10** showed a slightly larger chemical shift range for the H_α_ protons, as expected for a more rigid and structured peptide. Backbone cyclization was also reflected in a large splitting of the methylene H_α_ atoms of Gly-1 and H_β_ atoms of Phe. In the case of peptide **9**, the chemical shift difference between the two geminal protons was <0.2 (H_α_, Gly-1) and 0.1 (H_β_, Phe) p.p.m., whereas for peptide **10** this difference increased to >0.9 (H_α_, Gly-1) and >0.7 (H_β_, Phe) p.p.m. Backbone cyclization was further evidenced by significant changes in ^13^C_α_ chemical shifts and in the temperature coefficients of amide NH (Supplementary Fig. 23).

### Double stapling of linear peptides

Biaryl bismacrocyclic peptide-derived natural products such as vancomycin^55^ and complestatin^56^ display very interesting bioactivity profiles and as synthetic targets they are extremely difficult to prepare^57^. Thus, the development of new methodologies to access simplified scaffolds is crucial to enable structural diversification, thereby allowing practical medicinal chemistry and biological studies. Hence, it was envisioned that intramolecular double C–H arylation of a sequence containing a diiodinated Tyr (commercially available) flanked by two Trp units would give raise to bicyclic peptide topologies with adjancent biaryl moieties in a straightforward manner. In order to establish the conditions for this transformation, we performed some preliminary experiments where the intermolecular C–H activation of a Ac-*m,m′*-I,I-Tyr(OAc)-OH unit with 2.0 eq. of Ac-Trp-OH was tested. An increase in the amount of the Pd catalyst (40%) and AgBF_4_ (6.0 eq.)^58^ and use of a mild excess of pivalic acid resulted in a productive reaction (see Supplementary Methods). Next, we designed the linear peptide sequence **11** (Fig. 6c), which was synthesized in a straightforward manner on the solid phase. Using the previous C–H activation conditions for the diodoTyr, we successfully obtained the double stapled peptide **12** directly from the corresponding linear precursor in one step in a 25% HPLC conversion, together with monoarylated cycles and dehalogenated derivatives, which were probably produced in competitive processes (Fig. 6c). This remarkable result is the proof of principle that even these complex peptidic topologies are accessible through the present methodology. The ^1^H NMR spectrum of the double stapled peptide **12** is characterized by a wide chemical shift range for the NH and H_α_ protons (see Supplementary Fig. 134). The observed pattern of NOEs, summarized in Fig. 6d, indicates that each side of the Tyr aromatic ring faces a distinct Trp-Ala-Gly motif.

As an optimization of this methodology, we have developed the exclusive use of standard proteinogenic amino acids (Tyr), which, once coupled, are modified *in situ*, thus simplifying the protocol and rendering it considerably more affordable. In this way, the corresponding linear precursor of peptide **11** can be obtained through routine amide couplings followed by on-resin iodination of the Tyr residue to yield derivative **11** (41% conversion, unoptimized, see Supplementary Fig. 24)^59^. This remarkable result enables the direct synthesis of bicyclopeptide **12** to be performed on solid-phase from commercially available reagents in a single sequence.

## Discussion

Metal-catalysed arylations through C–H activation are suitable processes to gain access to minimalistic staples (two-electron) in relevant Trp-containing peptide sequences directly from Trp and iodo-phenylalanine (-tyrosine) precursors. The validation of the methodology includes the analysis of the scope of the transformation, compatibility with other amino acids and the applicability to SPPS. This approach has also been applied to constrain biologically active signalling sequences. In an intermolecular mode, the process efficiently links two Trp peptides to a benzene connector and is also useful to conjugate peptides through a C–C bond. All compounds showed a structured nature, as revealed by spectroscopic characterization. Finally, we have developed a simple protocol for the straightforward access to novel peptide topologies such as dimeric macrocycles, stapled bicyclopeptides and biaryl–biaryl species (see Supplementary Figs 4 and 25) from the corresponding linear precursors in only one step. These findings open up general access to a variety of novel constrained peptidic chemotypes, and we believe that this breakthrough will make a significant contribution to the development of a broad range of applications for peptides in biological and medicinal chemistry.

## Methods

### General

For abbreviations and detailed experimental procedures see Supplementary Methods. For NMR analysis of the compounds, see Supplementary Figs 26–148 and Supplementary Tables 1–40.

### Peptide synthesis

All peptides were manually synthesized on a 2-Chlorotrityl, H-Rink-Amide Chemmatrix or TentaGel S NH_2_ resin using standard Fmoc chemistry for SPPS.

### Linear peptide cyclization

The free-amine free-acid linear peptide (1.0 eq.) was dissolved in ACN/DMF or DMF (0.001–0.003 M) and N,N-diisopropylethylamine (DIEA) (6.0 eq.) and the corresponding coupling agents (1.5–3.0 eq., benzotriazol-1-yl-oxytripyrrolidinophosphonium hexafluorophosphate (PyBOP) with hydroxybenzotriazole (HOBt) or 1-[Bis(dimethylamino)methylene]-1H-1,2,3-triazolo[4,5-b]pyridinium 3-oxid hexafluorophosphate (HATU) and O-(benzotriazol-1-yl)-N,N,N′,N′-tetramethyluronium tetrafluoroborate (TBTU) or (7-azabenzotriazol-1-yloxy)tripyrrolidinophosphonium hexafluorophosphate (PyAOP)) were added. The solution was stirred at r.t until the cyclization was complete (1–3 h). Workup was performed by extraction with aqueous solutions of NH_4_Cl_sat_ and NaHCO_3sat_. Organic layers were combined, dried over anhydrous sodium sulfate, filtered and concentrated under vacuum. When remaining protecting groups were present, the macrocycle was treated with a 95% TFA, 2.5% triisopropylsilane (TIS) and 2.5% H_2_O cocktail (3 h), washed with Et_2_O, dissolved in ACN:H_2_O and lyophilized to furnish the corresponding deprotected peptide. When necessary, the macrocycle was purified by flash column chromatography on silica gel or semipreparative RP-HPLC.

### General protocol for the stapled bond formation in solution

The linear peptide (50 mg), AgBF_4_ (2.0 eq.), trifluoroacetic acid (1.0 eq.) and Pd(OAc)_2_ (0.05 eq.) were placed in a MW reactor vessel in DMF (1.2 ml). The mixture was heated under MW irradiation (250 W) at 90 °C for 20 min. The residue was filtered and purified using semipreparative RP-HPLC. This process was scaled up to 0.907 mmol of peptide, affording the stapled/locked peptides **2g**–**2k** in isolated yields ranging from 1 to 32%.

### Procedure for the single macrocycle conjugation

Cyclopeptide **4** (40.0 mg, 0.056 mmol), cyclopeptide **3** (55.3 mg, 0.084 mmol, 1.5 eq.), AgBF_4_ (43.8 mg, 0.225 mmol, 4.0 eq.), pivalic acid (5.7 mg, 0.056 mmol, 1.0 eq.) and Pd(OAc)_2_ (1.4 mg, 0.077 mmol, 0.1 eq.) were placed in a MW reactor vessel in 2 ml of PBS:DMF (1:1). The mixture was heated under MW irradiation (250 W) at 90 °C for 20 min. The irradiation cycle was repeated after adding a new portion of Pd(OAc)_2_ and AgBF_4_. The residue was filtered and partially purified in a PoraPak Rxn reverse phase column (1.63 mg, 2% estimated using HPLC-MS). A pure fraction was obtained using analytic RP-HPLC to yield pure conjugate **5**.

### Procedure for the double macrocycle conjugation

1,4-diiodobenzene (35 mg, 0.106 mmol), macrocycle **3** (209 mg, 0.318 mmol, 3.0 eq.), AgBF_4_ (124 mg, 0.637 mmol, 6.0 eq.), pivalic acid (16.3 mg, 0.159 mmol, 1.5 eq.) and Pd(OAc)_2_ (9.5 mg, 0.042 mmol, 0.4 eq.) were placed in a MW reactor vessel in 2 ml of PBS:DMF (1:1). The mixture was heated under MW irradiation (250 W) at 90 °C for 20 min. The crude product was filtered, and the workup was carried out by washing with AcOEt and then precipitating by adding ACN to the aqueous phase. The resulting precipitate was washed with ACN, decanted and dried, obtaining 159 mg of crude product (pale solid, 42% estimated using HPLC-MS). A pure fraction was obtained with semipreparative RP-HPLC to yield pure conjugate **6**.

### Typical procedure for the stapled cyclopeptide formation on the solid phase

Once sequence **7** was synthesized on a TentaGel S NH_2_ resin via an AB linker, the peptide anchored to the resin (139 mg, 0.145 mmol), AgBF_4_ (28 mg, 0.144 mmol, 1.0 eq.), 2-nitrobenzoic acid (36 mg, 0.215 mmol, 1.5 eq.) and Pd(OAc)_2_ (1.6 mg, 7.1 μmol, 0.05 eq.) in DMF (2 ml) was placed in a MW reactor vessel. The mixture was heated under MW irradiation (250 W) at 90 °C for 20 min. Eight more batches were carried out following the same procedure and were then combined. The resin was treated with 1% DDC in DMF and the Fmoc group was removed. The peptide was cleaved from the resin with a 95% TFA, 2.5% TIS and 2.5% H_2_O cocktail (1 h), yielding the stapled sequence **9** (85% purity, estimated using HPLC-MS). Finally, the stapled peptide **9** was cyclized (see above for standard cyclization procedure) and the crude product was purified using semipreparative RP-HPLC to yield bicyclopeptide **10** (pale solid, 18% unoptimized).

### One-step double arylation to biaryl–biaryl stapled peptide

Linear peptide **11** (50 mg, 0.044 mmol), AgBF_4_ (51 mg, 0.262 mmol, 6.0 eq.), pivalic acid (6.7 mg, 0.066 mmol, 1.5 eq.) and Pd(OAc)_2_ (3.9 mg, 0.018 mmol, 0.4 eq.) were placed in a MW reactor vessel in DMF (500 μl). The mixture was heated under MW irradiation (250 W) at 90 °C for 20 min. Three more batches were carried out following the same procedure and then filtered and combined (25% conversion, estimated using HPLC-MS). A pure fraction of bicyclopeptide **12** was isolated using semipreparative RP-HPLC.

## Additional information

**How to cite this article:** Mendive-Tapia, L. *et al*. New peptide architectures through C–H activation stapling between tryptophan–phenylalanine/tyrosine residues. *Nat. Commun.* 6:7160 doi: 10.1038/ncomms8160 (2015).

## Supplementary Material

Supplementary Information Supplementary Figures 1-148, Supplementary Tables 1-40, Supplementary Methods and Supplementary References

## Figures and Tables

**Table 1:** Results for the stapled bond formation of peptides **1** under microwave irradiation.

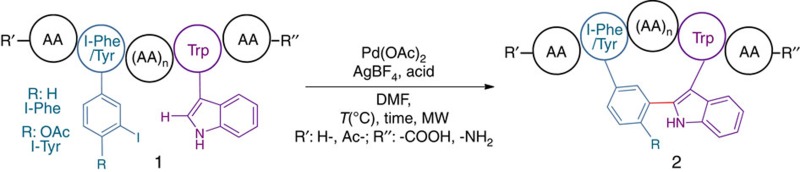					
**Entry**	***n***	**Linear peptide (1)**	Coupling conditions*	**Stapled peptide**	**Conv (%)**†
1	1	Ac-Ala-***m*****-I-Phe**-Ala-**Trp**-Ala-OH **(1a)**	A	2a	38
2	2	Ac-Ala-***m*****-I-Phe**-Ala-Ala-**Trp**-Ala-OH **(1b)**	A	2b	100
3	3	Ac-Ala-***m*****-I-Phe**-Ala-Ala-Ala-**Trp**-Ala-OH **(1c)**	A	2c	100
4	1	Ac-Ala-***m*****-I-Tyr**-Ala-**Trp**-Ala-OH **(1d)**	A	2d	100
5	2	Ac-Ala-***m*****-I-Tyr**-Ala-Ala-**Trp**-Ala-OH **(1e)**	A	2e	100
6	3	Ac-Ala-***m*****-I-Tyr**-Ala-Ala-Ala-**Trp**-Ala-OH **(1f)**	A	2f	100
7	3	Ac-***m*****-I-Phe**-Asn-Gly-Arg-**Trp**-NH_2_ **(1g)**	B	2g	77
8	3	Ac-***m*****-I-Phe**-Arg-Gly-Asp-**Trp**-NH_2_ **(1h)**	B	2h	70
9	2	H-Ala-***m*****-I-Phe**-Ser-Ala-**Trp**-Ala-OH **(1i)**	B	2i	39‡
10	1	Ac-Ala-***m*****-I-Phe**-Val-**Trp**-Ala-OH **(1j)**	B	2j	71
11	0	Ac-Ala-***p*****-I-Phe**-**Trp**-Ala-OH **(1k)**	B	2k§	60

Conv, conversion; HPLC–MS, high-performance liquid chromatography–mass spectrometry; MW, microwave.

^*^Coupling conditions (A): 5 mol % Pd(OAc)_2_, 1.0 eq. of AgBF_4_, 1.5 eq. of 2-NO_2_BzOH in DMF:PBS (1:1), MW 80 °C, 15 min. (B) 5 mol % Pd(OAc)_2_, 2.0 eq. of AgBF_4_, 1.0 eq. of TFA in DMF, MW 90 °C, 20 min.

^†^Conversion: estimated yield (HPLC-MS).

^‡^Additional MW irradiation cycles were necessary to obtain the desired product as the main compound (HPLC-MS).

^§^Cyclodimer **2k** was obtained in place of the putative monomeric structure **2k′**.
