# Supplementary material for: New peptide architectures through C–H activation stapling between tryptophan–phenylalanine/tyrosine residues
Source: Nat Commun. 2015 May 21;6:7160. doi: 10.1038/ncomms8160 (PMC4455059; doi:10.1038/ncomms8160)
Supplement: Supplementary Information — Supplementary Figures 1-148, Supplementary Tables 1-40, Supplementary Methods and Supplementary References [file ncomms8160-s1.pdf]

# Supplementary Figures

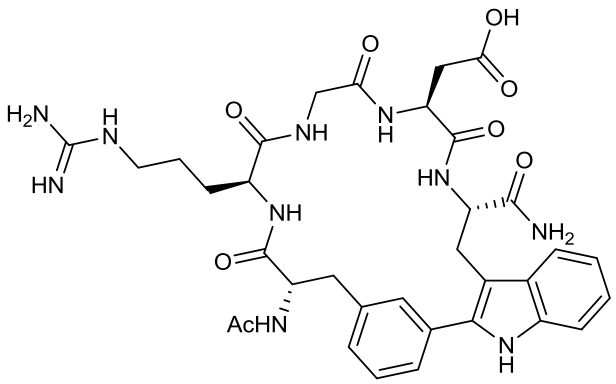

**2h**

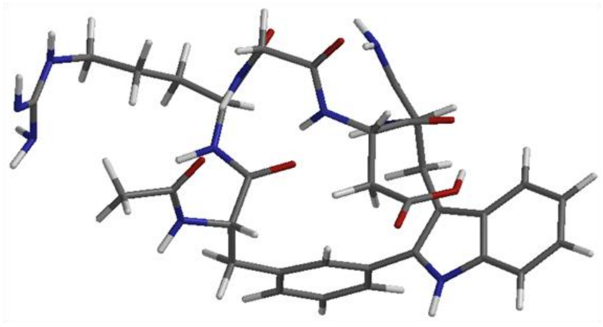

**2h**

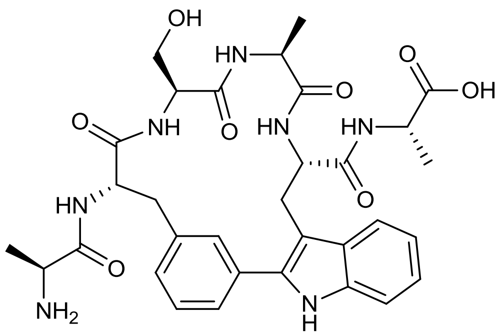

**2i**

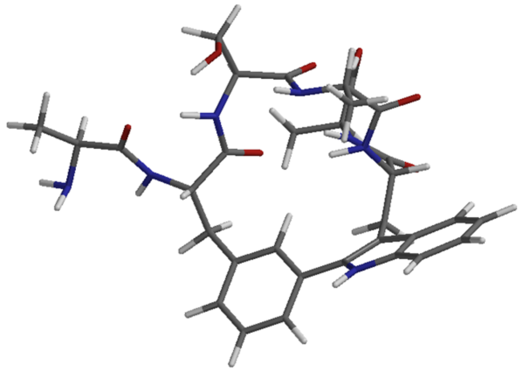

**2i**

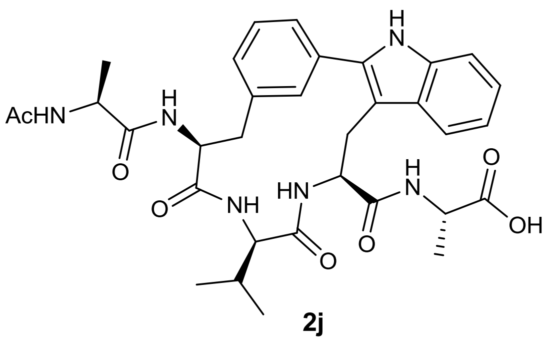

**2j**

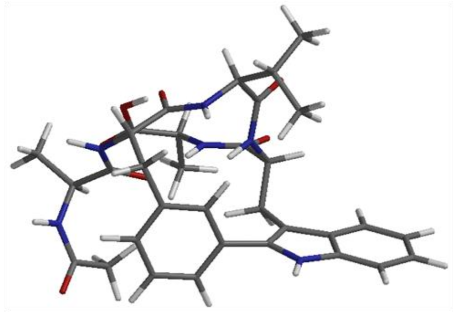

**2j**

**Supplementary Figure 1 | Minimized geometries of compounds 2h-j generated by the Spartan ’14 suite (molecular mechanics, MMFF94).[8]**

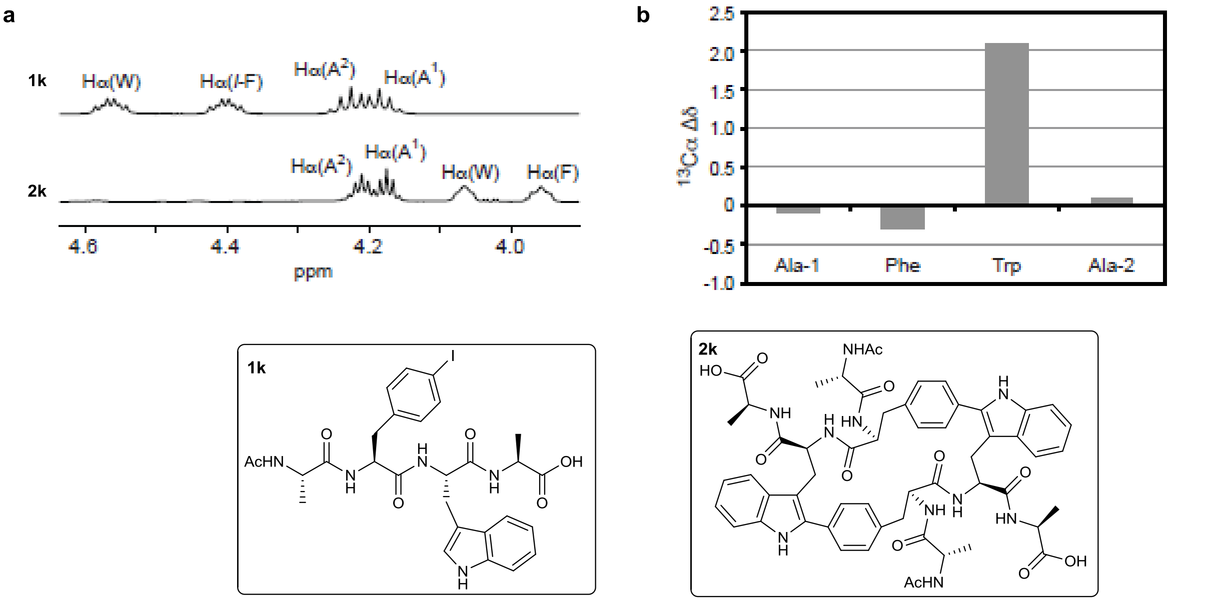


**Supplementary Figure 2 | Peptide NMR spectra comparison between compounds 2k and 1k. a,** NMR H[illegible]region of peptide **2k** and its linear precursor **1k**. **b,** Plot of the $^{13}C_{\alpha}$ chemical shift differences ($^{13}C_{\alpha}$ $\Delta\delta_{cyclic\text{-}linear}$) between cyclodimer **2k** and its linear counterpart **1k**. Temperature coefficients of the NH amide protons, $\Delta\delta/\Delta T$ (ppb/ºK), were -3.5 ($A^1$), -2.8 (*I*-F), -3.8 (W), -5.9 ($A^2$) and -4.1 ($A^1$), -5.6 (F), 0.7 (W), -5.3 ($A^2$) for peptide **1k** and **2k**, respectively.

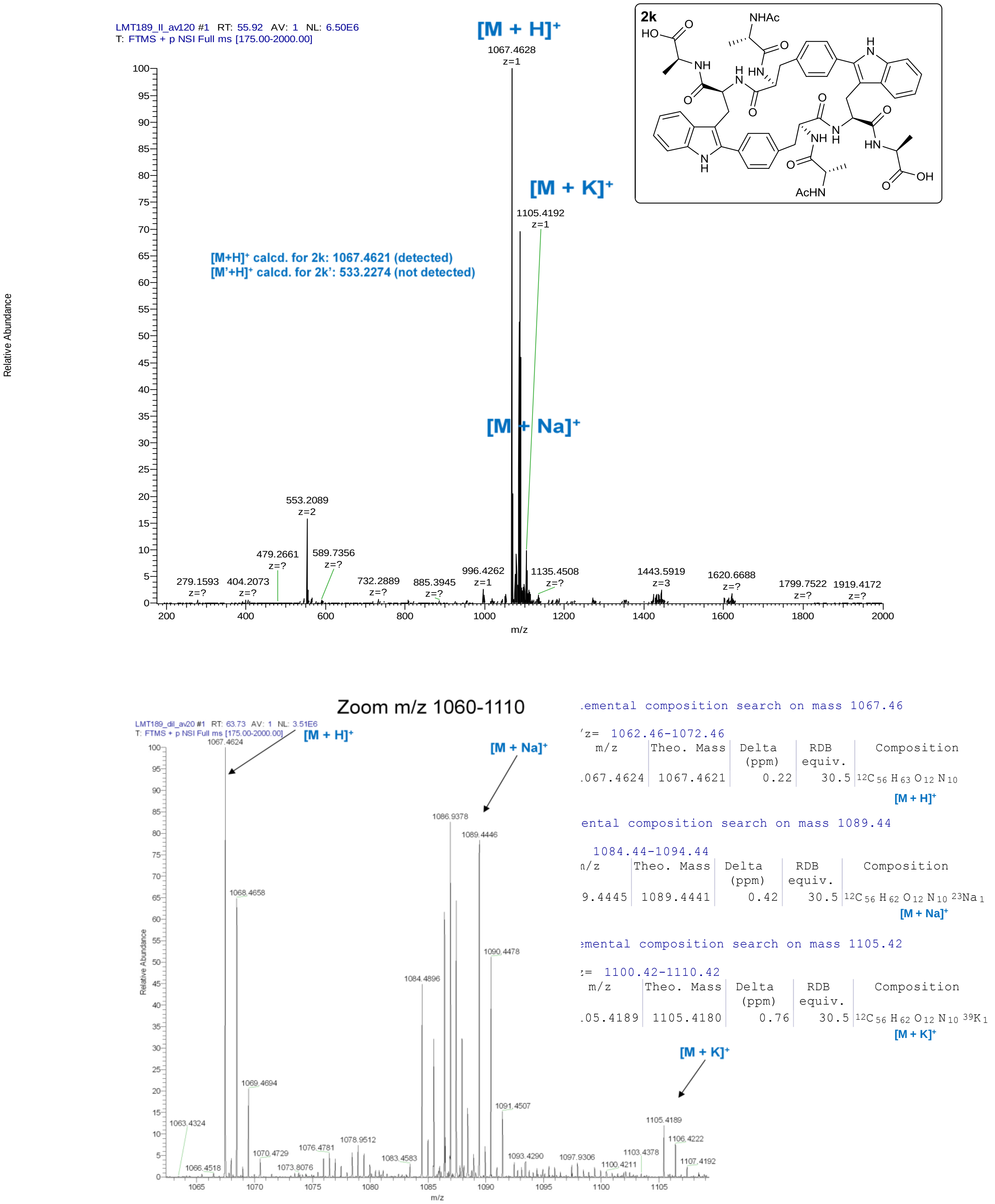


**Supplementary Figure 3 | High resolution mass spectrometry analysis of compound 2k.** Sample was reconstructed in 100 µL of ACN and diluted 1/100 in $H_2O$/ACN (1:1) 1% formic acid for MS analysis. **HRMS** (ESI) (m/z); found, 1067.4624. $[M+H]^+$ calcd. for compound **2k'** ($C_{28}H_{31}N_5O_6$), 533.2274; not found.

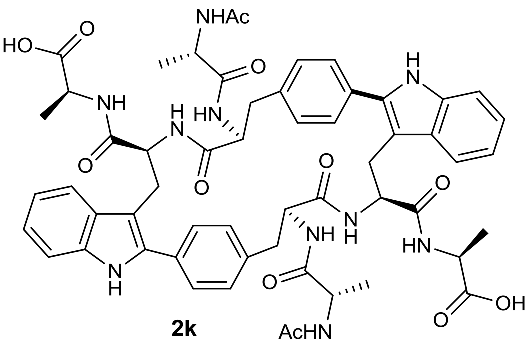


**2k**

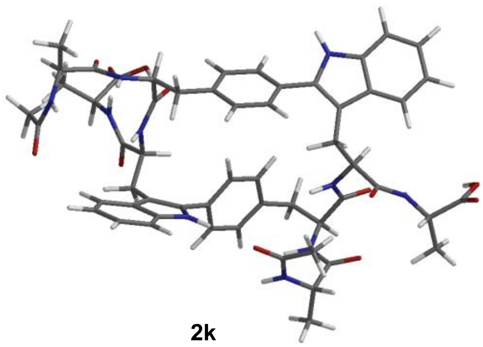

**2k**

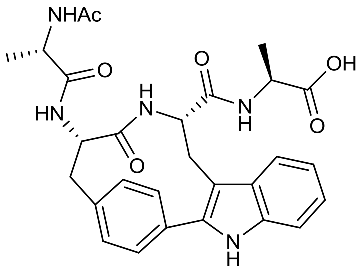


**2k' (not observed)**

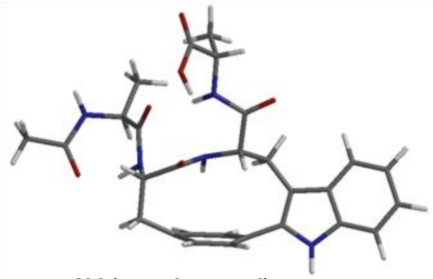

**2k' (not observed)**

**Supplementary Figure 4 | Minimized geometries of compound 2k (left) and its monomeric analog 2k' (right) generated by the Spartan '14 suite.**[8] Structure **2k** display planar phenyl rings in contrast with the constrained monomeric structure **2k'**, where a phenyl ring is severely distorted.

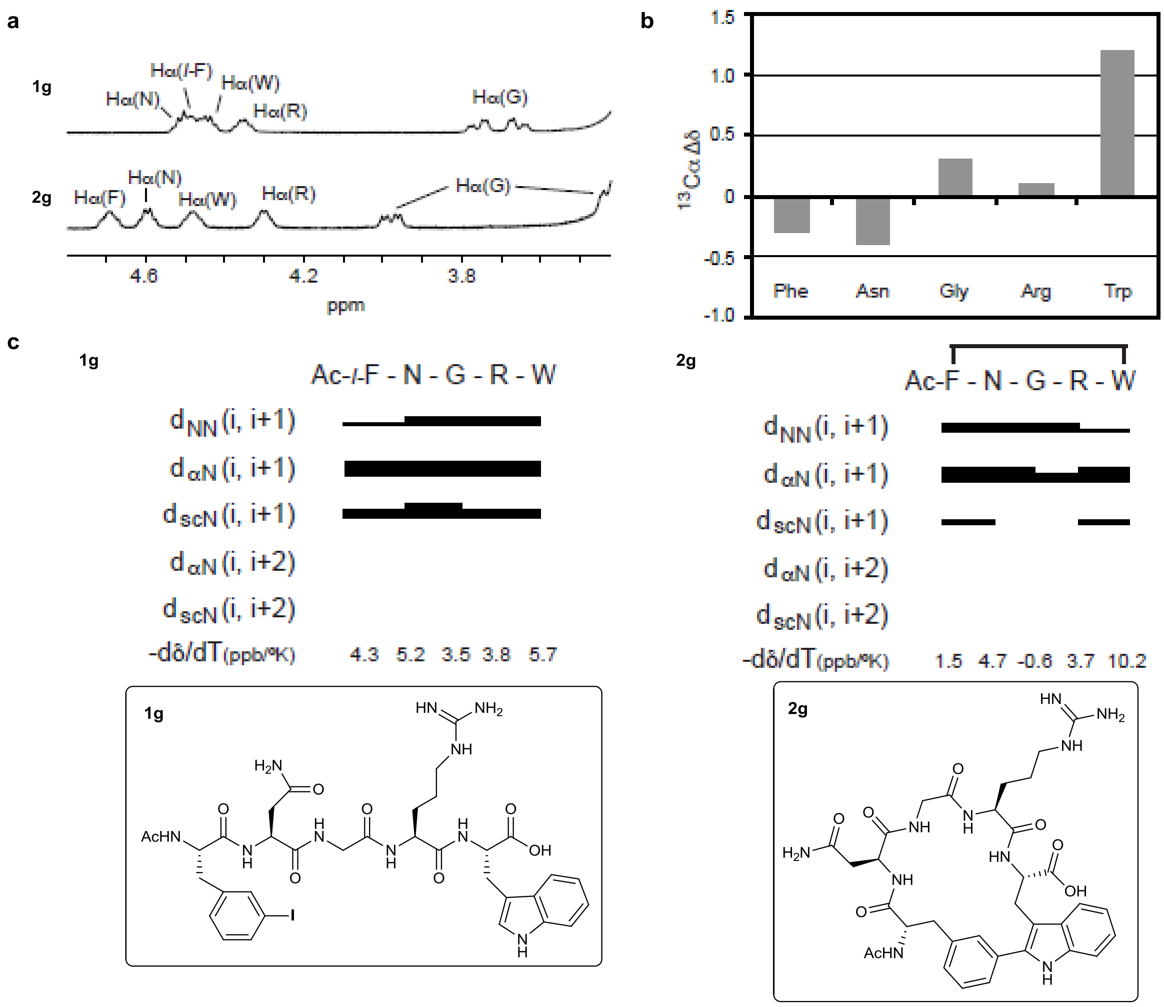


**Supplementary Figure 5 | Peptide NMR spectra comparison between compounds 2g and 1g. a,** NMR $H_\alpha$ region of peptide **2g** and its linear precursor **1g**. **b,** Plot of the $^{13}C_\alpha$ chemical shift differences ($^{13}C_\alpha\ \Delta\delta_{cyclic\text{-}linear}$) between stapled peptide **2g** and its linear counterpart **1g**. **c,** Summary of NOE connectivities and temperature coefficients of the NH amide protons ($\Delta\delta/\Delta T$) of peptide **1g** (bottom left) and **2g** (bottom right). The thickness of the bars reflects the intensity of the NOEs, i.e. weak ( ▬ ), medium ( ■ ) and strong ( ■ ). *I*-F: *m*-iodophenylalanine.

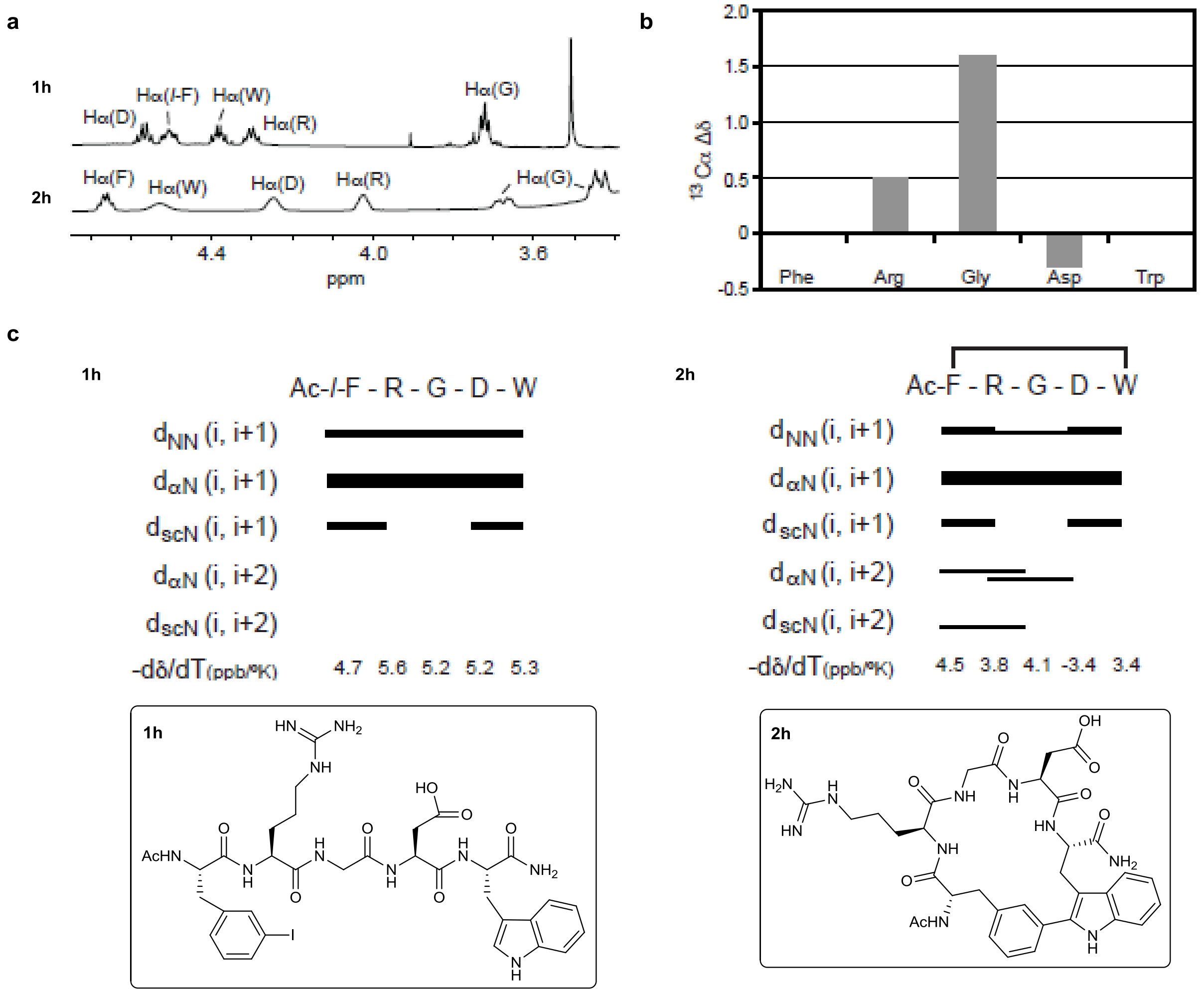


**Supplementary Figure 6 | Peptide NMR spectra comparison between compounds 2h and 1h. a,** NMR $H_\alpha$ region of peptide **2h** and its linear precursor **1h**. **b,** Plot of the $^{13}C_\alpha$ chemical shift differences ($^{13}C_\alpha$ $\Delta\delta_{cyclic-linear}$) between stapled peptide **2h** and its linear counterpart **1h**. **c,** Summary of NOE connectivities and temperature coefficients of the NH amide protons ($\Delta\delta/\Delta T$) of peptide **1h** (bottom left) and **2h** (bottom right). The thickness of the bars reflects the intensity of the NOEs, i.e. weak ( ▬ ), medium ( ▬ ) and strong ( ▬ ). *I*-F: *m*-iodophenylalanine.

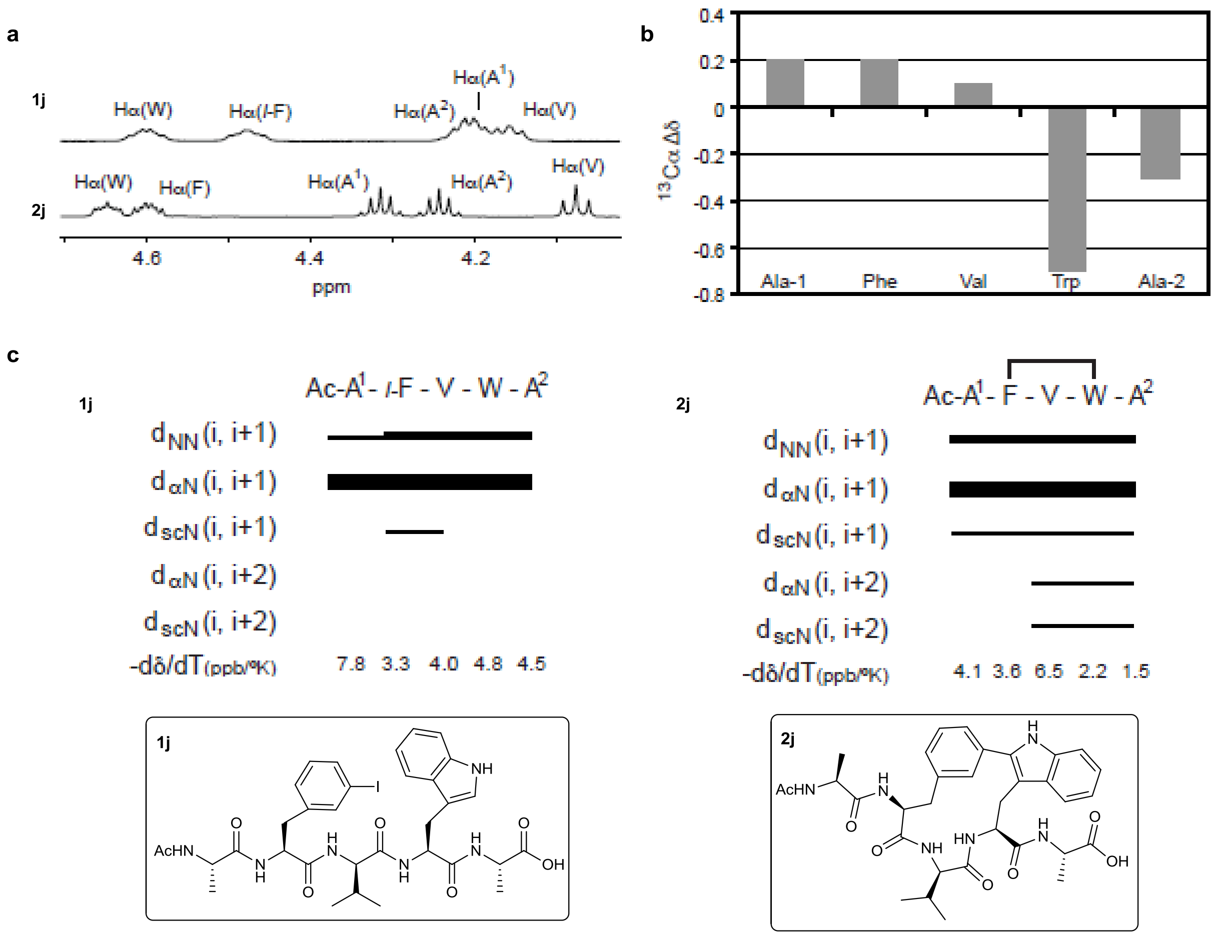


**Supplementary Figure 7 | Peptide NMR spectra comparison between compounds 2j and 1j. a,** NMR $H_{\alpha}$ region of peptide **2j** and its linear precursor **1j**. **b,** Plot of the $^{13}C_{\alpha}$ chemical shift differences ($^{13}C_{\alpha}$ $\Delta\delta_{cyclic\text{-}linear}$) between stapled peptide **2j** and its linear counterpart **1j**. **c,** Summary of NOE connectivities and temperature coefficients of the NH amide protons ($\Delta\delta/\Delta T$) of peptide **1j** (bottom left) and **2j** (bottom right). The thickness of the bars reflects the intensity of the NOEs, i.e. weak ( ▬ ), medium ( ▬ ) and strong ( ▬ ). *I*-F: *m*-iodophenylalanine.

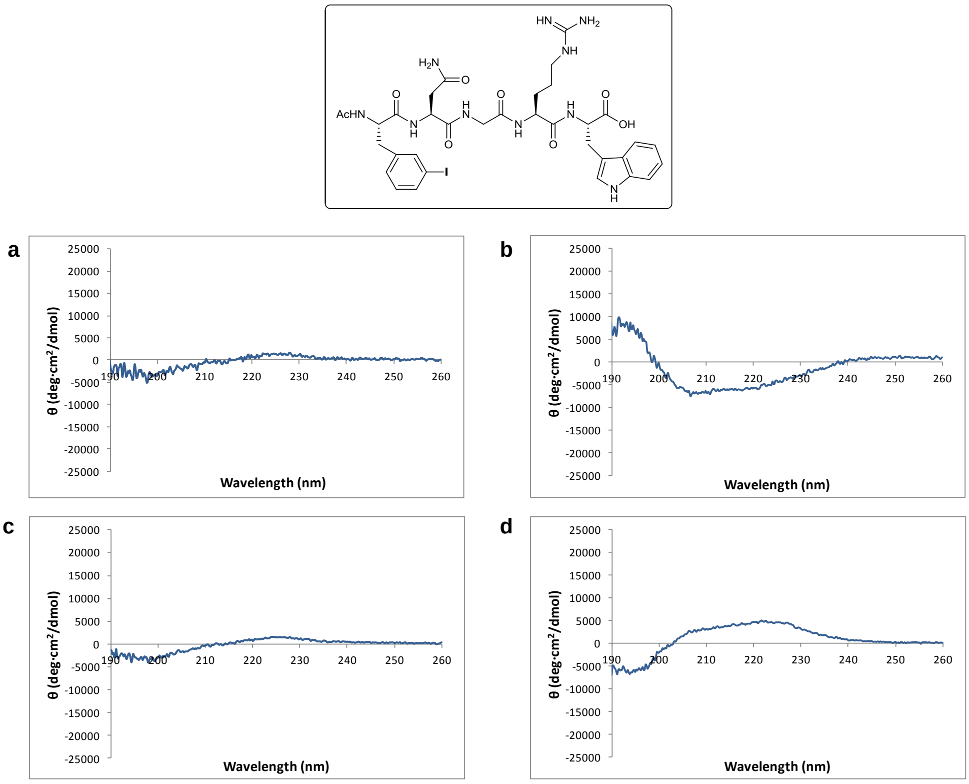


**Supplementary Figure 8 | Circular dichroism spectra of 1g**. **a,** At 100 µM in a buffer of 25 mM $Na_2HPO_4$ (pH 7). **b,** At 100 µM in a buffer of 25 mM $Na_2HPO_4$ (pH 7) (90%) and 10% of TFE. **c,** At 200 µM in a buffer of 25 mM $Na_2HPO_4$ (pH 7). **c,** At 200 µM in PBS (90%) and TFE (10%).

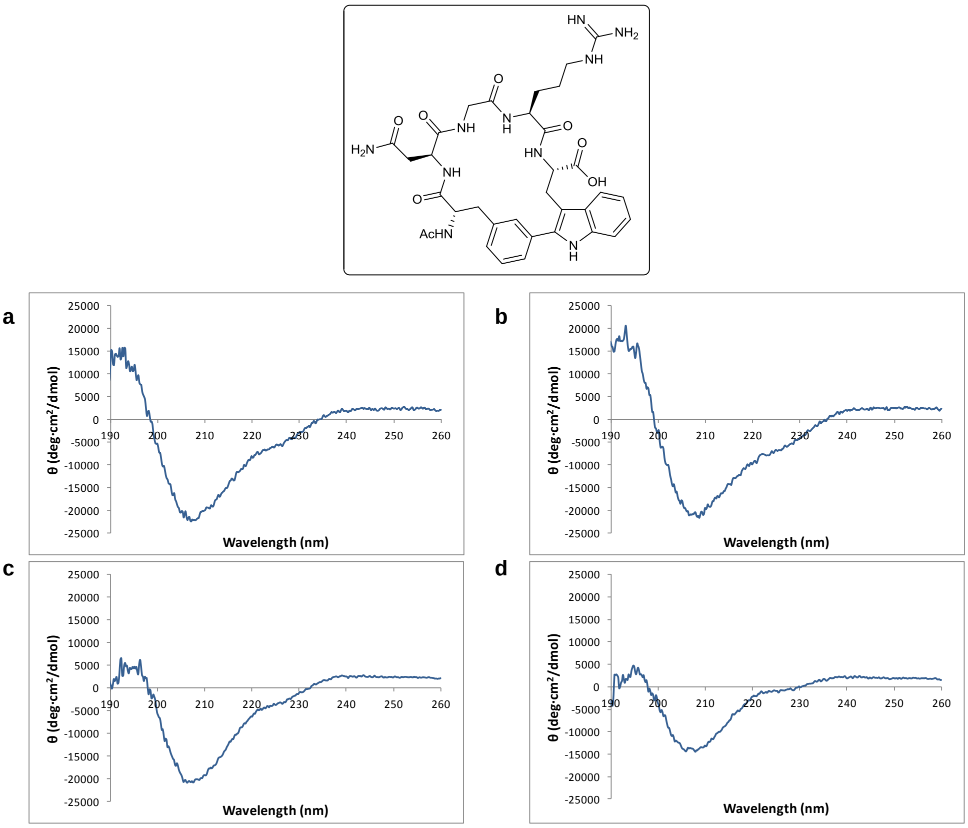


**Supplementary Figure 9 | Circular dichroism spectra of 2g**. **a,** At 100 µM in a buffer of 25 mM $Na_2HPO_4$ (pH 7). **b,** At 100 µM in a buffer of 25 mM $Na_2HPO_4$ (pH 7) (90%) and 10% of TFE. **c,** At 200 µM in a buffer of 25 mM $Na_2HPO_4$ (pH 7). **d,** At 200 µM in PBS (90%) and TFE (10%).

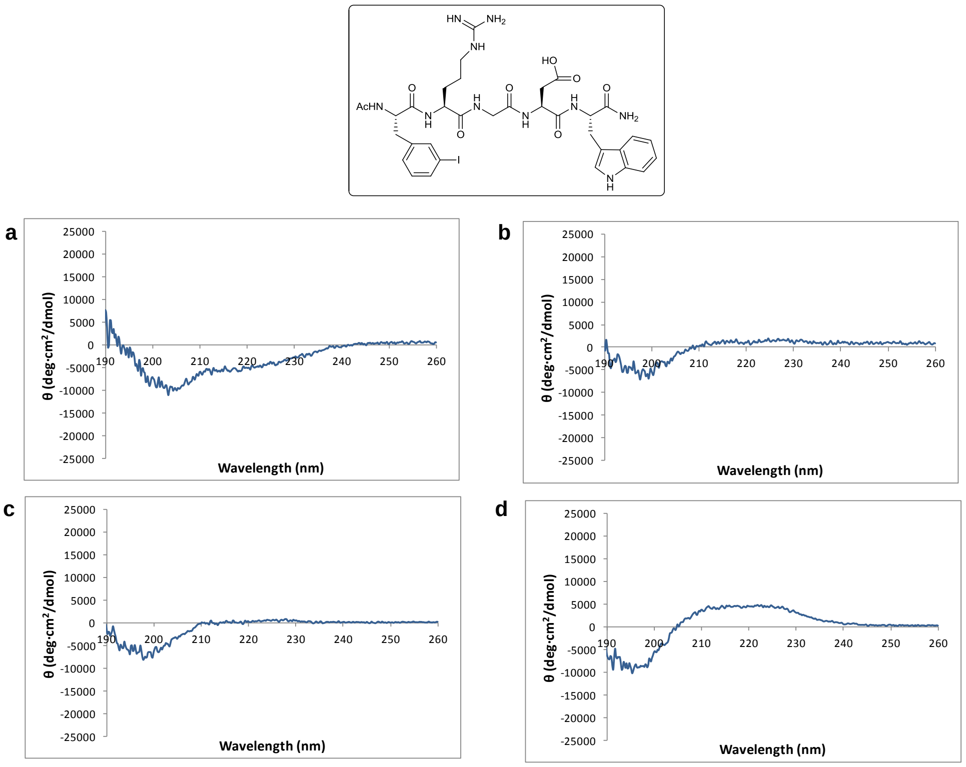


**Supplementary Figure 10 | Circular dichroism spectra of 1h**. **a,** At 100 µM in a buffer of 25 mM $Na_2HPO_4$ (pH 7). **b,** At 100 µM in a buffer of 25 mM $Na_2HPO_4$ (pH 7) (90%) and 10% of TFE. **c,** At 200 µM in a buffer of 25 mM $Na_2HPO_4$ (pH 7). **d,** At 200 µM in PBS (90%) and TFE (10%).

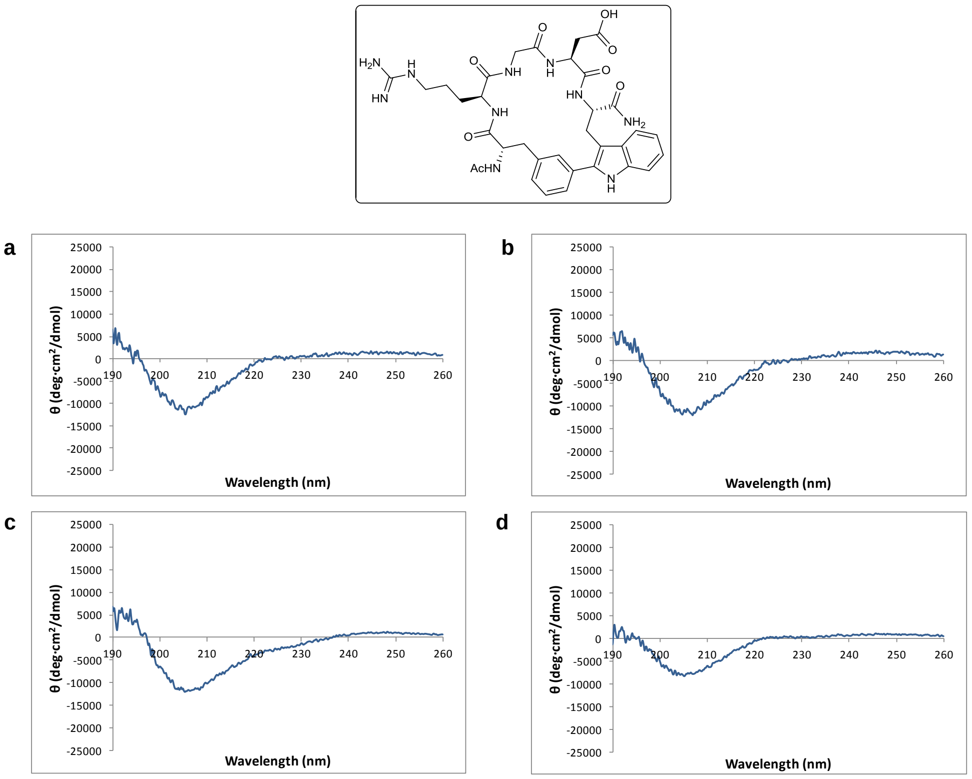


**Supplementary Figure 11 | Circular dichroism spectra of 2h**. **a,** At 100 µM in a buffer of 25 mM $Na_2HPO_4$ (pH 7). **b,** At 100 µM in a buffer of 25 mM $Na_2HPO_4$ (pH 7) (90%) and 10% of TFE. **c,** At 200 µM in a buffer of 25 mM $Na_2HPO_4$ (pH 7). **d,** At 200 µM in PBS (90%) and TFE (10%).

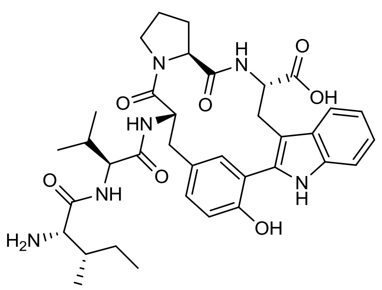

**2l**

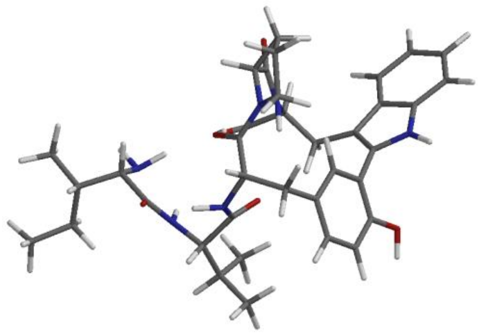

**2l**

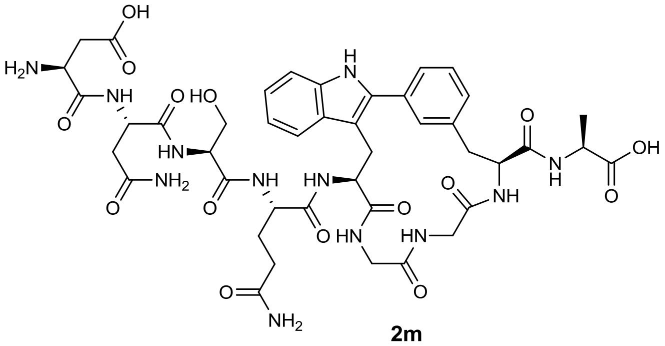

**2m**

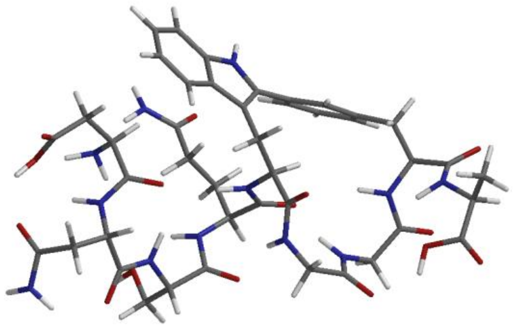

**2m**

**Supplementary Figure 12 | Minimized geometries of compounds 2l and 2m generated by the Spartan '14 suite (molecular mechanics, MMFF94).[8]**

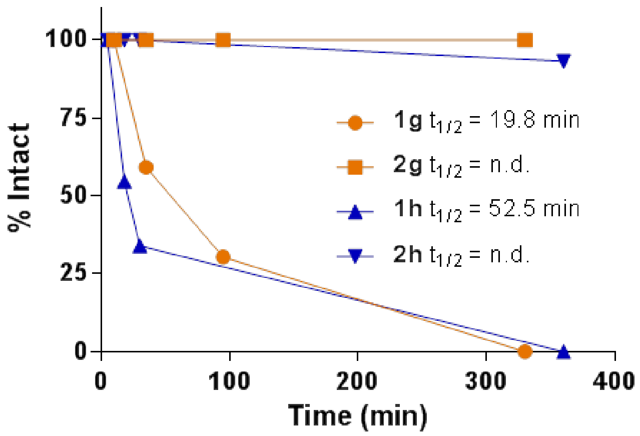


**Supplementary Figure 13 | Proteolytic degradation assay of stapled peptides 2g and 2h and their linear precursors 1g and 1h.** With respect to the linear compounds **1g** and **1h**, α-chymotrypsin cleavage products (from the hydrolysis on the *C*-terminal side of *l*-phenylalanine)[12] were observed, being the degradation complete after 6 h. Significant different behavior was observed for the corresponding stapled peptides **2g** and **2h,** which remained unaltered and only traces of the corresponding C-terminal hydrolysis of *l*-phenylalanine were detected.

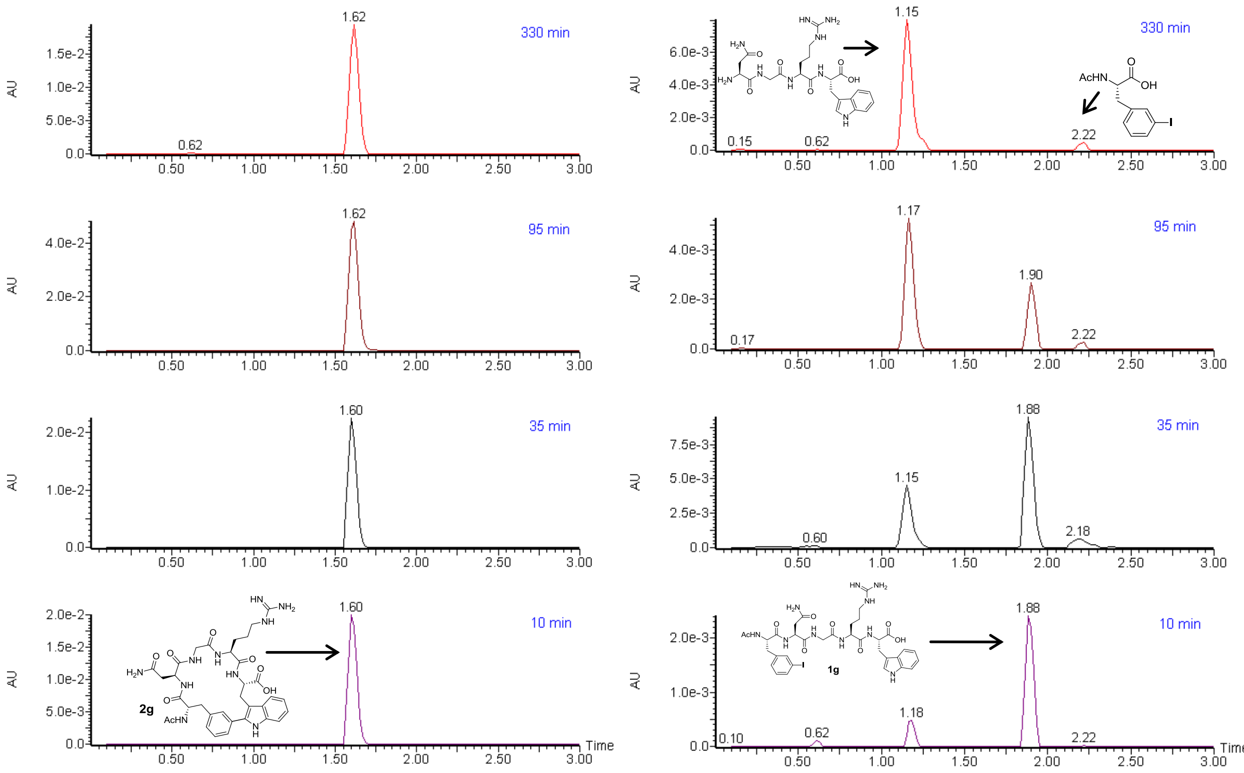

**Supplementary Figure S14 | HPLC-MS chromatograms of stapled peptide 2g (left) and its linear precursor 1g (right) monitorized by at 300 and 280 nm, respectively.**

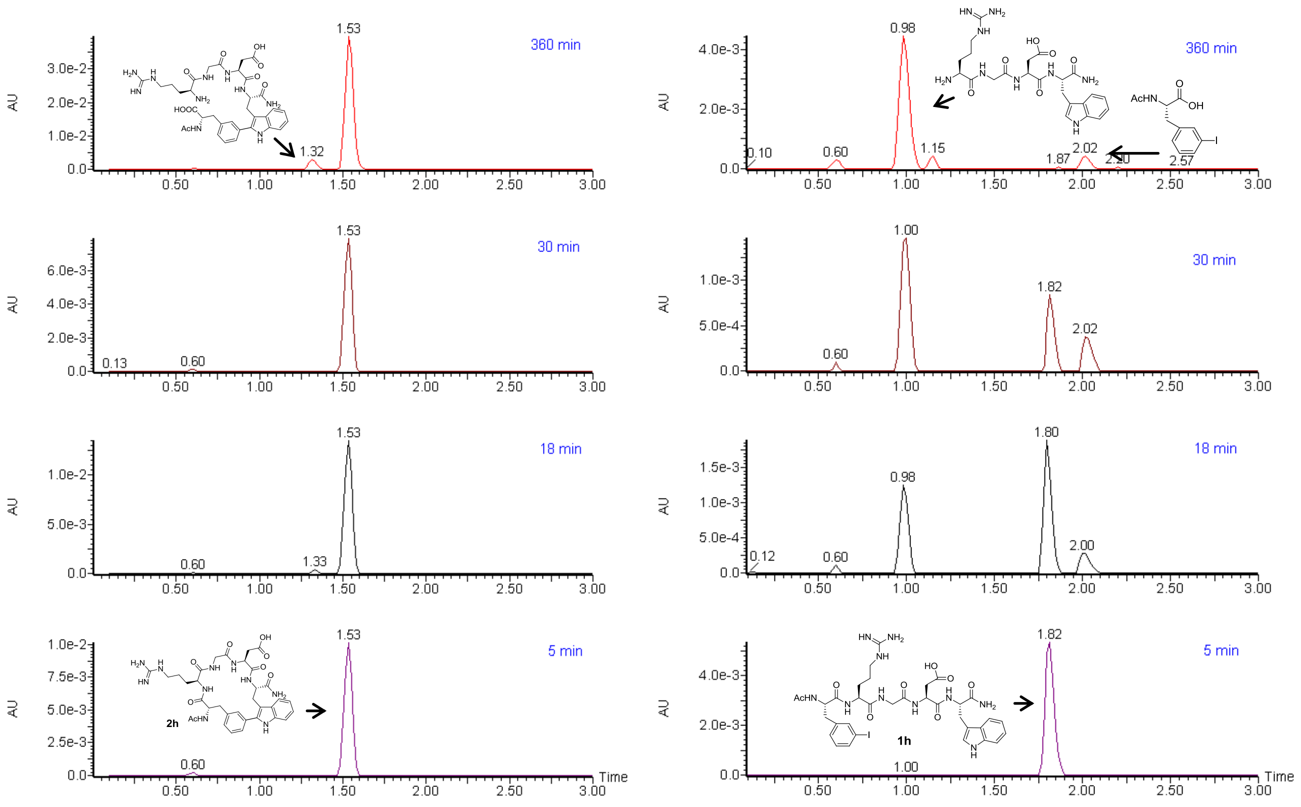

**Supplementary Figure S15 | HPLC-MS chromatograms of stapled peptide 2h (left) and its linear precursor 1h (right) monitorized at 300 and 280 nm, respectively.**

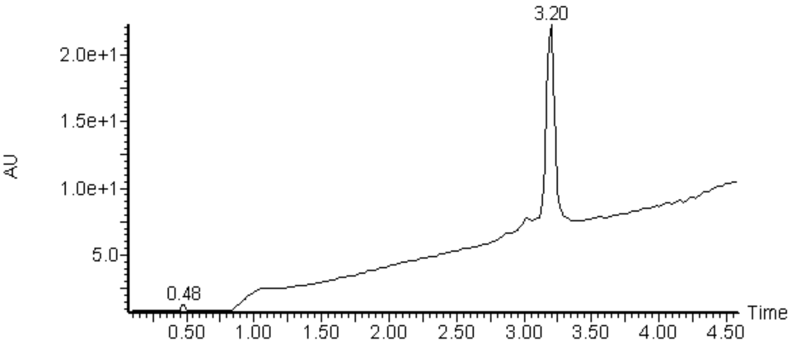


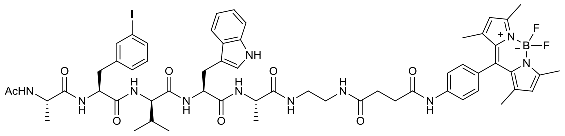

1j-Bodipy

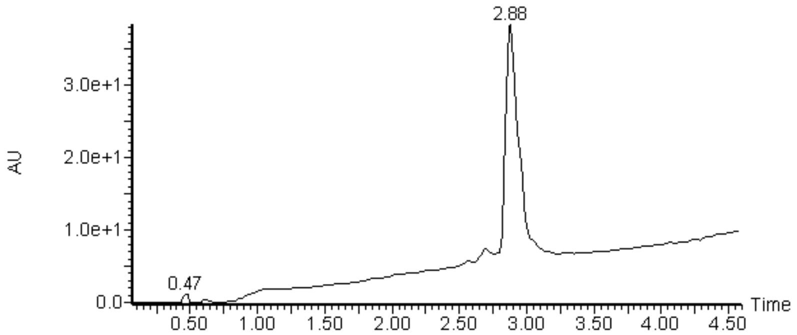


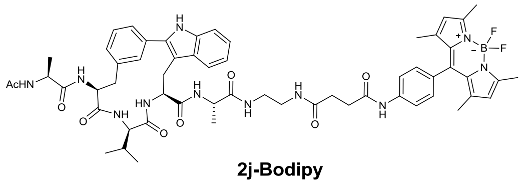

2j-Bodipy

**Supplementary Figure 16 [illegible] HPLC-MS chromatograms of BODIPY-labelled peptides 1j-BODIPY (above) and 2j-BODIPY (down).**

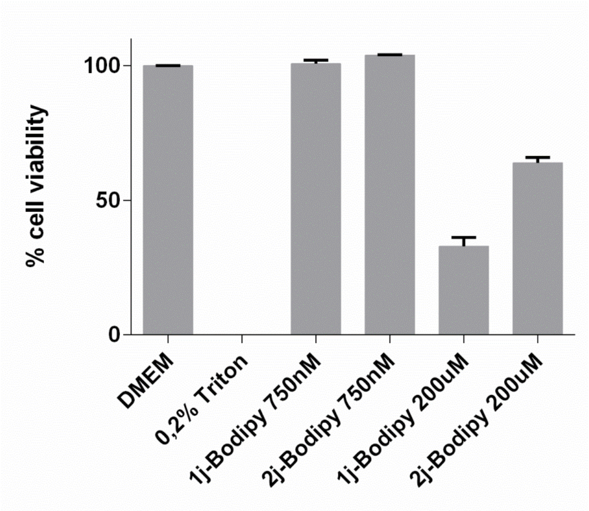


**Supplementary Figure 17 | Cytotoxicity of 1j-Bodipy and 2j-Bodipy peptides using the MTT assay.** The toxicity assay was carried out as described in the literature.[13] SH-SY5Y cells were transferred to 96-well plates (100 µL medium/well) at a density of 5000 cell/well. After 24 h, cells in triplicate were treated either with **1j-Bodipy** or **2j-Bodipy** (750 nM and 50 µM) or TRITON 0.2% as positive control for 24 h. Values are represented as means ± SD from three independent experiments.

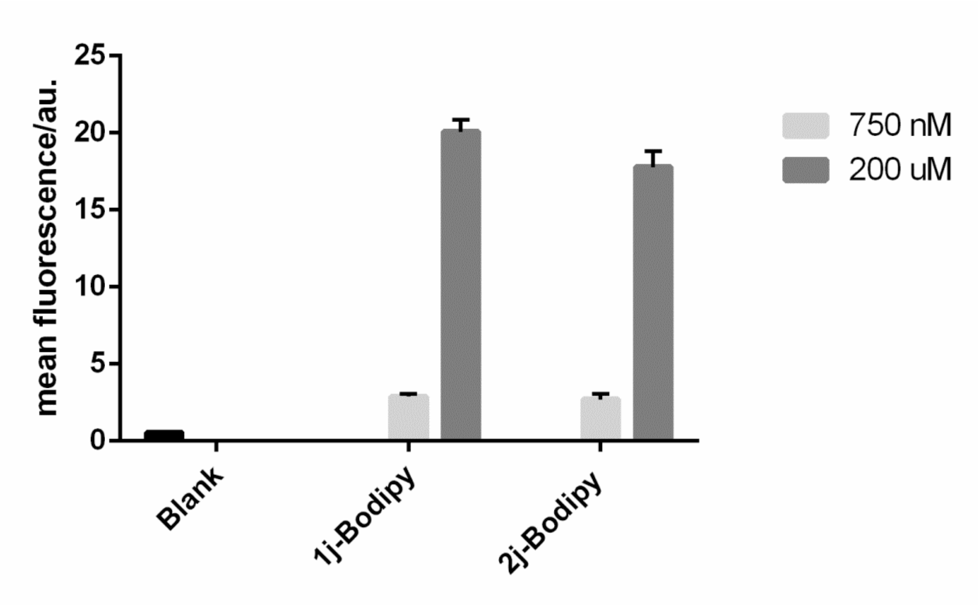


**Supplementary Figure 18 | FACS analysis of SH-SY5Y cells upon incubation with 1j-Bodipy and 2j-Bodipy.** For FACS analysis, SH-SY5Y cells were seeded on a plastic 24 well plate and cultured for 24 h. The culture medium was discarded, and cells were incubated for 30 min at 37 ºC under 5% $CO_2$ with fresh medium containing either **1j-Bodipy** or **2j-Bodipy**. Cells were then rinsed, treated with trypsin, collected, centrifuged at 4 °C, filtered and re-suspended in cold medium. Fluorescence sorting was performed with a Gallios Beckman Coulter flow cytometer.

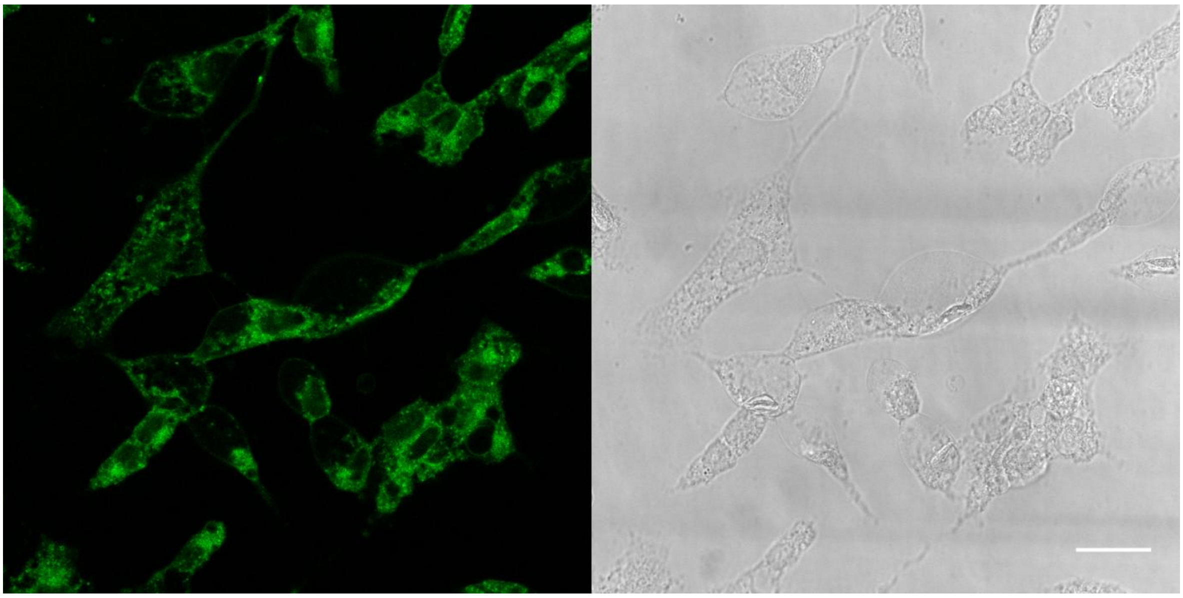

**Supplementary Figure 19 | Spontaneous internalization of 1j-Bodipy in SH-SY5Y cells (scale bar, 25 μm).**

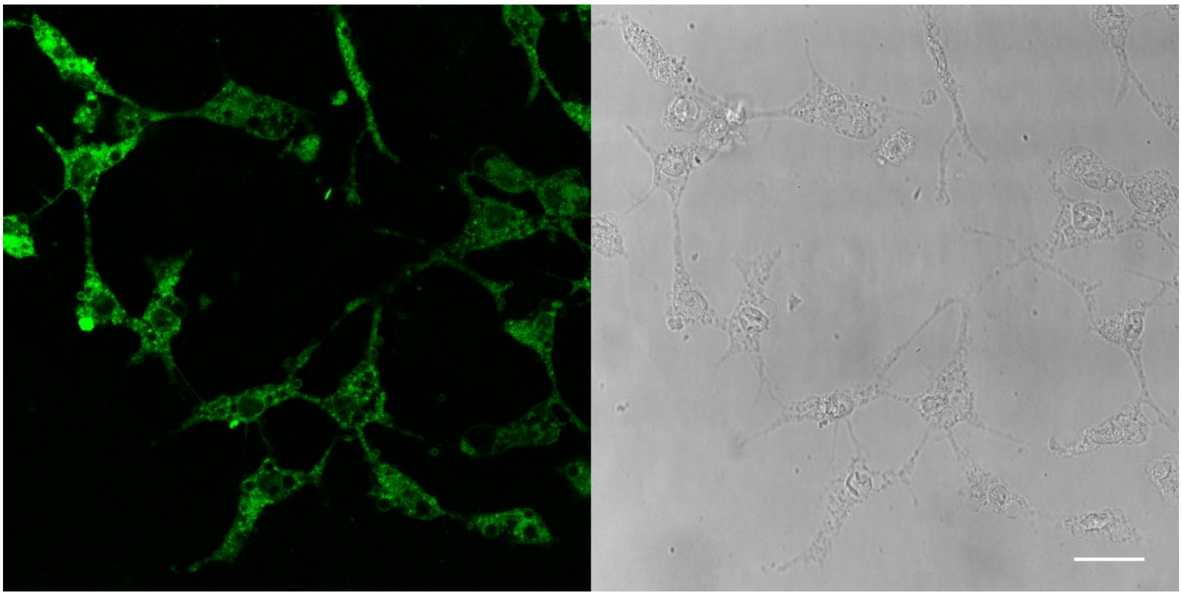

**Supplementary Figure 20 | Spontaneous internalization of 2j-Bodipy in SH-SY5Y cells (scale bar, 25 μm).**

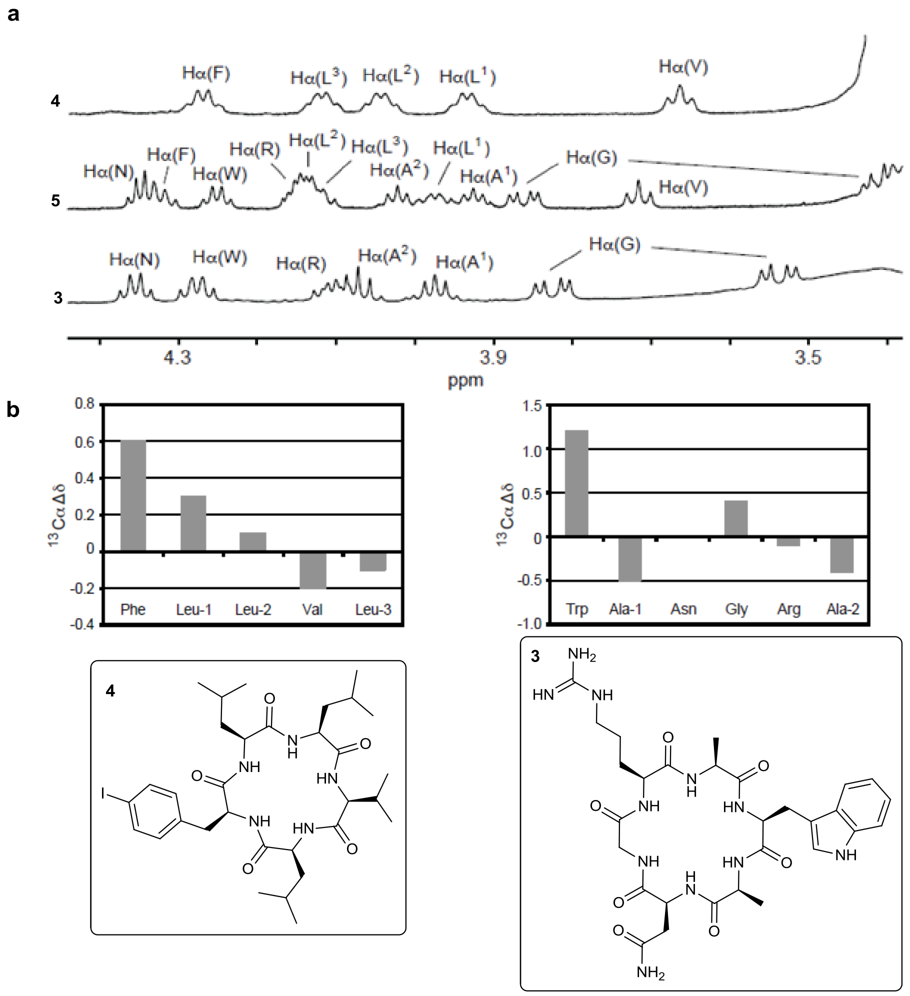


**Supplementary Figure 21 | Peptide NMR spectra comparison between compounds 3 and 4. a,** NMR $H_\alpha$ region of peptides **3, 4** and **5**. **b,** Plot of the $^{13}C_\alpha$ chemical shift differences between conjugated peptide **5** and its macrocyclic precursors **3** and **4** ($^{13}C_\alpha\ \Delta\delta_{5-x}$, where x=3 or 4).

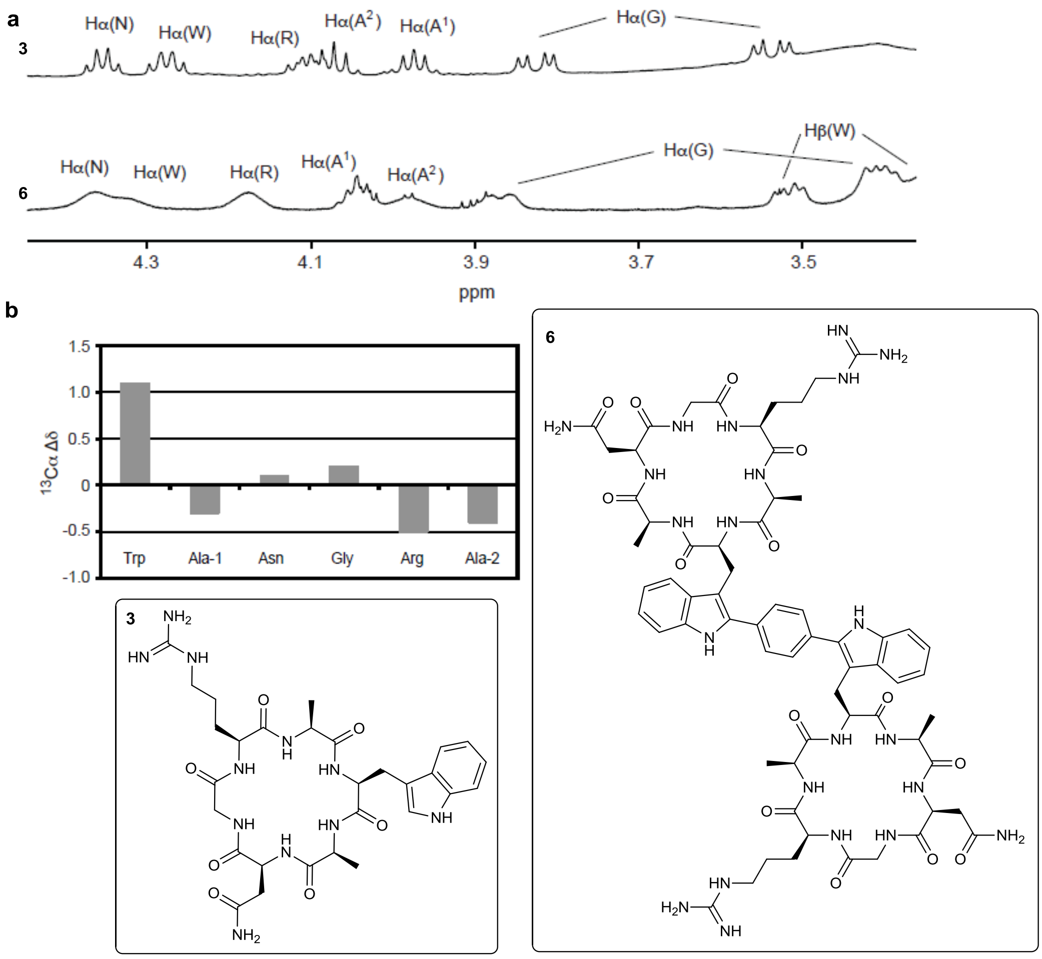


**Supplementary Figure 22 | Peptide NMR spectra comparison between compounds 3 and 6. a,** NMR $H_{\alpha}$ region of peptides **3** and **6**. **b,** Plot of the $^{13}C_{\alpha}$ chemical shift differences between conjugated peptide **6** and its macrocyclic precursor **3** ($^{13}C_{\alpha}$ $\Delta\delta_{6\text{-}3}$).

**a**

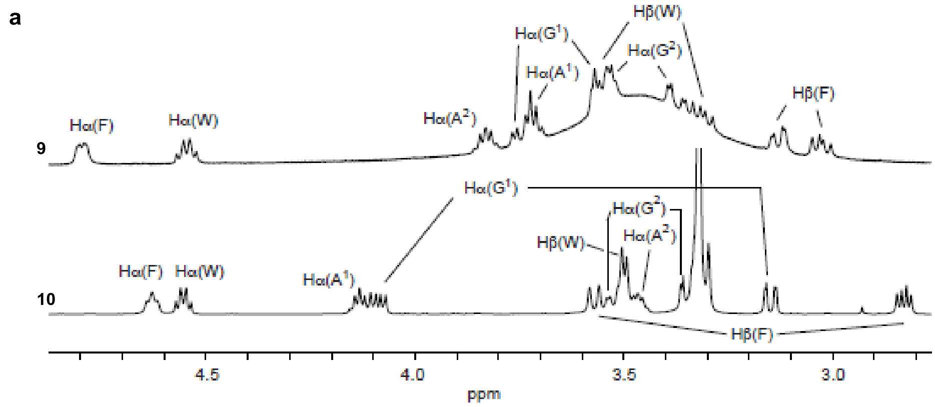


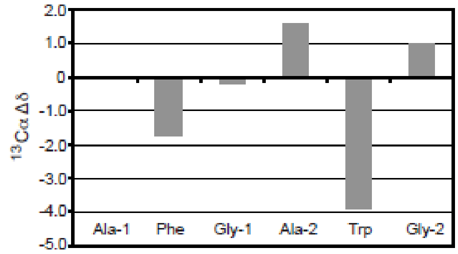


| Peptide | Δδ/ΔT (ppb/°K) | | | | | |
|---|---|---|---|---|---|---|
| | Ala-1 | Phe | Gly-1 | Ala-2 | Trp | Gly-2 |
| **9** | — | -10.1 | -4.9 | -9.5 | -1.7 | -3.4 |
| **10** | -4.1 | -2.2 | 1.0 | -3.3 | 1.7 | 0.1 |

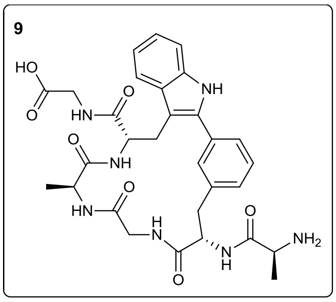


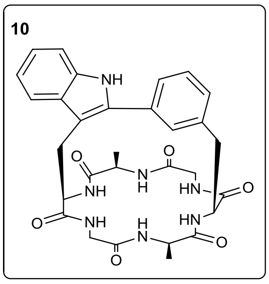


**Supplementary Figure 23 | Peptide NMR spectra comparison between compounds 9 and 10. a,** NMR $H_{\alpha}$ region of peptides **9** and **10**. **b,** Plot of the $^{13}C_{\alpha}$ chemical shift differences between peptides **9** and **10** ($^{13}C_{\alpha}$ $\Delta\delta_{10\text{-}9}$). **c,** Summary of temperature coefficients of the NH amide protons ($\Delta\delta/\Delta T$) of peptides **9** and **10**.

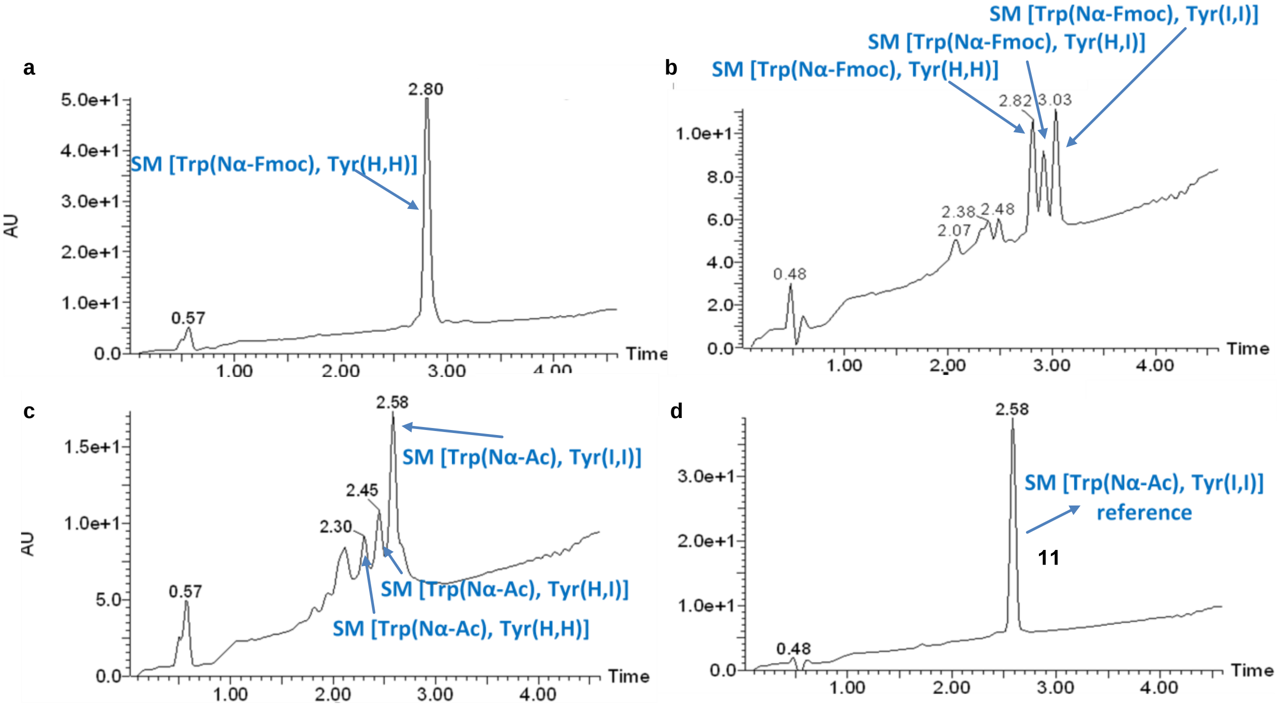


**Supplementary Figure 24 | HPLC-MS chromatograms relative to diiodination of the corresponding non-halogenated N-terminal Fmoc-protected linear sequence of 11 on resin.** In a preliminary procedure the corresponding non-halogenated linear sequence of **11** anchored to TentaGel resin provided with HMPPA linker was treated with $IPy_2BF_4$ in DCM. Although this approach is not free from byproducts relative to the halogenation of the linker, it clearly shows the feasibility of the methodology.**a,** Non-halogenated linear precursor of compound **11** obtained through routine amide couplings. **b,** On-resin iodination of the Tyr residue to yield derivative N-terminal Fmoc-protected of **11** (41% conversion). Reaction conditions: $IPy_2BF_4$ (2.2 eq.), DCM, r.t, (1 x 10 min, 1 x 50 min). **c,** On-resin iodination of the Tyr residue followed by Fmoc protecting group removal and N-terminal acetilation. Reaction conditions: (i) $IPy_2BF_4$ (4.4 eq.), DCM, r.t, (1 x 1 h), (ii) piperidine-DMF (1:4) (1 × 1 min, 2 × 5 min), (iii) DIEA (10.0 eq.), acetic anhydride (10.0 eq.),DMF, r.t,, 30 min. **d,** HPLC-MS chromatogram reference of the halogenated compound **11**. All the peptides were cleaved from the resin with TFA-DCM (95:5), r.t, 1h to be analyzed by HPLC-MS. Gradient from 5 to 100% ACN (0,1% FA).

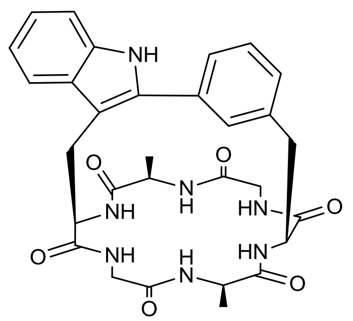

**10**

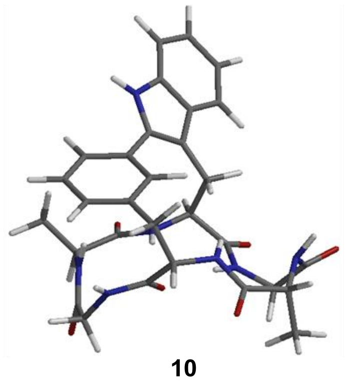

**10**

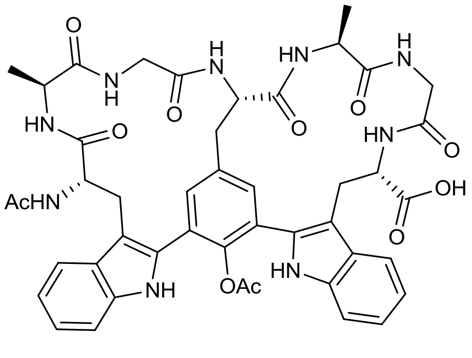

**12**

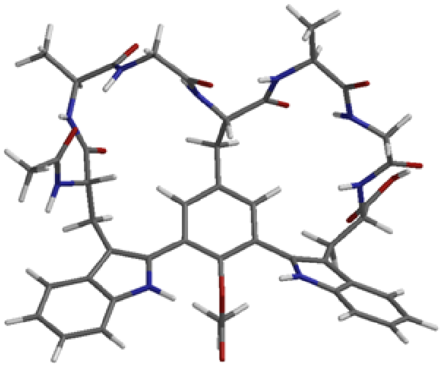

**12**

**Supplementary Figure 25 | Minimized geometries of compounds 10 and 12 generated by the Spartan ’14 suite.[8]**

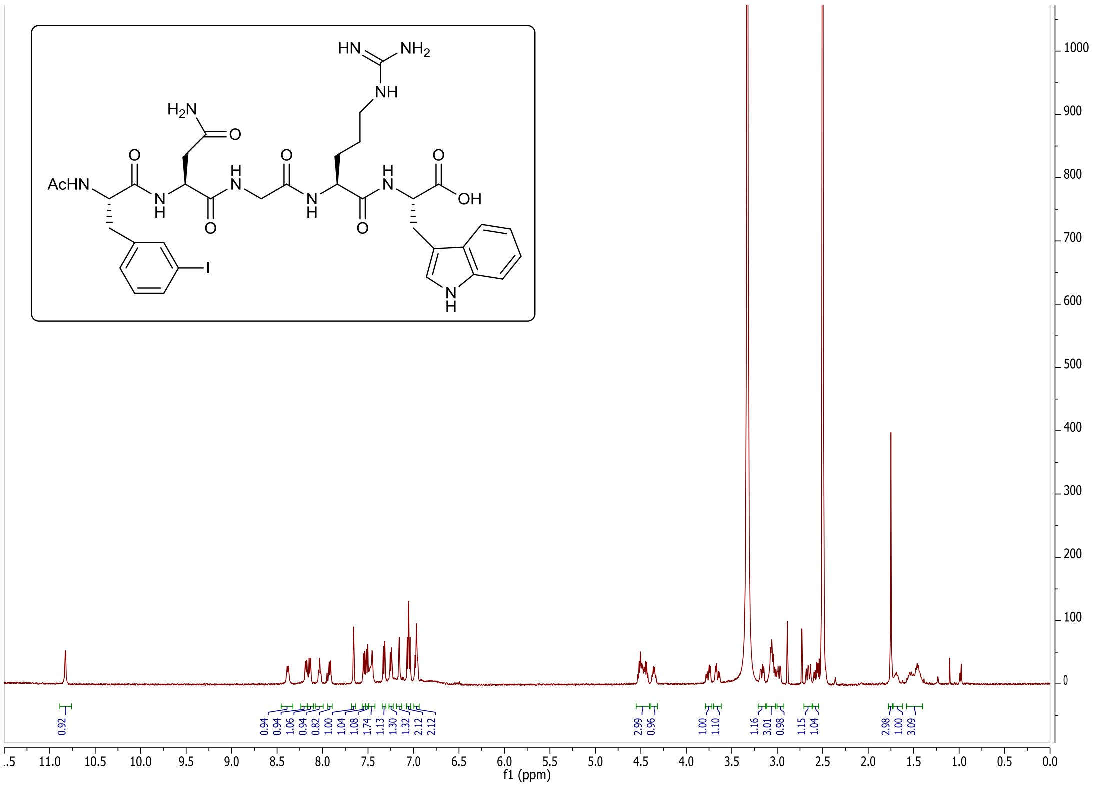

**Supplementary Figure 26 | $^{1}$H NMR spectrum of compound Ac-*m*-I-Phe-Asn-Gly-Arg-Trp-OH (1g).**

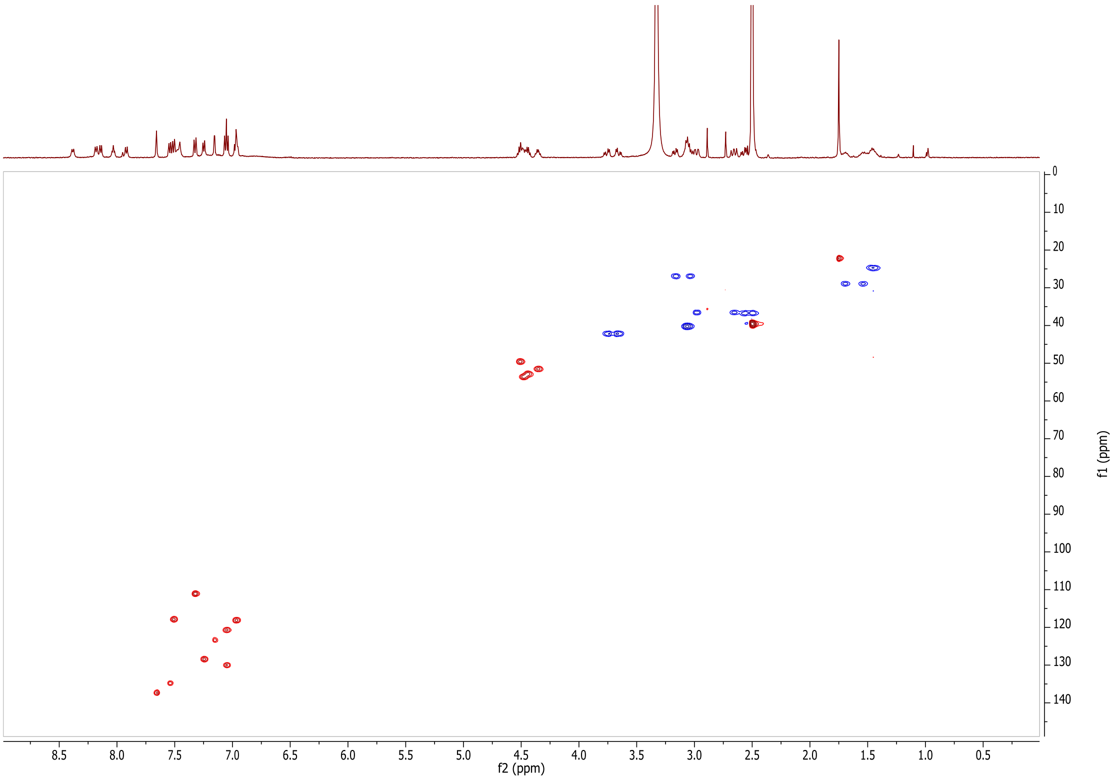

**Supplementary Figure 27 | $^{1}$H-$^{13}$C HSQC NMR spectrum of compound Ac-*m*-I-Phe-Asn-Gly-Arg-Trp-OH (1g).**

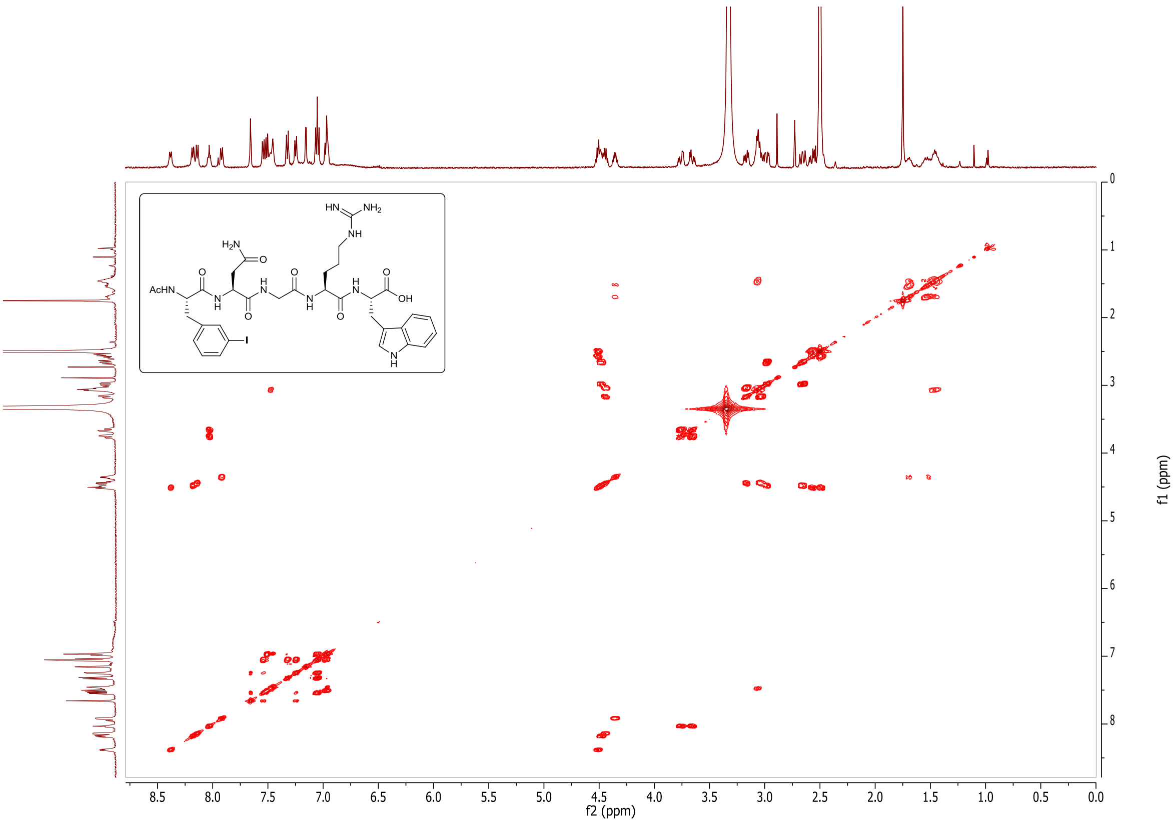


**Supplementary Figure 28 | COSY NMR spectrum of compound Ac-*m*-I-Phe-Asn-Gly-Arg-Trp-OH (1g).**

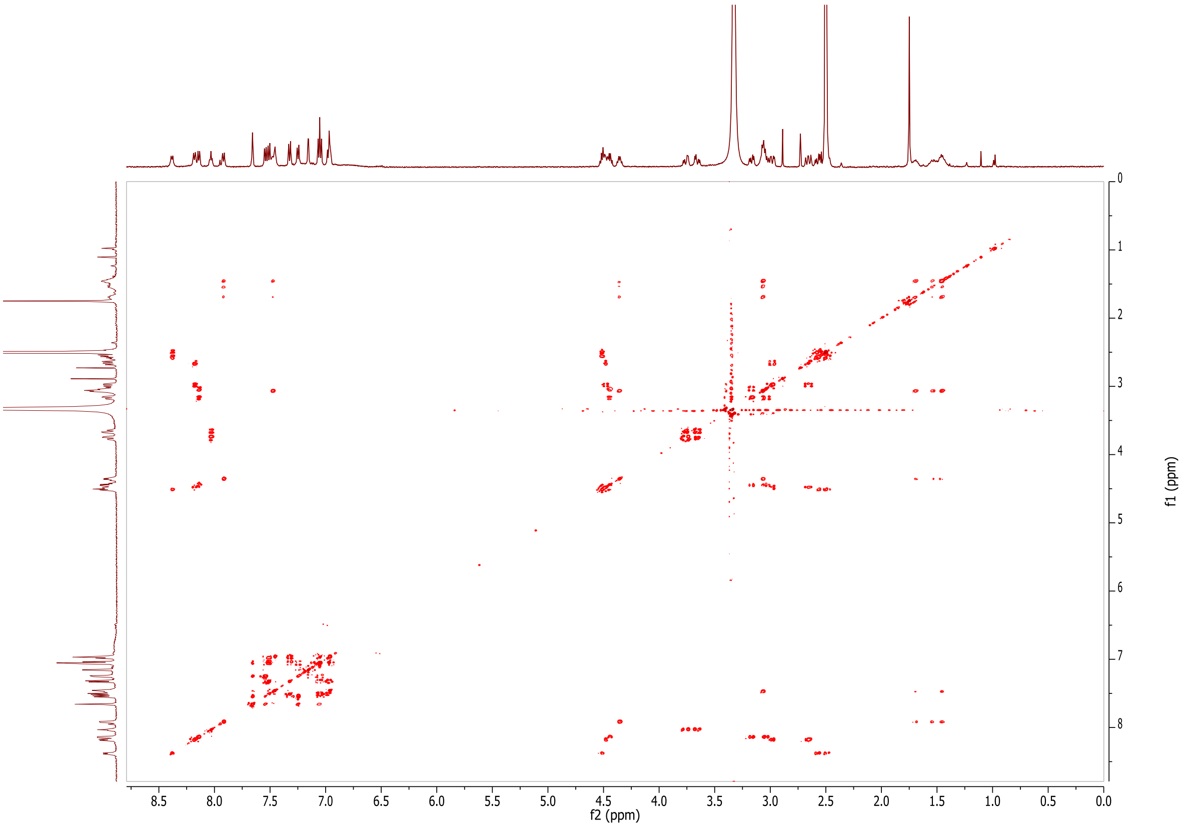


**Supplementary Figure 29 | TOCSY NMR spectrum of compound Ac-*m*-I-Phe-Asn-Gly-Arg-Trp-OH (1g).**

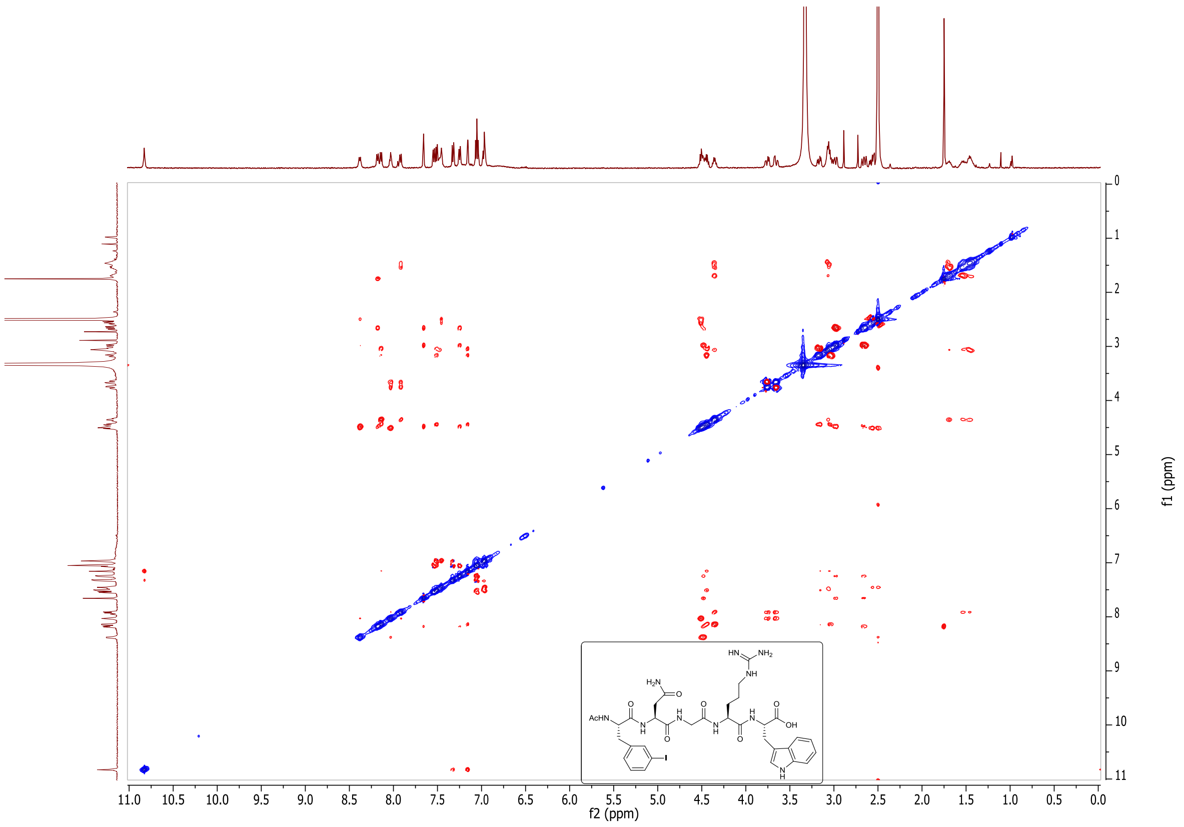


**Supplementary Figure 30 | ROESY NMR spectrum of compound Ac-*m*-I-Phe-Asn-Gly-Arg-Trp-OH (1g).**

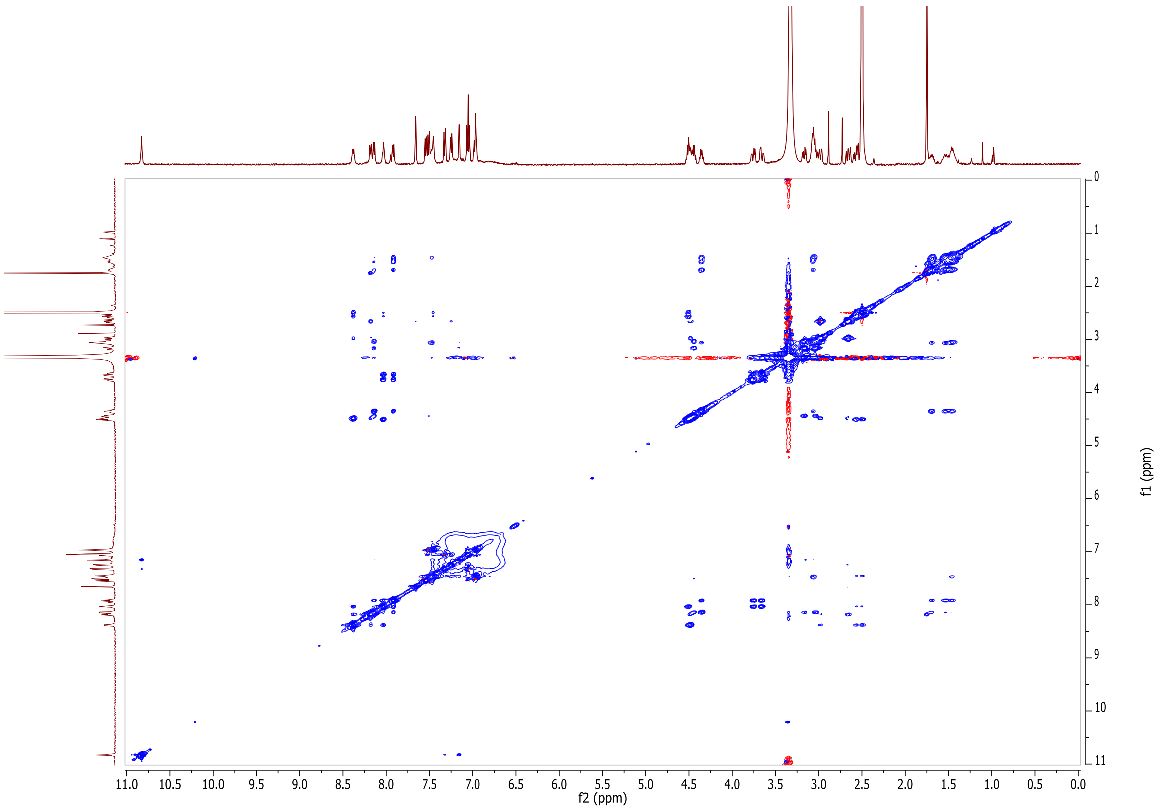


**Supplementary Figure 31 | NOESY NMR spectrum of compound Ac-*m*-I-Phe-Asn-Gly-Arg-Trp-OH (1g).**

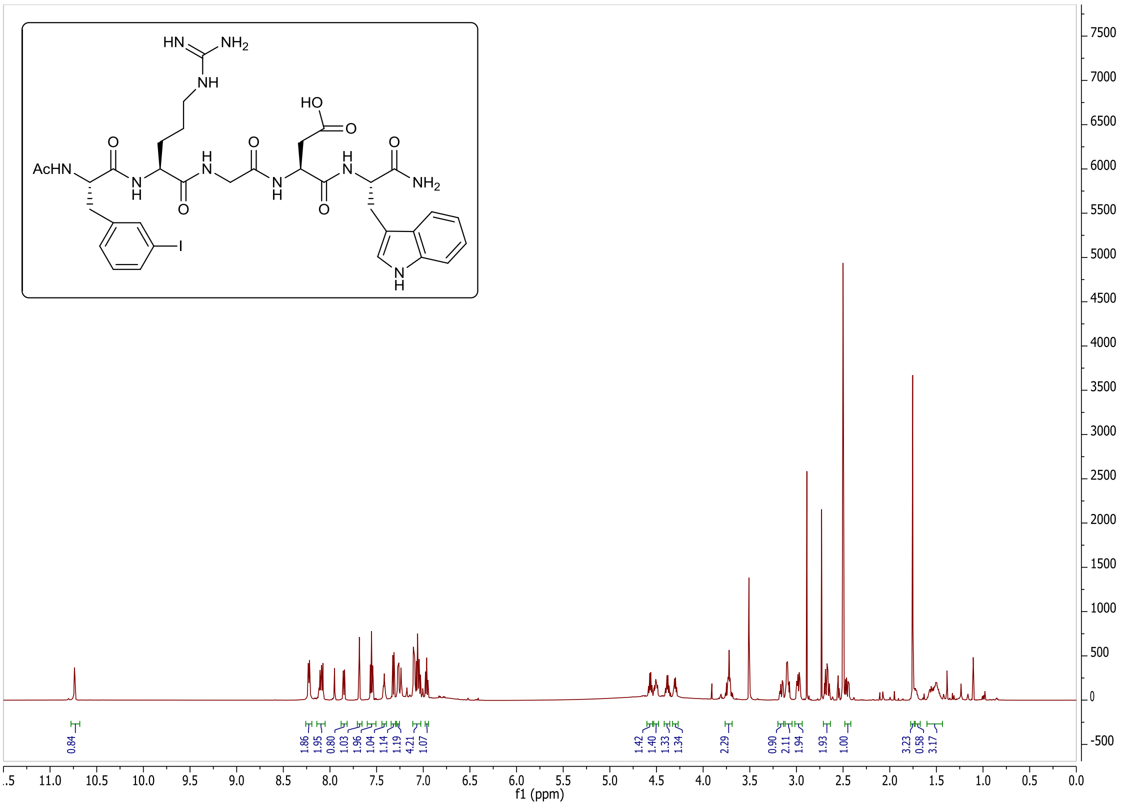


**Supplementary Figure 32 | $^{1}$H NMR spectrum of compound Ac-*m*-I-Phe-Arg-Gly-Asp-Trp-OH (1h).**

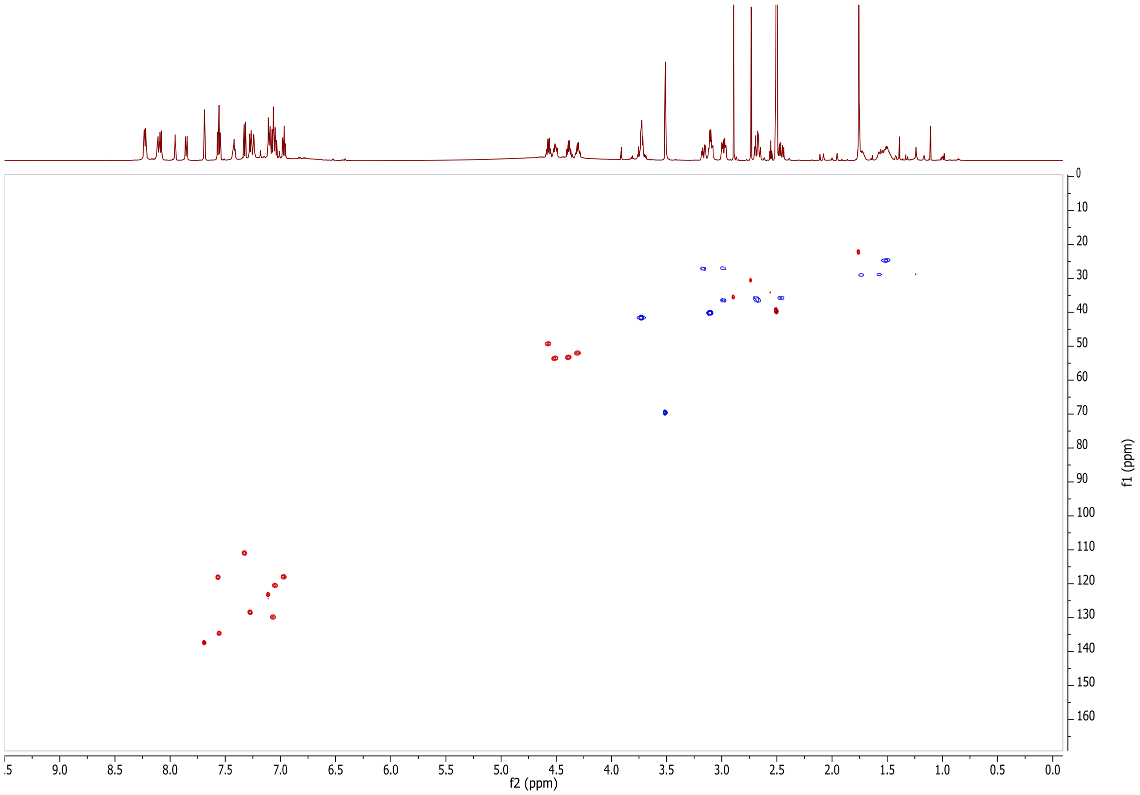


**Supplementary Figure 33 | $^{1}$H-$^{13}$C HSQC NMR spectrum of compound Ac-*m*-I-Phe-Arg-Gly-Asp-Trp-OH (1h).**

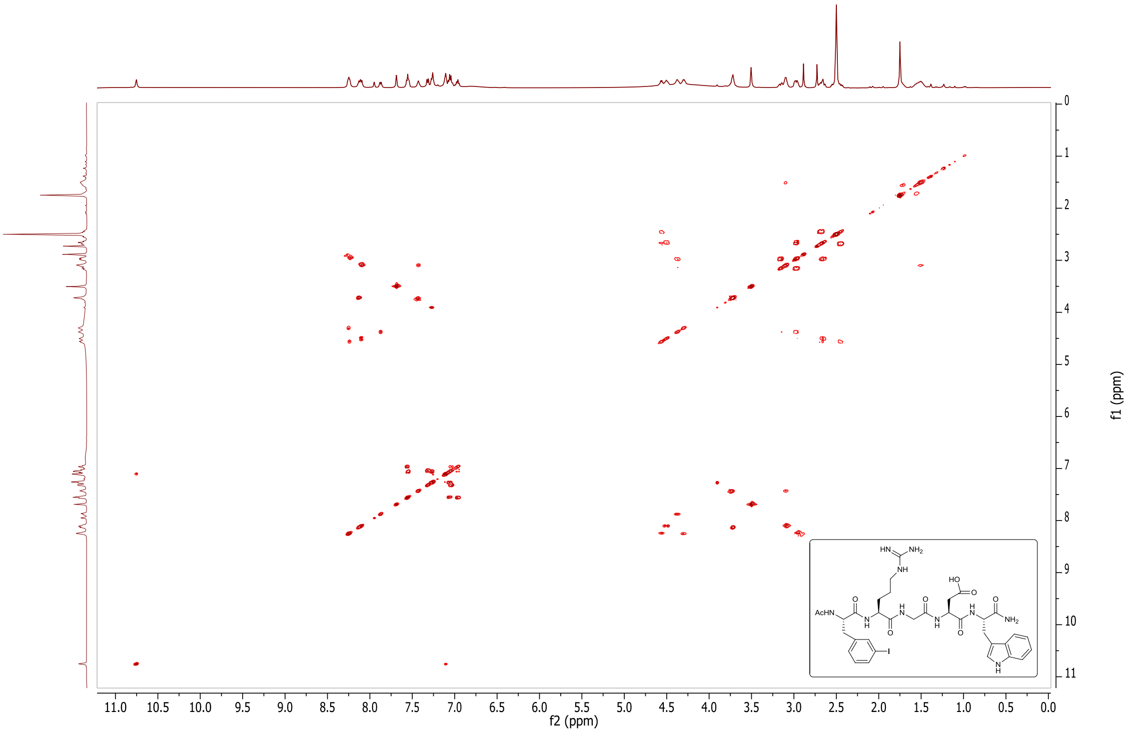

**Supplementary Figure 34 | COSY NMR spectrum of compound Ac-*m*-I-Phe-Arg-Gly-Asp-Trp-OH (1h).**

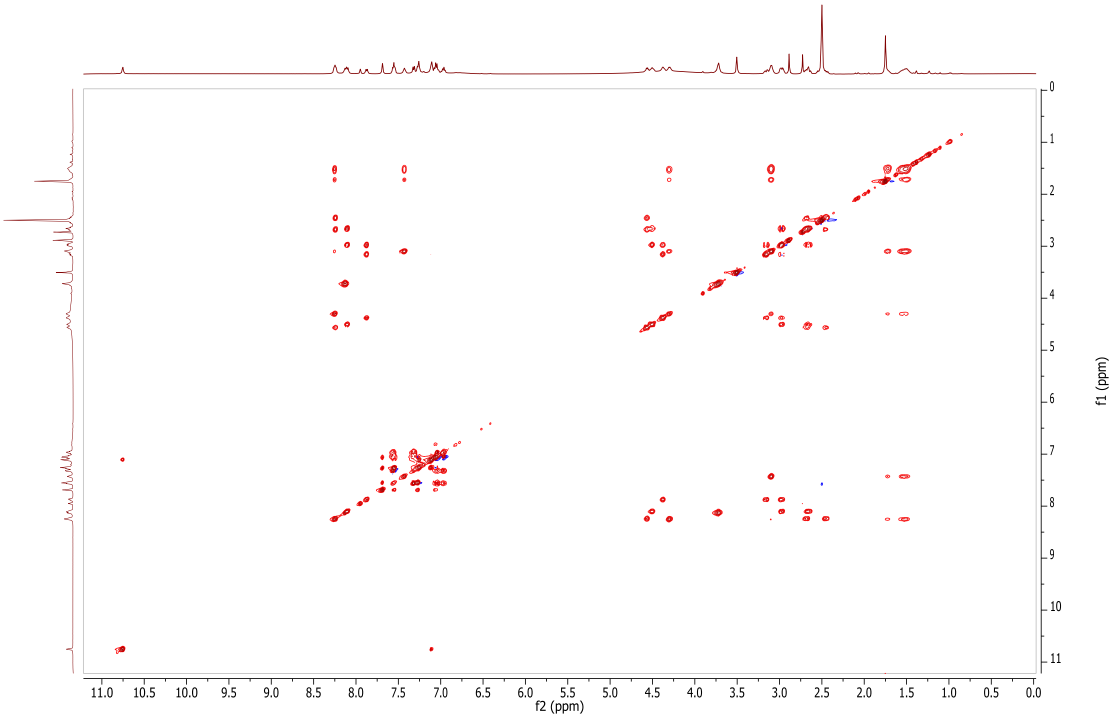

**Supplementary Figure 35 | TOCSY NMR spectrum of compound Ac-*m*-I-Phe-Arg-Gly-Asp-Trp-OH (1h).**

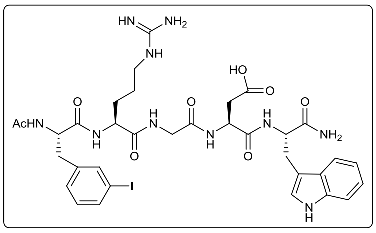


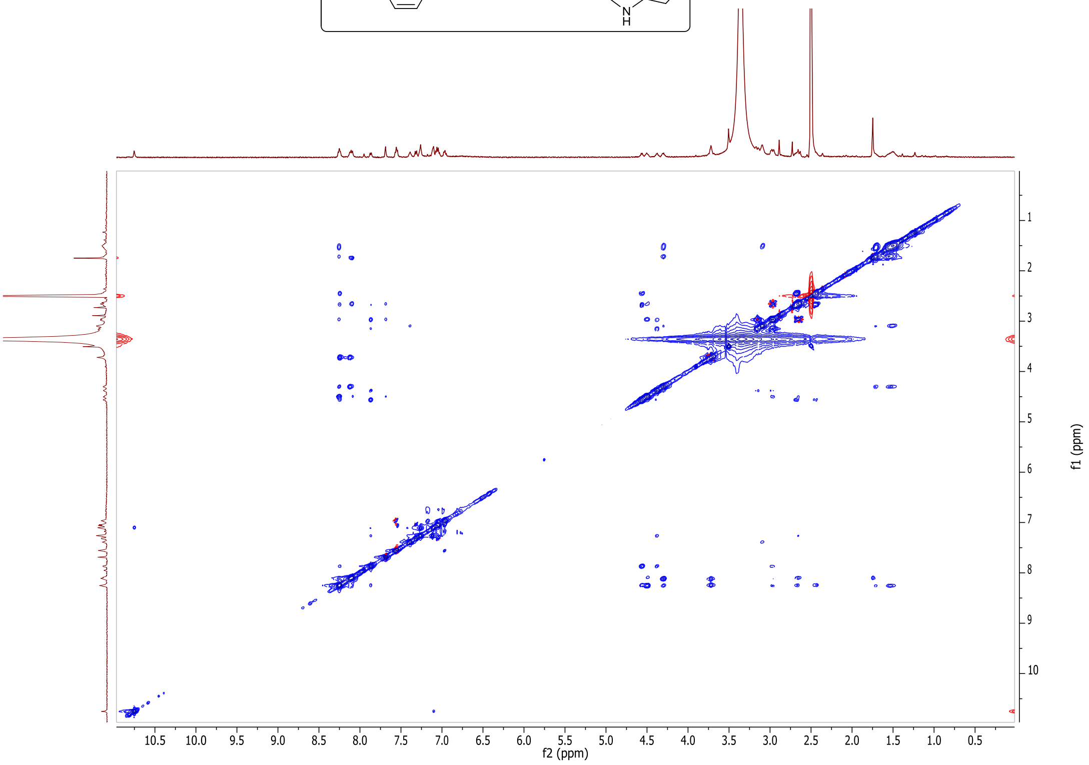


**Supplementary Figure 36 | NOESY NMR spectrum of compound Ac-*m*-I-Phe-Arg-Gly-Asp-Trp-OH (1h).**

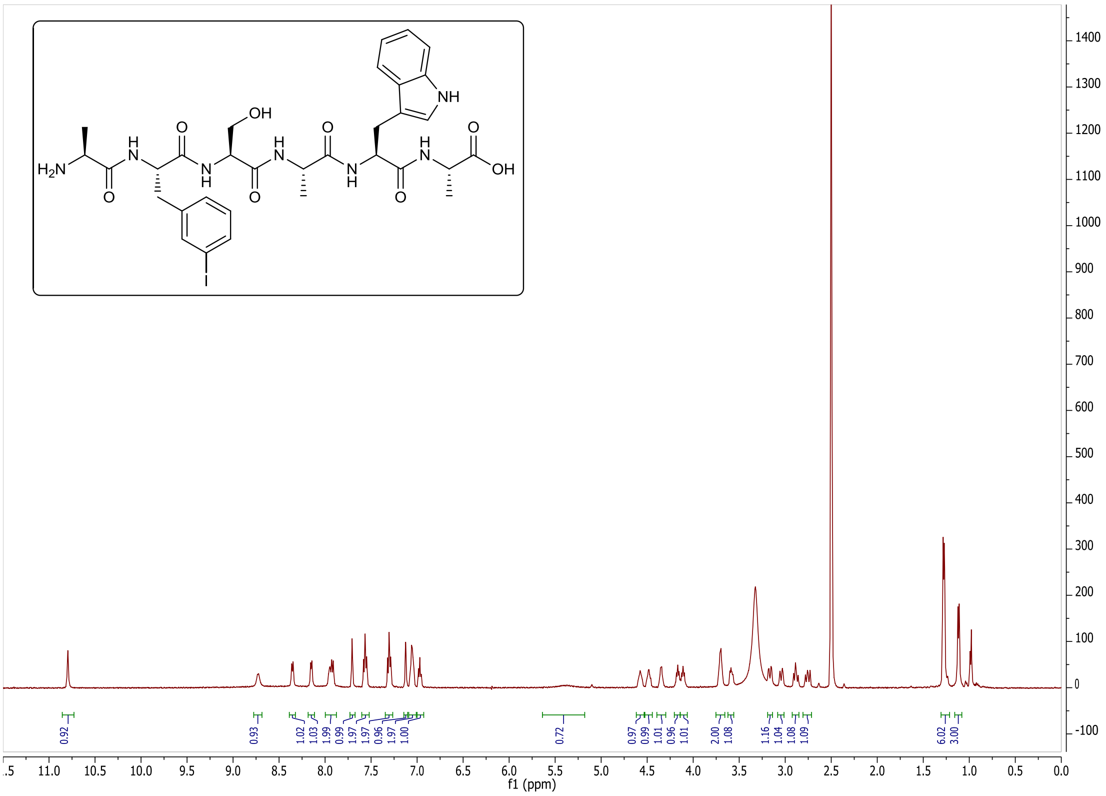

**Supplementary Figure 37 | $^{1}$H NMR spectrum of compound H-Ala-*m*-I-Phe-Ser-Ala-Trp-Ala-OH (1i).**

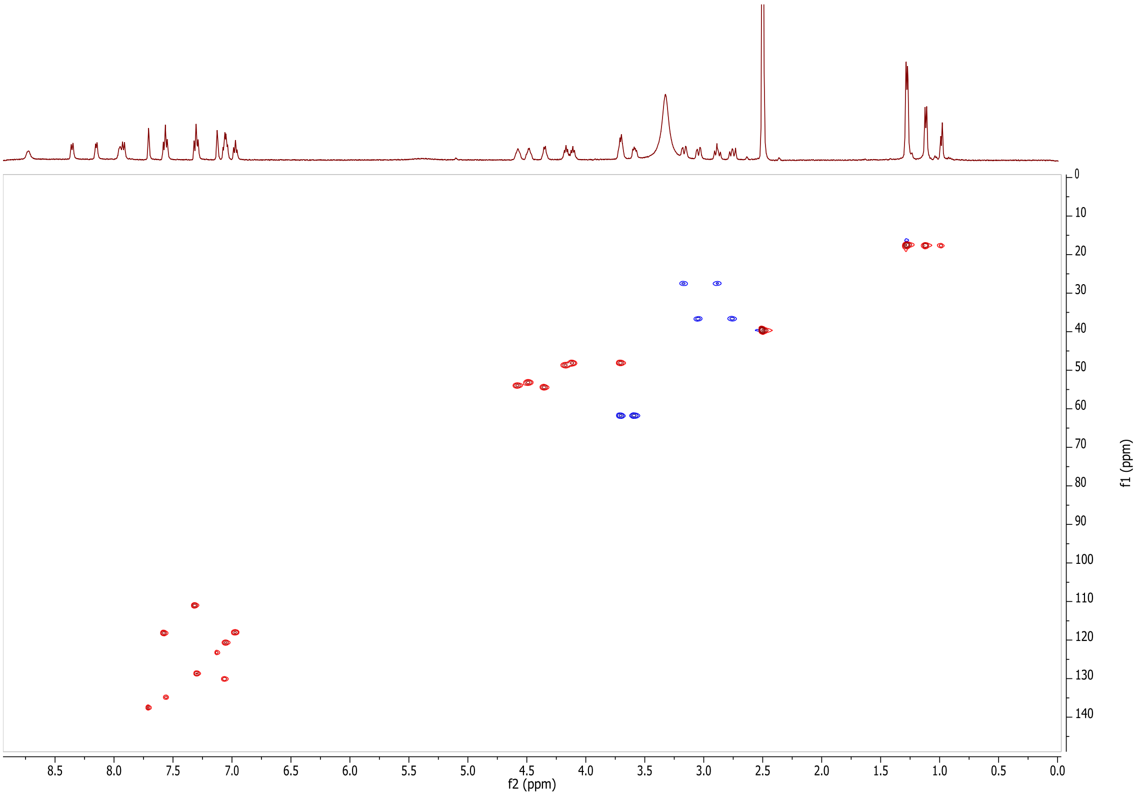

**Supplementary Figure 38 | $^{1}$H-$^{13}$C HSQC NMR spectrum of compound H-Ala-*m*-I-Phe-Ser-Ala-Trp-Ala-OH (1i).**

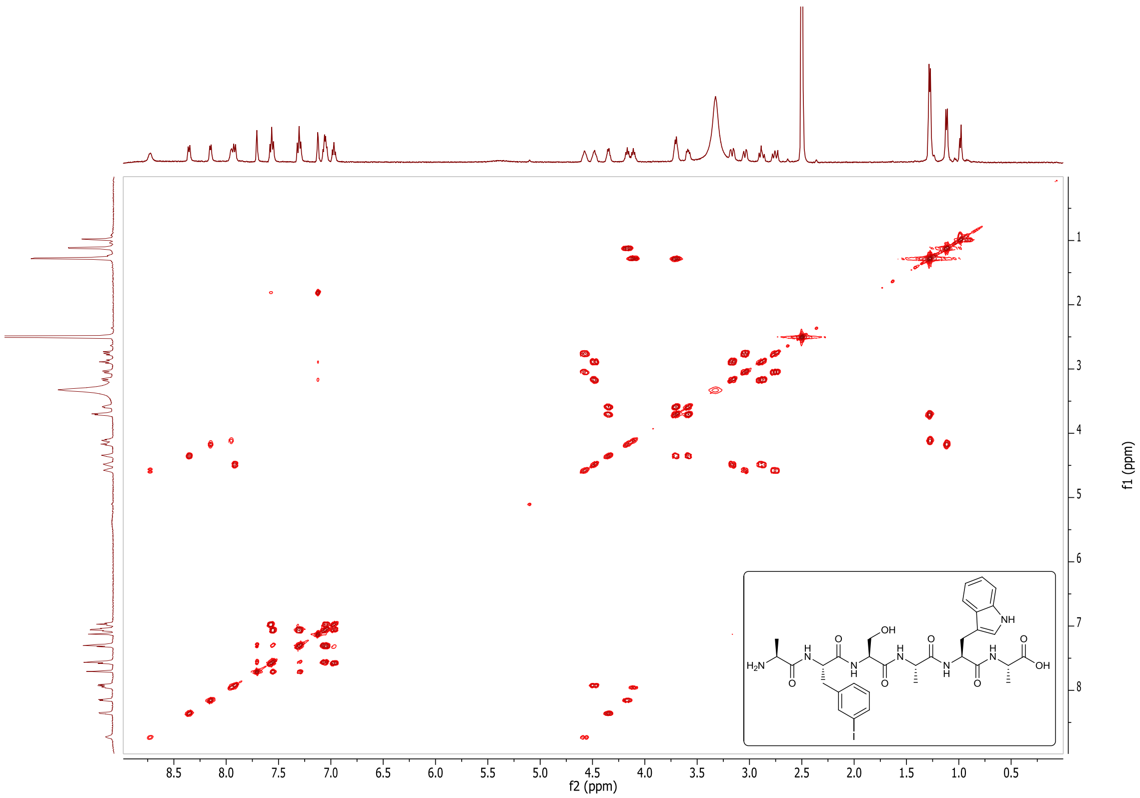


**Supplementary Figure 39 | COSY NMR spectrum of compound H-Ala-*m*-I-Phe-Ser-Ala-Trp-Ala-OH (1i).**

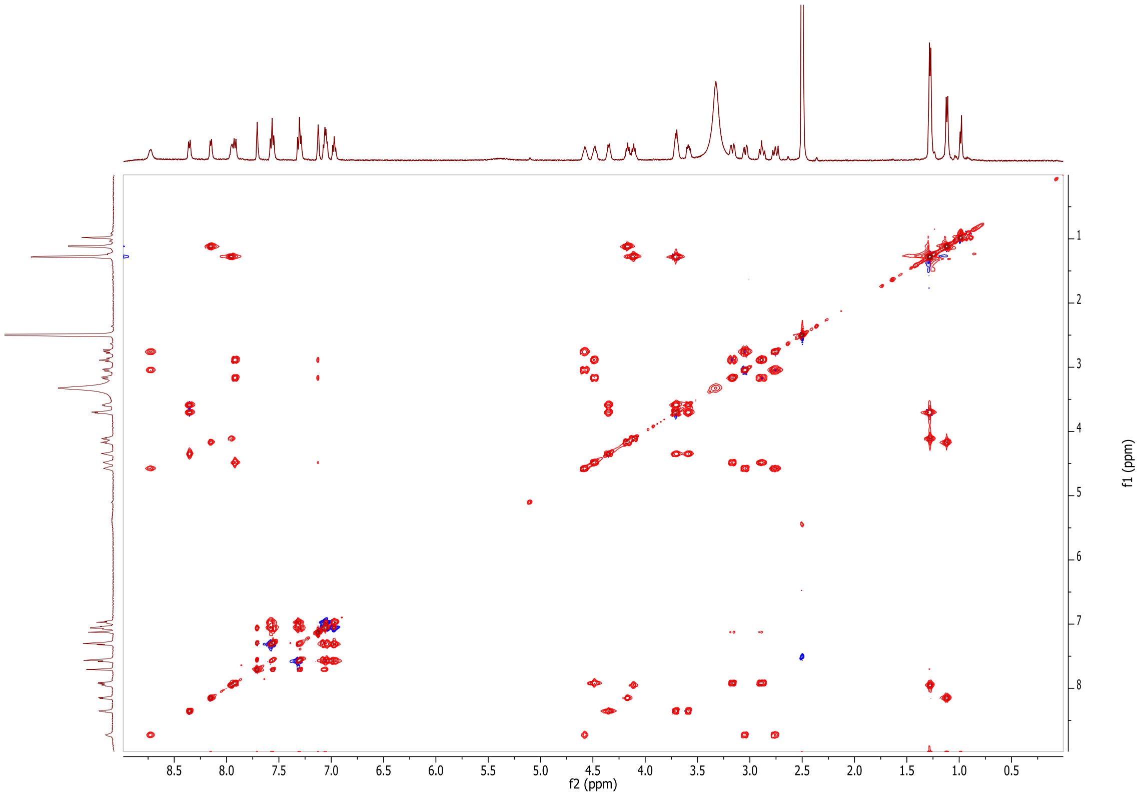


**Supplementary Figure 40 | TOCSY NMR spectrum of compound H-Ala-*m*-I-Phe-Ser-Ala-Trp-Ala-OH (1i).**

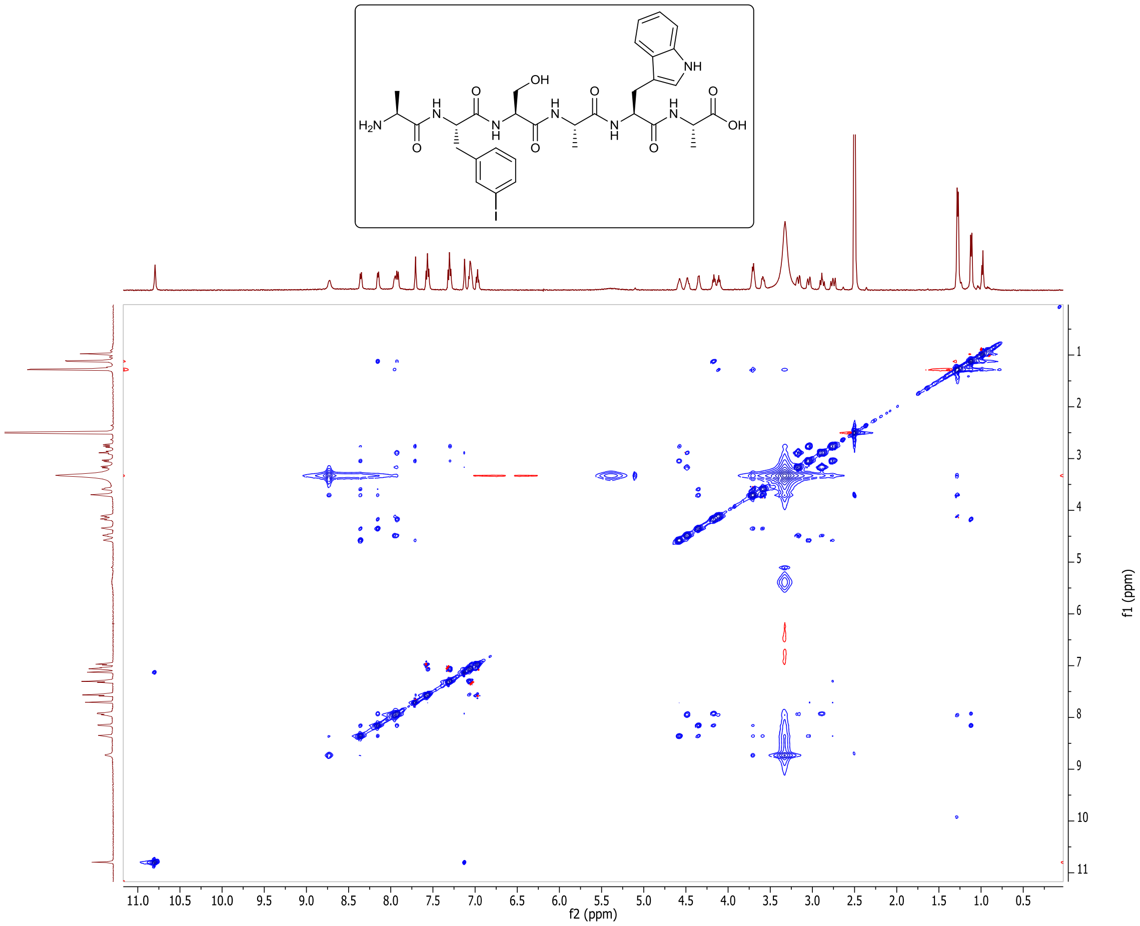

**Supplementary Figure 41 | NOESY NMR spectrum of compound H-Ala-*m*-I-Phe-Ser-Ala-Trp-Ala-OH (1i).**

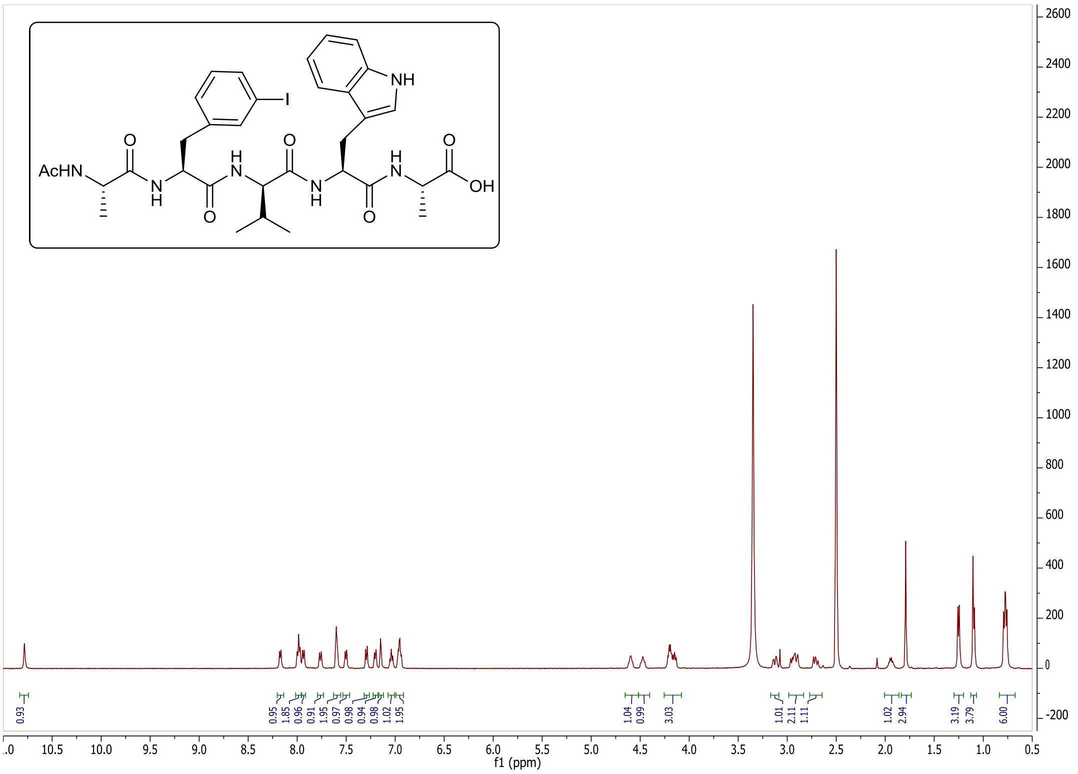


**Supplementary Figure 42 | $^{1}$H NMR spectrum of compound Ac-Ala-*m*-I-Phe-Val-Trp-Ala-OH (1j).**

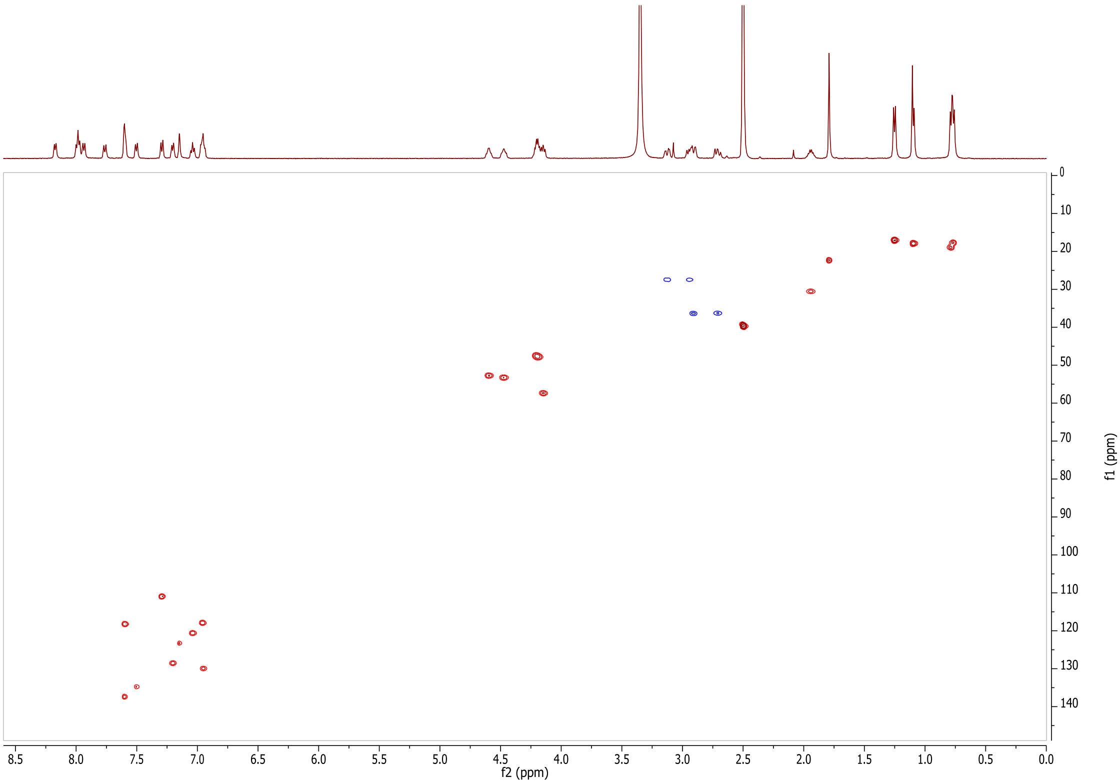


**Supplementary Figure 43 | $^{1}$H-$^{13}$C HSQC NMR spectrum of compound Ac-Ala-*m*-I-Phe-Val-Trp-Ala-OH (1j).**

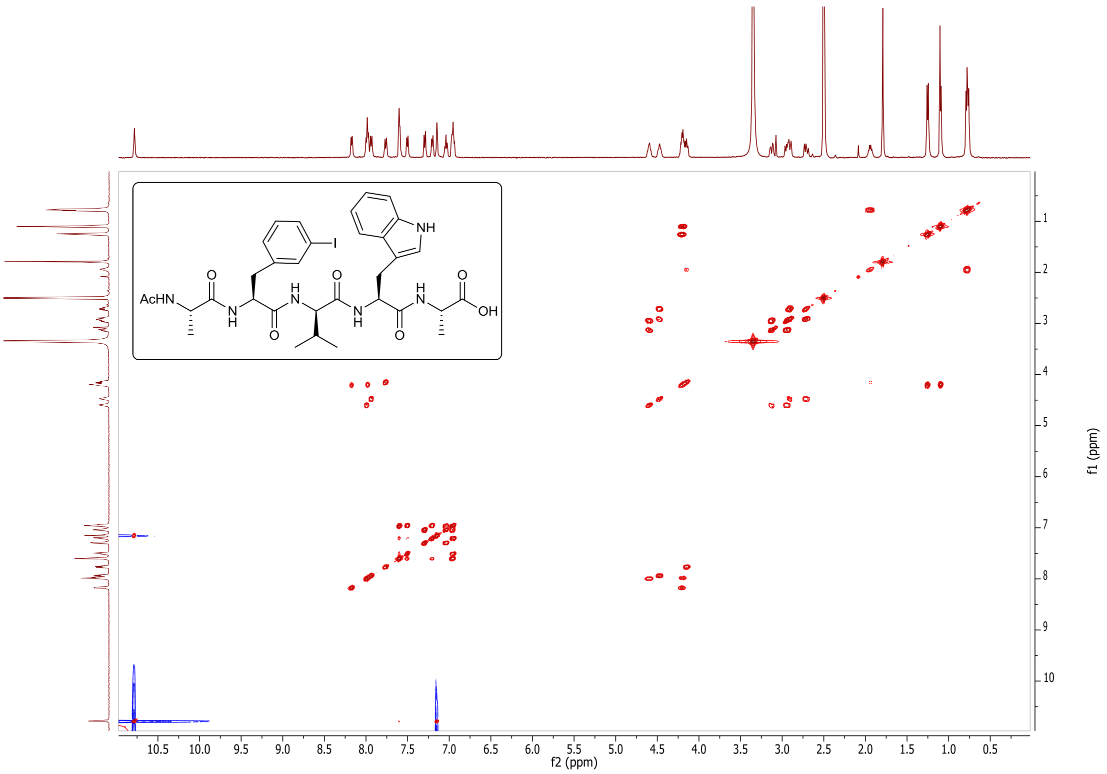


**Supplementary Figure 44 | COSY NMR spectrum of compound Ac-Ala-*m*-I-Phe-Val-Trp-Ala-OH (1j).**

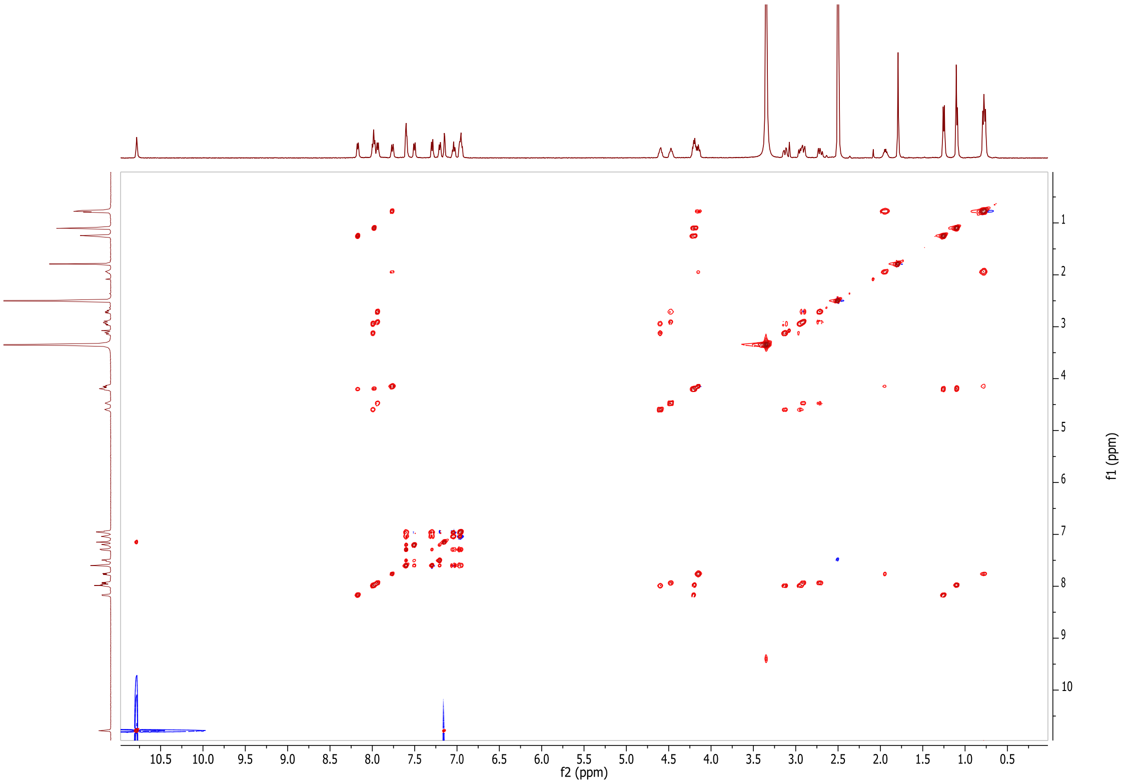


**Supplementary Figure 45 | TOCSY NMR spectrum of compound Ac-Ala-*m*-I-Phe-Val-Trp-Ala-OH (1j).**

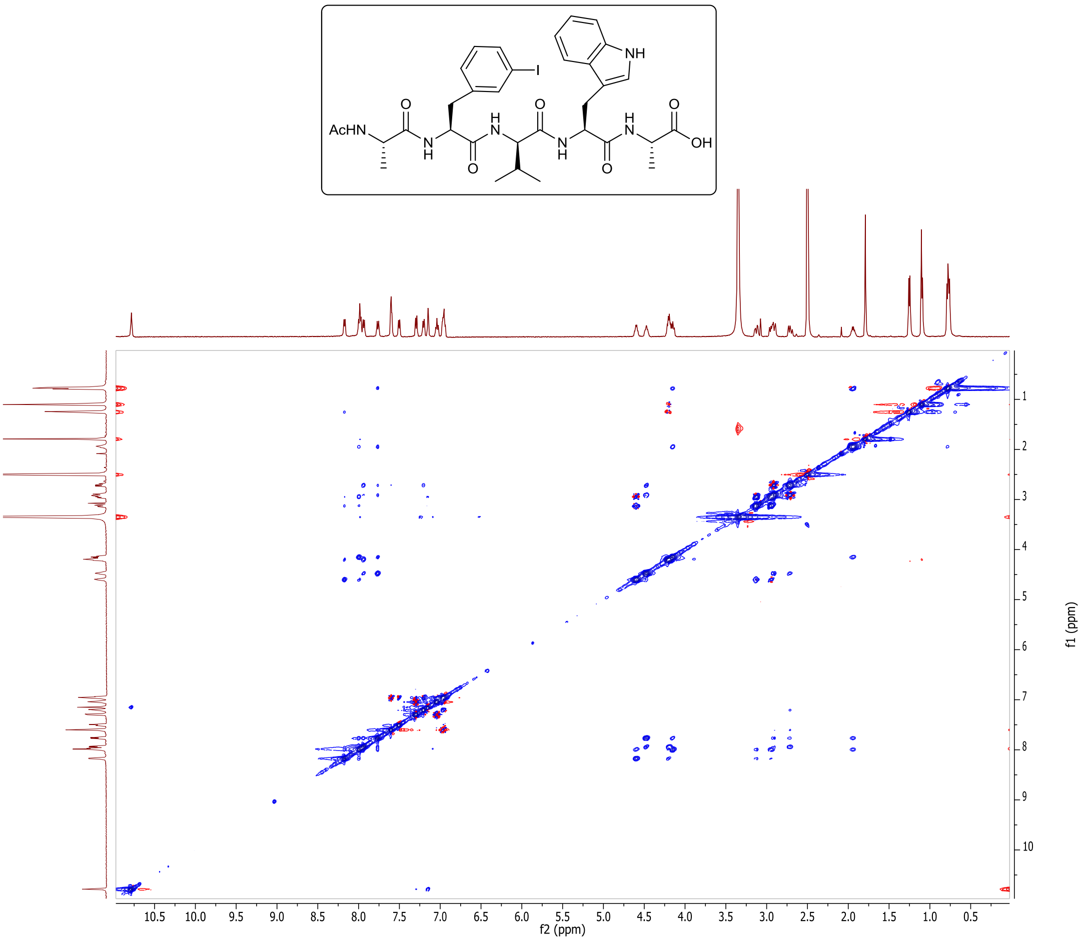


**Supplementary Figure 46 | NOESY NMR spectrum of compound Ac-Ala-*m*-I-Phe-Val-Trp-Ala-OH (1j).**

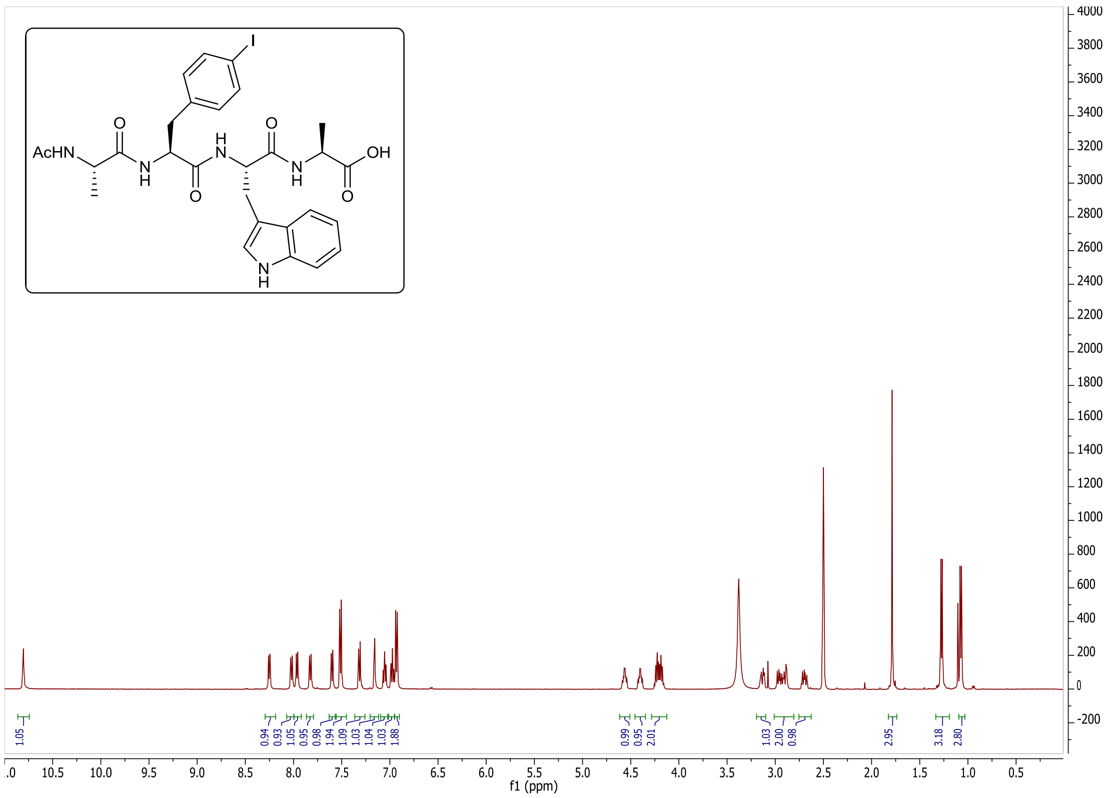

**Supplementary Figure 47 | $^1$H NMR spectrum of compound Ac-Ala-*p*-I-Phe-Trp-Ala-OH (1k).**

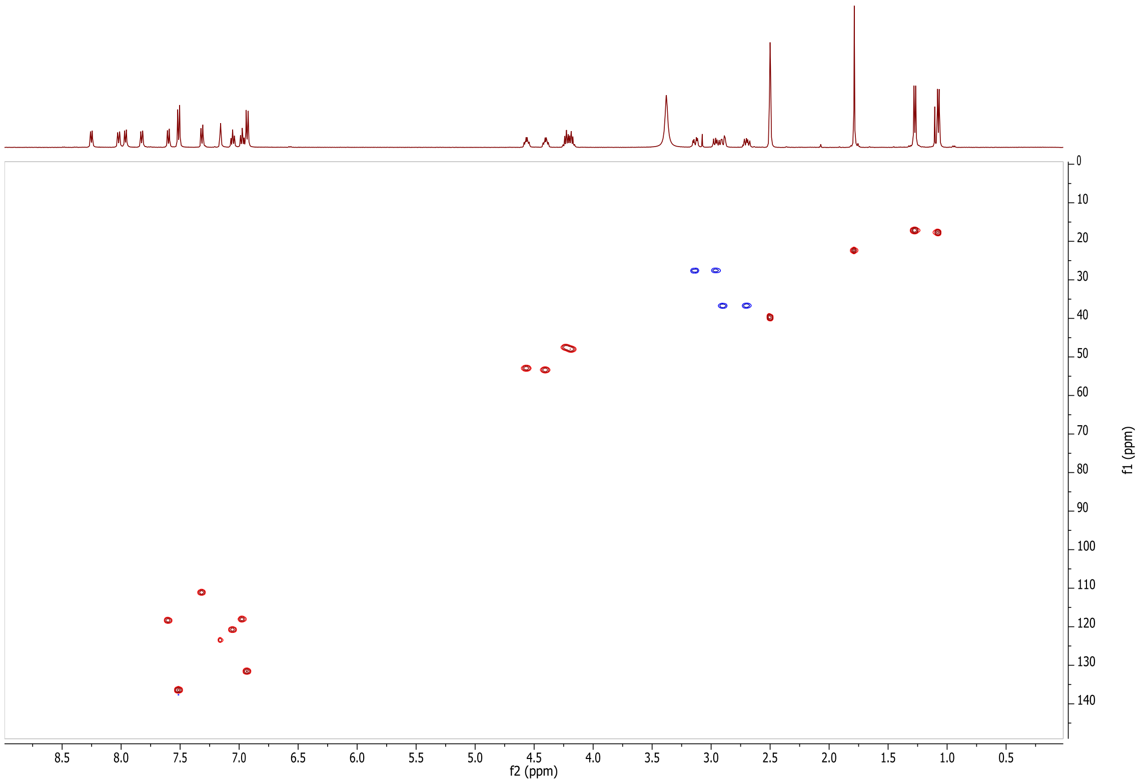

**Supplementary Figure 48 | $^1$H-$^{13}$C HSQC NMR spectrum of compound Ac-Ala-*p*-I-Phe-Trp-Ala-OH (1k).**

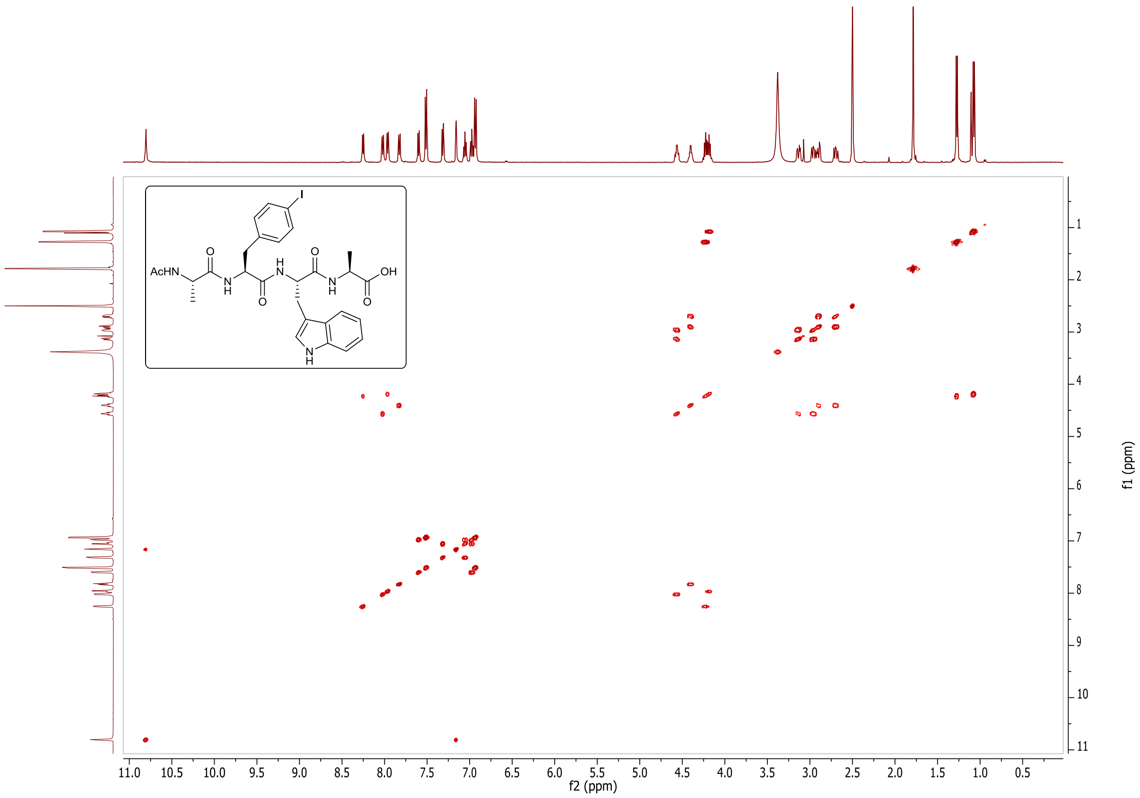


**Supplementary Figure 49 | COSY NMR spectrum of compound Ac-Ala-*p*-I-Phe-Trp-Ala-OH (1k).**

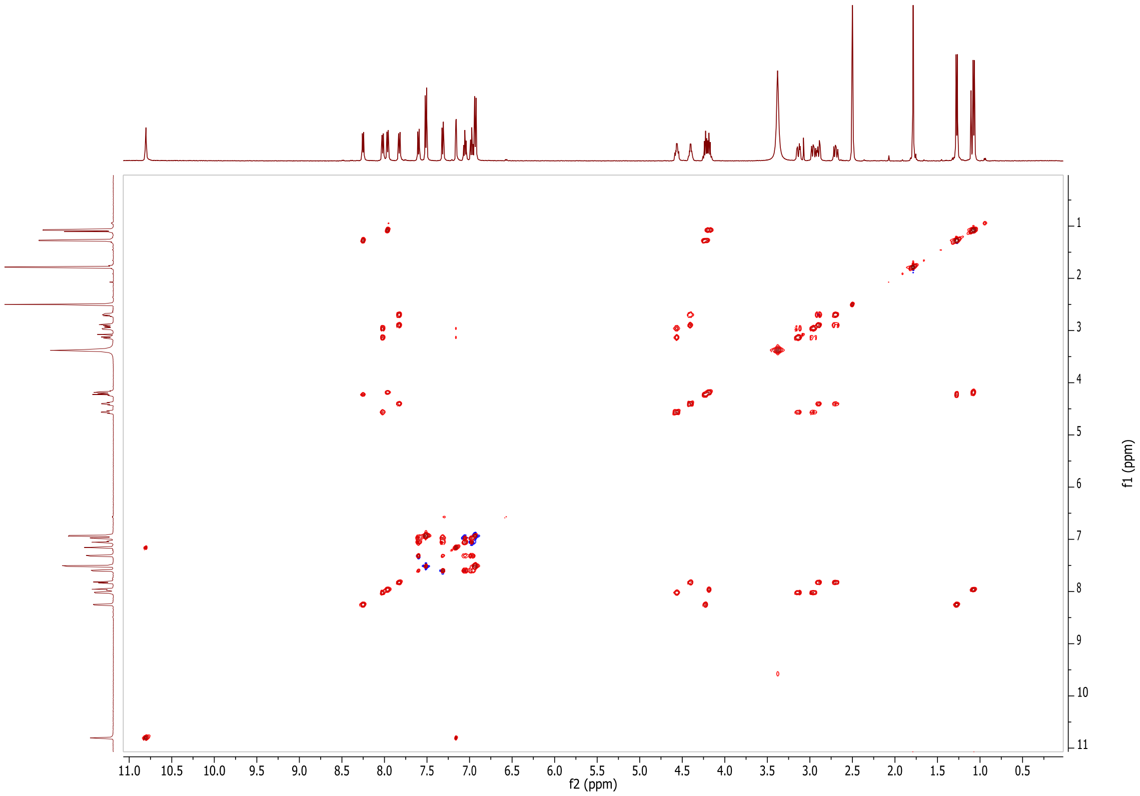


**Supplementary Figure 50 | TOCSY NMR spectrum of compound Ac-Ala-*p*-I-Phe-Trp-Ala-OH (1k).**

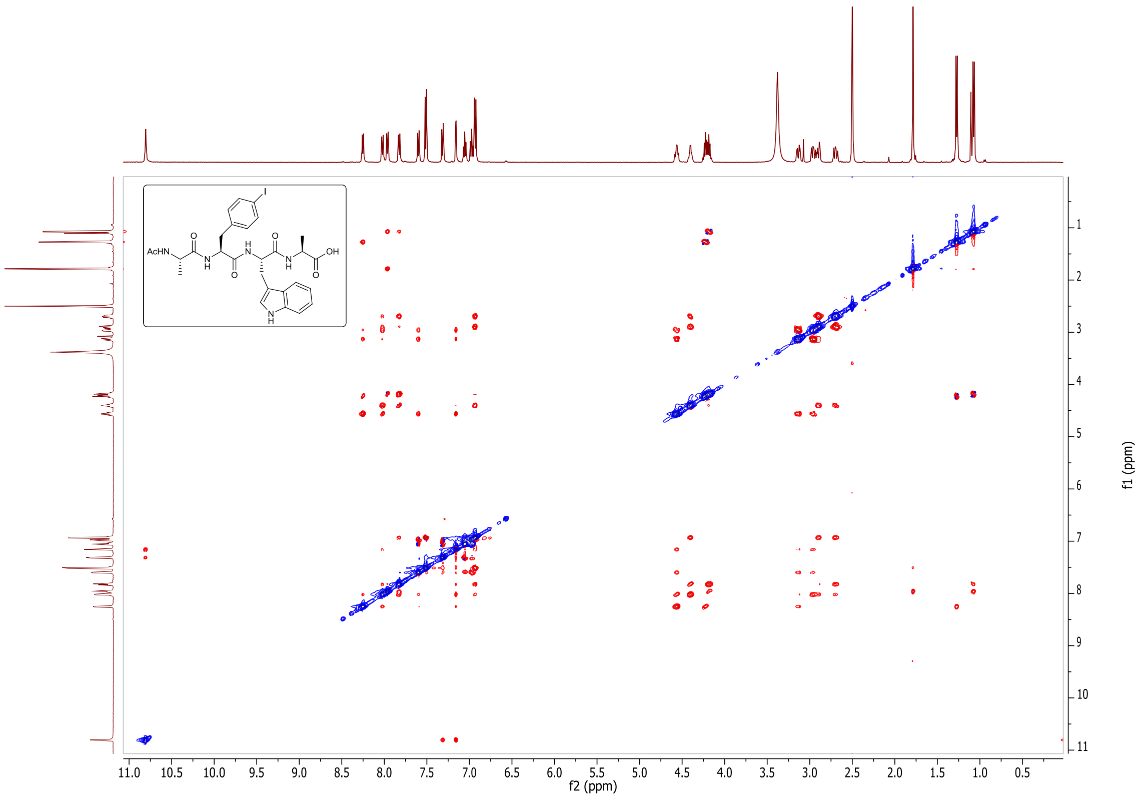

**Supplementary Figure 51 | ROESY NMR spectrum of compound Ac-Ala-*p*-I-Phe-Trp-Ala-OH (1k).**

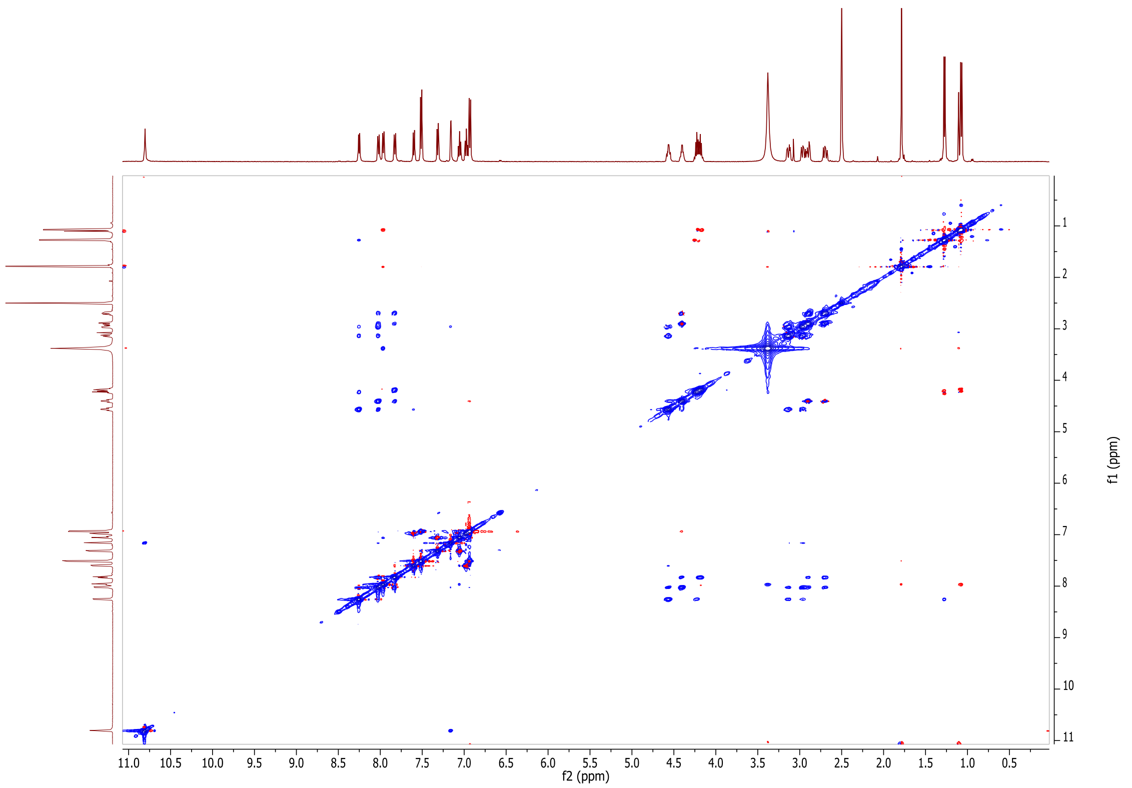

**Supplementary Figure 52 | NOESY NMR spectrum of compound Ac-Ala-*p*-I-Phe-Trp-Ala-OH (1k).**

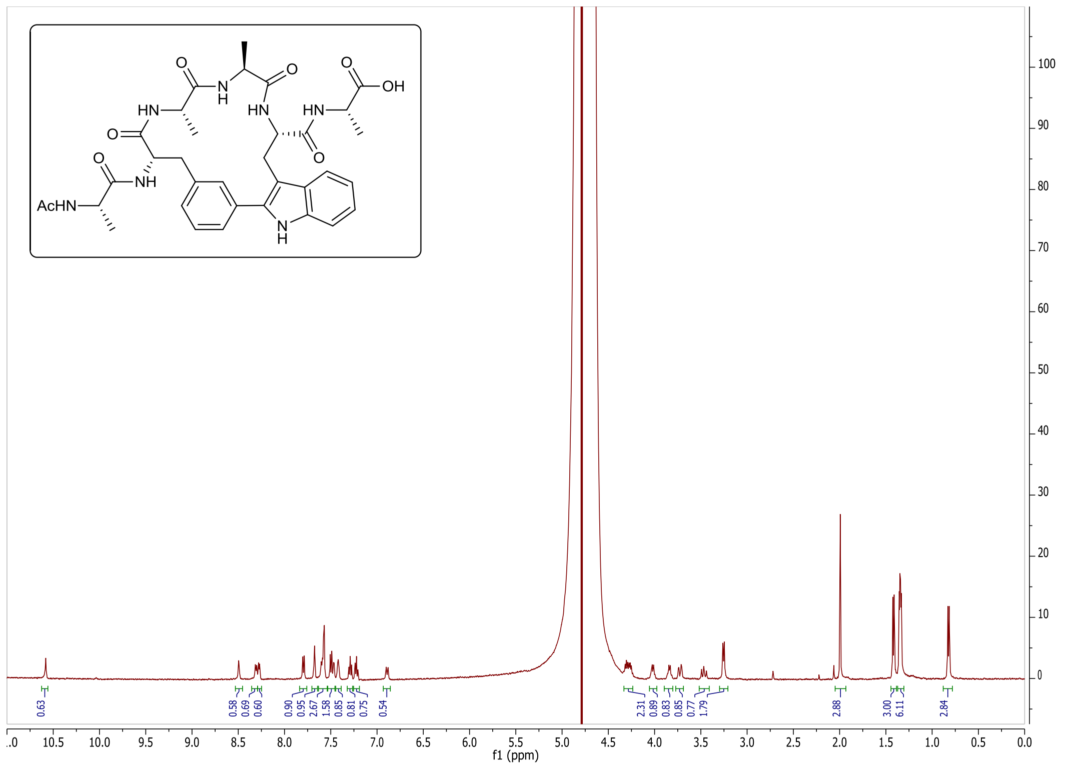

**Supplementary Figure 53 | ¹H NMR spectrum of compound Ac-Ala-(Cyclo-*m*)-[Phe-Ala-Ala-Trp]-Ala-OH (2b).**

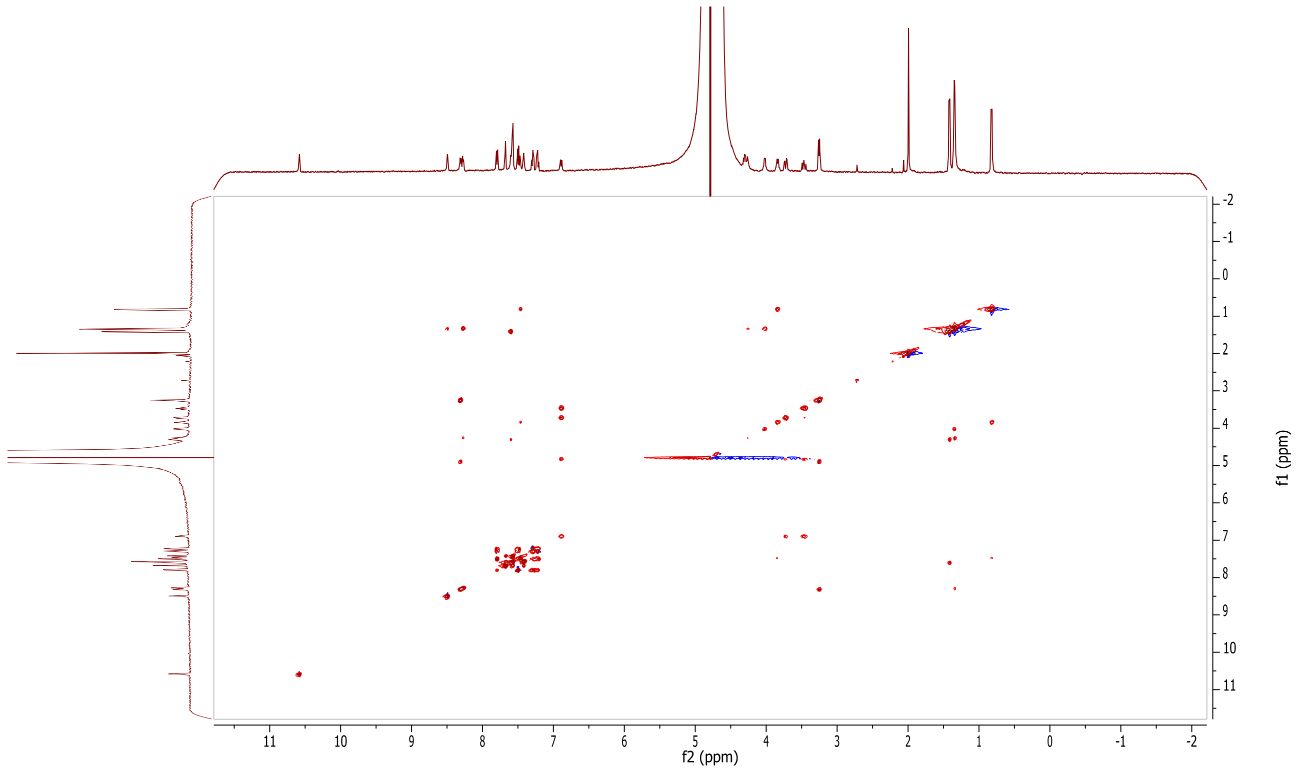

**Supplementary Figure 54 | TOCSY NMR spectrum of compound Ac-Ala-(Cyclo-*m*)-[Phe-Ala-Ala-Trp]-Ala-OH (2b).**

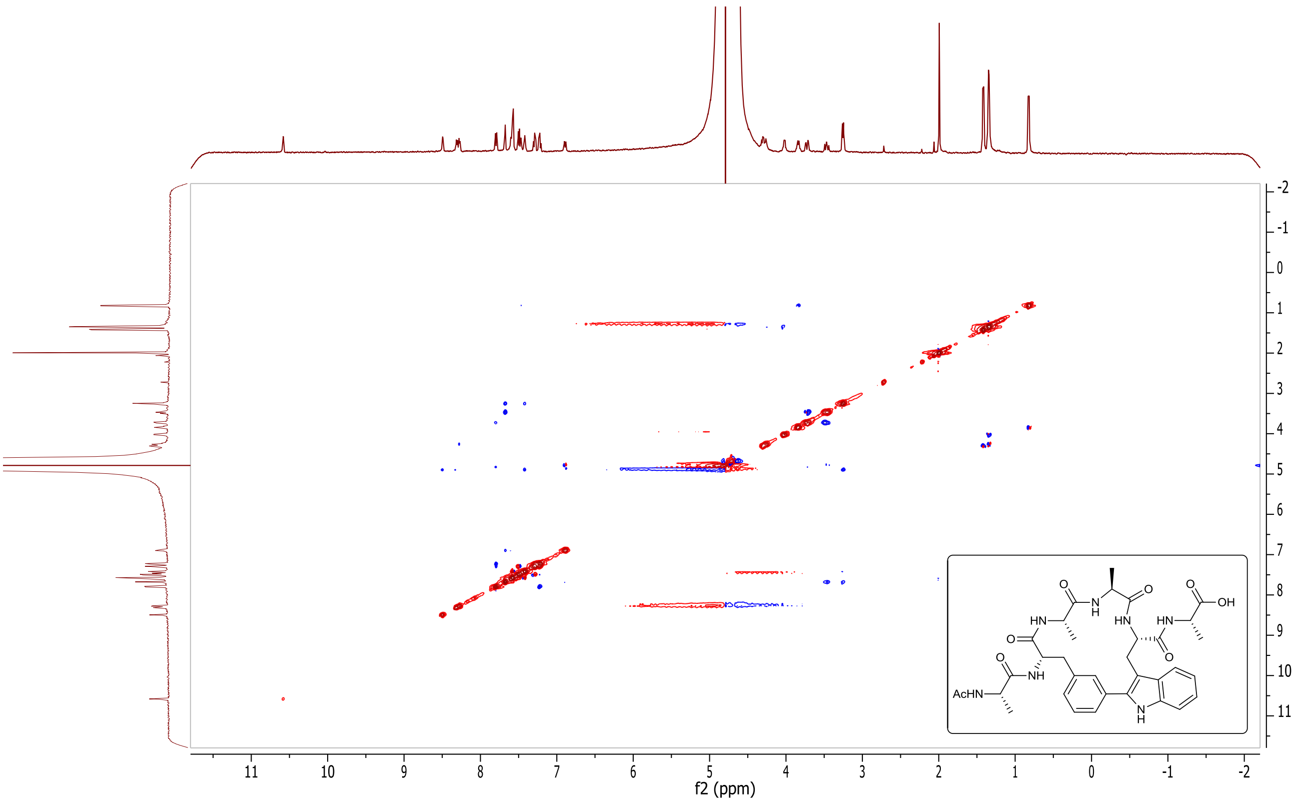


**Supplementary Figure 55 | ROESY NMR spectrum of compound Ac-Ala-(Cyclo-*m*)-[Phe-Ala-Ala-Trp]-Ala-OH (2b).**

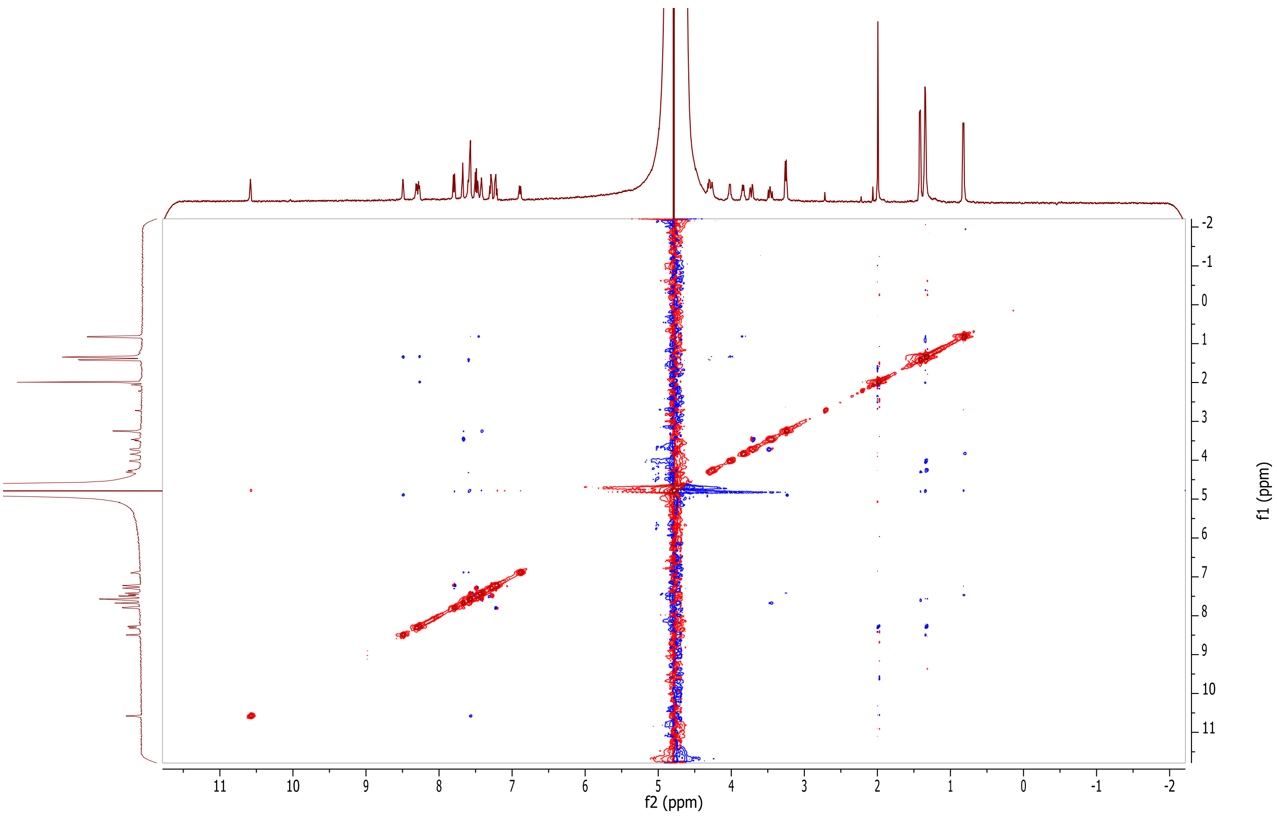


**Supplementary Figure 56 | NOESY NMR spectrum of compound Ac-Ala-(Cyclo-*m*)-[Phe-Ala-Ala-Trp]-Ala-OH (2b).**

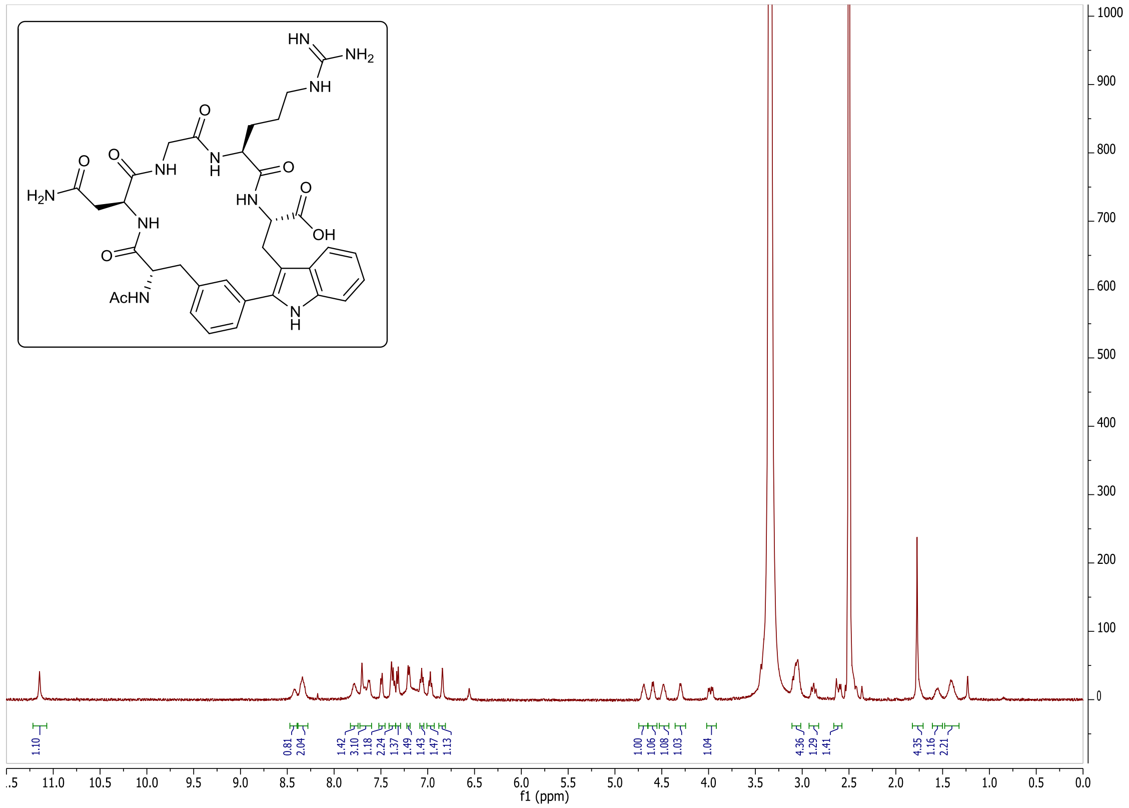

**Supplementary Figure 57 | $^{1}$H NMR spectrum of compound Ac-(Cyclo-*m*)-[Phe-Asn-Gly-Arg-Trp]-OH (2g).**

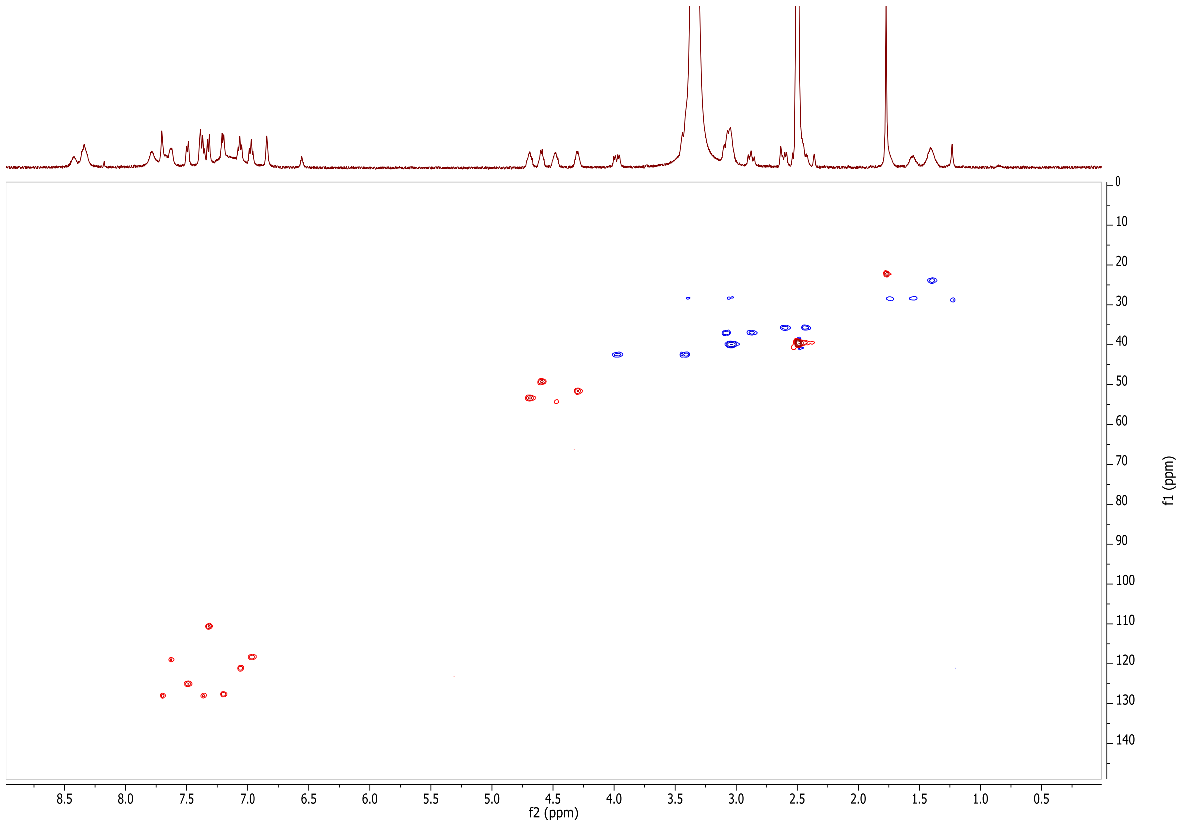

**Supplementary Figure 58 | $^{1}$H-$^{13}$C HSQC NMR spectrum of compound Ac-(Cyclo-*m*)-[Phe-Asn-Gly-Arg-Trp]-OH (2g).**

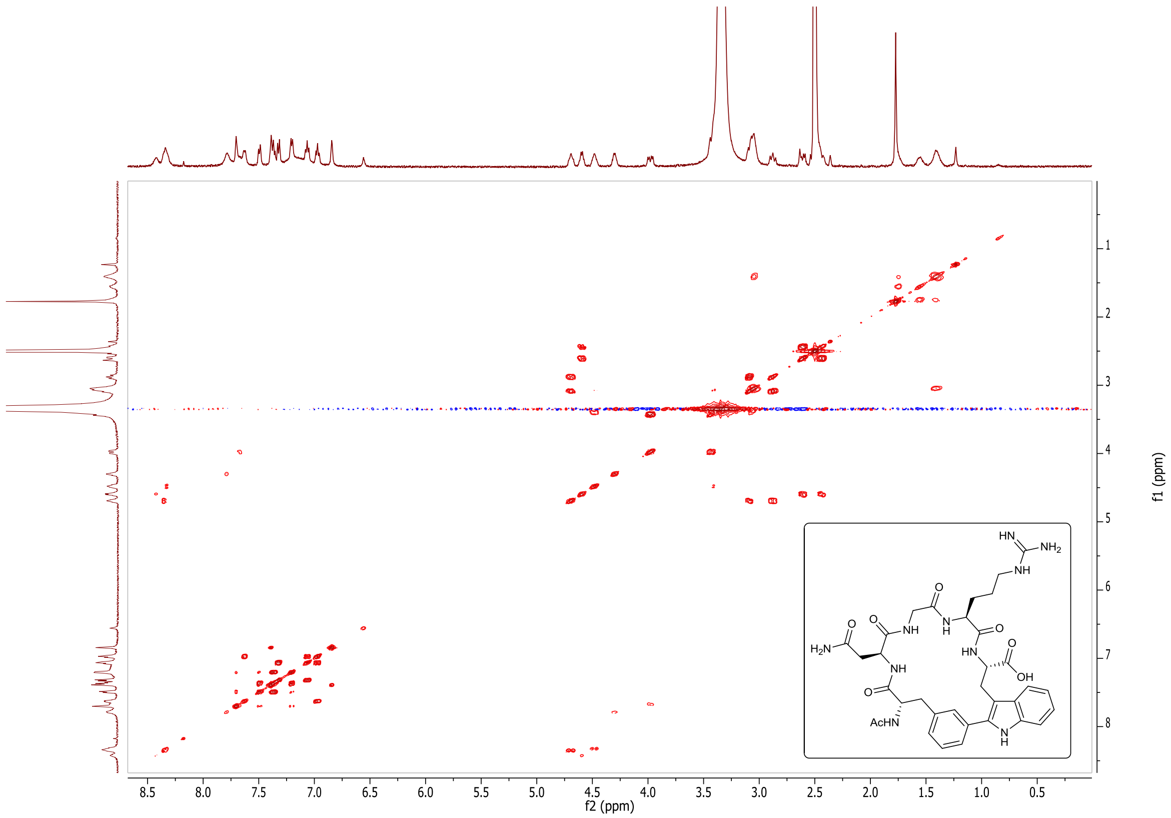


**Supplementary Figure 59 | COSY NMR spectrum of compound Ac-(Cyclo-*m*)-[Phe-Asn-Gly-Arg-Trp]-OH (2g).**

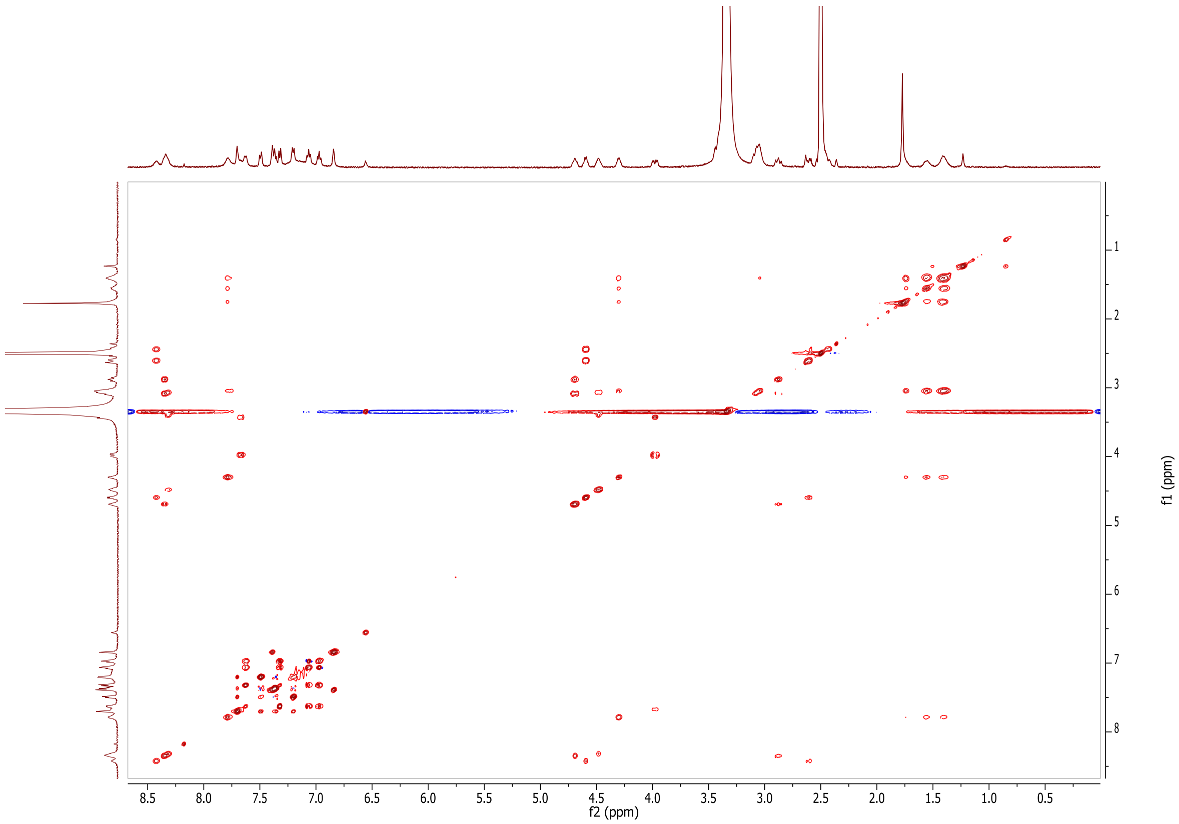


**Supplementary Figure 60 | TOCSY NMR spectrum of compound Ac-(Cyclo-*m*)-[Phe-Asn-Gly-Arg-Trp]-OH (2g).**

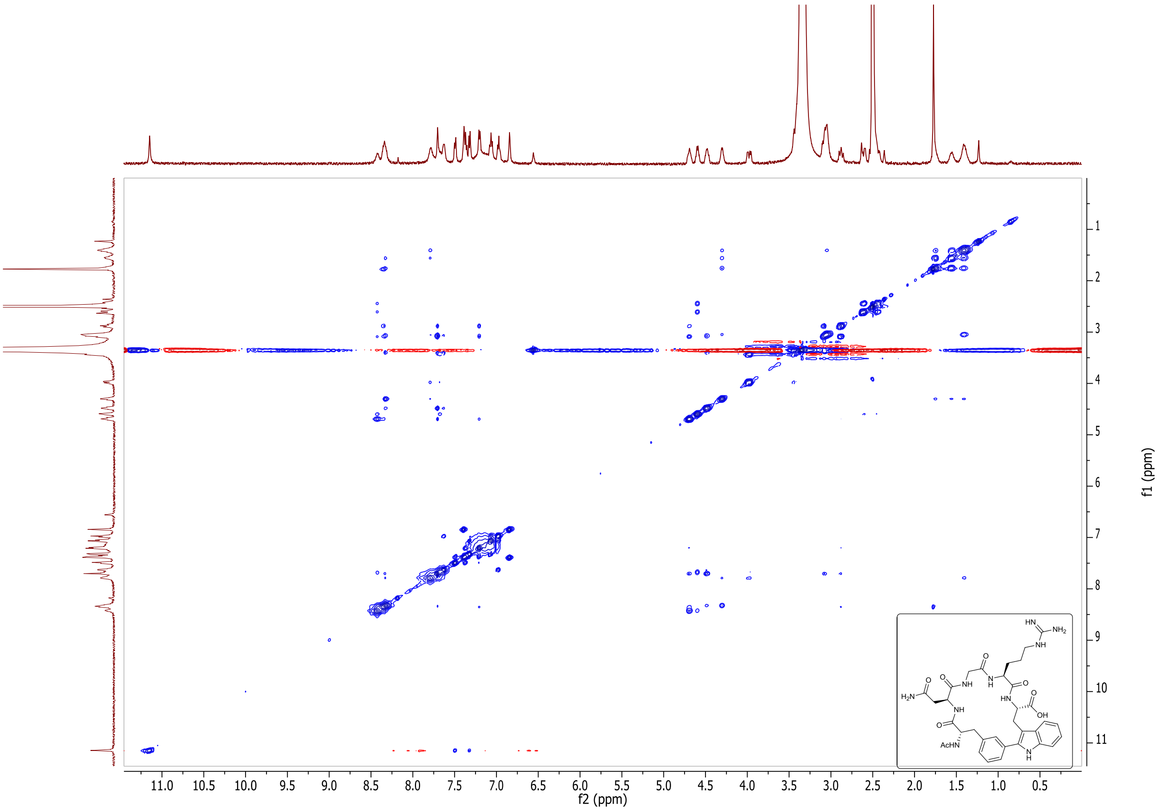


**Supplementary Figure 61 | NOESY NMR spectrum of compound Ac-(Cyclo-*m*)-[Phe-Asn-Gly-Arg-Trp]-OH (2g).**

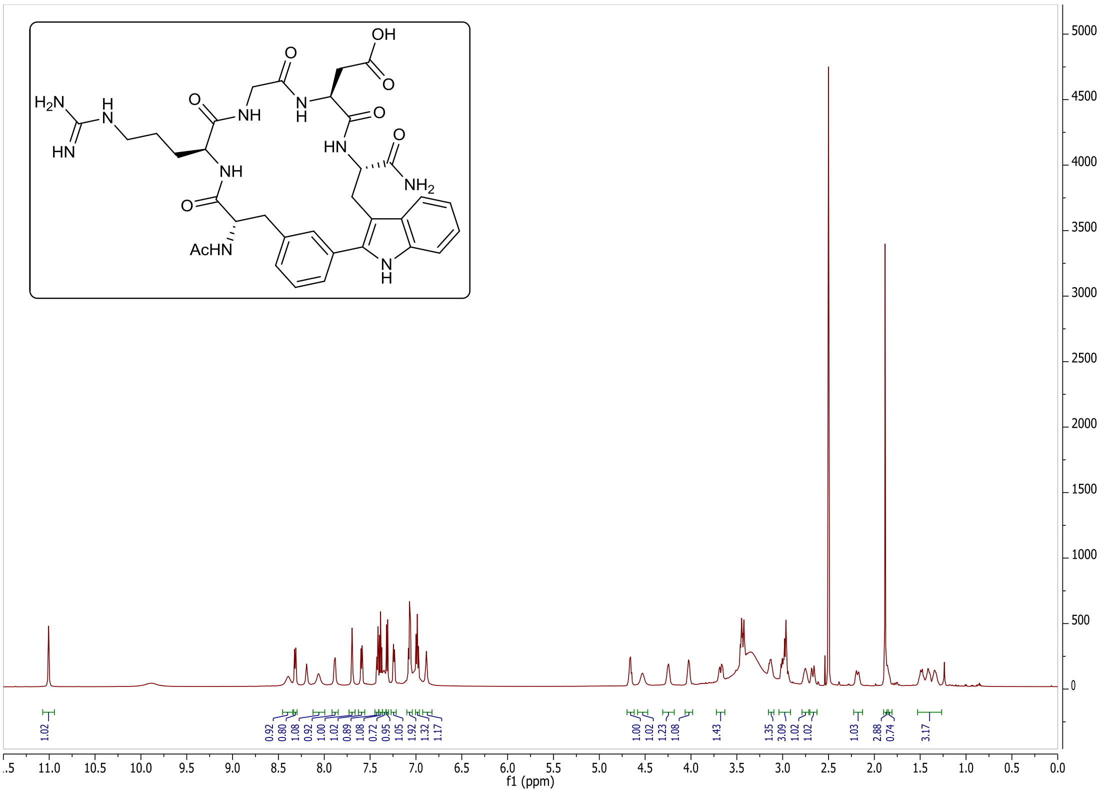


**Supplementary Figure 62 | $^{1}$H NMR spectrum of compound Ac-(Cyclo-*m*)-[Phe-Arg-Gly-Asp-Trp]-NH$_2$ (2h).**

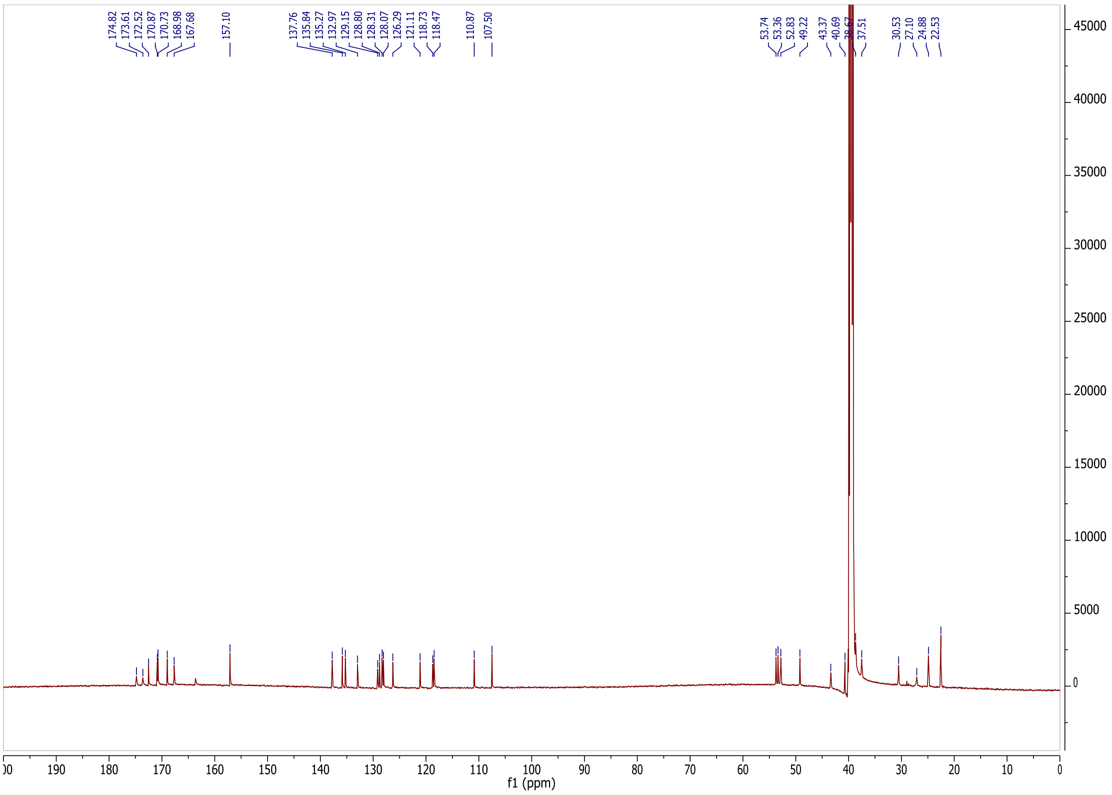


**Supplementary Figure 63 | $^{13}$C NMR spectrum of compound Ac-(Cyclo-*m*)-[Phe-Arg-Gly-Asp-Trp]-NH$_2$ (2h).**

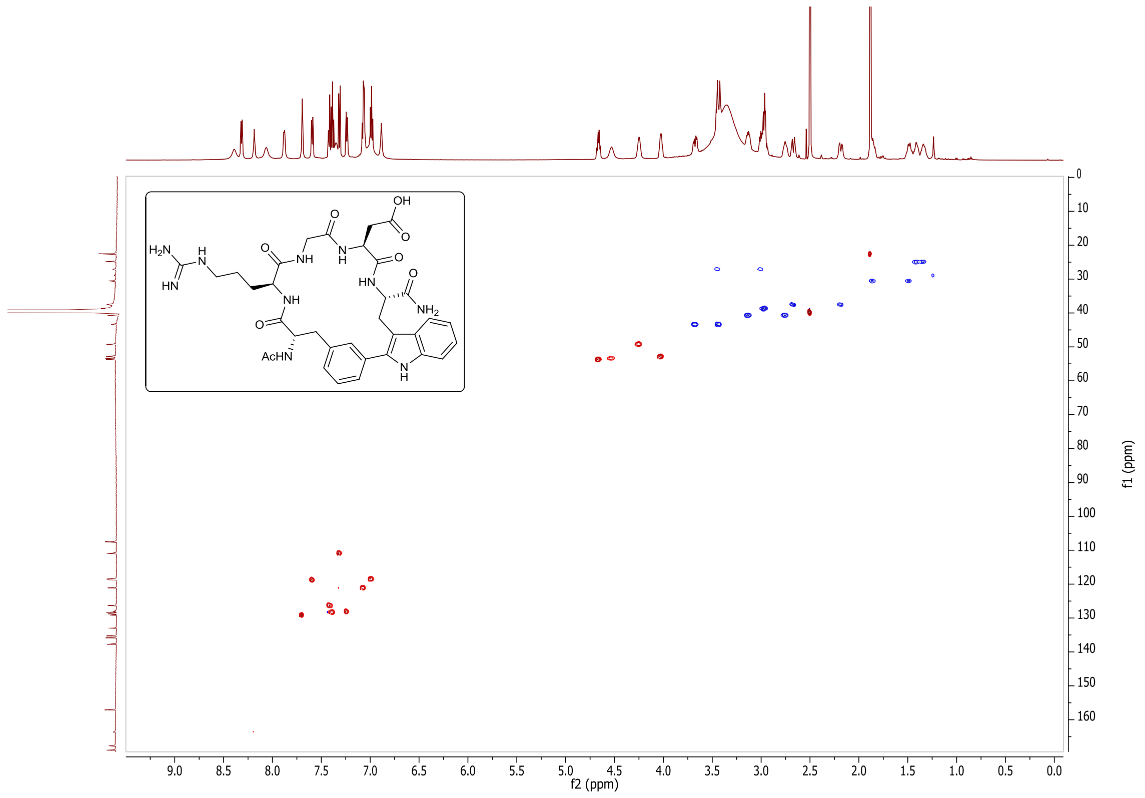

**Supplementary Figure 64 | $^{1}H$-$^{13}C$ HSQC NMR spectrum of compound Ac-(Cyclo-*m*)-[Phe-Arg-Gly-Asp-Trp]-$NH_2$ (2h).**

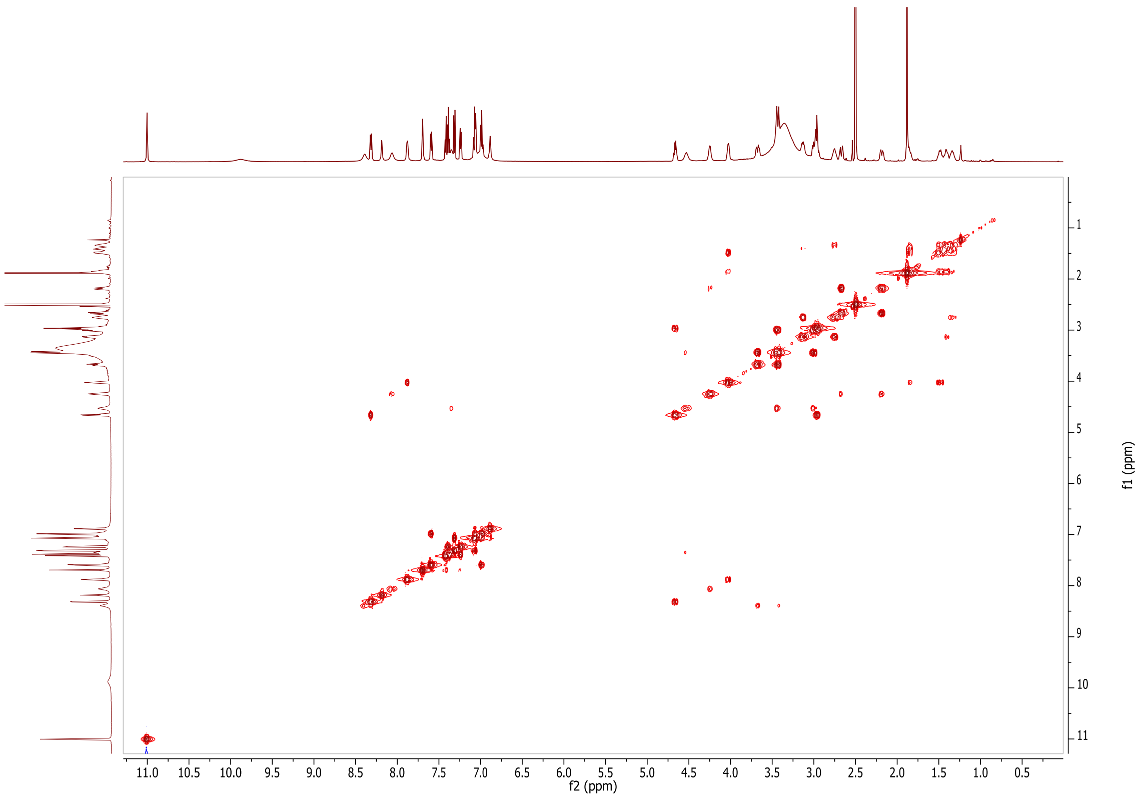

**Supplementary Figure 65 | COSY NMR spectrum of compound Ac-(Cyclo-*m*)-[Phe-Arg-Gly-Asp-Trp]-$NH_2$ (2h).**

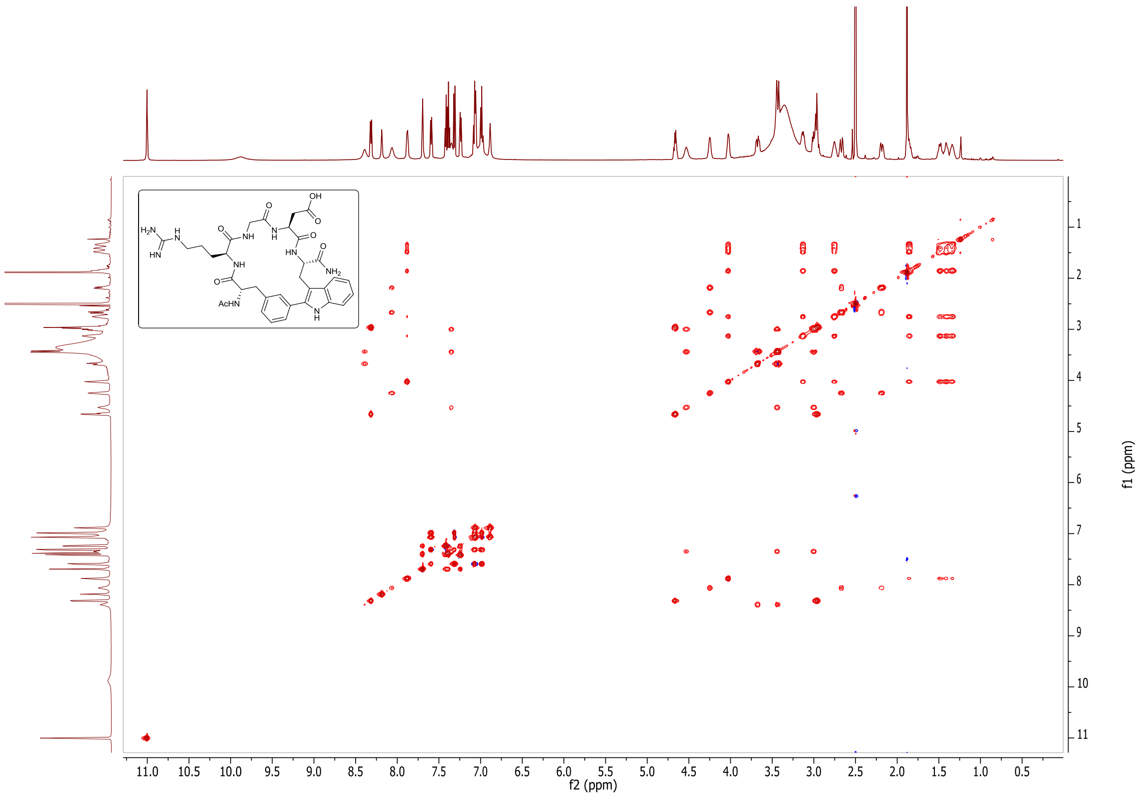

**Supplementary Figure 66 | TOCSY NMR spectrum of compound Ac-(Cyclo-*m*)-[Phe-Arg-Gly-Asp-Trp]-$NH_2$ (2h).**

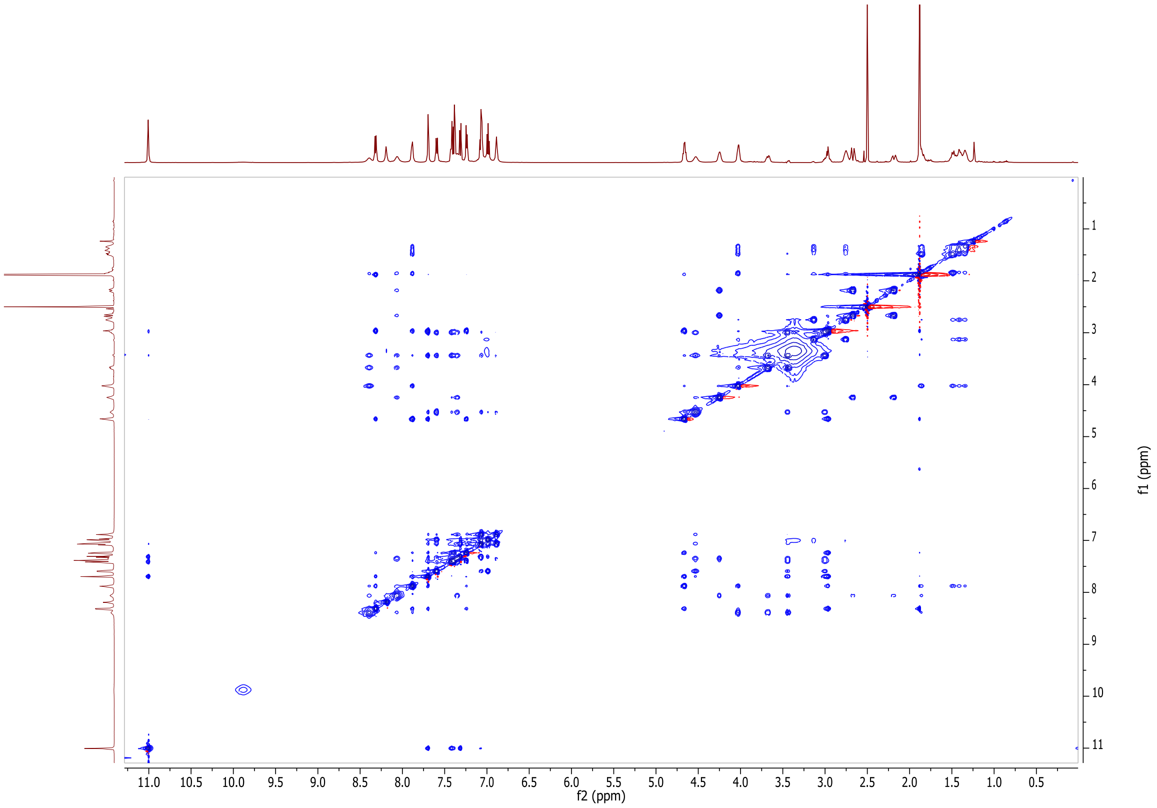

**Supplementary Figure 67 | NOESY NMR spectrum of compound Ac-(Cyclo-*m*)-[Phe-Arg-Gly-Asp-Trp]-$NH_2$ (2h).**

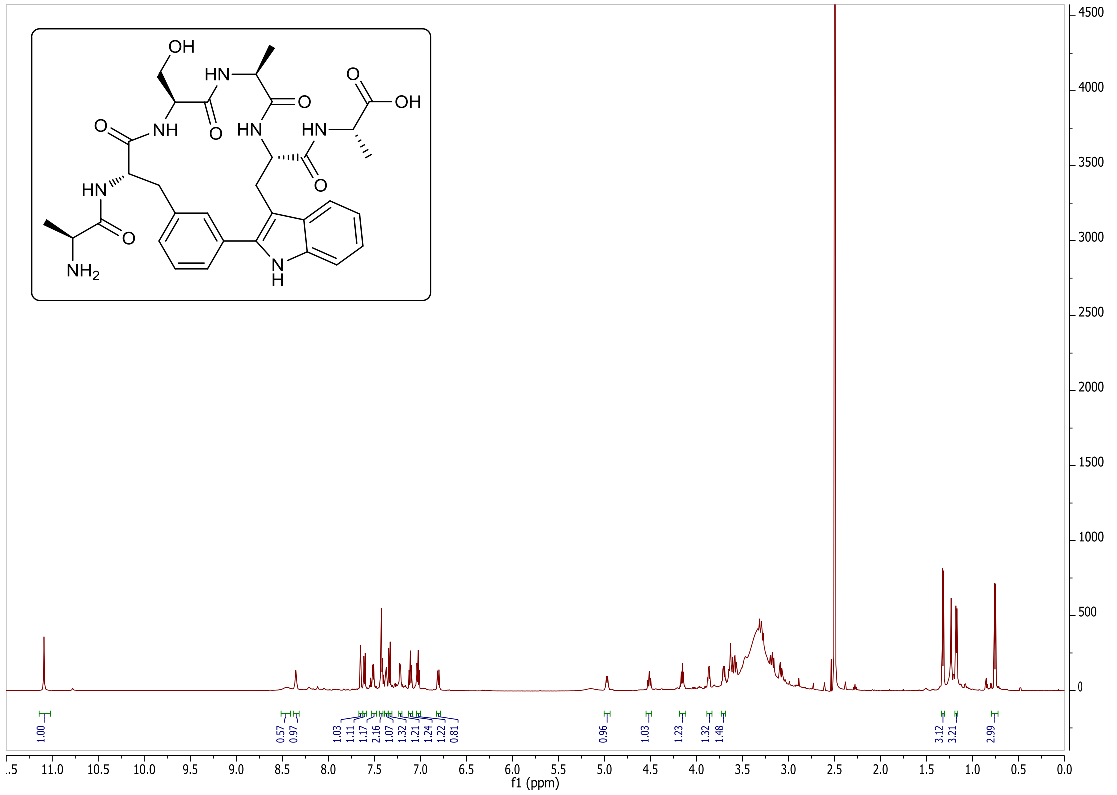

**Supplementary Figure 68 | $^1$H NMR spectrum of compound H-Ala-(Cyclo-*m)*-[Phe-Ser-Ala-Trp]-Ala-OH (2i).**

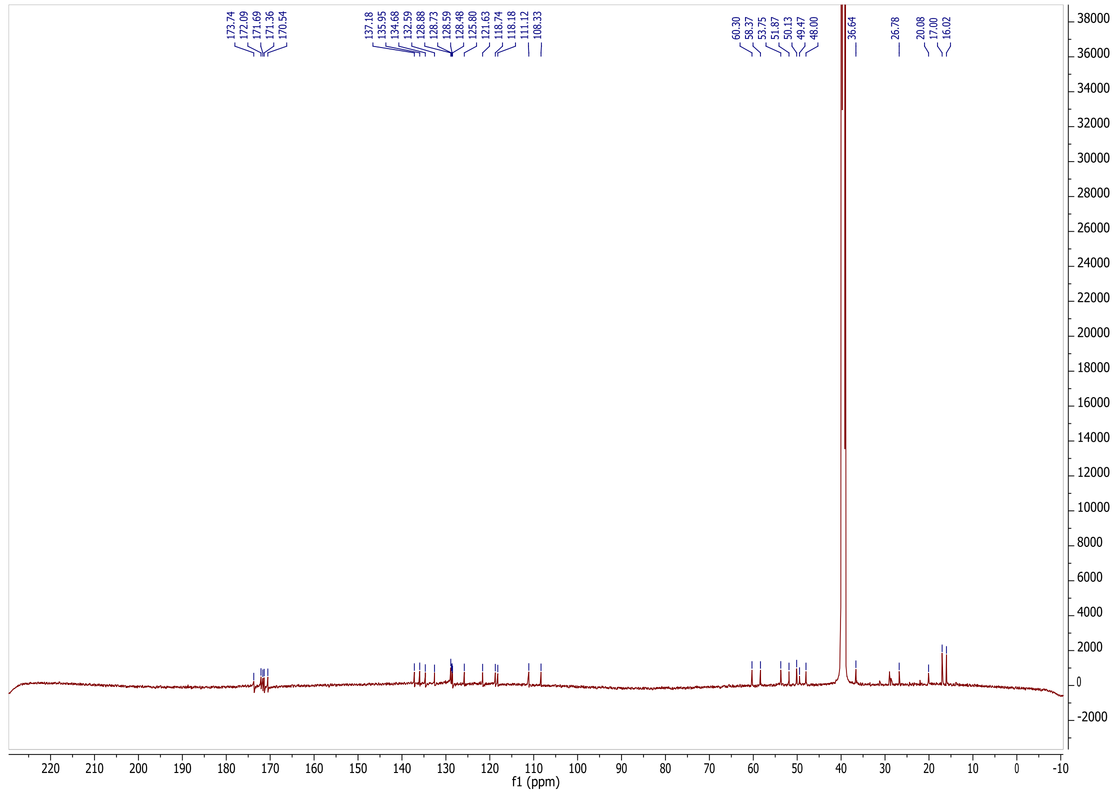

**Supplementary Figure 69 | $^{13}$C NMR spectrum of compound H-Ala-(Cyclo-*m)*-[Phe-Ser-Ala-Trp]-Ala-OH (2i).**

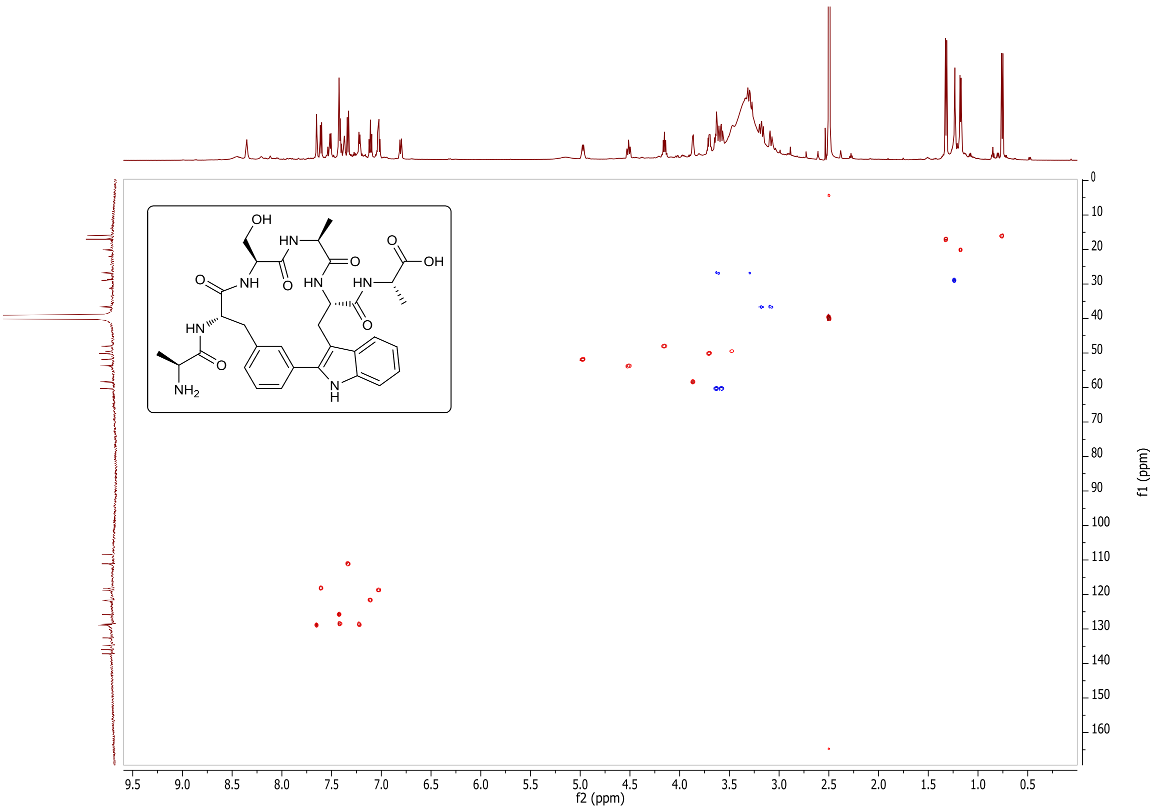

**Supplementary Figure 70 | $^{1}H$-$^{13}C$ HSQC NMR spectrum of compound H-Ala-(Cyclo-*m)*-[Phe-Ser-Ala-Trp]-Ala-OH (2i).**

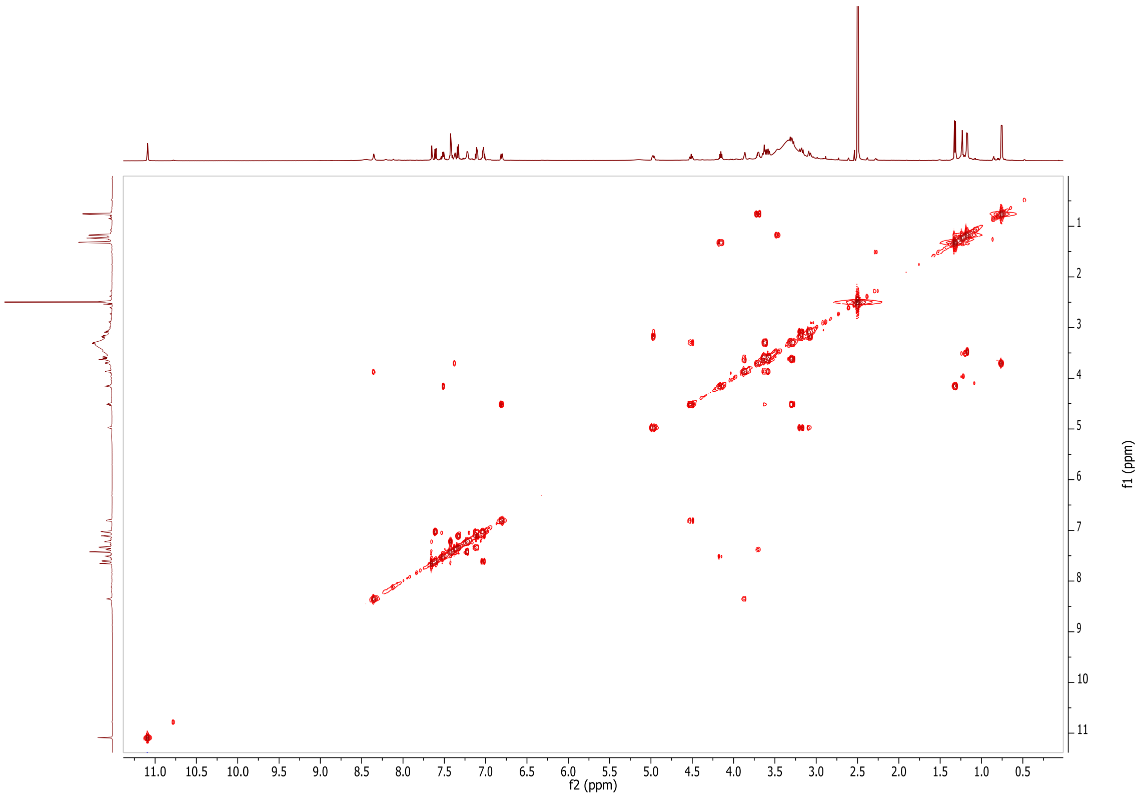

**Supplementary Figure 71 | COSY NMR spectrum of compound H-Ala-(Cyclo-*m)*-[Phe-Ser-Ala-Trp]-Ala-OH (2i).**

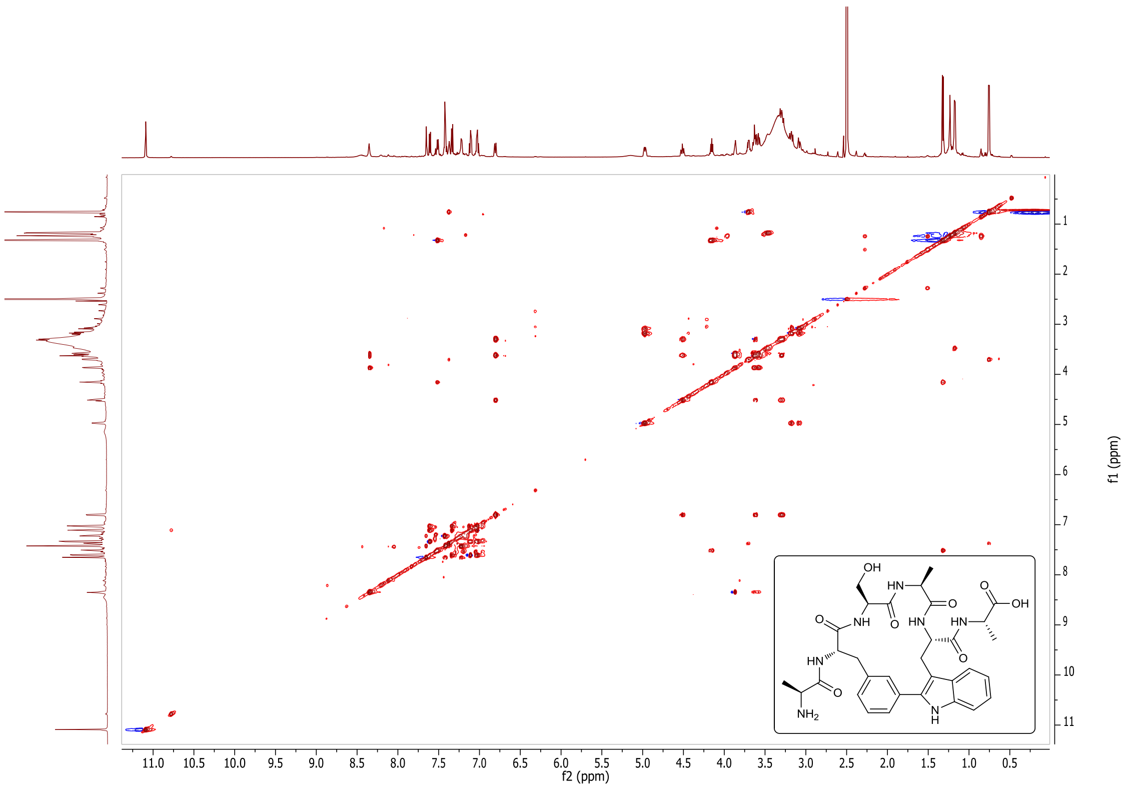


**Supplementary Figure 72 | TOCSY NMR spectrum of compound H-Ala-(Cyclo-*m)*-[Phe-Ser-Ala-Trp]-Ala-OH (2i).**

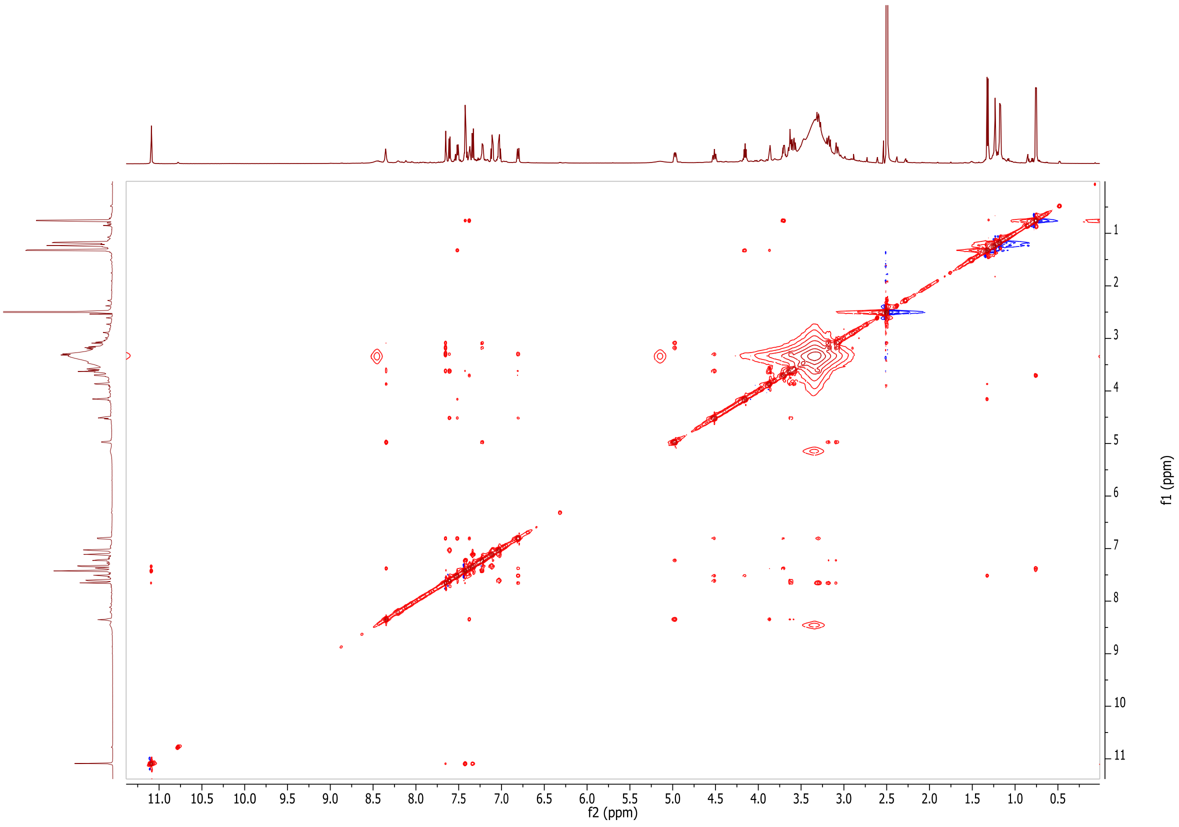


**Supplementary Figure 73 | NOESY NMR spectrum of compound H-Ala-(Cyclo-*m)*-[Phe-Ser-Ala-Trp]-Ala-OH (2i).**

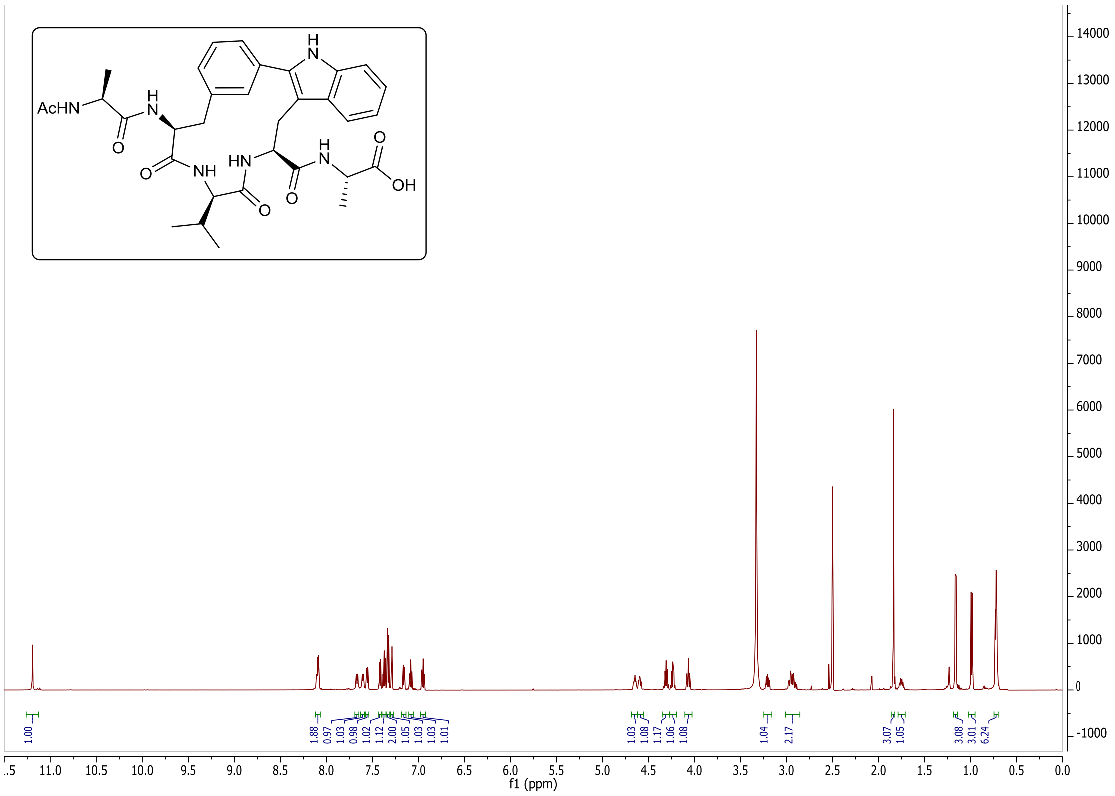

**Supplementary Figure 74 | $^{1}$H NMR spectrum of compound Ac-Ala-(Cyclo-*m*)-[Phe-Val-Trp]-Ala-OH (2j).**

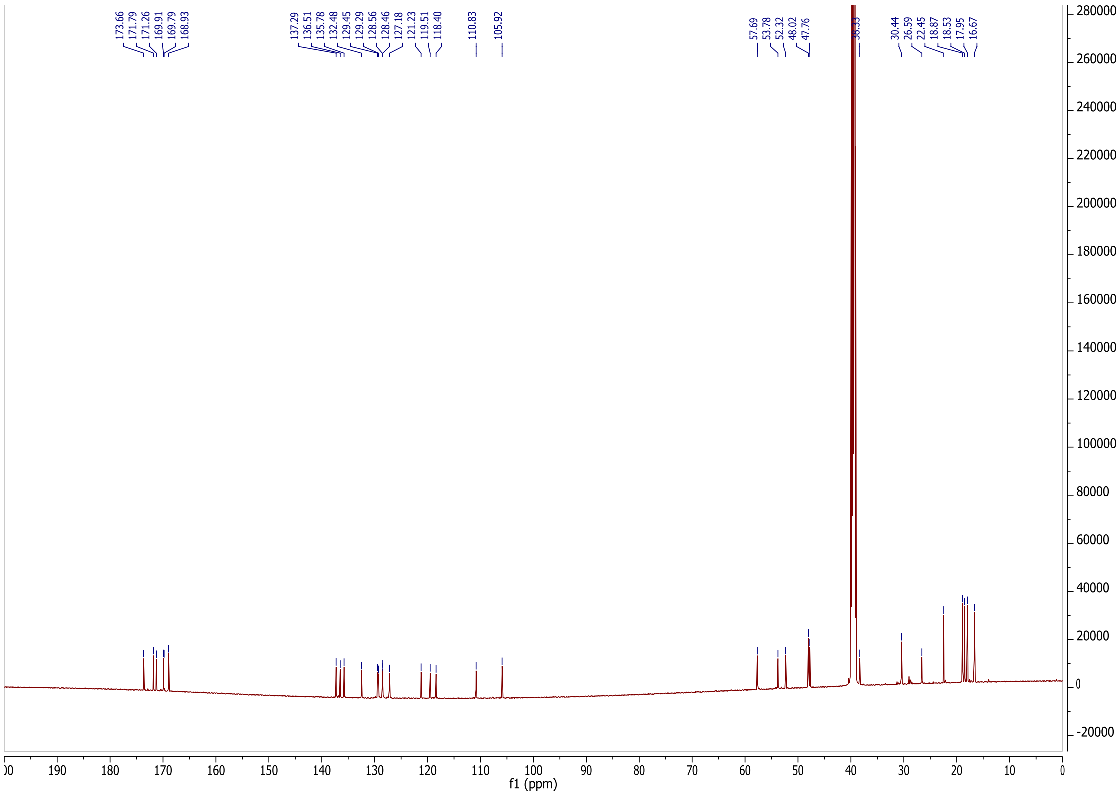

**Supplementary Figure 75 | $^{13}$C NMR spectrum of compound Ac-Ala-(Cyclo-*m*)-[Phe-Val-Trp]-Ala-OH (2j).**

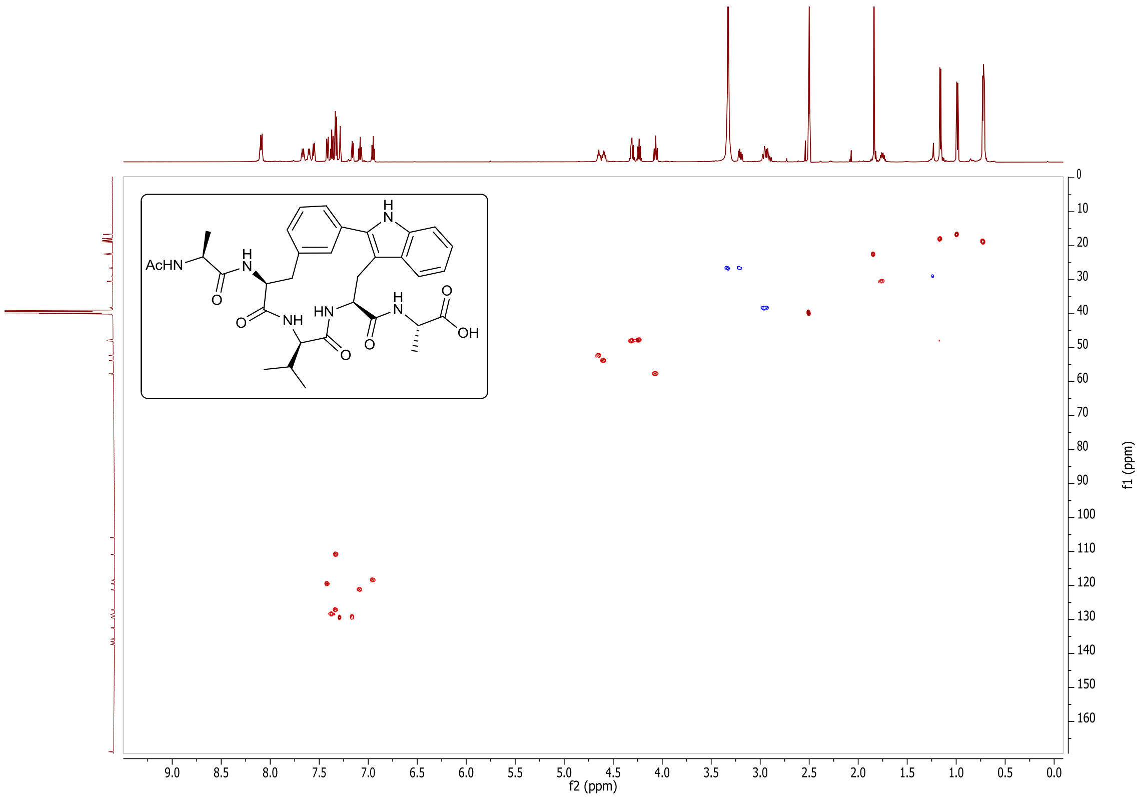


**Supplementary Figure 76 | $^{1}$H-$^{13}$C HSQC NMR spectrum of compound Ac-Ala-(Cyclo-*m)*-[Phe-Val-Trp]-Ala-OH (2j).**

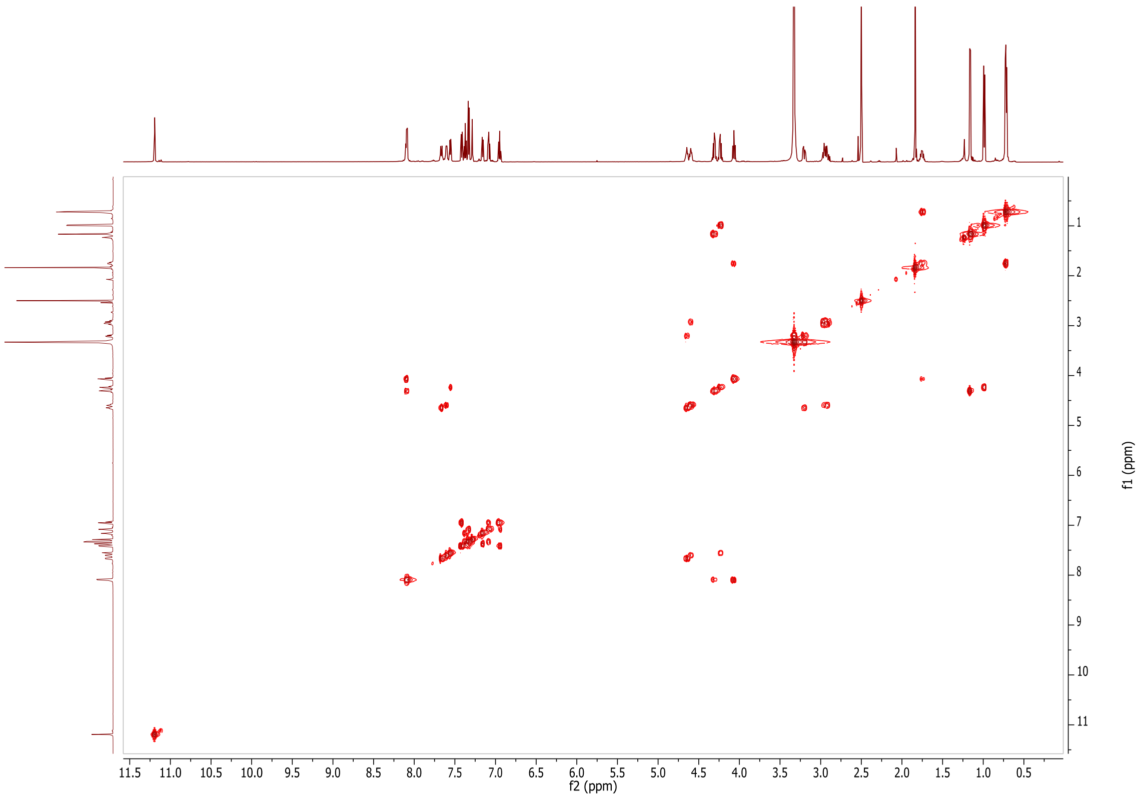


**Supplementary Figure 77 | COSY NMR spectrum of compound Ac-Ala-(Cyclo-*m)*-[Phe-Val-Trp]-Ala-OH (2j).**

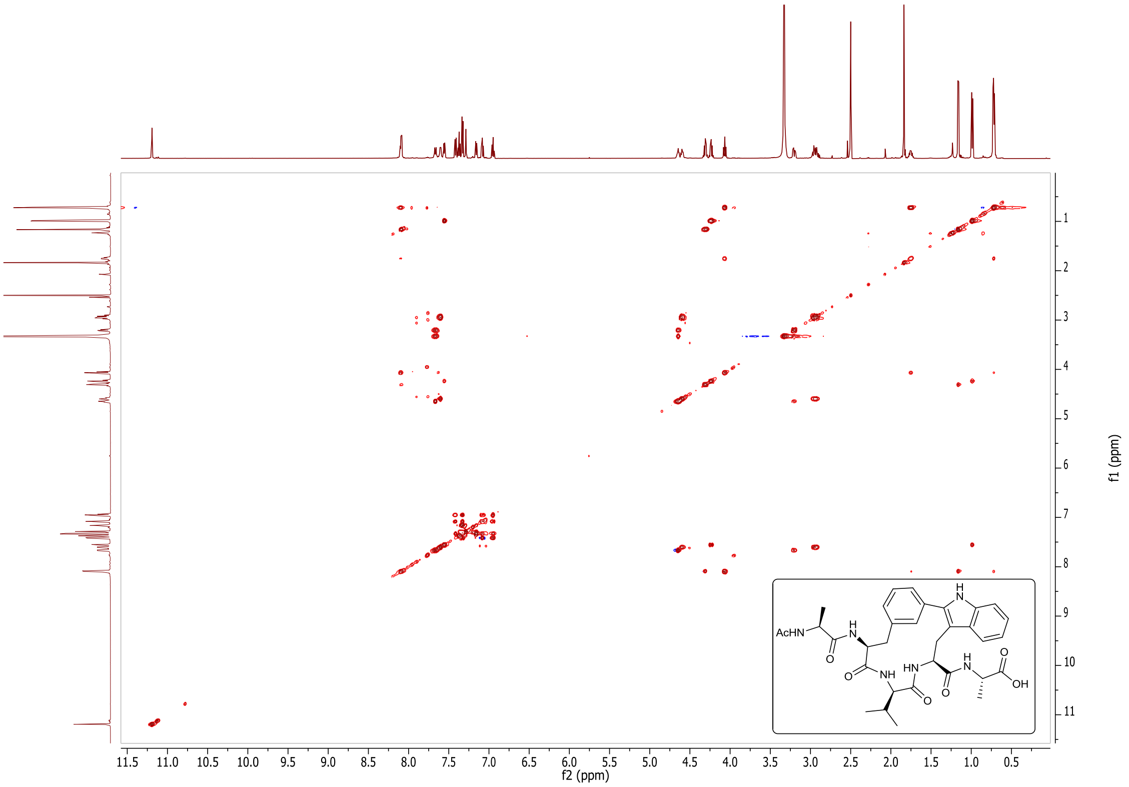


**Supplementary Figure 78 | TOCSY NMR spectrum of compound Ac-Ala-(Cyclo-*m)*-[Phe-Val-Trp]-Ala-OH (2j).**

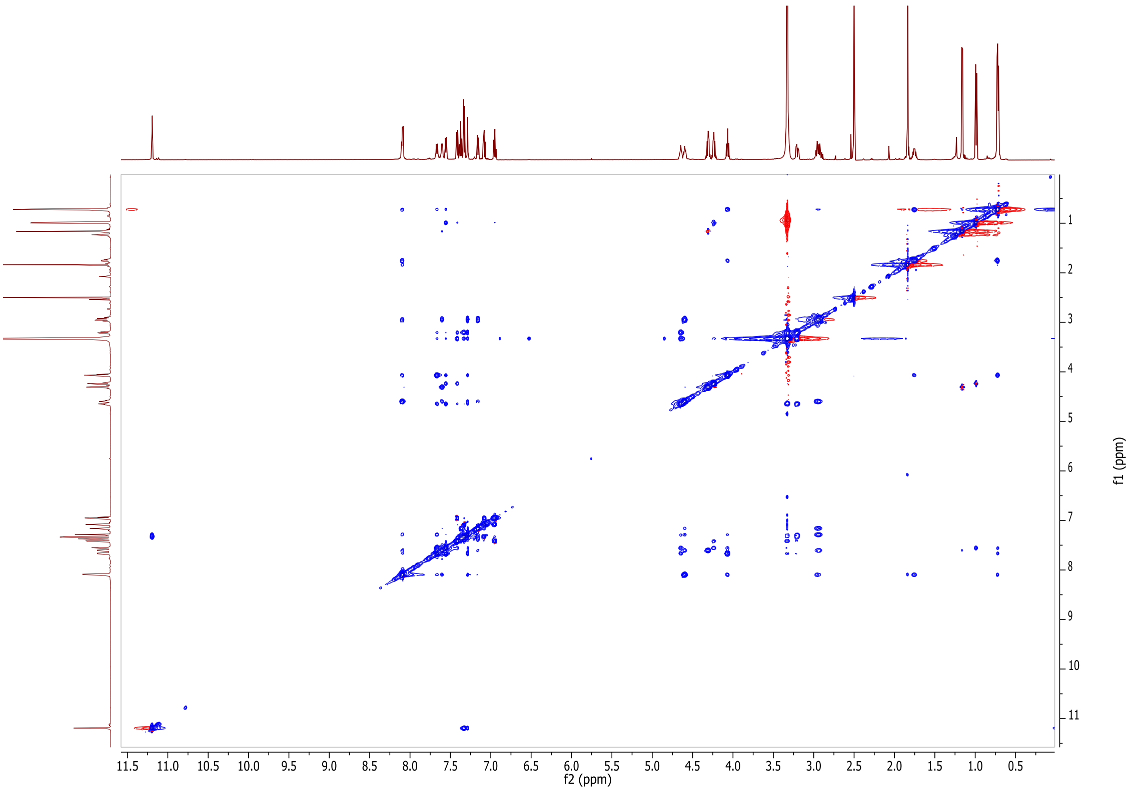


**Supplementary Figure 79 | NOESY NMR spectrum of compound Ac-Ala-(Cyclo-*m)*-[Phe-Val-Trp]-Ala-OH (2j).**

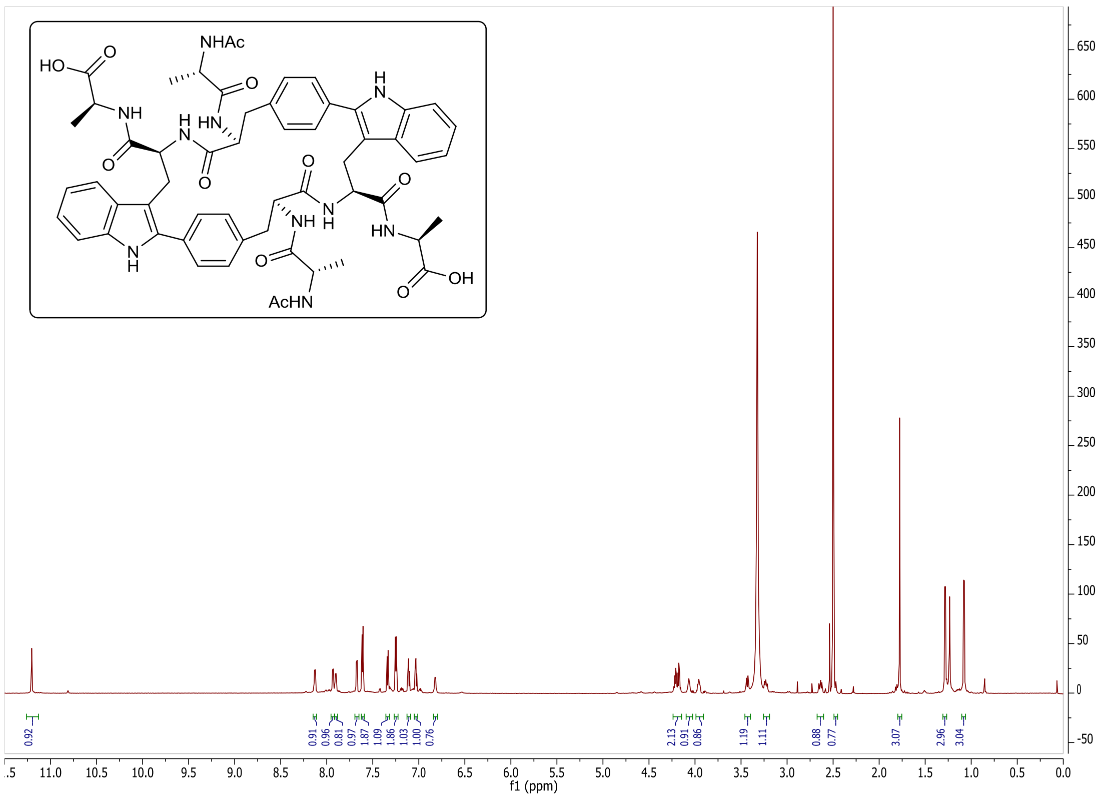

**Supplementary Figure 80 | $^{1}H$ NMR spectrum of compound (Cyclo-*p,p)*bis-[Phe-Trp]-(Ac-Ala-Phe-Trp-Ala-OH) (2k).**

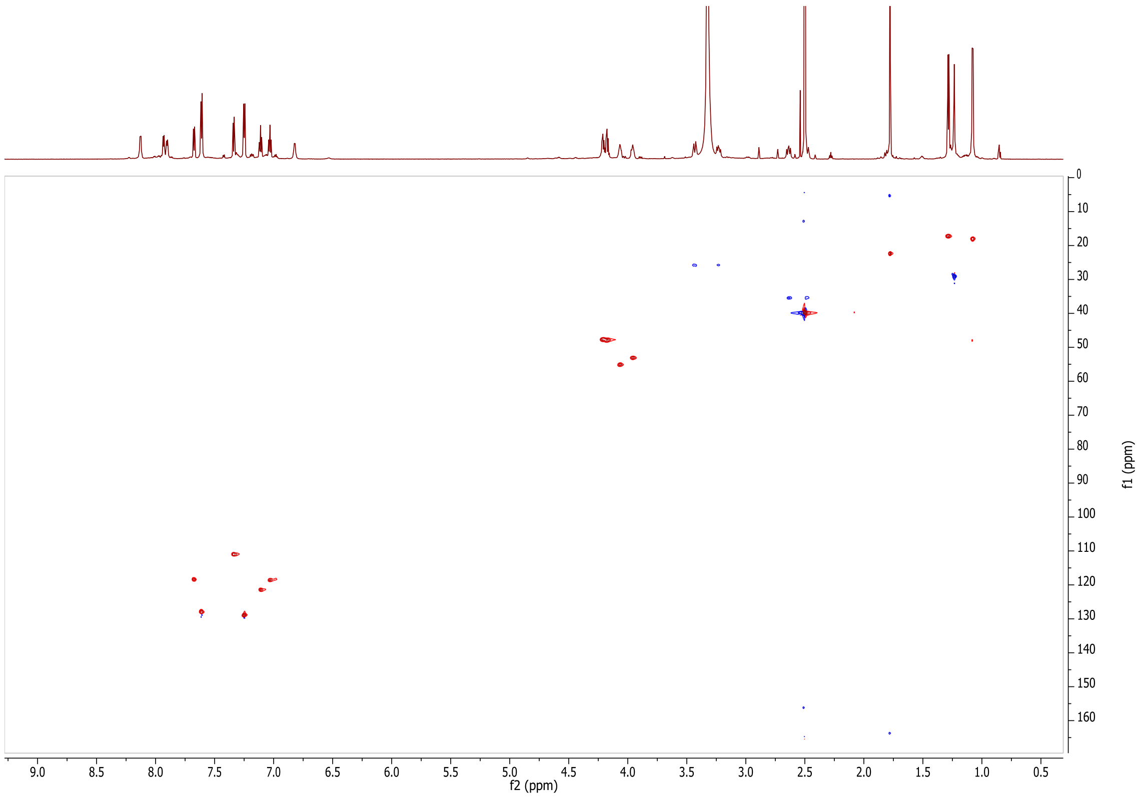

**Supplementary Figure 81 | $^{1}H$-$^{13}C$ HSQC NMR spectrum of compound (Cyclo-*p,p)*bis-[Phe-Trp]-(Ac-Ala-Phe-Trp-Ala-OH) (2k).**

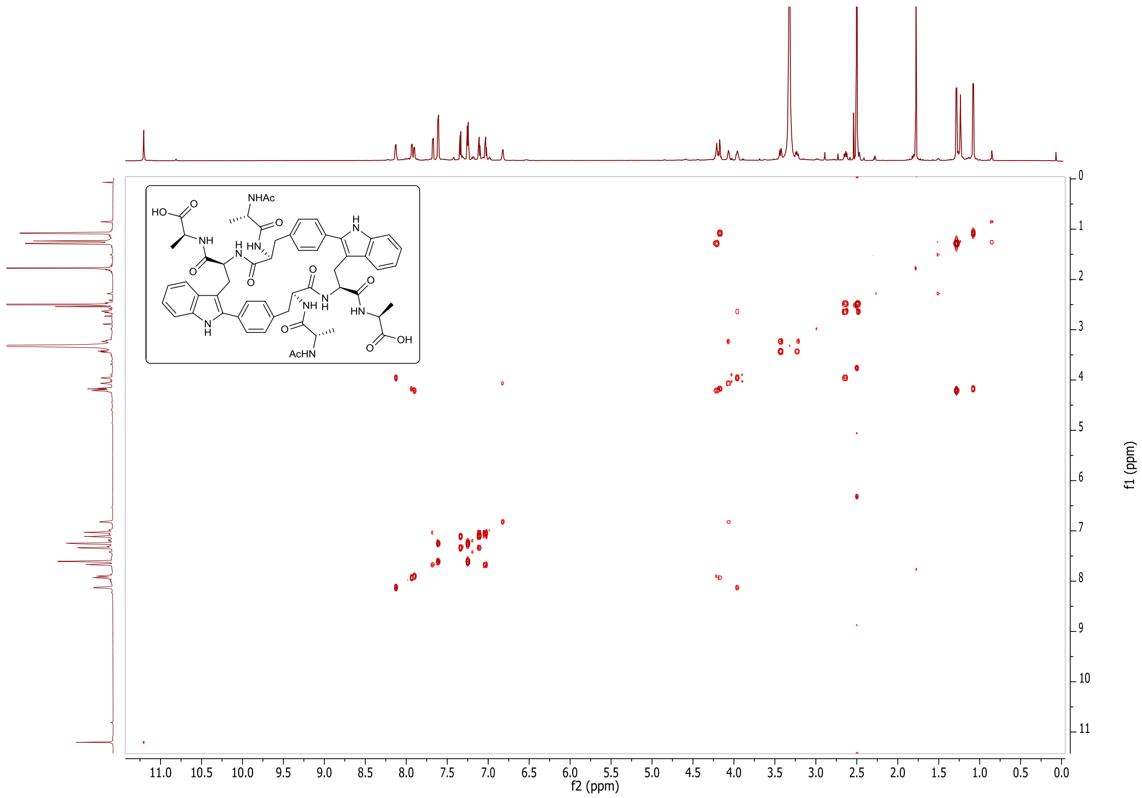


**Supplementary Figure 82 | COSY NMR spectrum of compound (Cyclo-*p,p)*bis-[Phe-Trp]-(Ac-Ala-Phe-Trp-Ala-OH) (2k).**

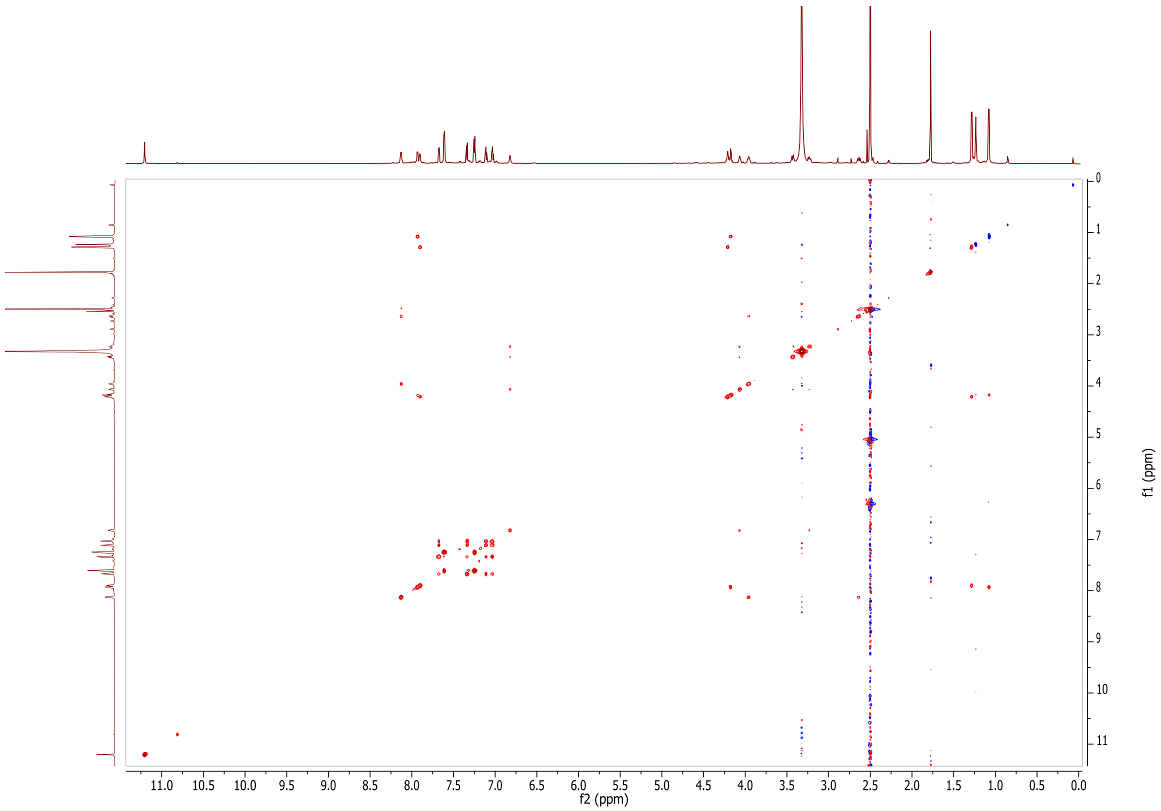


**Supplementary Figure 83 | TOCSY NMR spectrum of compound (Cyclo-*p,p)*bis-[Phe-Trp]-(Ac-Ala-Phe-Trp-Ala-OH) (2k).**

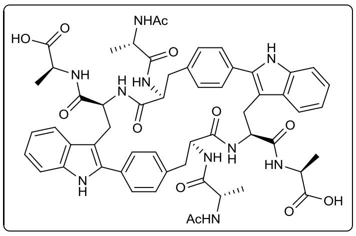

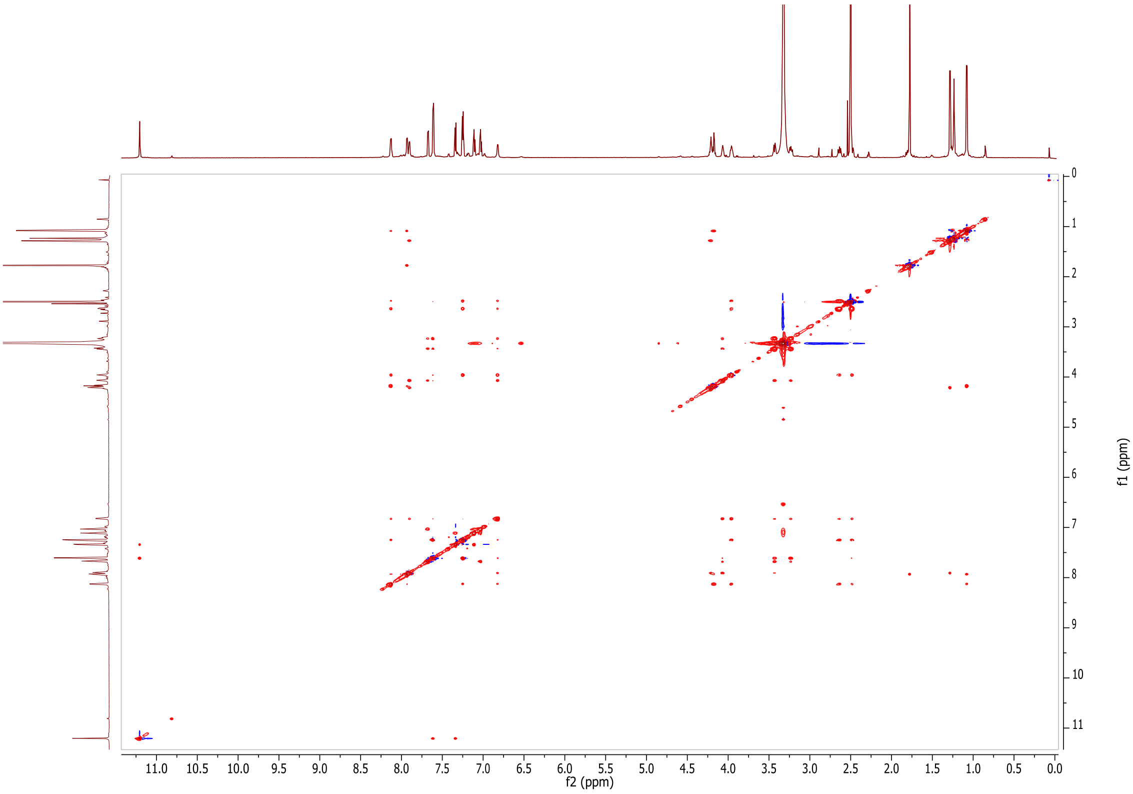

**Supplementary Figure 84 | NOESY NMR spectrum of compound (Cyclo-*p,p)*bis-[Phe-Trp]-(Ac-Ala-Phe-Trp-Ala-OH) (2k).**

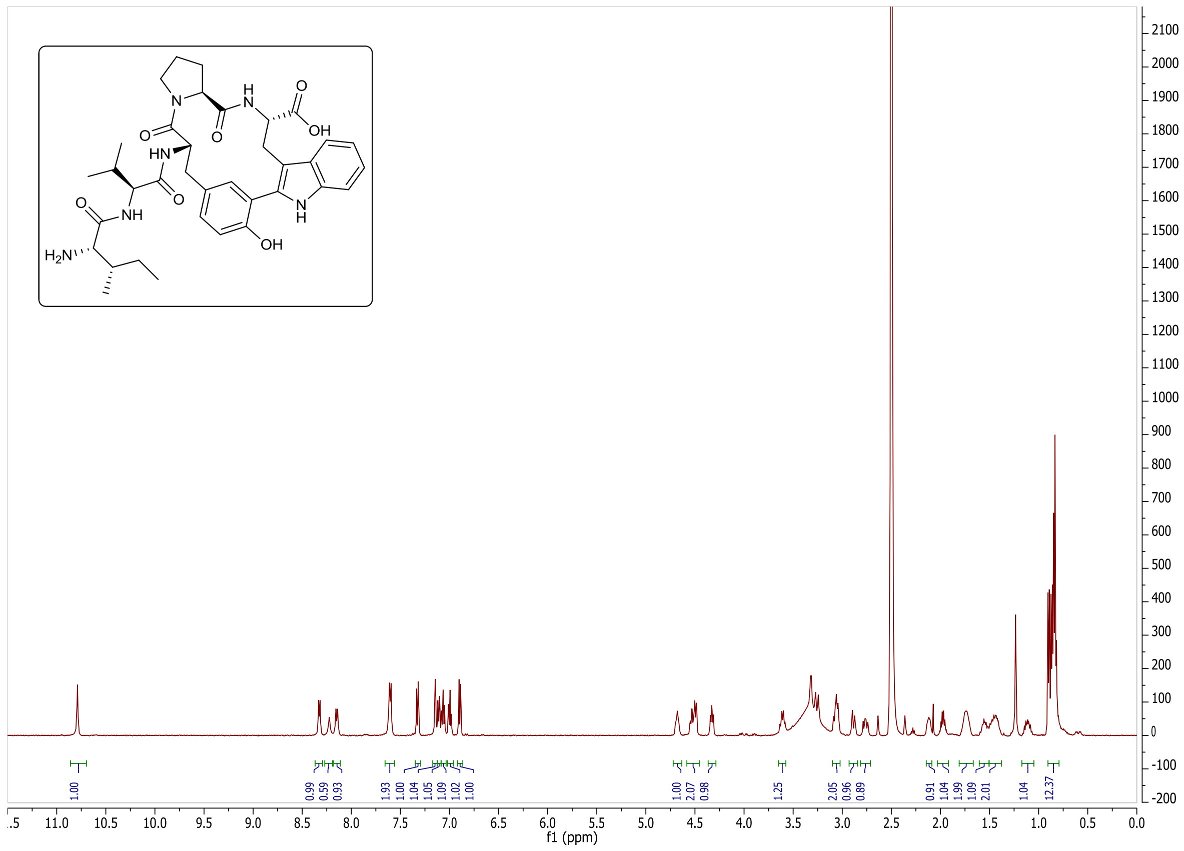

**Supplementary Figure 85 | $^{1}$H NMR spectrum of compound H-Ile-Val-(Cyclo-*m*)-[Tyr-Pro-Trp]-OH (2l).**

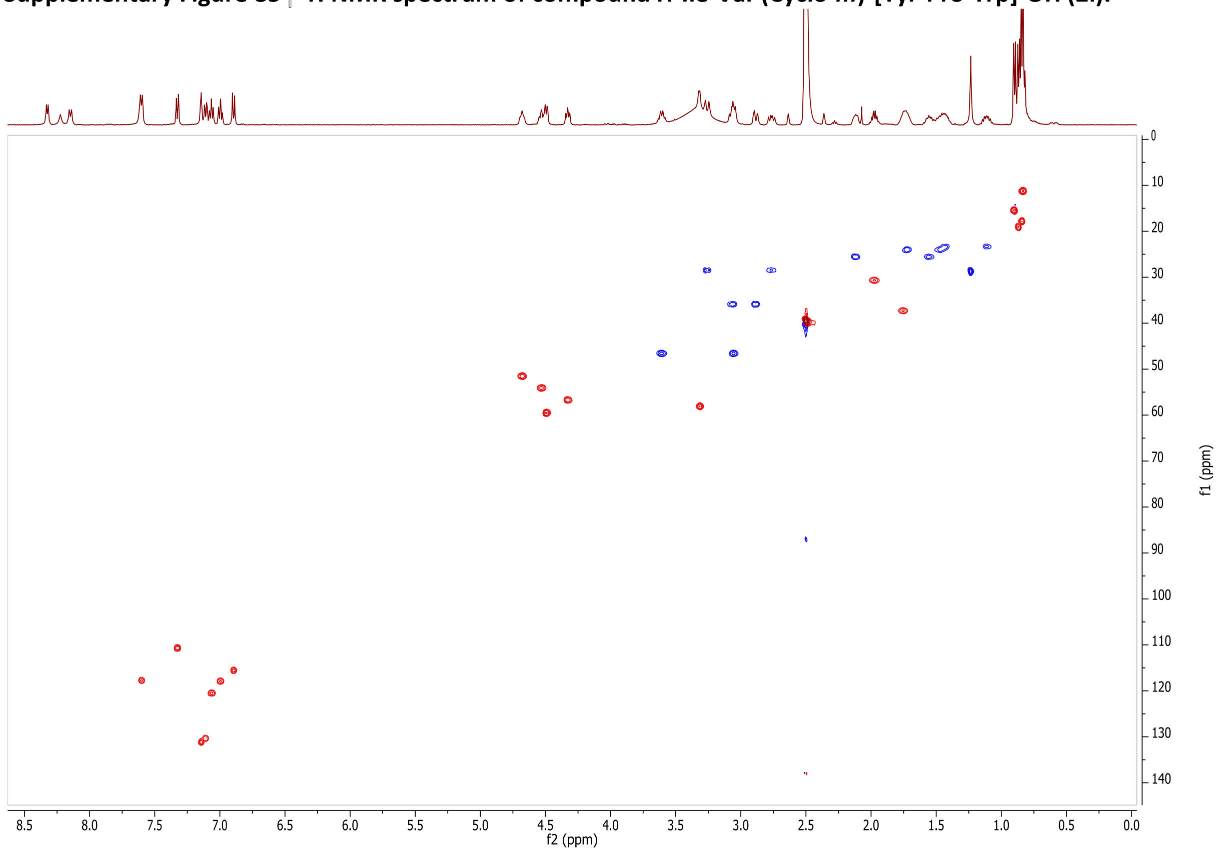

**Supplementary Figure 86 | $^{1}$H-$^{13}$C HSQC NMR spectrum of compound H-Ile-Val-(Cyclo-*m*)-[Tyr-Pro-Trp]-OH (2l).**

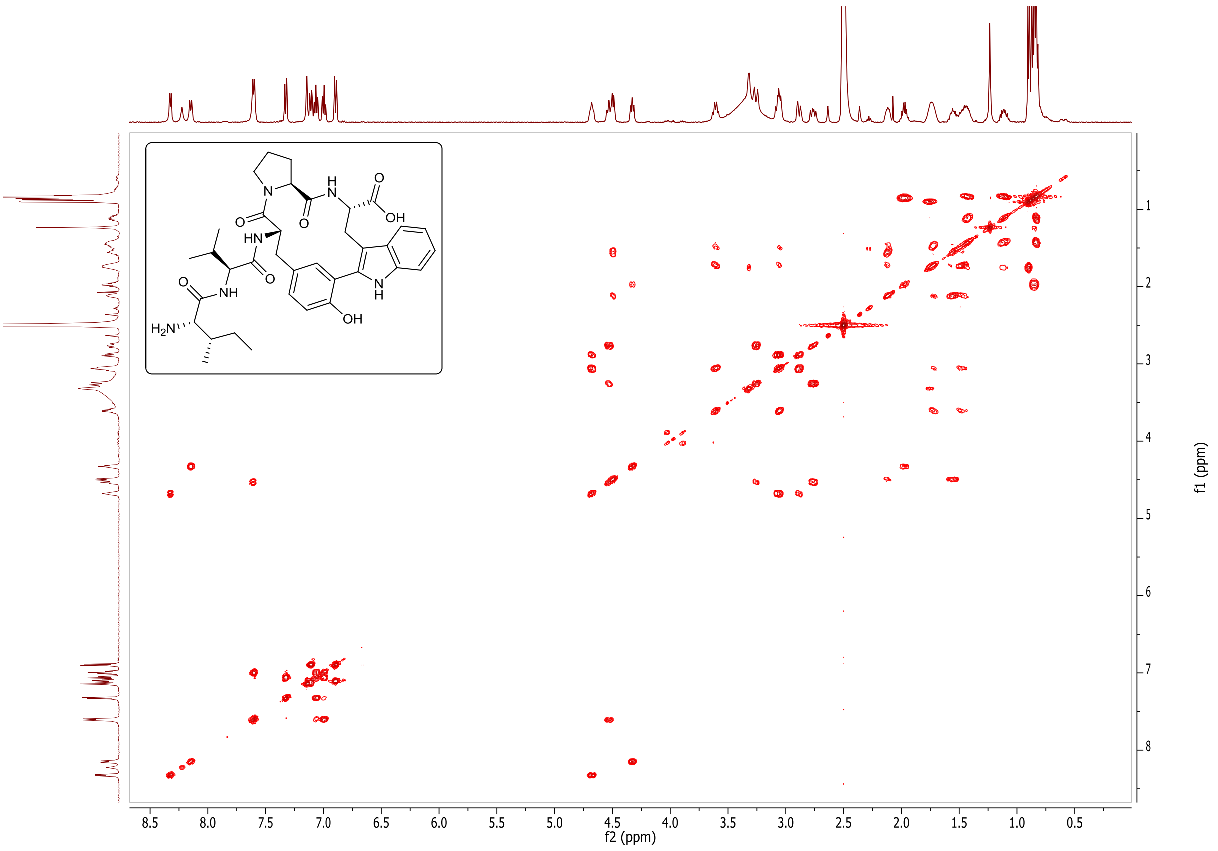


**Supplementary Figure 87 | COSY NMR spectrum of compound H-Ile-Val-(Cyclo-*m*)-[Tyr-Pro-Trp]-OH (2l).**

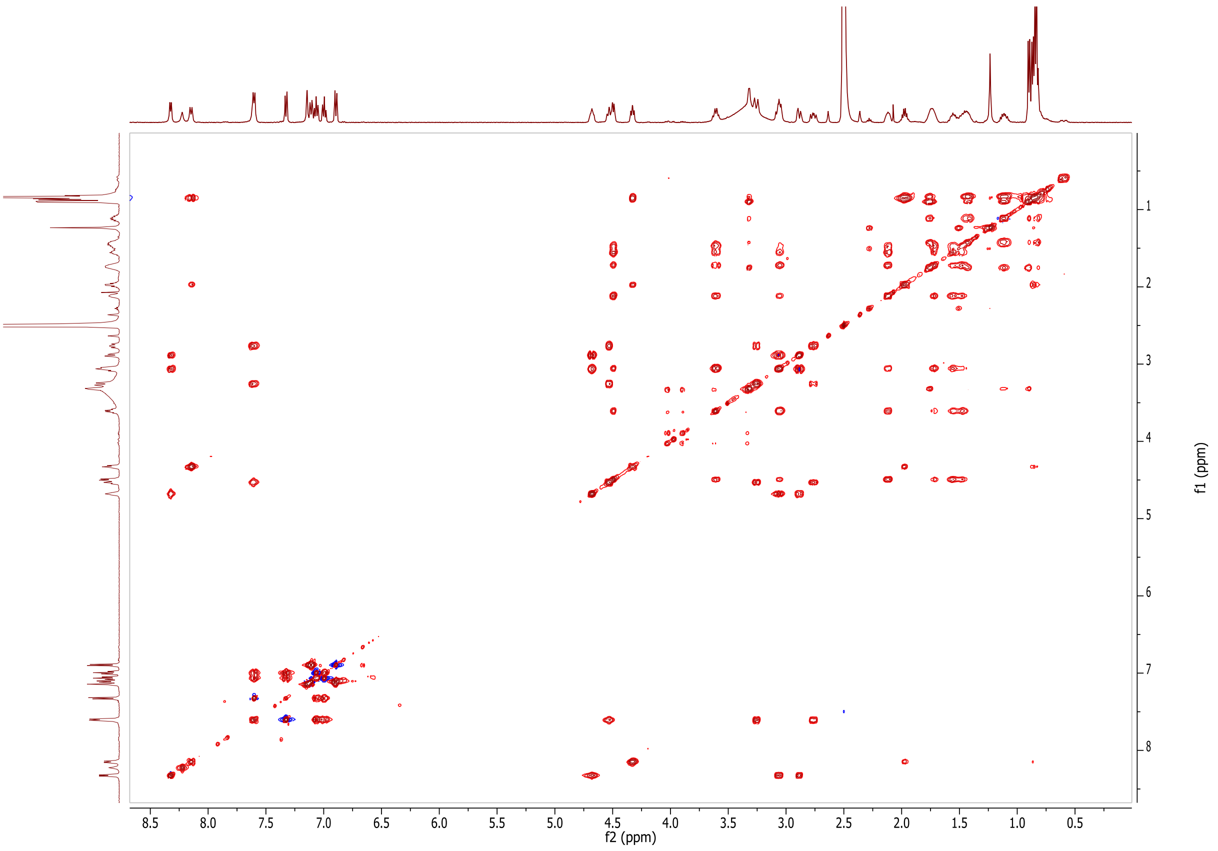


**Supplementary Figure 88 | TOCSY NMR spectrum of compound H-Ile-Val-(Cyclo-*m*)-[Tyr-Pro-Trp]-OH (2l).**

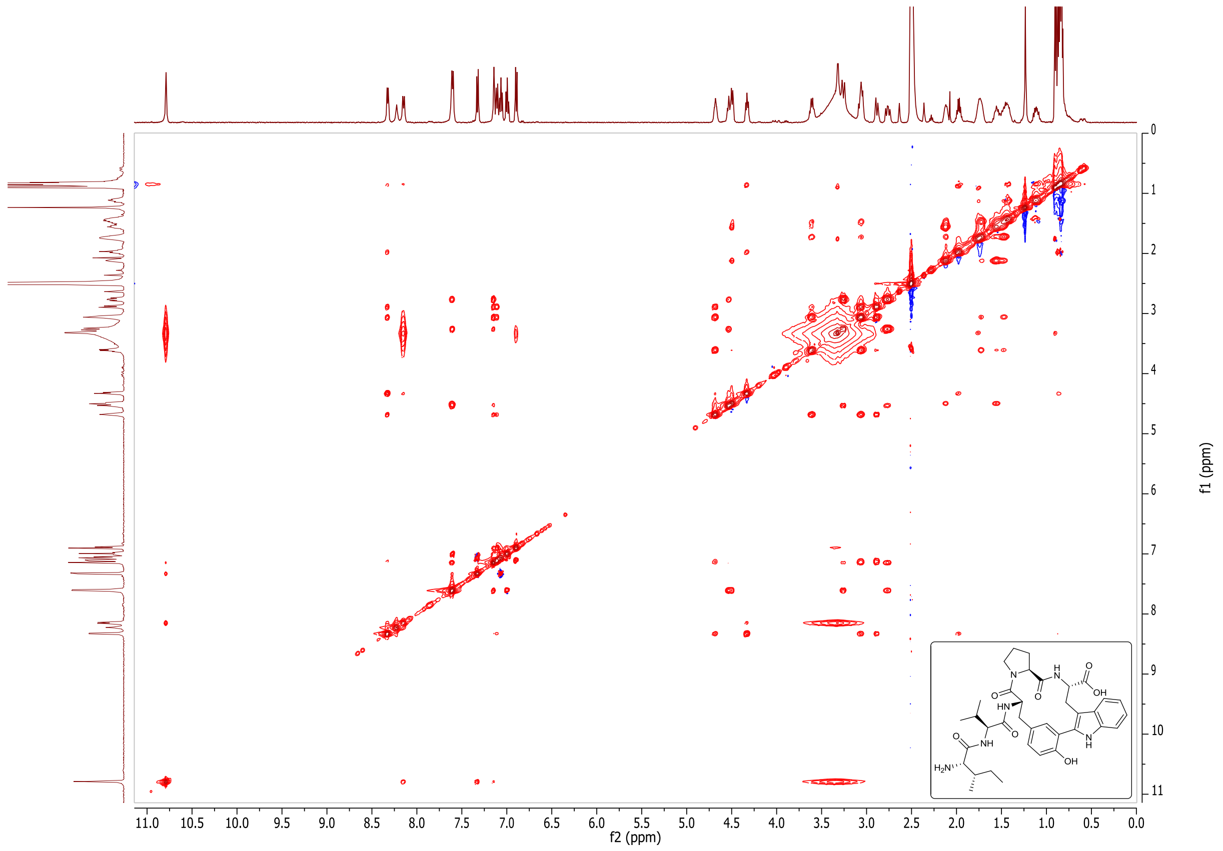


**Supplementary Figure 89 | NOESY NMR spectrum of compound H-Ile-Val-(Cyclo-*m*)-[Tyr-Pro-Trp]-OH (2l).**

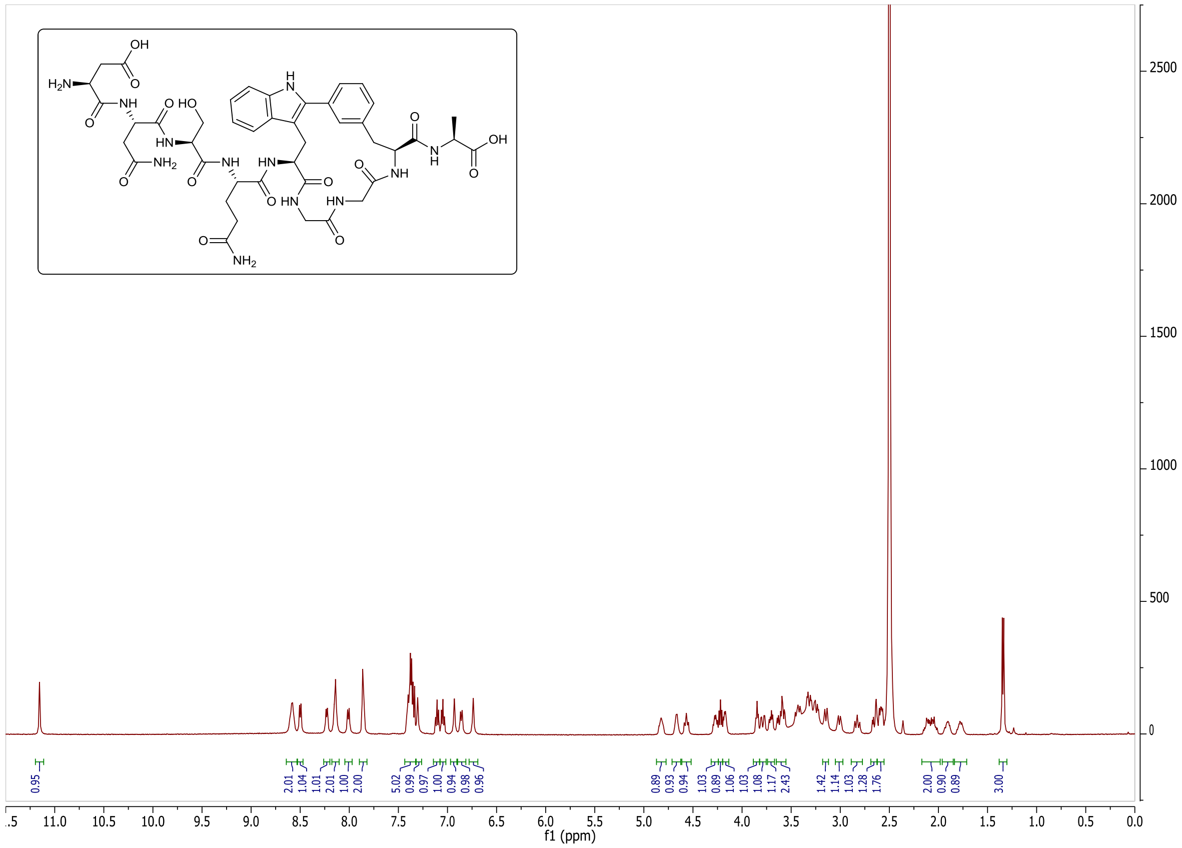

**Supplementary Figure 90 | $^{1}$H NMR spectrum of compound H-Asp-Asn-Ser-Gln-(Cyclo-*m*)-[Trp-Gly-Gly-Phe]-Ala-OH (2m).**

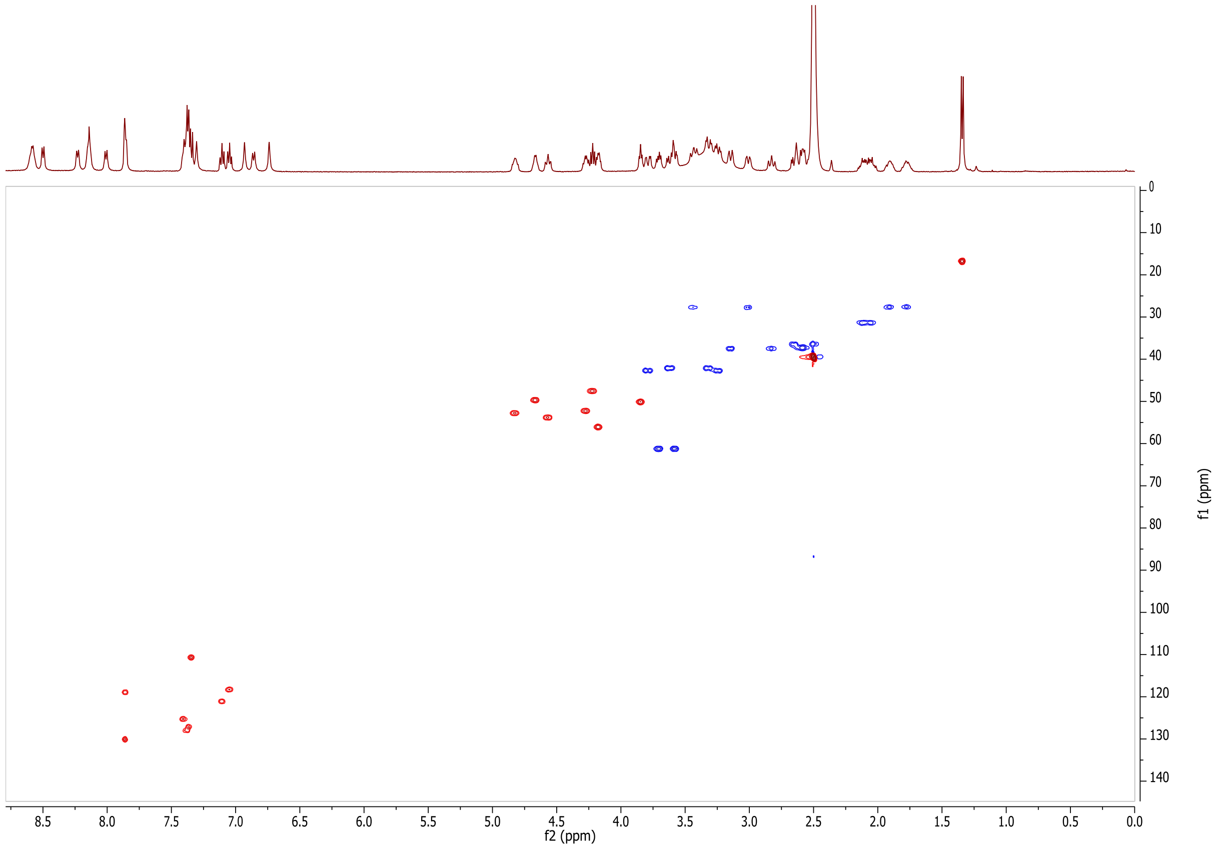

**Supplementary Figure 91 | $^{1}$H-$^{13}$C HSQC NMR spectrum of compound H-Asp-Asn-Ser-Gln-(Cyclo-*m*)-[Trp-Gly-Gly-Phe]-Ala-OH (2m).**

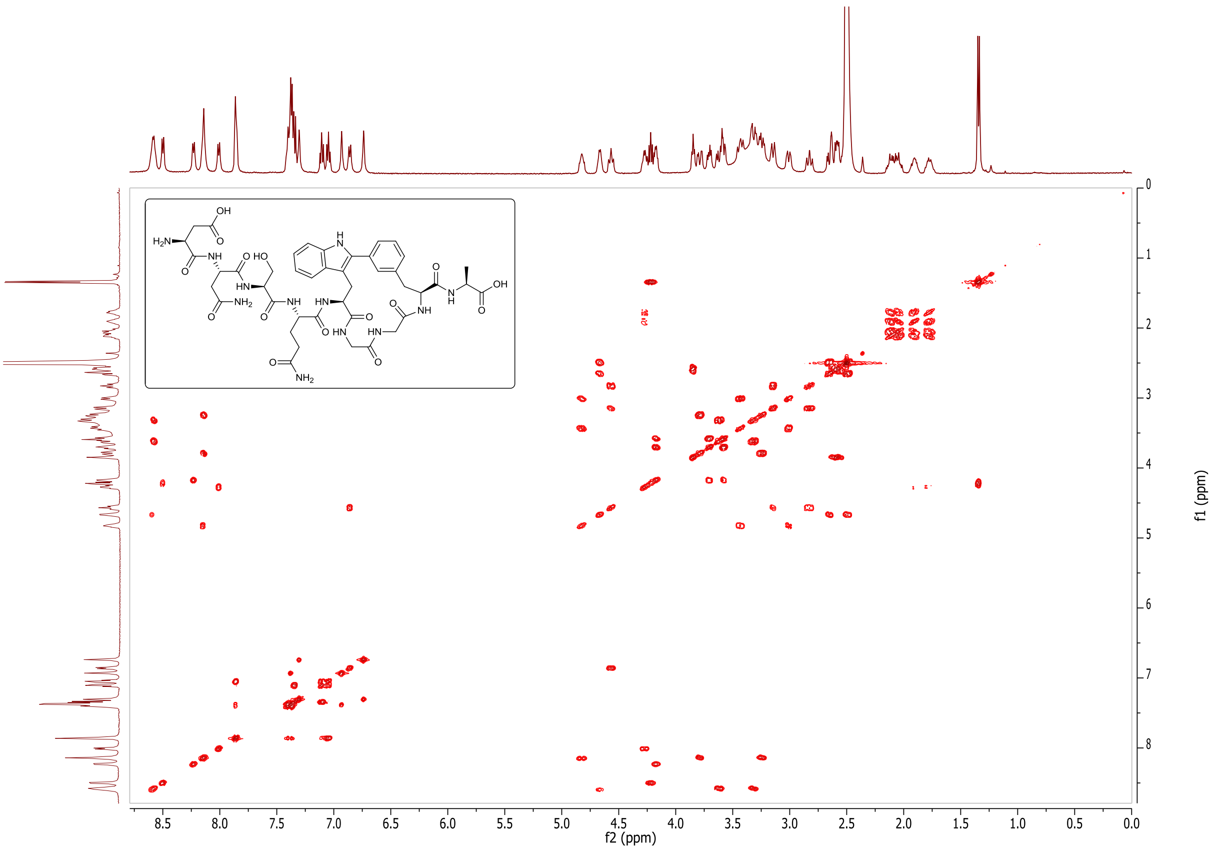


**Supplementary Figure 92 | COSY NMR spectrum of compound H-Asp-Asn-Ser-Gln-(Cyclo-*m*)-[Trp-Gly-Gly-Phe]-Ala-OH (2m).**

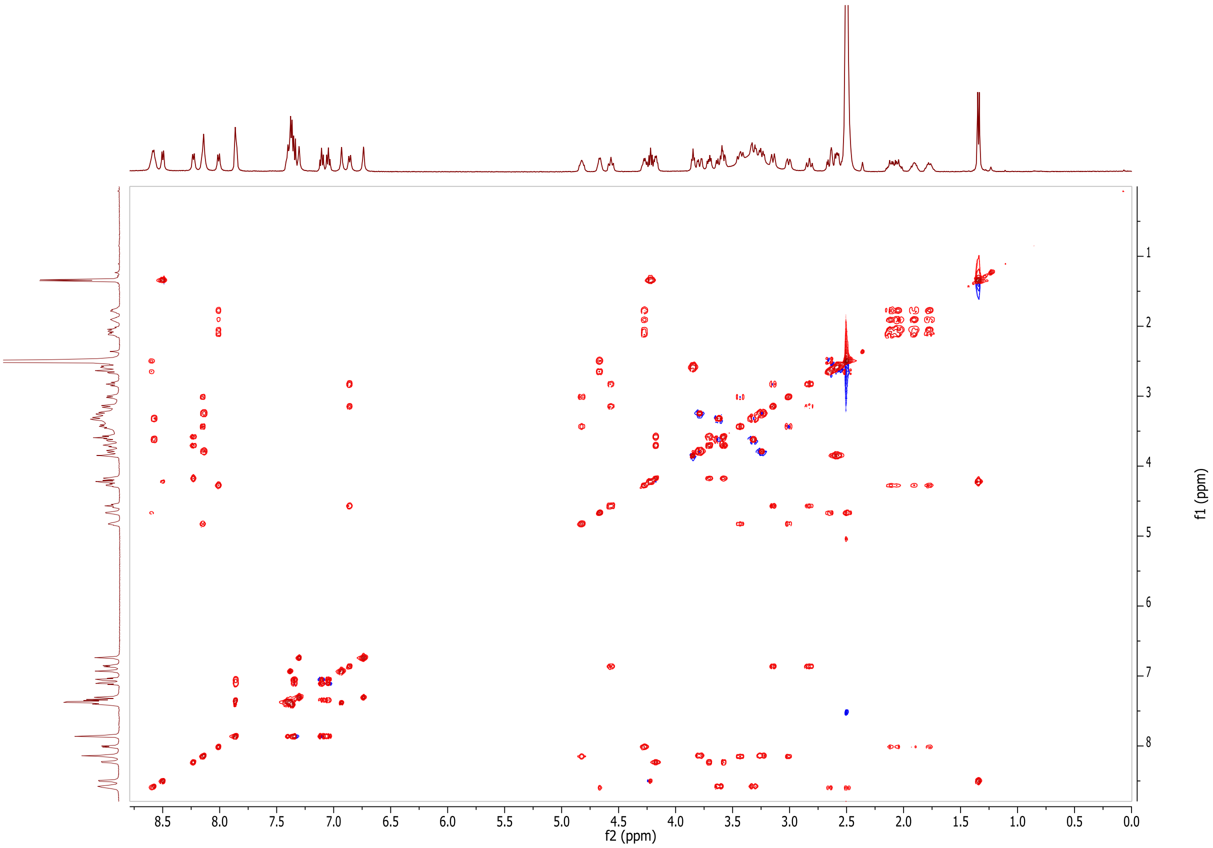


**Supplementary Figure 93 | TOCSY NMR spectrum of compound H-Asp-Asn-Ser-Gln-(Cyclo-*m*)-[Trp-Gly-Gly-Phe]-Ala-OH (2m).**

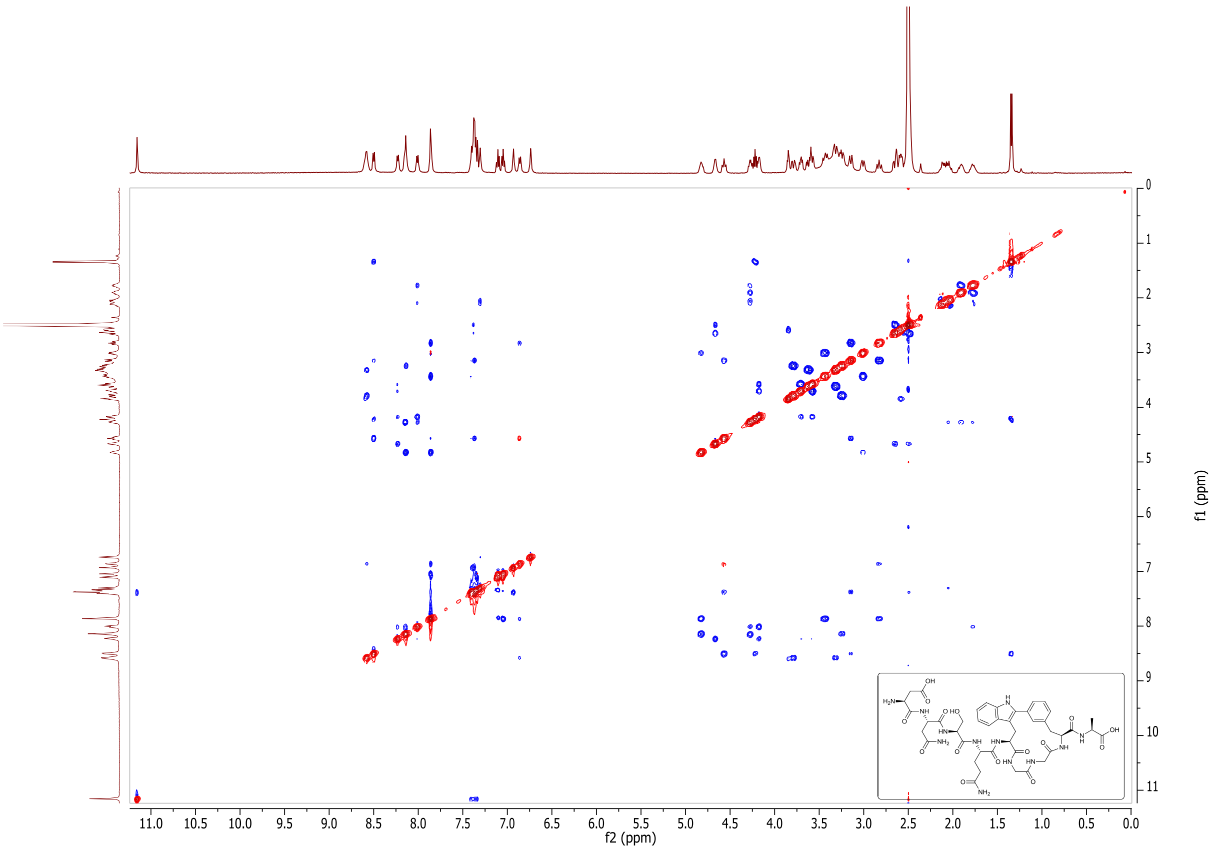


**Supplementary Figure 94 | ROESY NMR spectrum of compound H-Asp-Asn-Ser-Gln-(Cyclo-*m*)-[Trp-Gly-Gly-Phe]-Ala-OH (2m).**

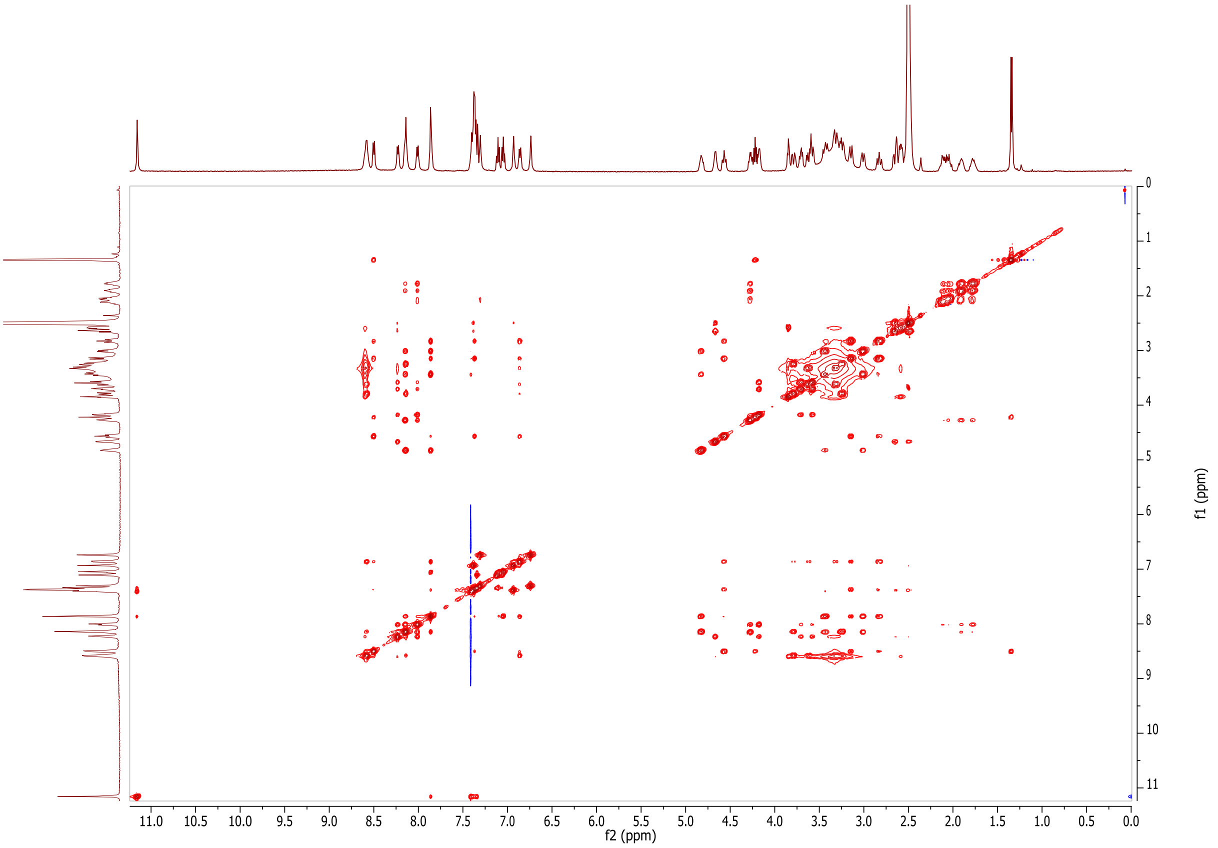


**Supplementary Figure 95 | NOESY NMR spectrum of compound H-Asp-Asn-Ser-Gln-(Cyclo-*m*)-[Trp-Gly-Gly-Phe]-Ala-OH (2m).**

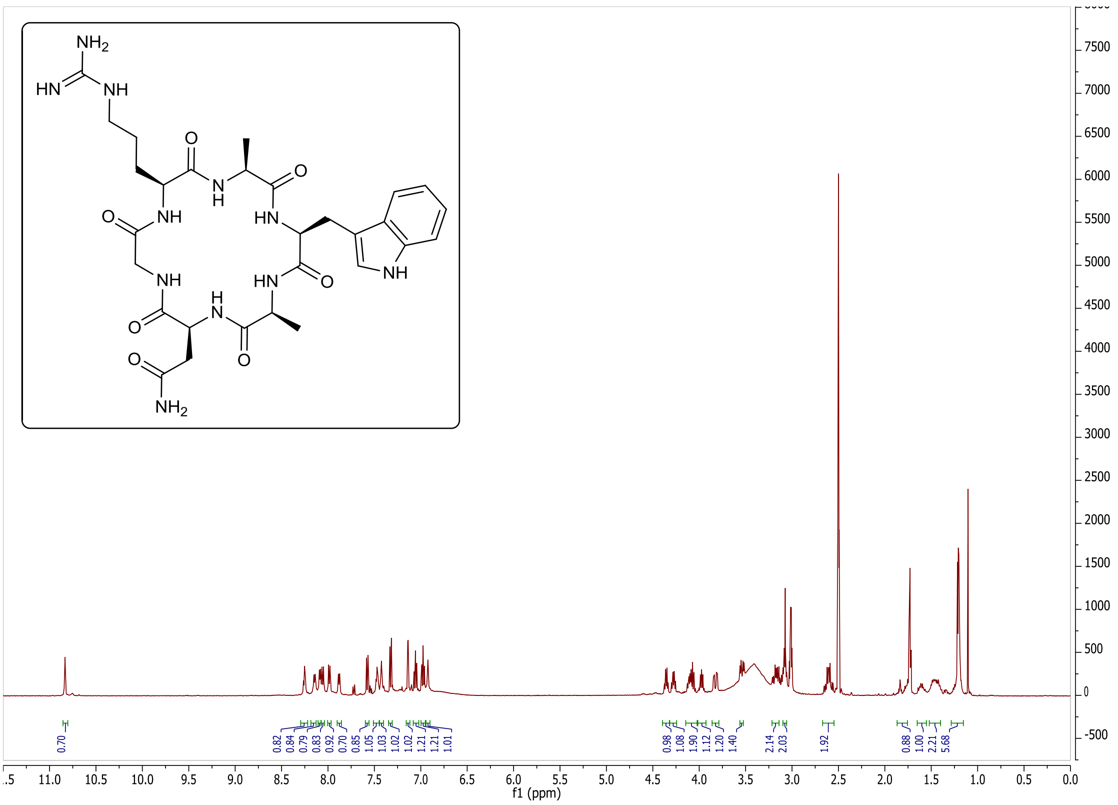


**Supplementary Figure 96 | $^{1}$H NMR spectrum of compound Cyclo(-Arg-Ala-Trp-Ala-Asn-Gly-) (3).**

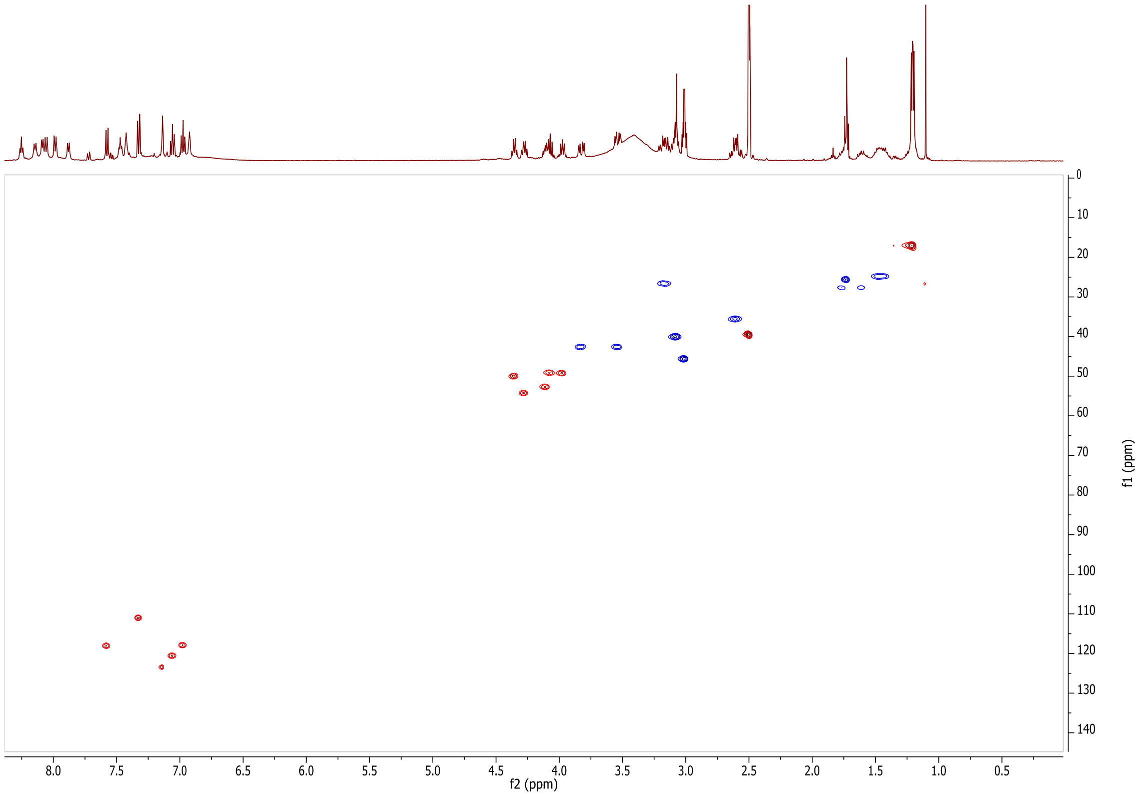


**Supplementary Figure 97 | $^{1}$H-$^{13}$C HSQ NMR spectrum of compound Cyclo(-Arg-Ala-Trp-Ala-Asn-Gly-) (3).**

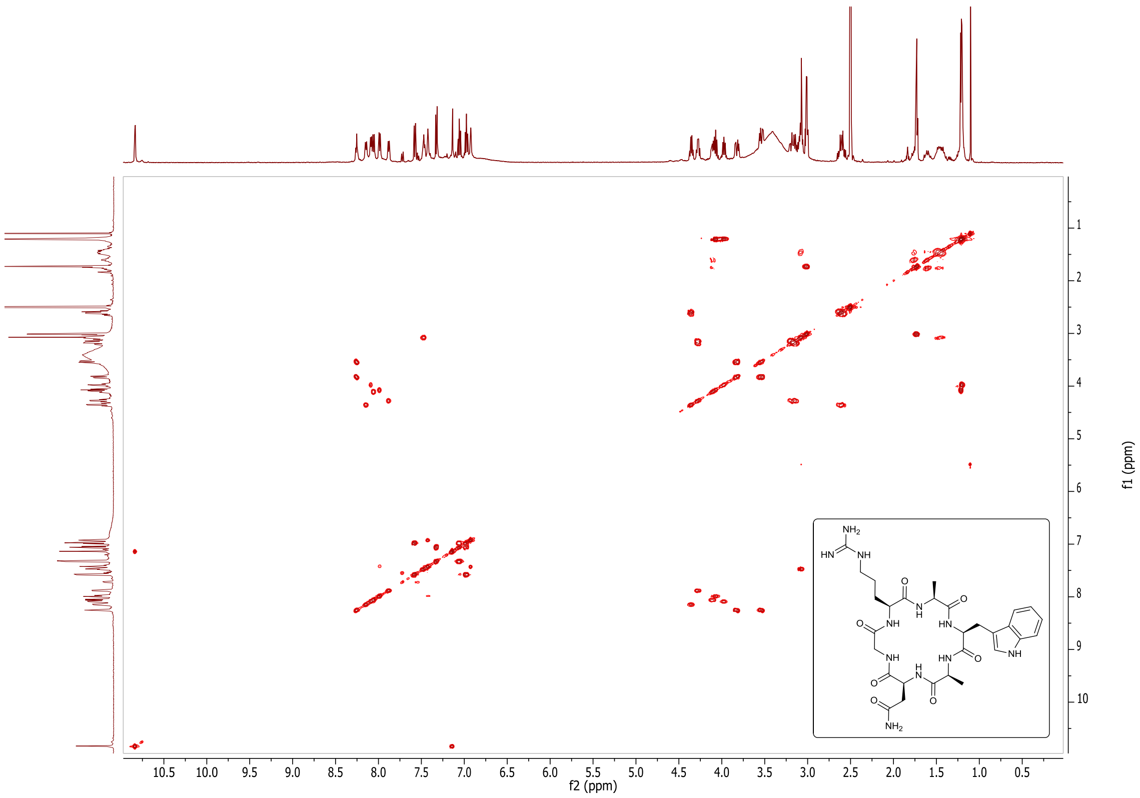


**Supplementary Figure 98 | COSY NMR spectrum of compound Cyclo(-Arg-Ala-Trp-Ala-Asn-Gly-) (3).**

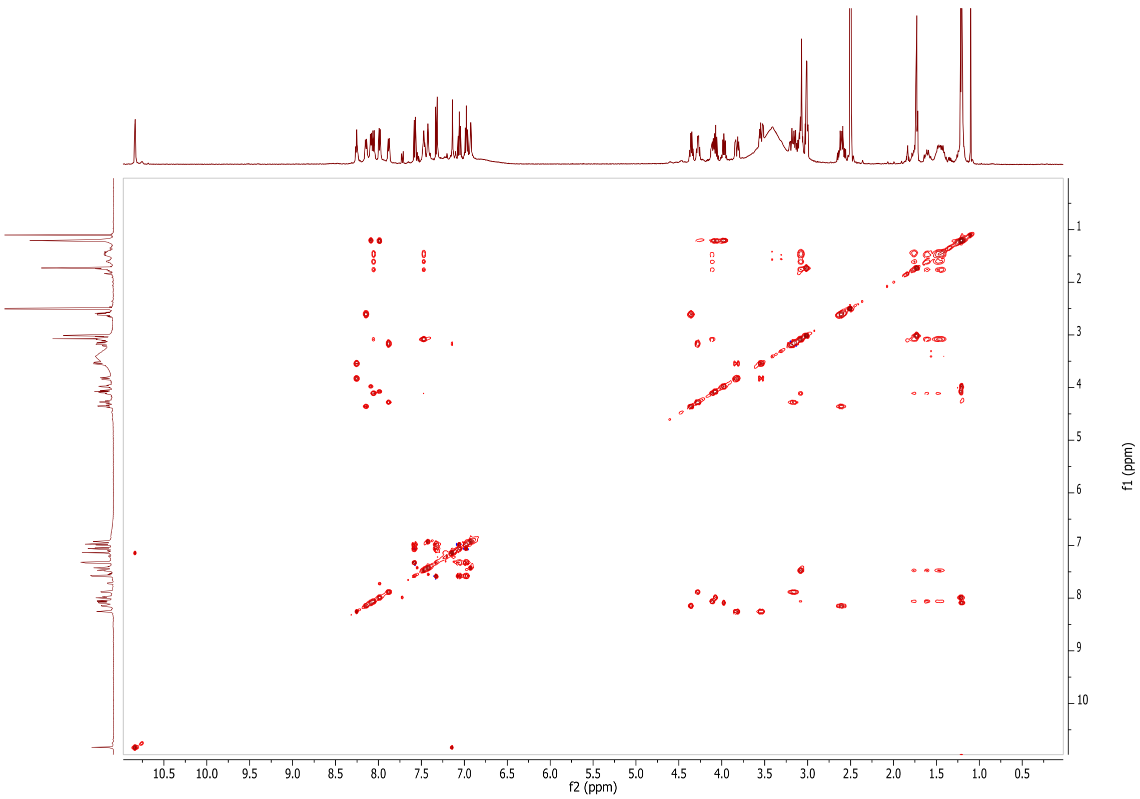


**Supplementary Figure 99 | TOCSY NMR spectrum of compound Cyclo(-Arg-Ala-Trp-Ala-Asn-Gly-) (3).**

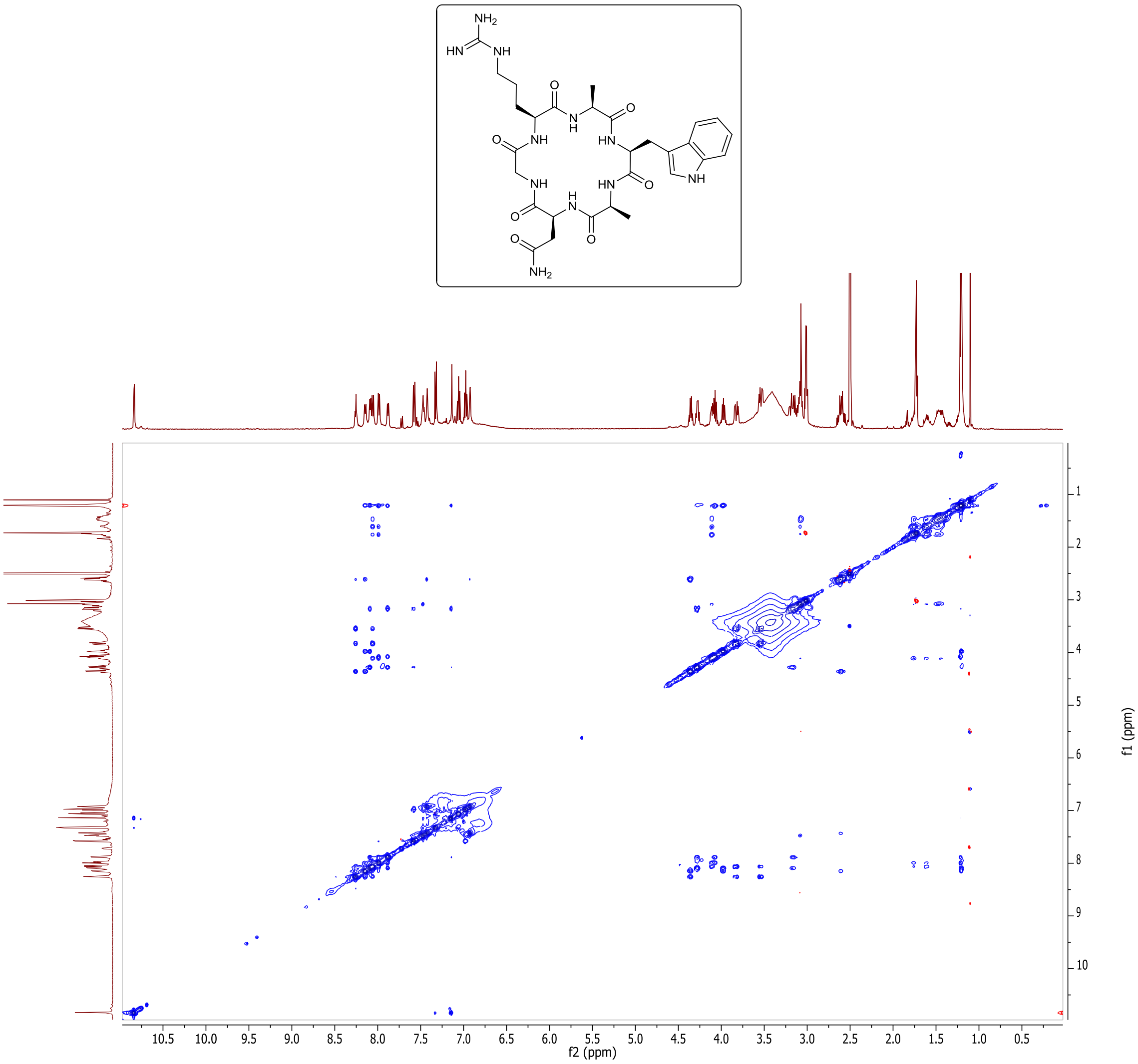


**Supplementary Figure 100 | NOESY NMR spectrum of compound Cyclo(-Arg-Ala-Trp-Ala-Asn-Gly-) (3).**

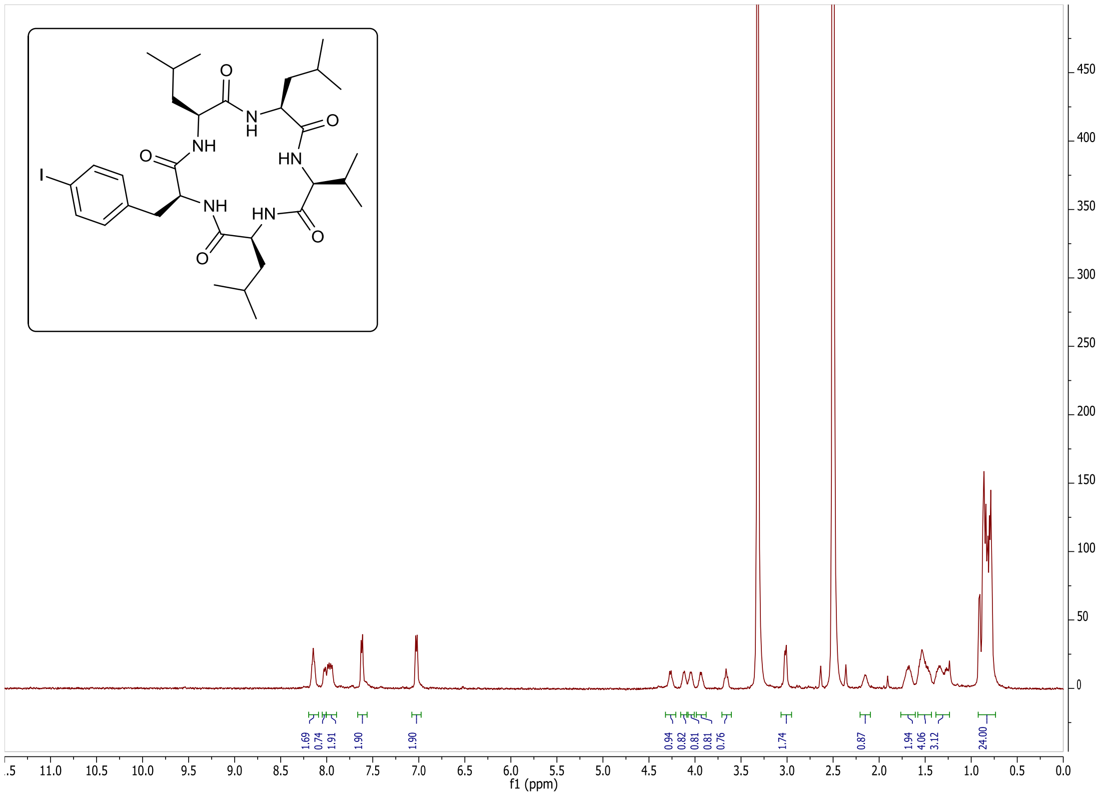


**Supplementary Figure 101 | $^{1}$H NMR spectrum of compound Cyclo(-Leu-Leu-Val-Leu-*p*-I-Phe-) (4).**

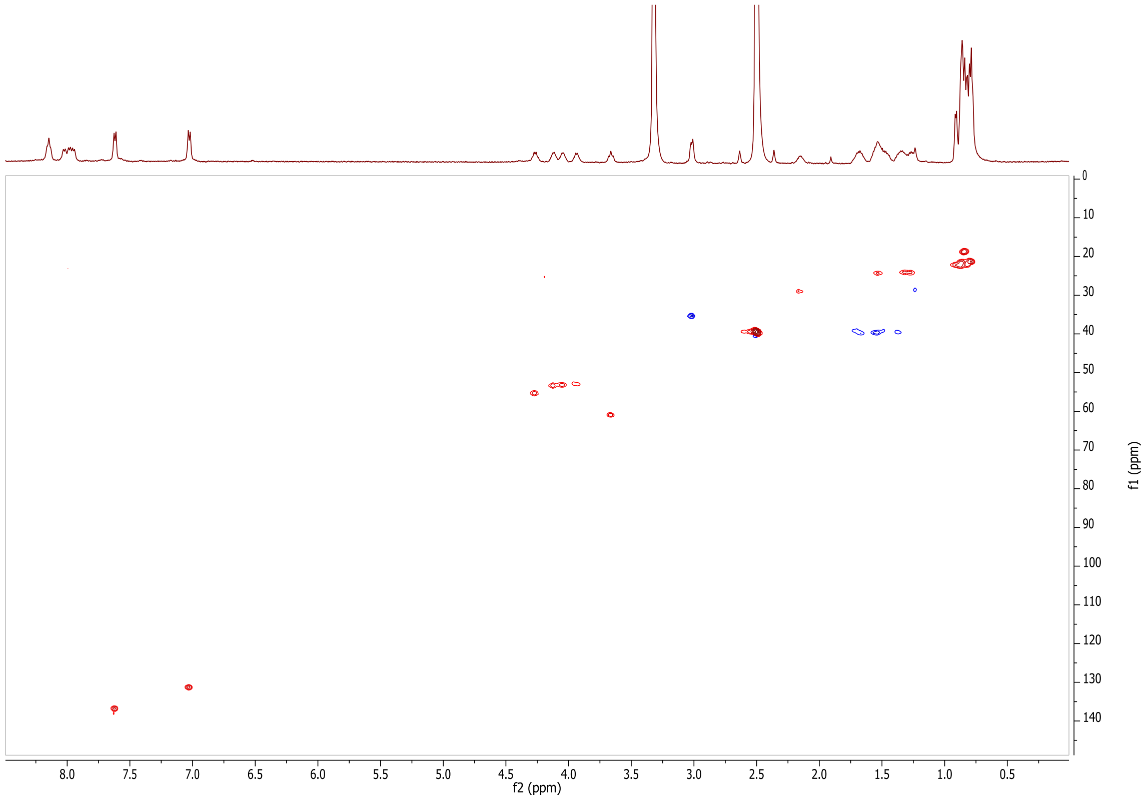


**Supplementary Figure 102 | $^{1}$H-$^{13}$C HSQC NMR spectrum of compound Cyclo(-Leu-Leu-Val-Leu-*p*-I-Phe-) (4).**

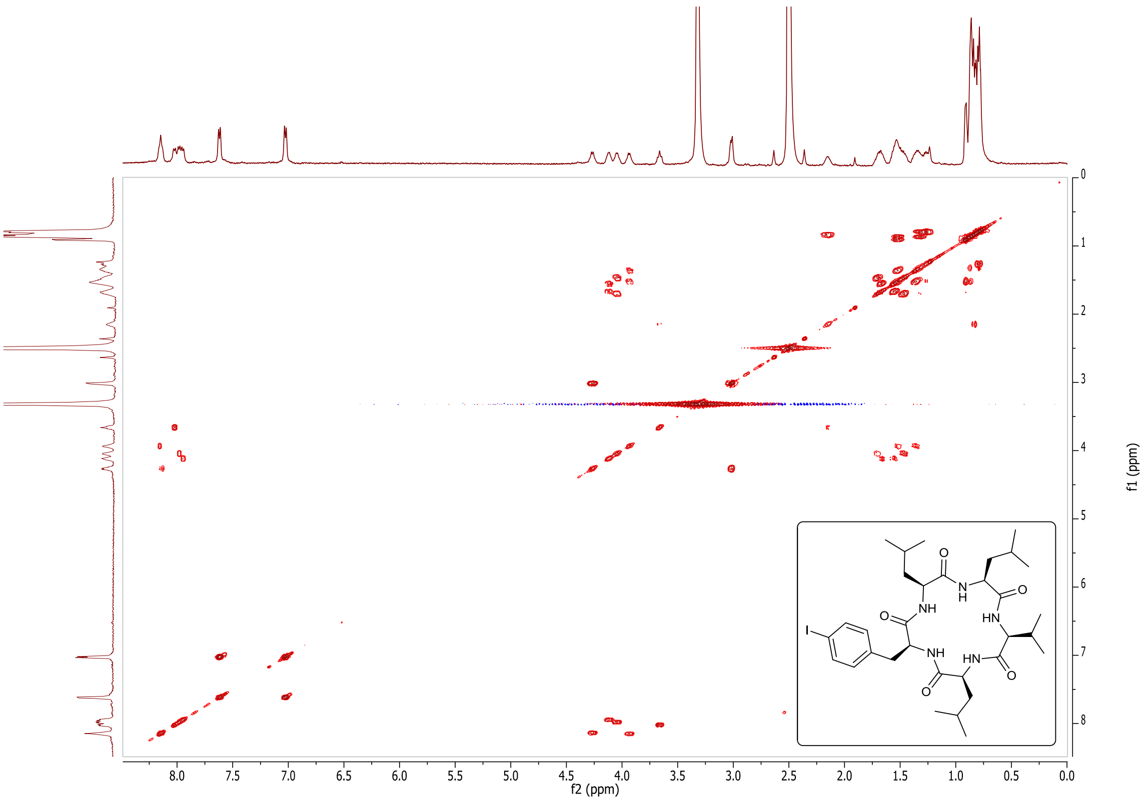


**Supplementary Figure 103 | COSY NMR spectrum of compound Cyclo(-Leu-Leu-Val-Leu-*p*-I-Phe-) (4).**

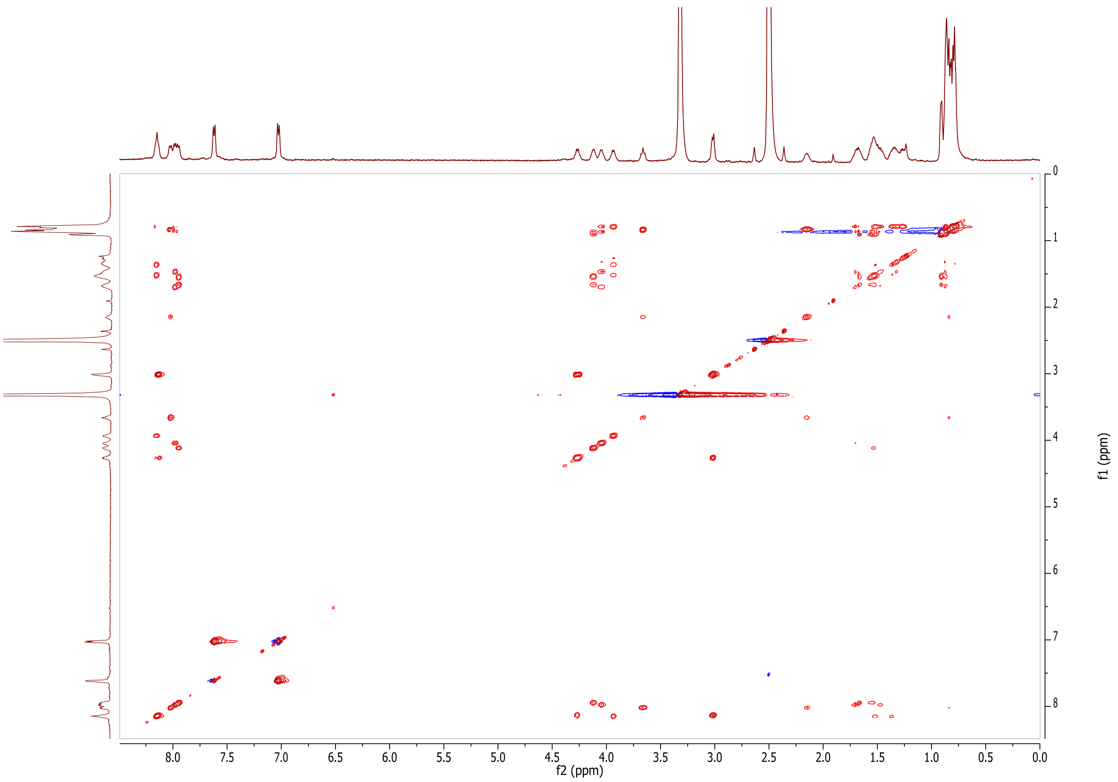


**Supplementary Figure 104 | TOCSY NMR spectrum of compound Cyclo(-Leu-Leu-Val-Leu-*p*-I-Phe-) (4).**

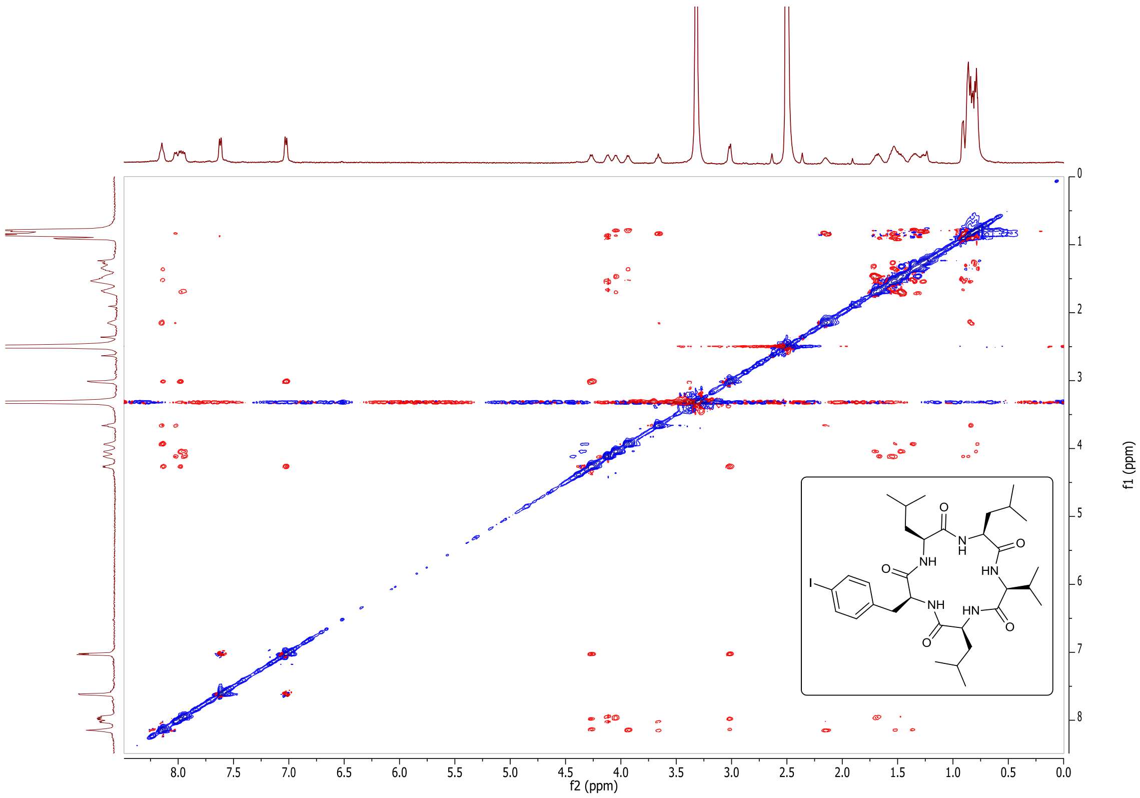


**Supplementary Figure 105 | ROESY NMR spectrum of compound Cyclo(-Leu-Leu-Val-Leu-*p*-I-Phe-) (4).**

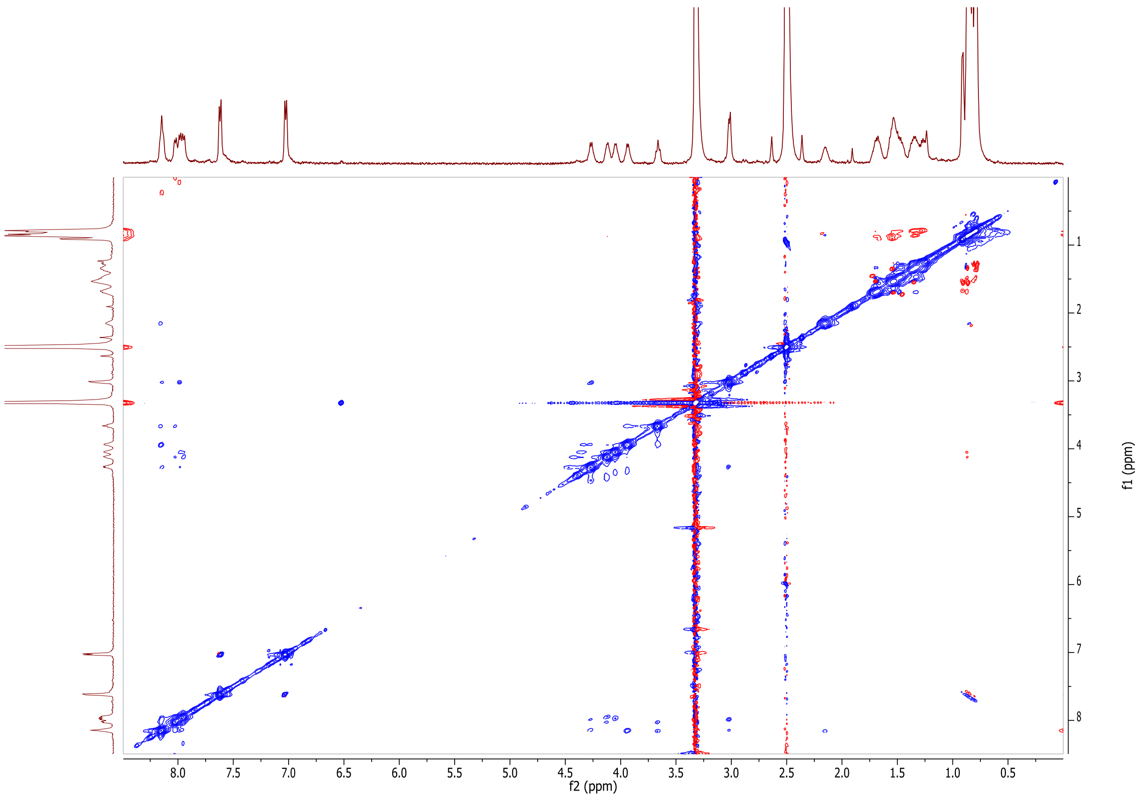


**Supplementary Figure 106 | NOESY NMR spectrum of compound Cyclo(-Leu-Leu-Val-Leu-*p*-I-Phe-) (4).**

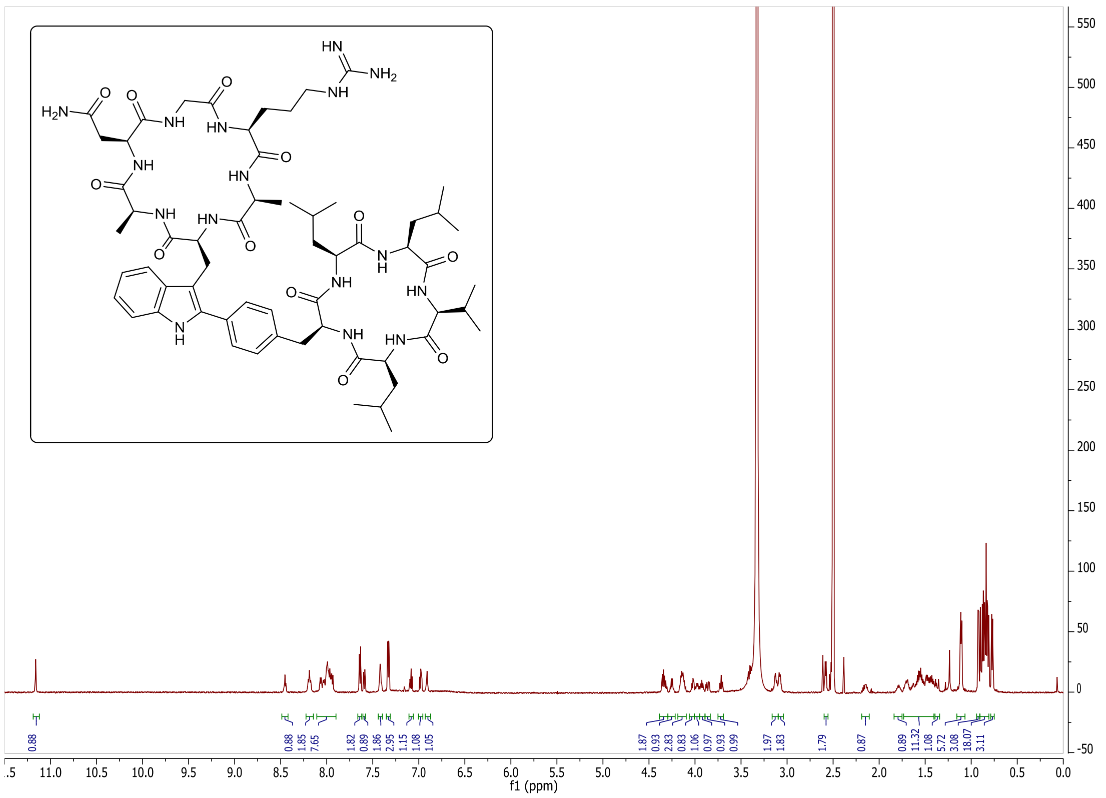

**Supplementary Figure 107 | $^{1}$H NMR spectrum of compound Cyclo(Ala-Asn-Gly-Arg-Ala-C2-Trp-)—Cyclo(C4-Phe-Leu-Leu-Val-Leu-) (5).**

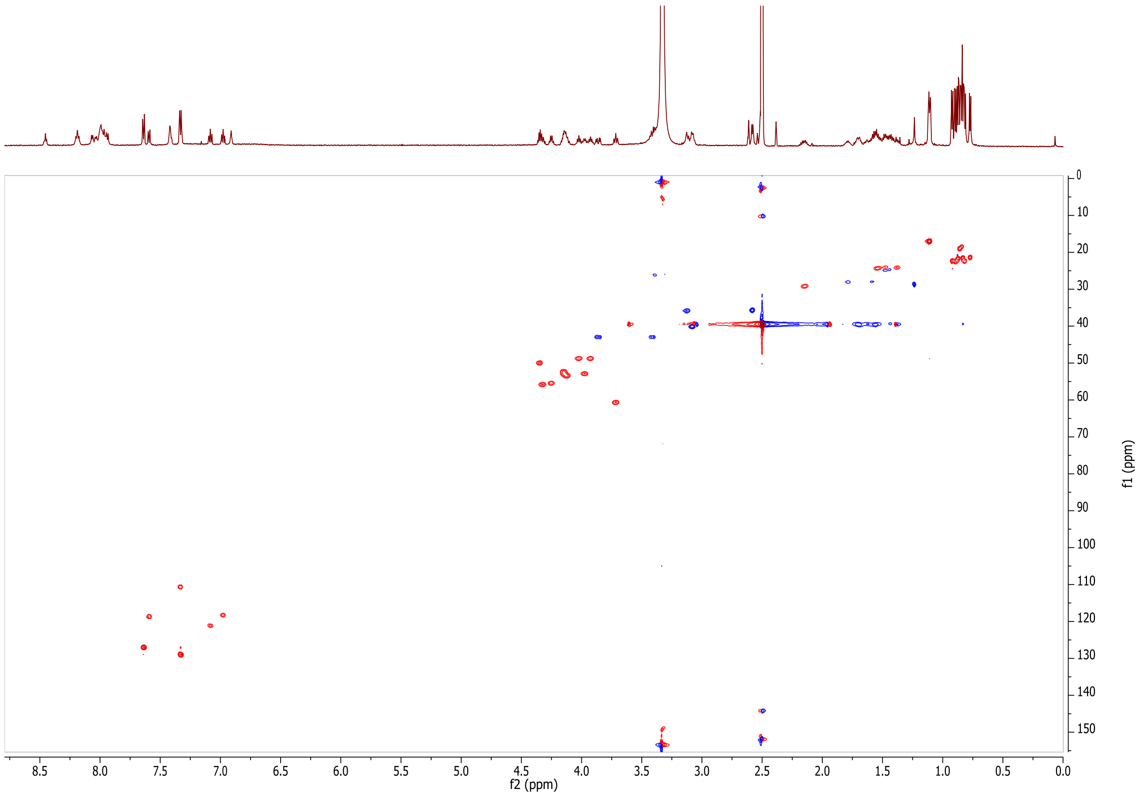

**Supplementary Figure 108 | $^{1}$H-$^{13}$C HSQC NMR spectrum of compound Cyclo(Ala-Asn-Gly-Arg-Ala-C2-Trp-)—Cyclo(C4-Phe-Leu-Leu-Val-Leu-) (5).**

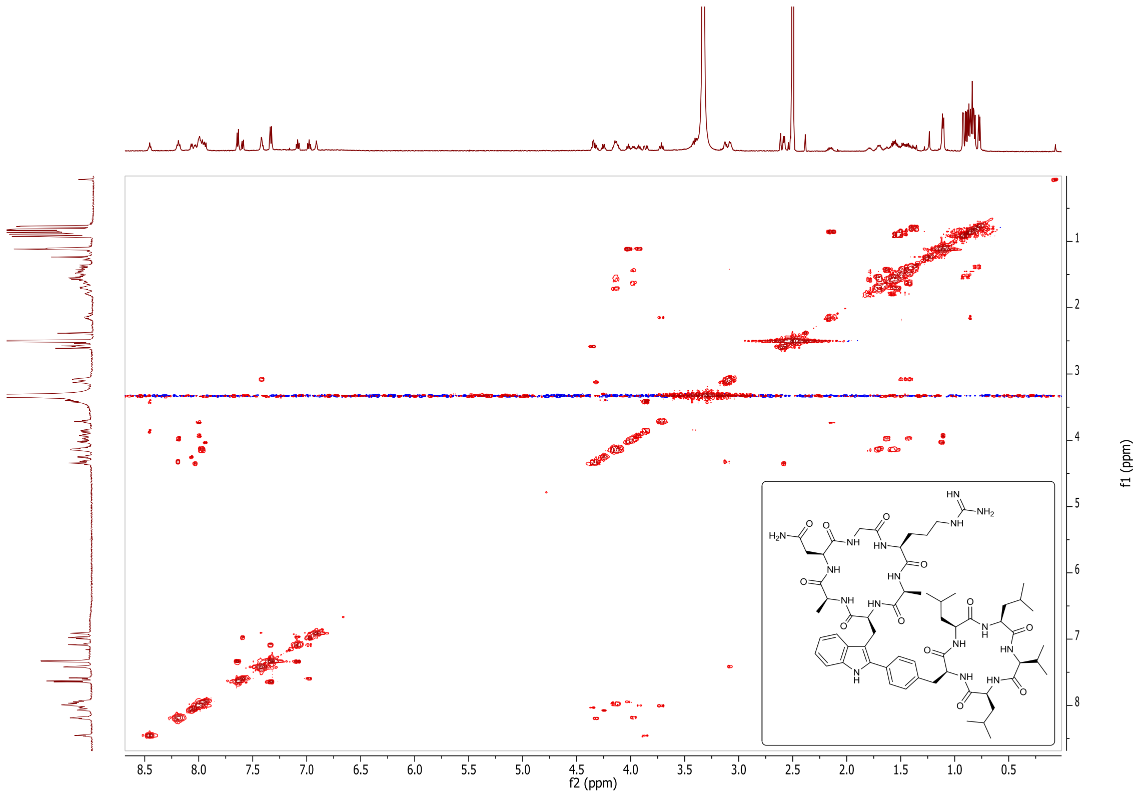


**Supplementary Figure 109 | COSY NMR spectrum of compound Cyclo(Ala-Asn-Gly-Arg-Ala-C2-Trp-)—Cyclo(C4-Phe-Leu-Leu-Val-Leu-) (5).**

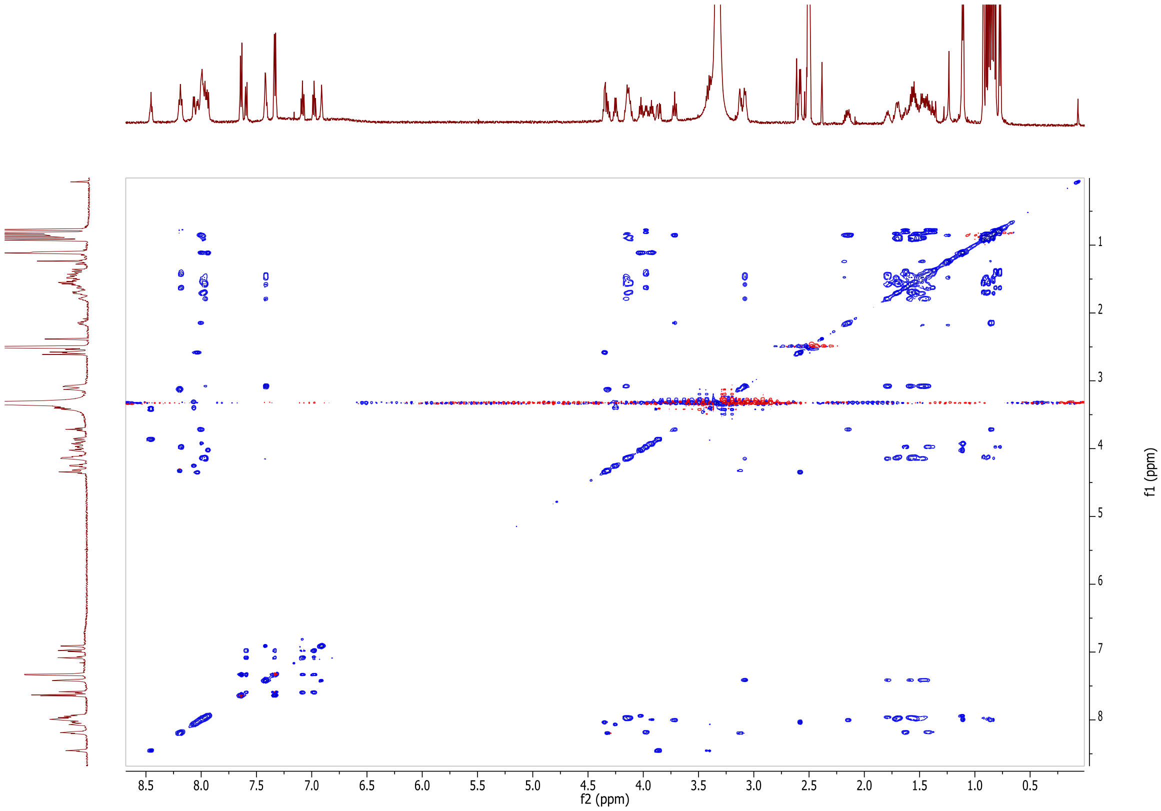


**Supplementary Figure 110 | TOCSY NMR spectrum of compound Cyclo(Ala-Asn-Gly-Arg-Ala-C2-Trp-)—Cyclo(C4-Phe-Leu-Leu-Val-Leu-) (5).**

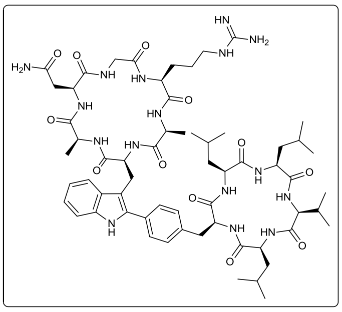

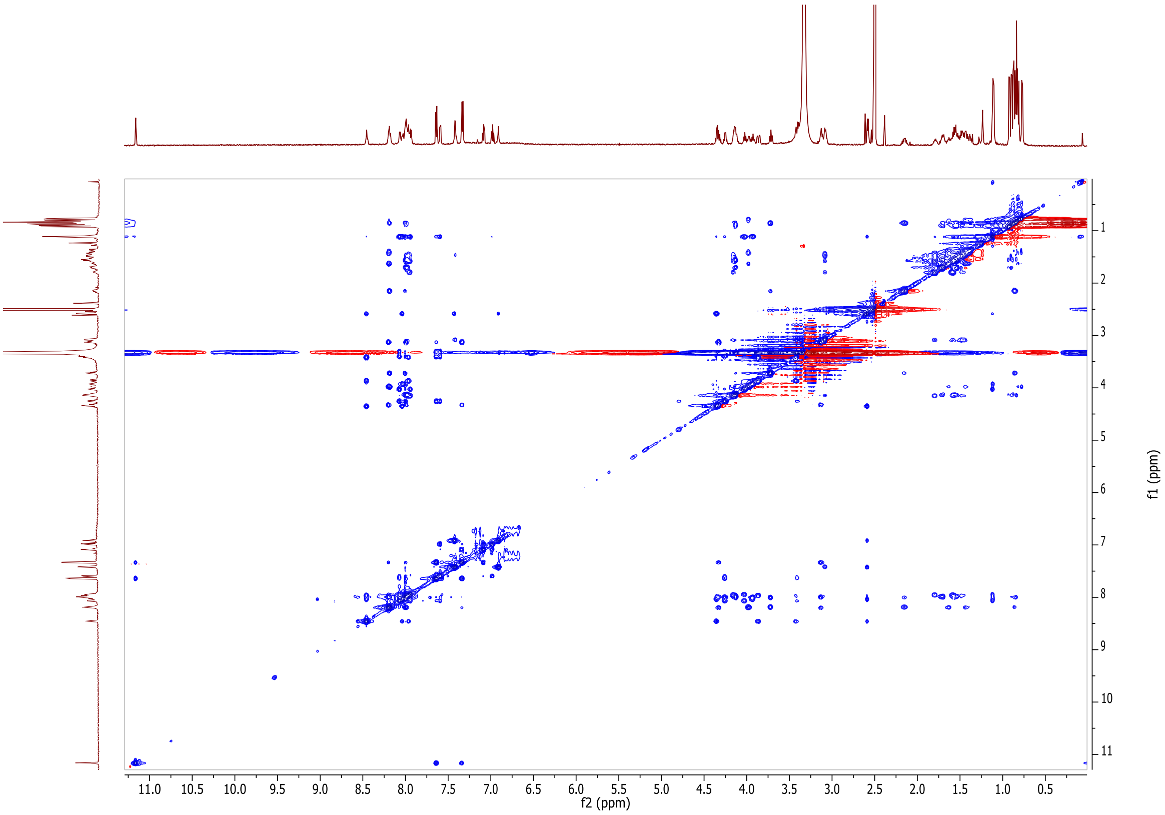


**Supplementary Figure 111 | NOESY NMR spectrum of compound Cyclo(Ala-Asn-Gly-Arg-Ala-C2-Trp-)—Cyclo(C4-Phe-Leu-Leu-Val-Leu-) (5).**

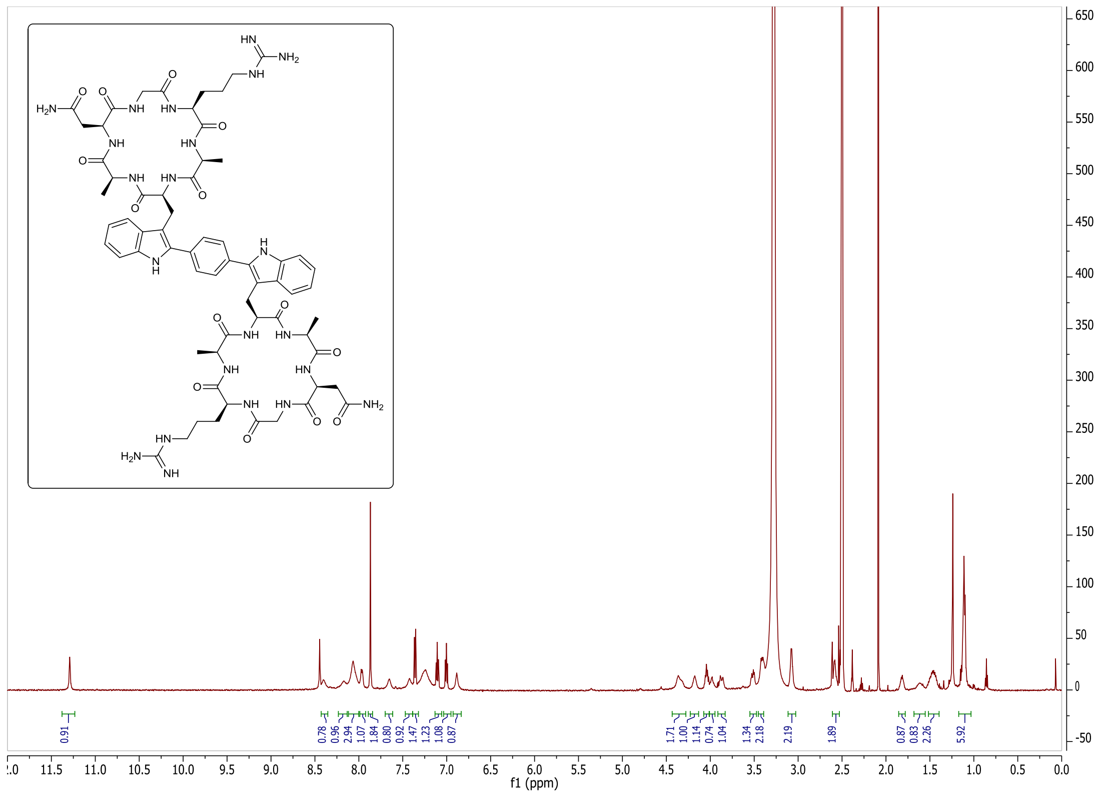

**Supplementary Figure 112 | $^1$H NMR spectrum of compound Bis[cyclo(-Arg-Ala-Trp-Ala-Asn-Gly-)] adduct (6).**

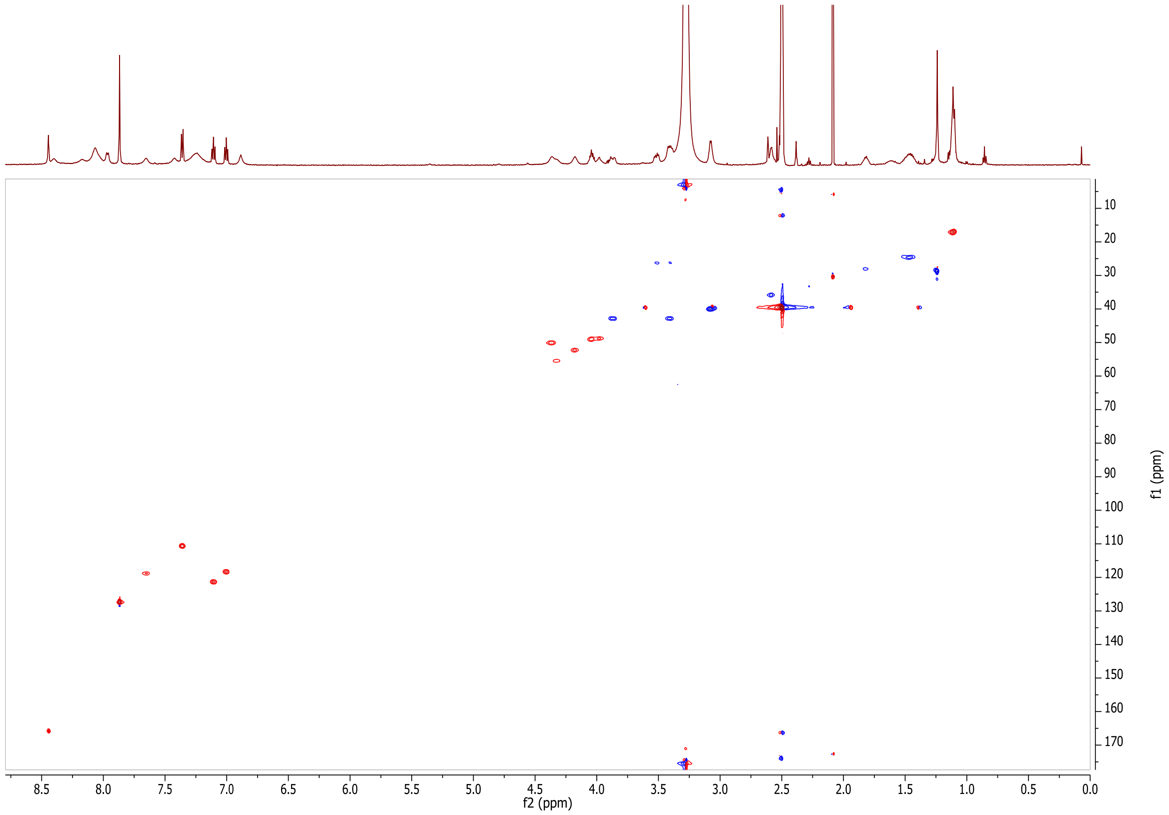

**Supplementary Figure 113 | $^1$H-$^{13}$C HSQC NMR spectrum of compound Bis[cyclo(-Arg-Ala-Trp-Ala-Asn-Gly-)] adduct (6).**

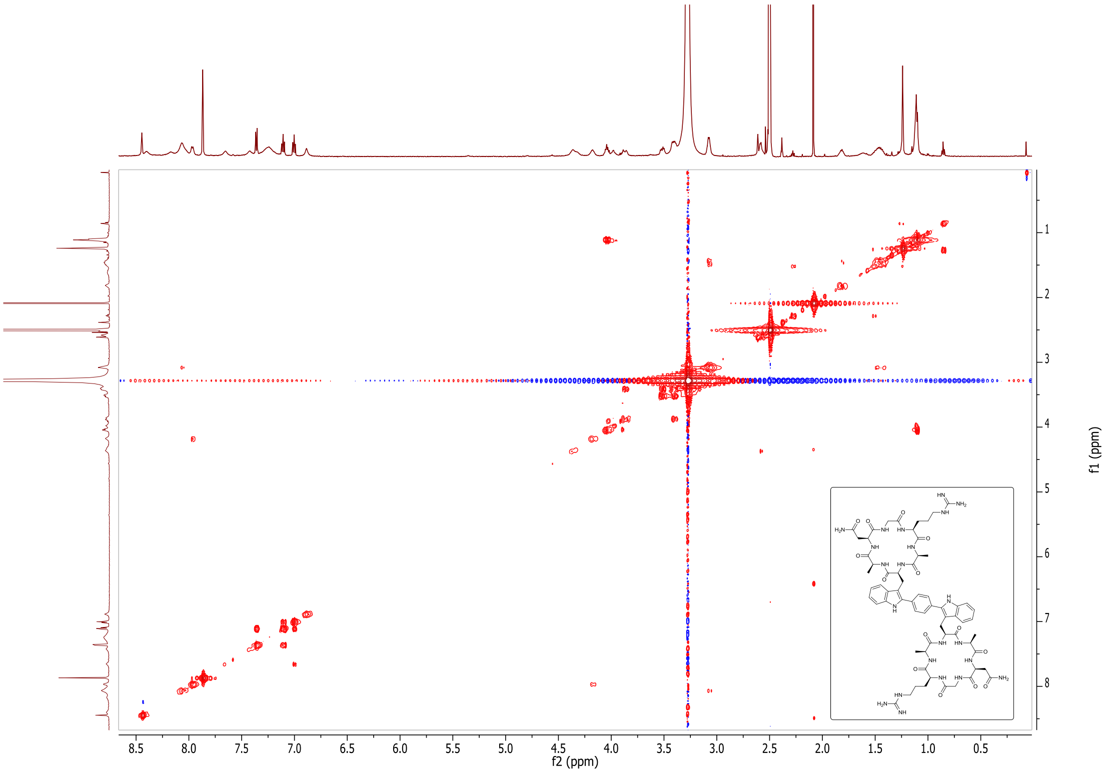


**Supplementary Figure 114 | COSY NMR spectrum of compound Bis[cyclo(-Arg-Ala-Trp-Ala-Asn-Gly-)] adduct (6).**

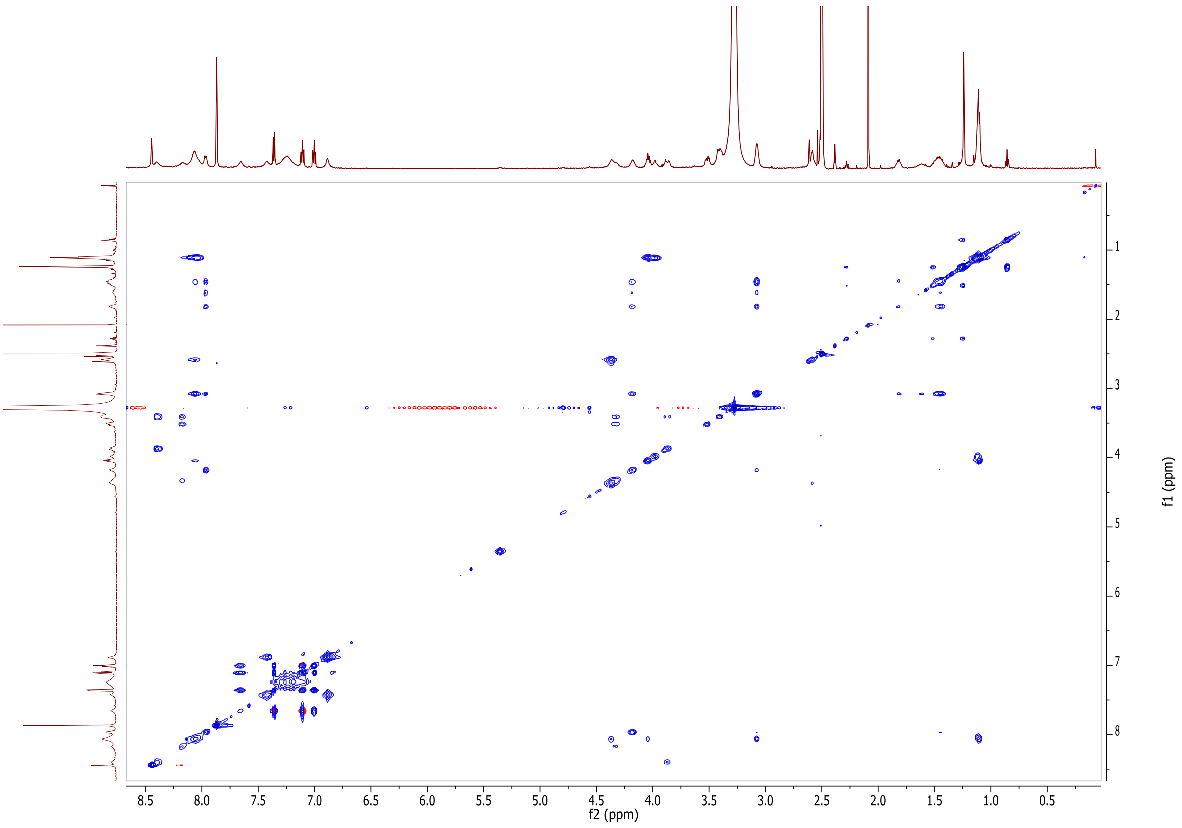


**Supplementary Figure 115 | TOCSY NMR spectrum of compound Bis[cyclo(-Arg-Ala-Trp-Ala-Asn-Gly-)] adduct (6).**

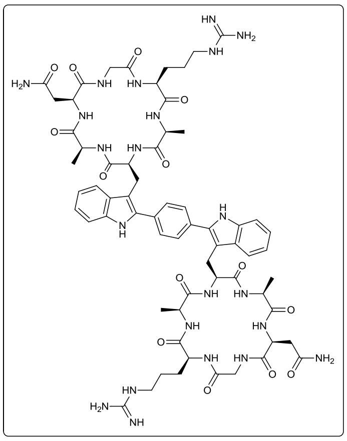

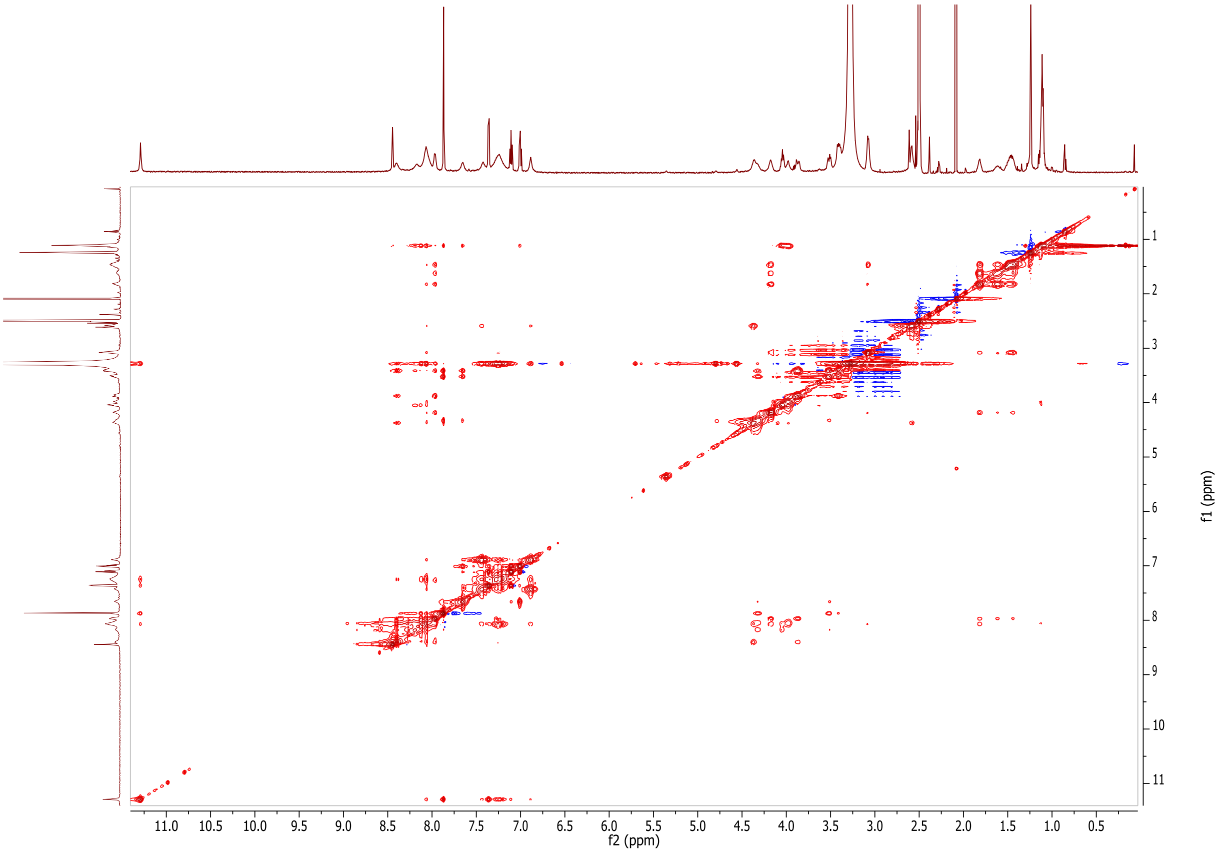


**Supplementary Figure 116 | NOESY NMR spectrum of compound Bis[cyclo(-Arg-Ala-Trp-Ala-Asn-Gly-)] adduct (6).**

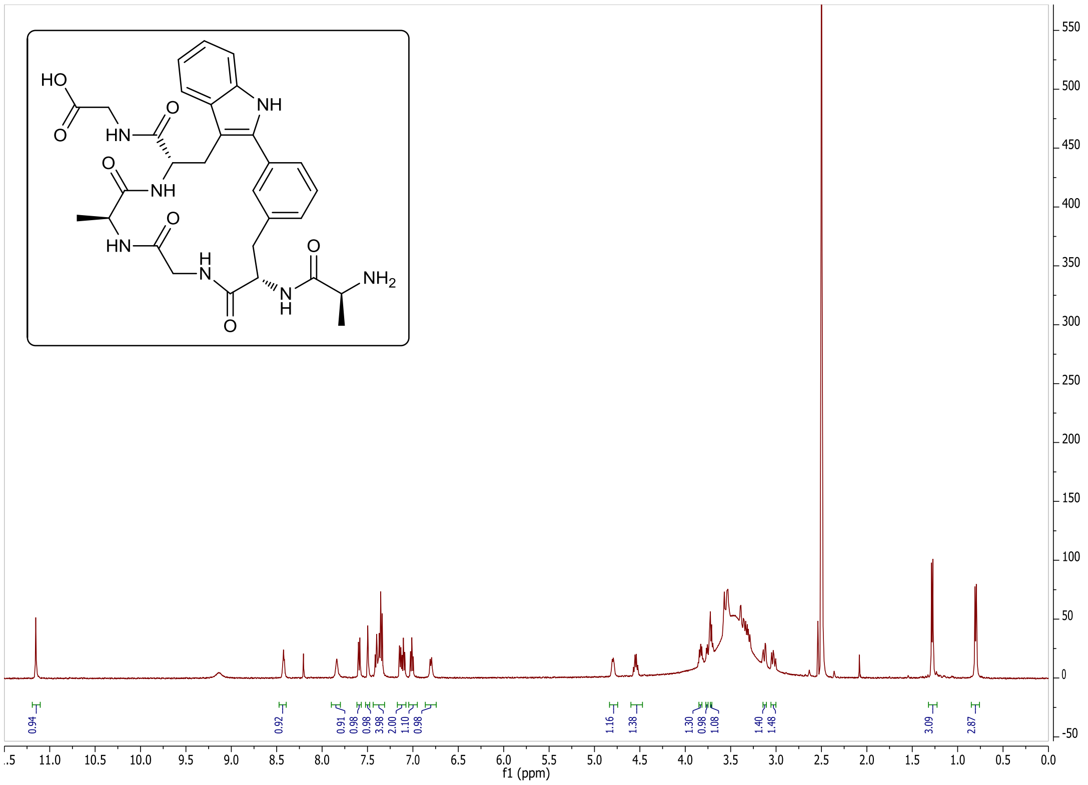

**Supplementary Figure 117 | $^{1}$H NMR spectrum of compound H-Ala-(Cyclo-*m*)-[Phe-Gly-Ala-Trp]-Gly-OH (9).**

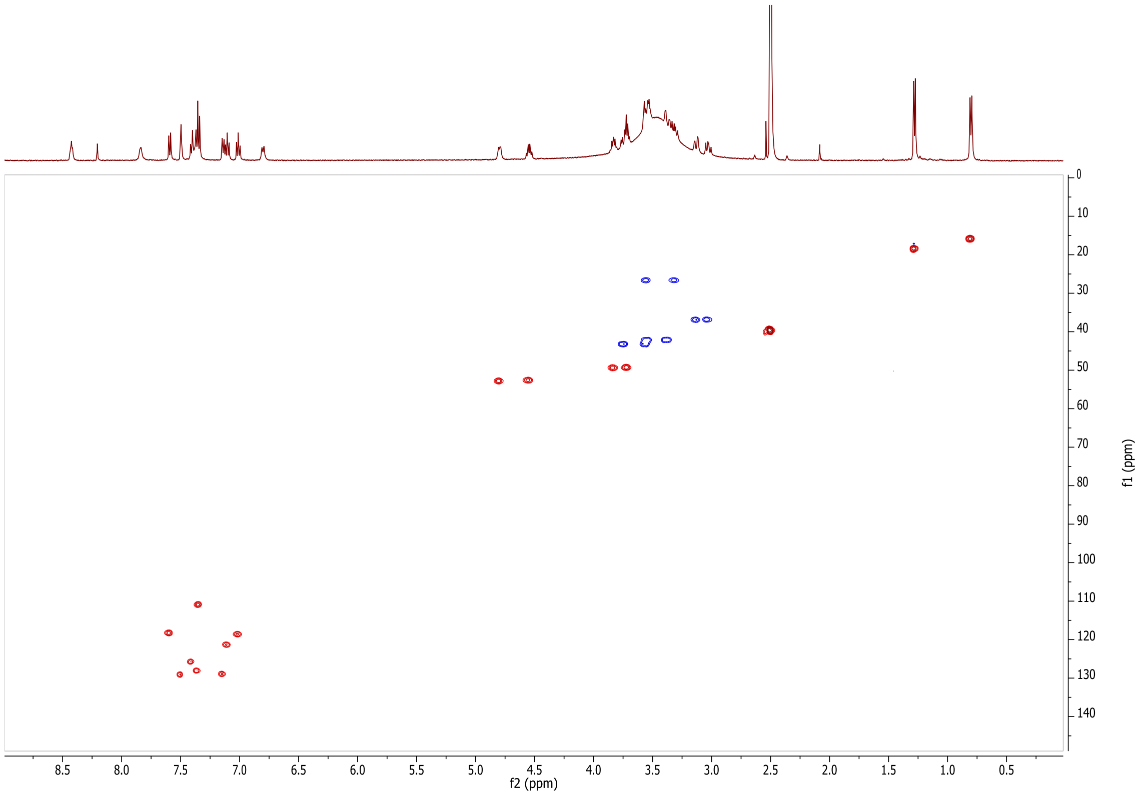

**Supplementary Figure 118 | $^{1}$H-$^{13}$C HSQC NMR spectrum of compound H-Ala-(Cyclo-*m*)-[Phe-Gly-Ala-Trp]-Gly-OH (9).**

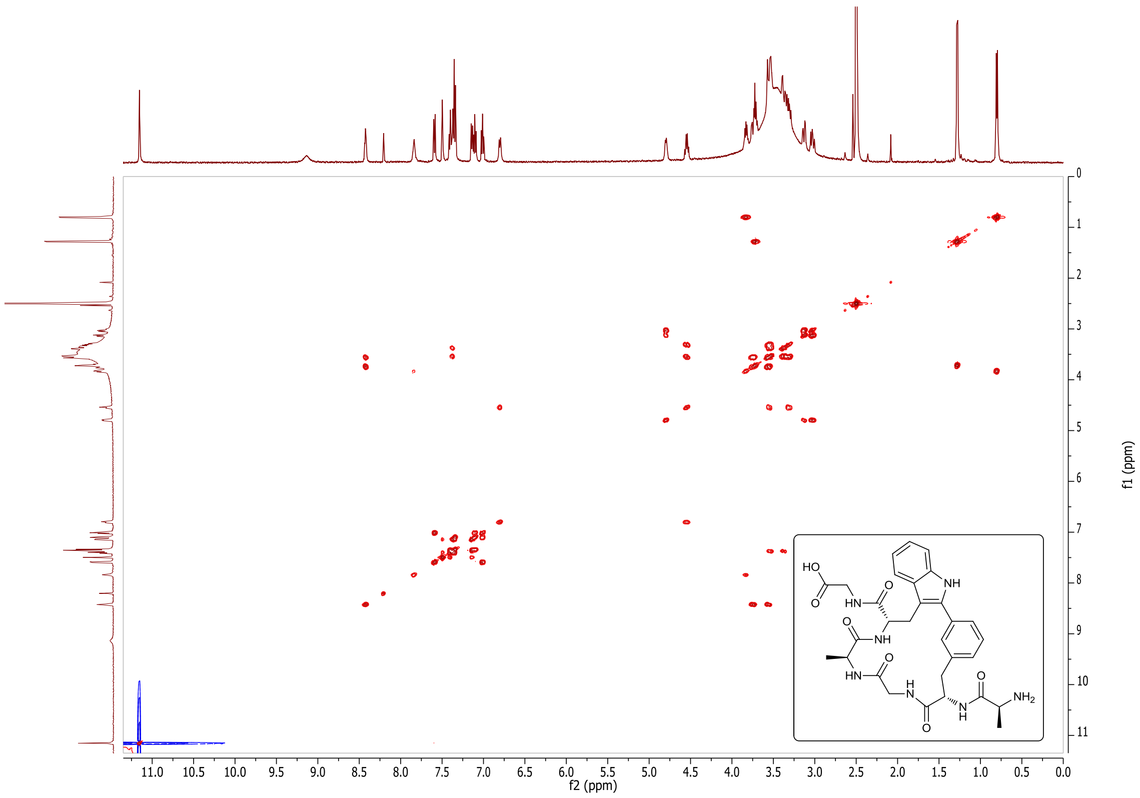


**Supplementary Figure 119 | COSY NMR spectrum of compound H-Ala-(Cyclo-*m*)-[Phe-Gly-Ala-Trp]-Gly-OH (9).**

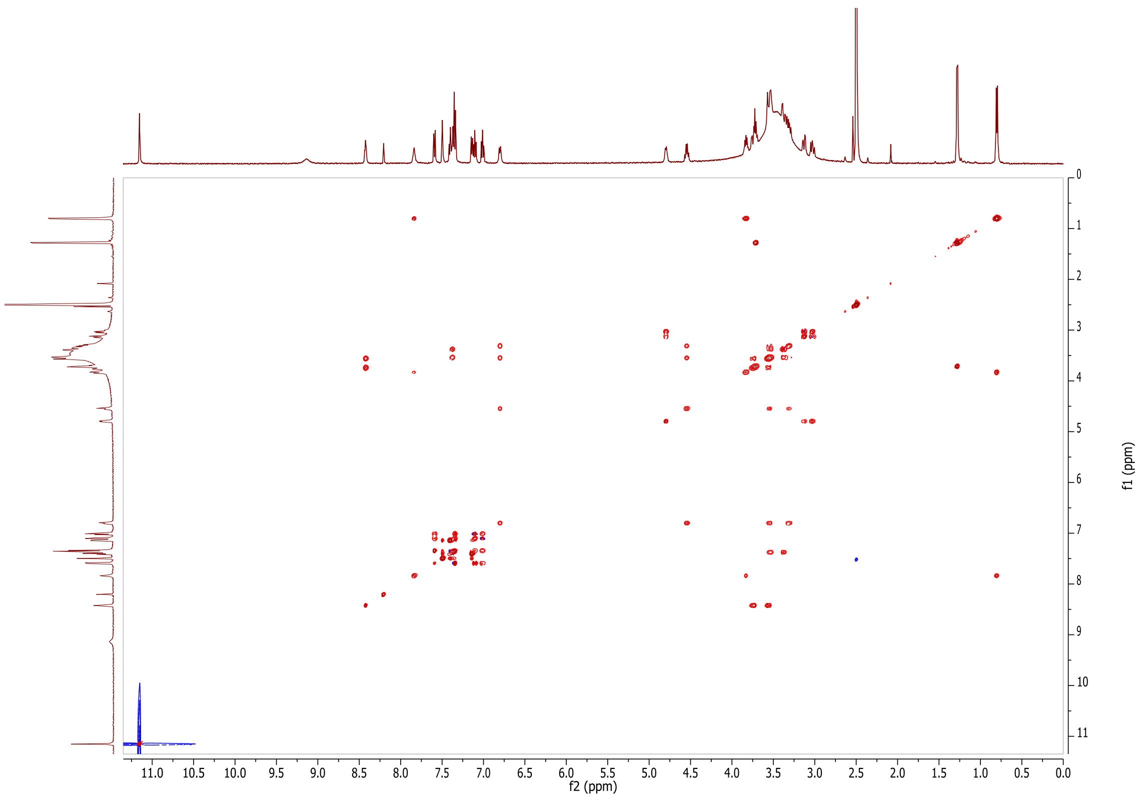


**Supplementary Figure 120 | TOCSY NMR spectrum of compound H-Ala-(Cyclo-*m*)-[Phe-Gly-Ala-Trp]-Gly-OH (9).**

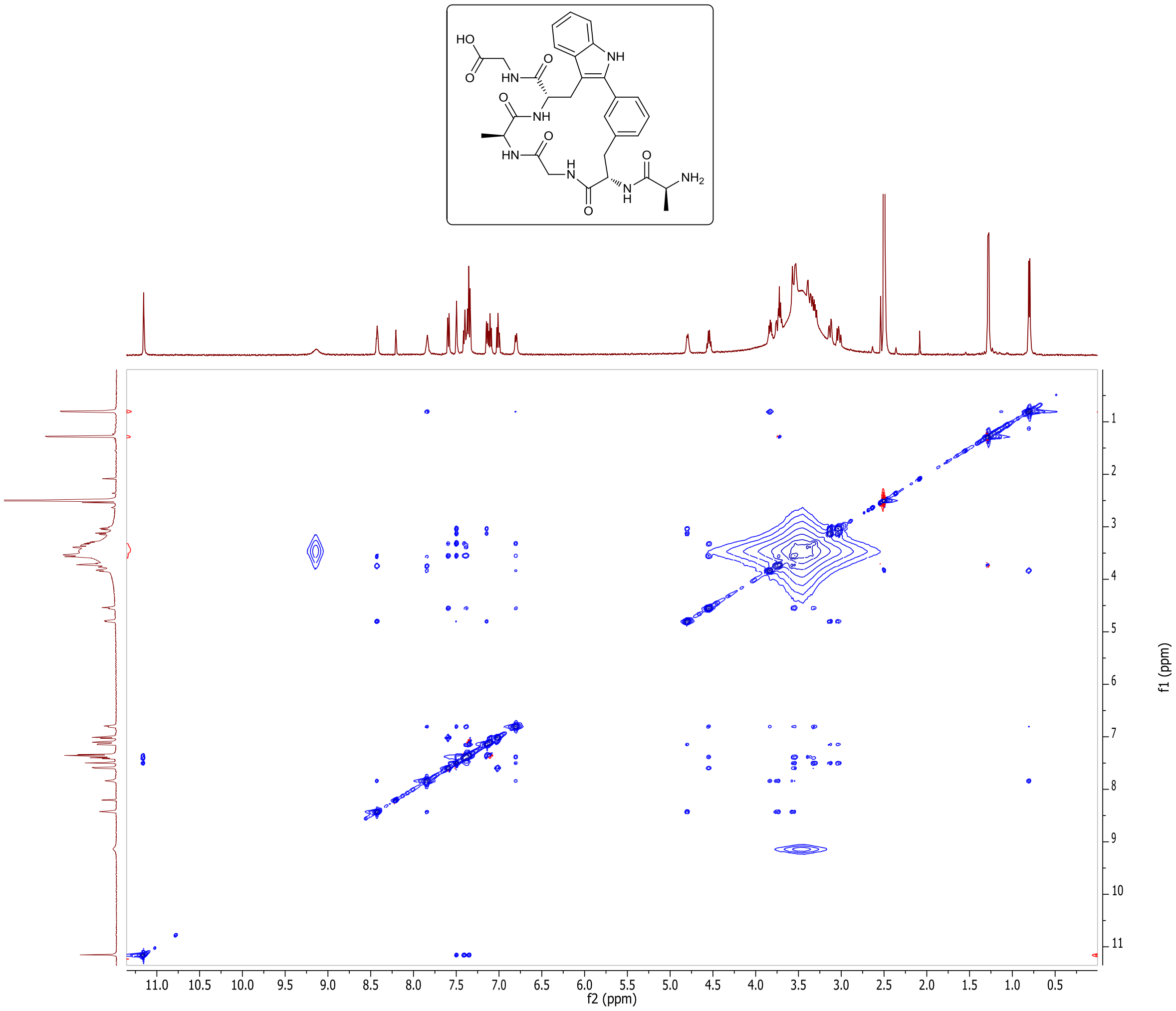


**Supplementary Figure 121 | NOESY NMR spectrum of compound H-Ala-(Cyclo-*m*)-[Phe-Gly-Ala-Trp]-Gly-OH (9).**

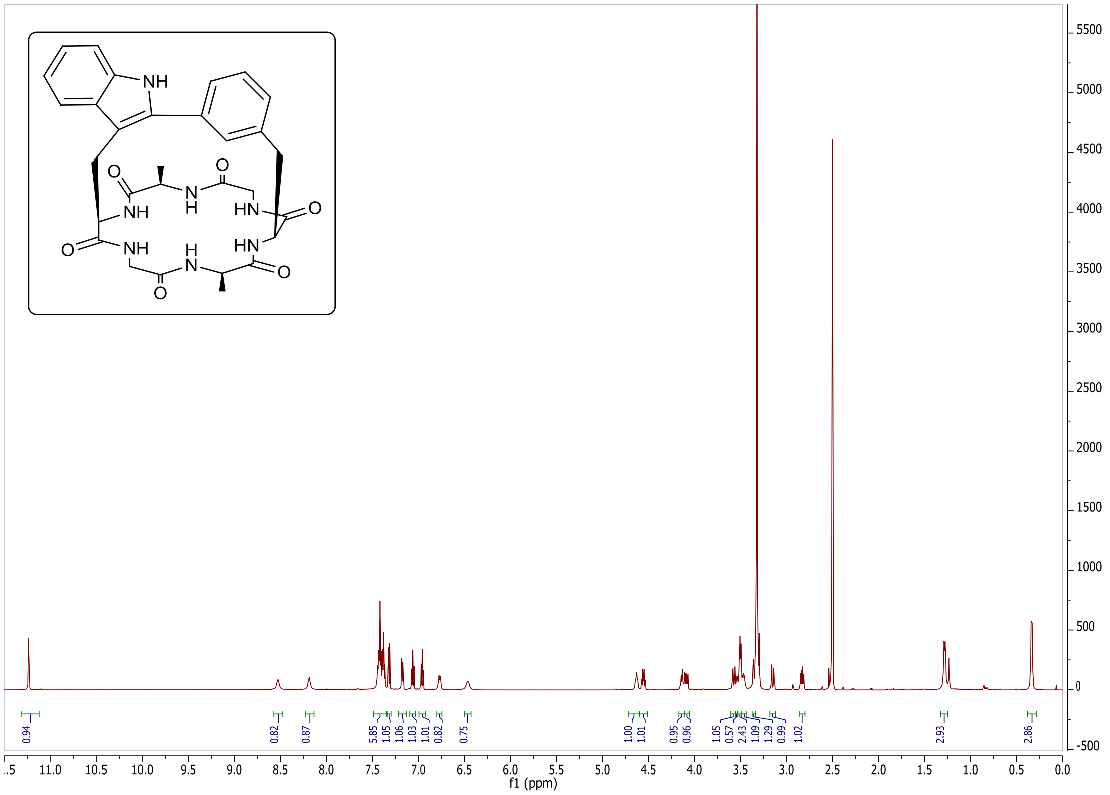


**Supplementary Figure 122 | $^{1}$H NMR spectrum of compound Cyclo[-Ala-(Cyclo-*m*)-[Phe-Gly-Ala-Trp]-Gly-] (10).**

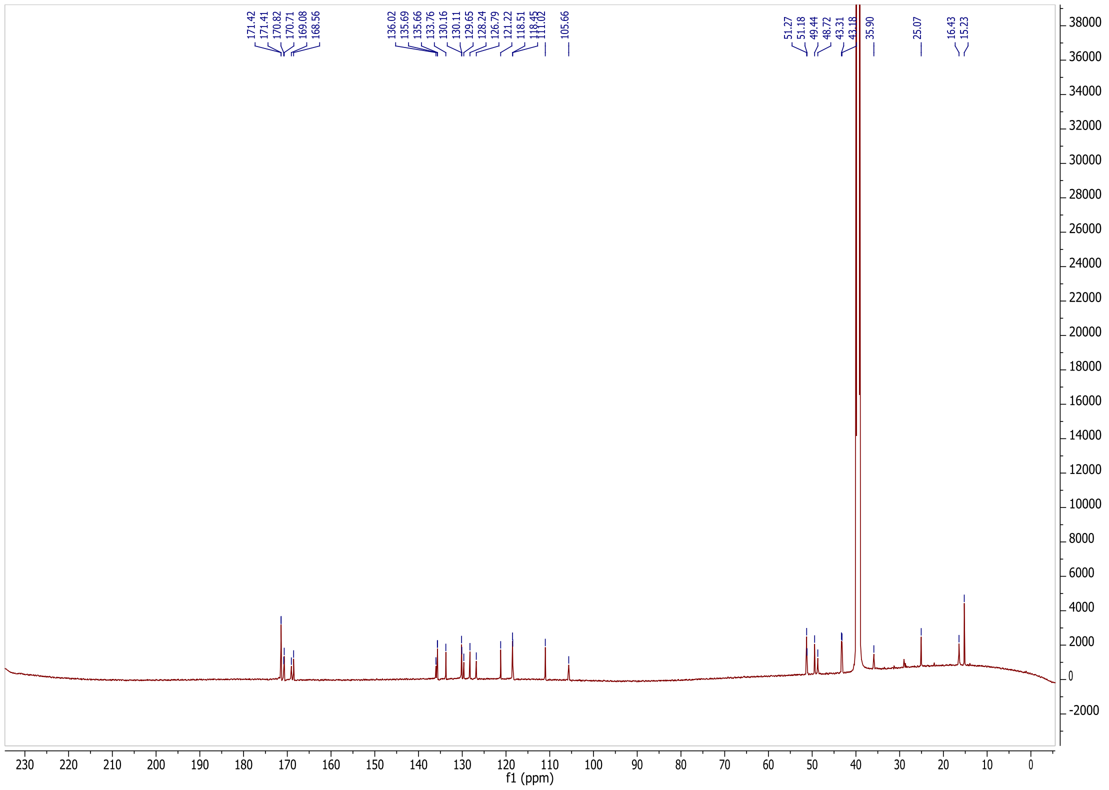


**Supplementary Figure 123 | $^{13}$C NMR spectrum of compound Cyclo[-Ala-(Cyclo-*m*)-[Phe-Gly-Ala-Trp]-Gly-] (10).**

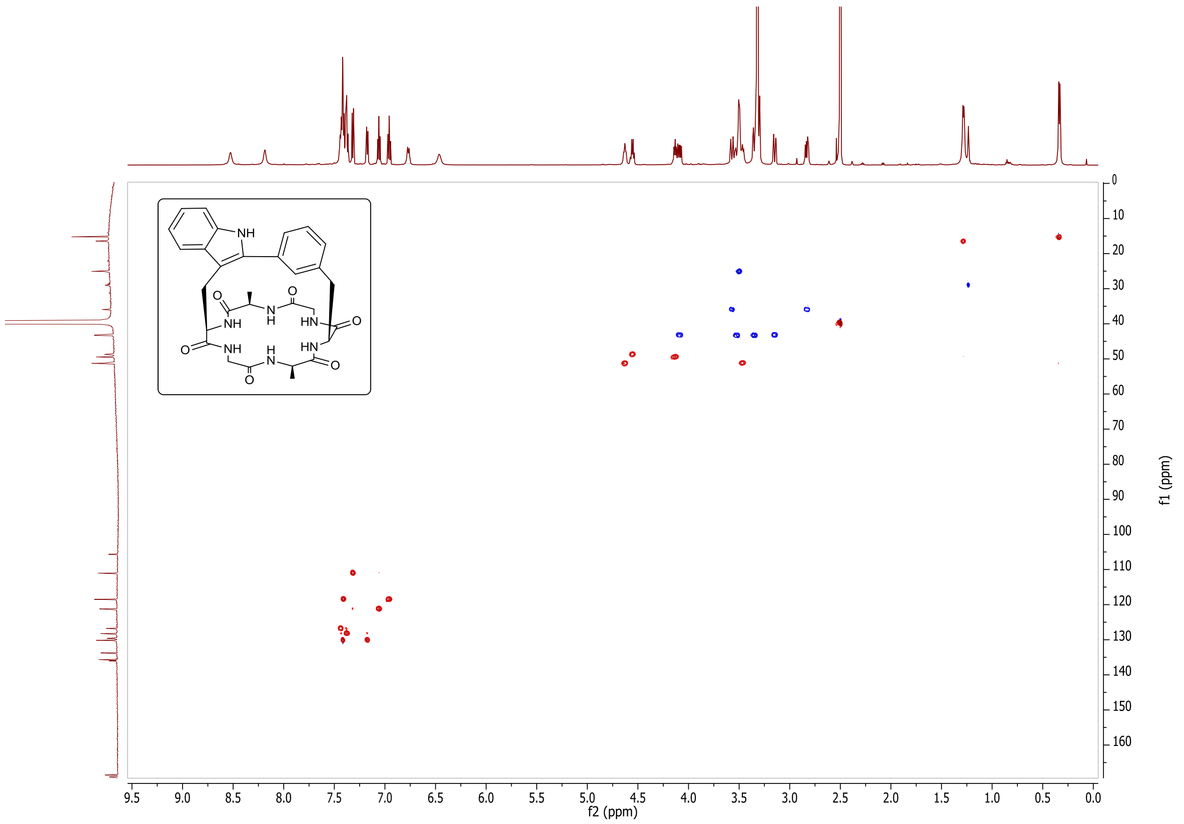


**Supplementary Figure 124 | $^{1}H$-$^{13}C$ HSQC NMR spectrum of compound Cyclo[-Ala-(Cyclo-*m*)-[Phe-Gly-Ala-Trp]-Gly-] (10).**

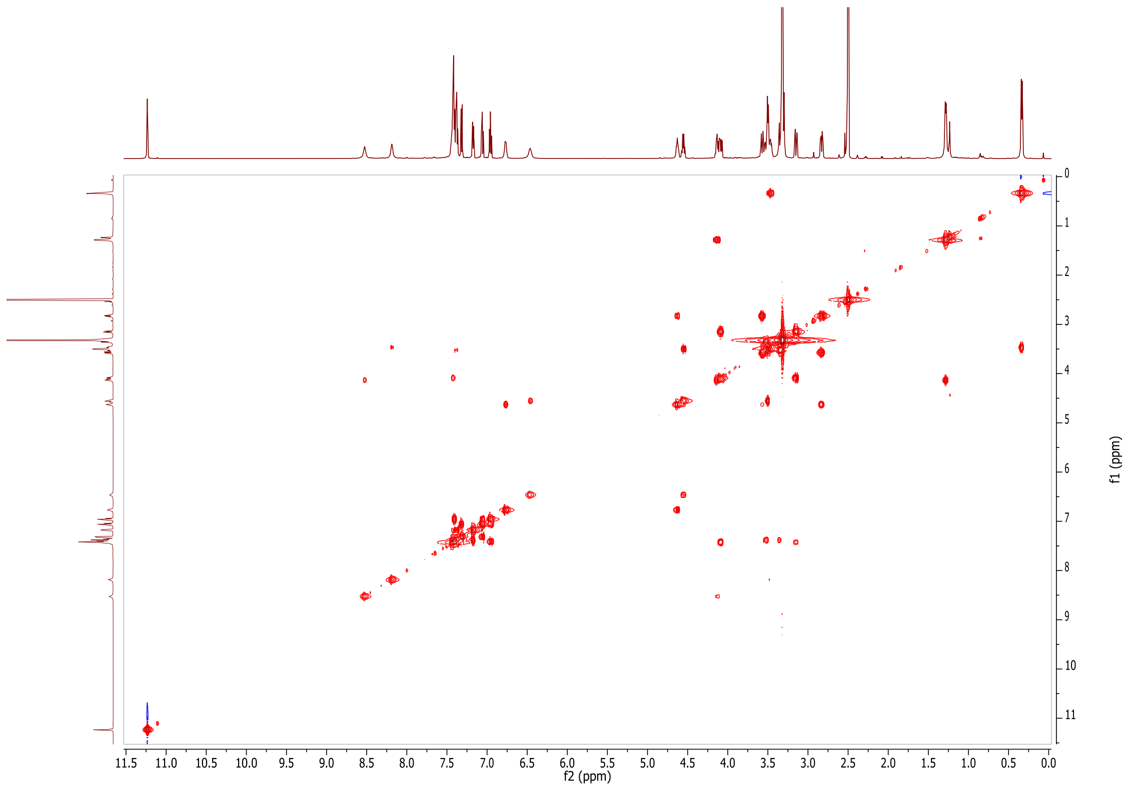


**Supplementary Figure 125 | COSY NMR spectrum of compound Cyclo[-Ala-(Cyclo-*m*)-[Phe-Gly-Ala-Trp]-Gly-] (10).**

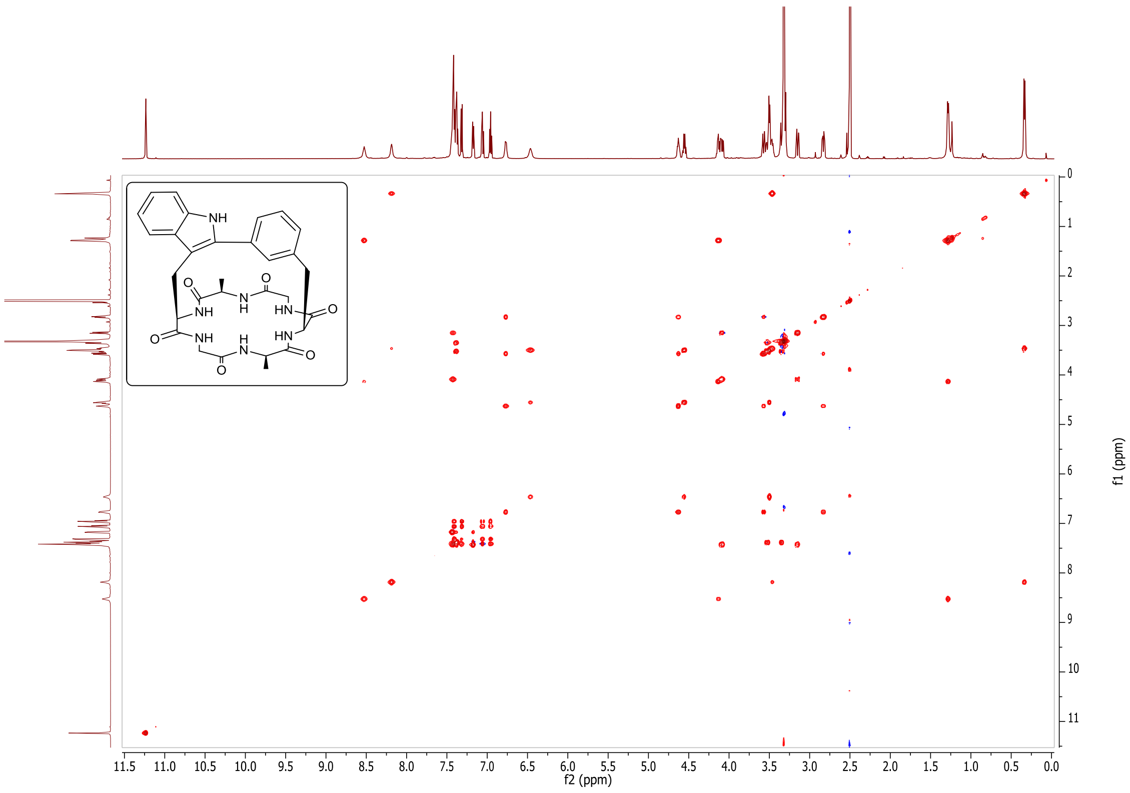


**Supplementary Figure 126 | TOCSY NMR spectrum of compound Cyclo[-Ala-(Cyclo-*m*)-[Phe-Gly-Ala-Trp]-Gly-] (10).**

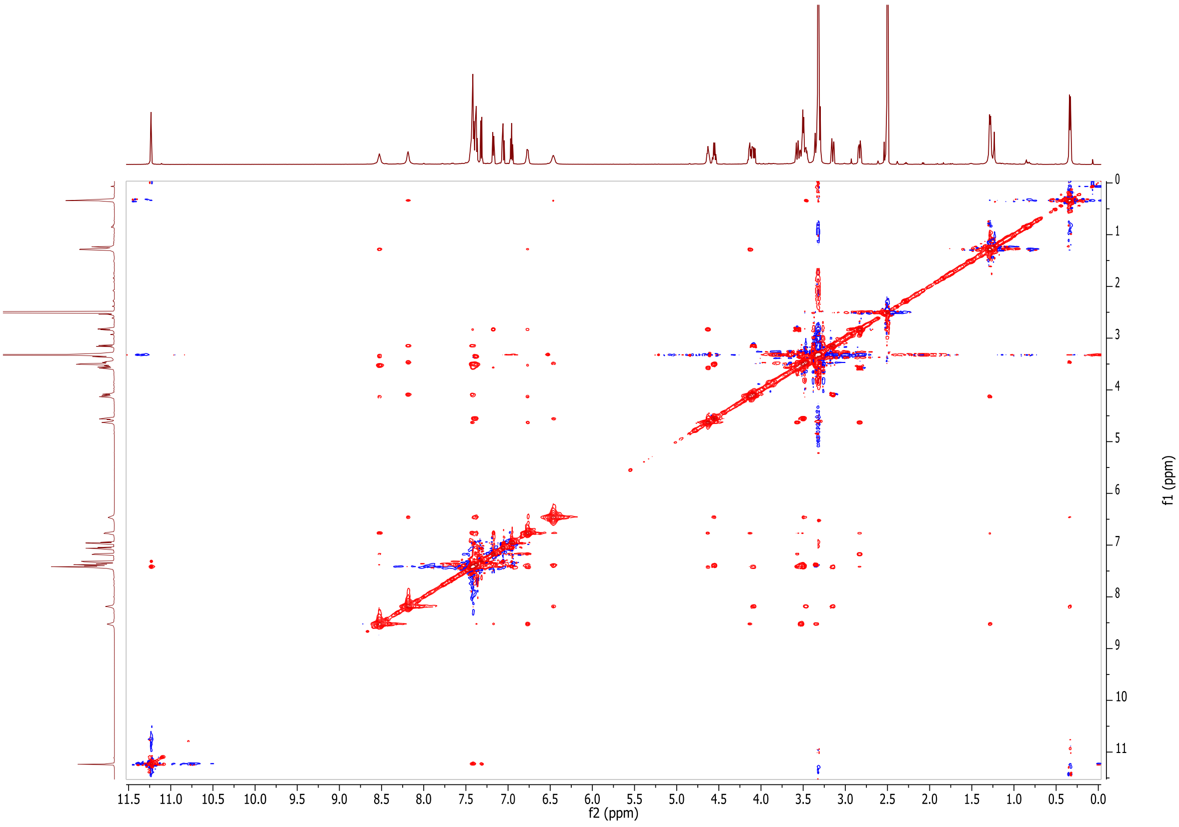


**Supplementary Figure 127 | NOESY NMR spectrum of compound Cyclo[-Ala-(Cyclo-*m*)-[Phe-Gly-Ala-Trp]-Gly-] (10).**

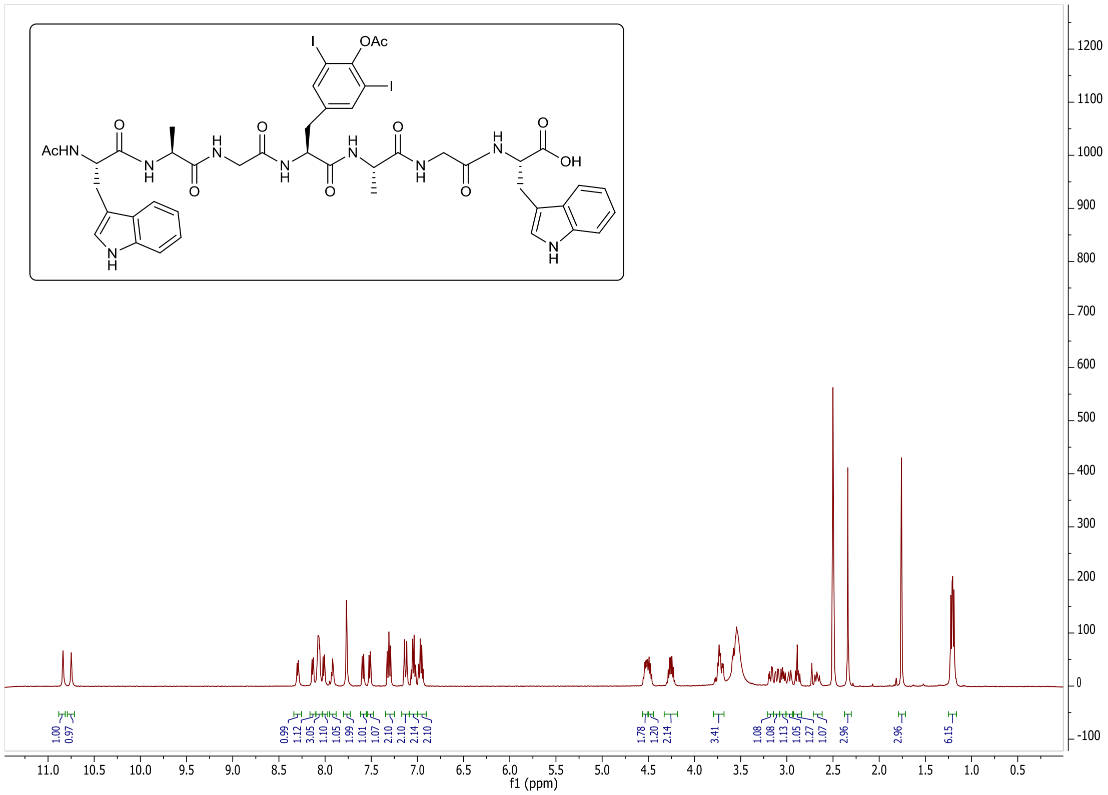


**Supplementary Figure 128** | $^{1}$H NMR spectrum of compound Ac-Trp-Ala-Gly-*3,5*-I,I-Tyr(OAc)-Ala-Gly-Trp-OH (11).

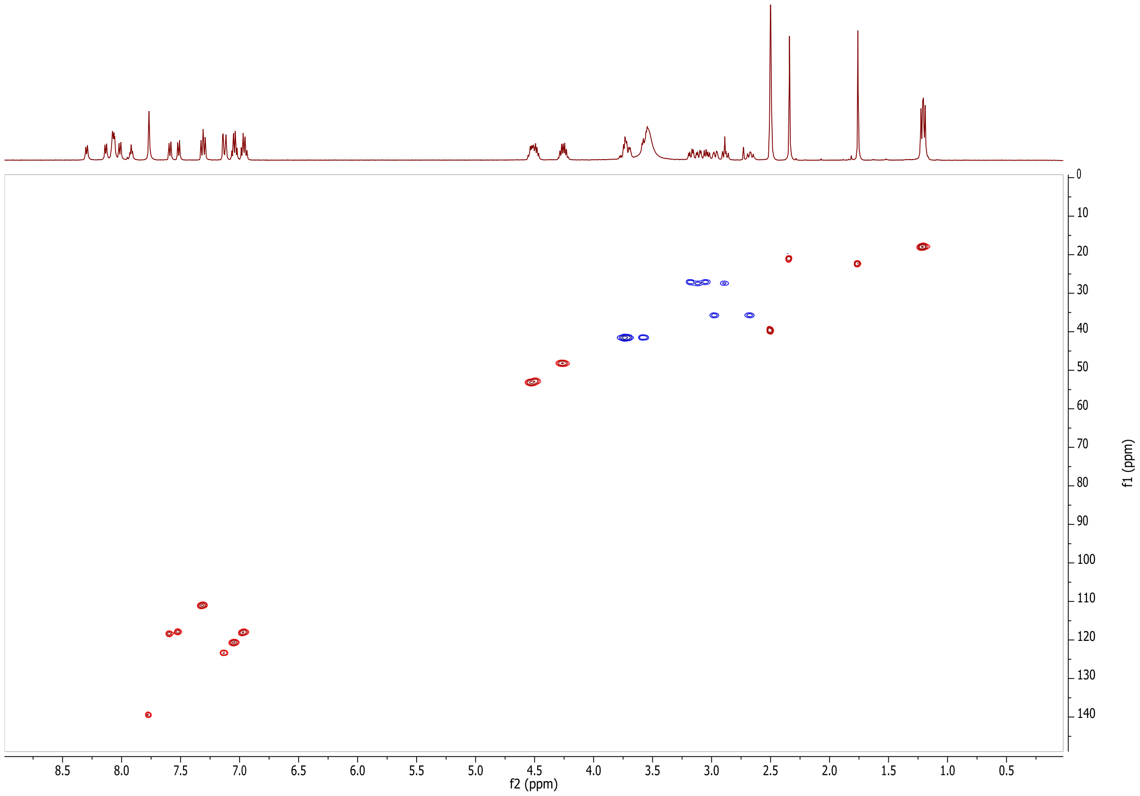


**Supplementary Figure 129** | $^{1}$H-$^{13}$C HSQC NMR spectrum of compound Ac-Trp-Ala-Gly-*3,5*-I,I-Tyr(OAc)-Ala-Gly-Trp-OH (11).

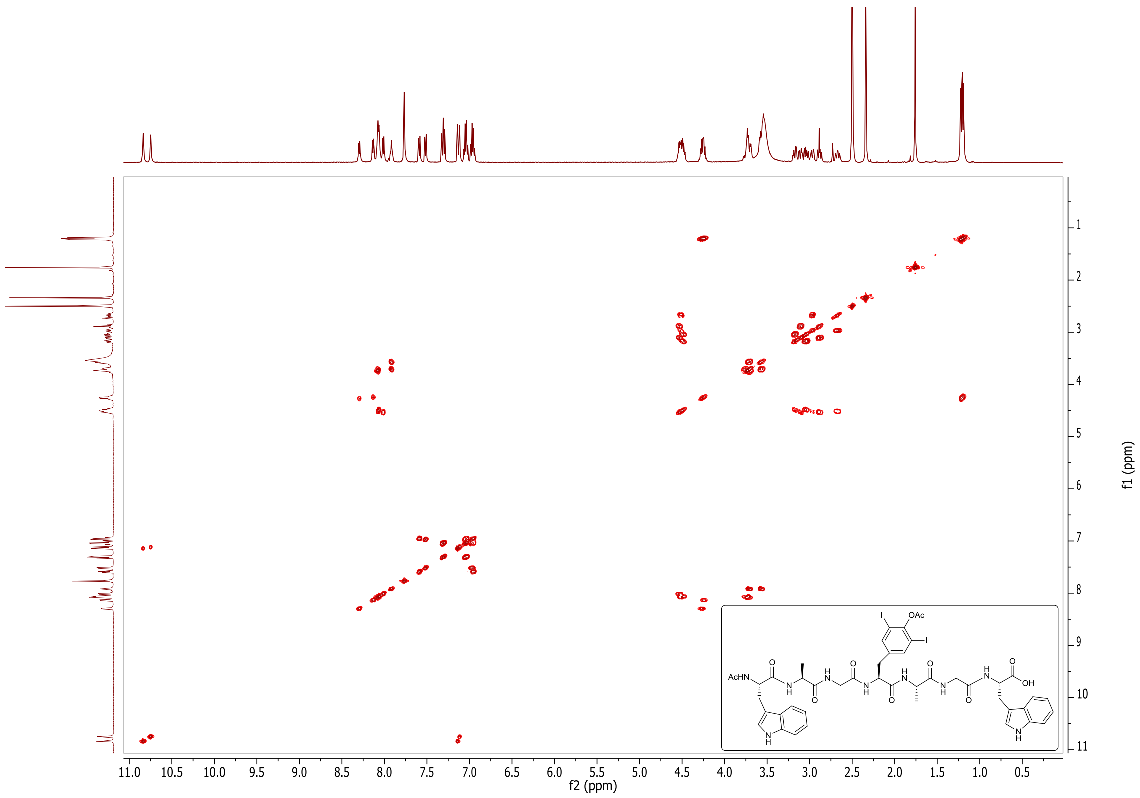


**Supplementary Figure 130 | COSY NMR spectrum of compound Ac-Trp-Ala-Gly-*3,5*-I,I-Tyr(OAc)-Ala-Gly-Trp-OH (11).**

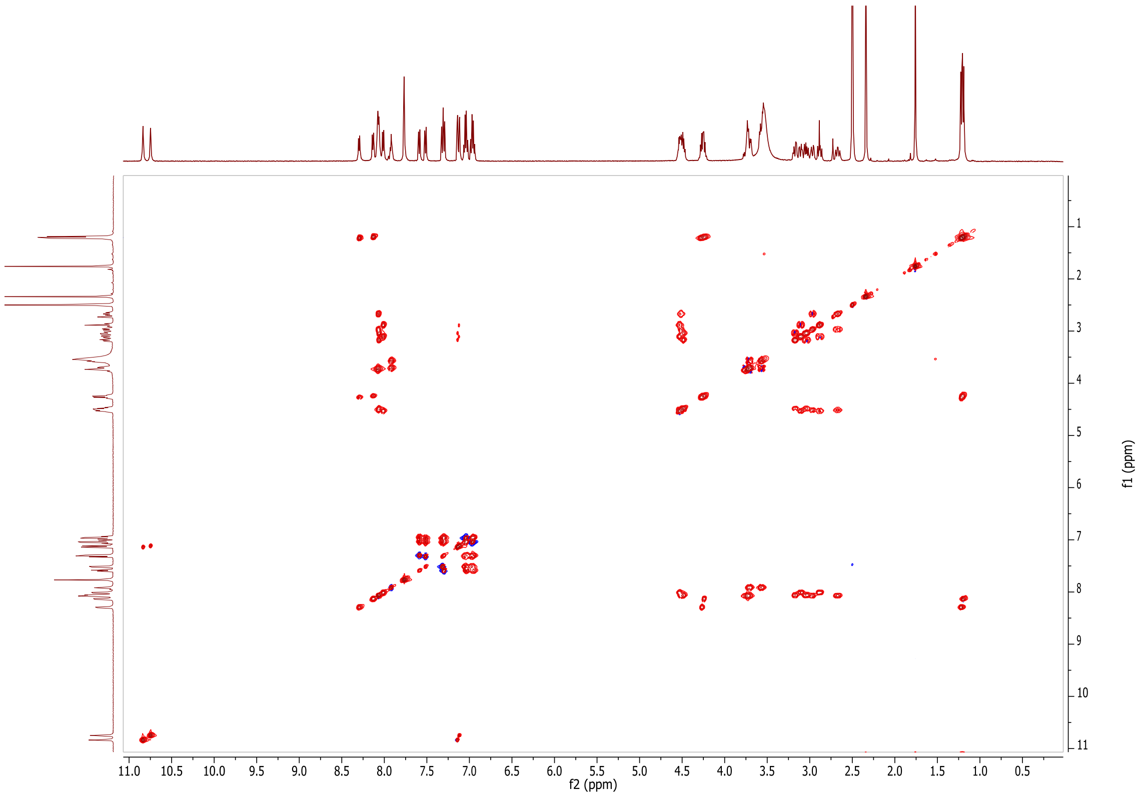


**Supplementary Figure 131 | TOCSY NMR spectrum of compound Ac-Trp-Ala-Gly-*3,5*-I,I-Tyr(OAc)-Ala-Gly-Trp-OH (11).**

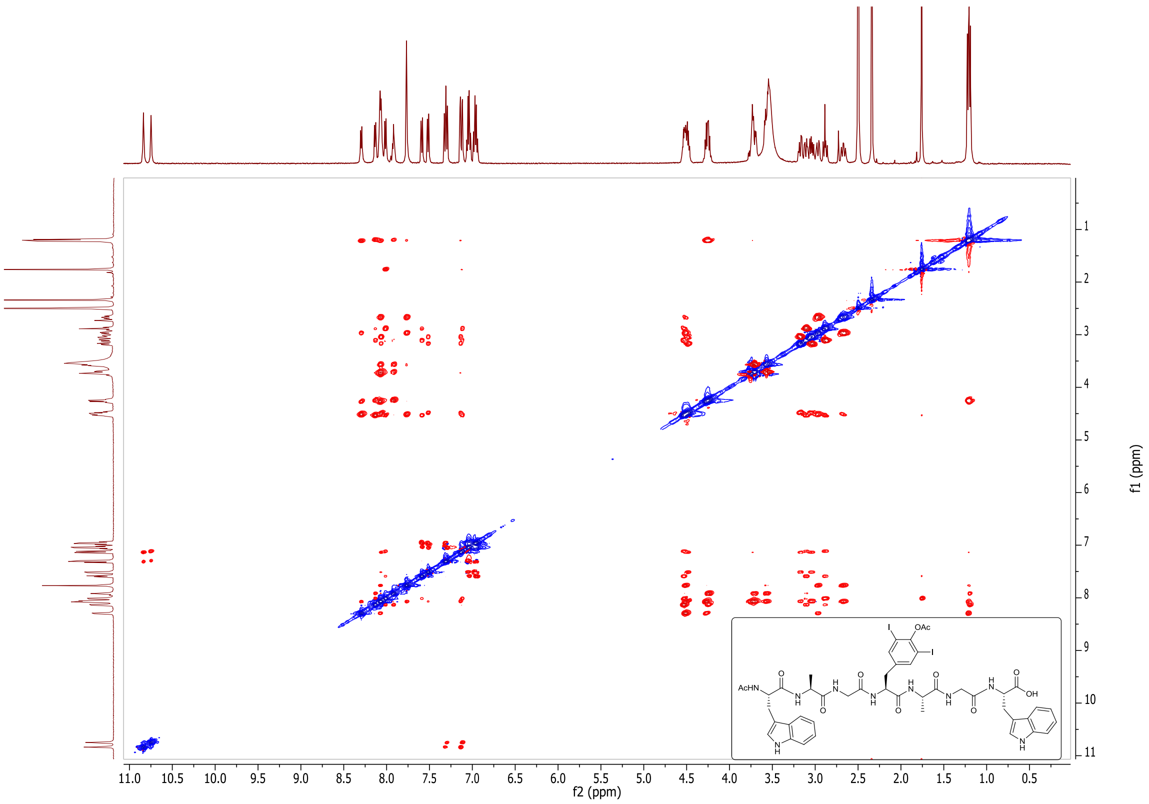


**Supplementary Figure 132 | ROESY NMR spectrum of compound Ac-Trp-Ala-Gly-*3,5*-I,I-Tyr(OAc)-Ala-Gly-Trp-OH (11).**

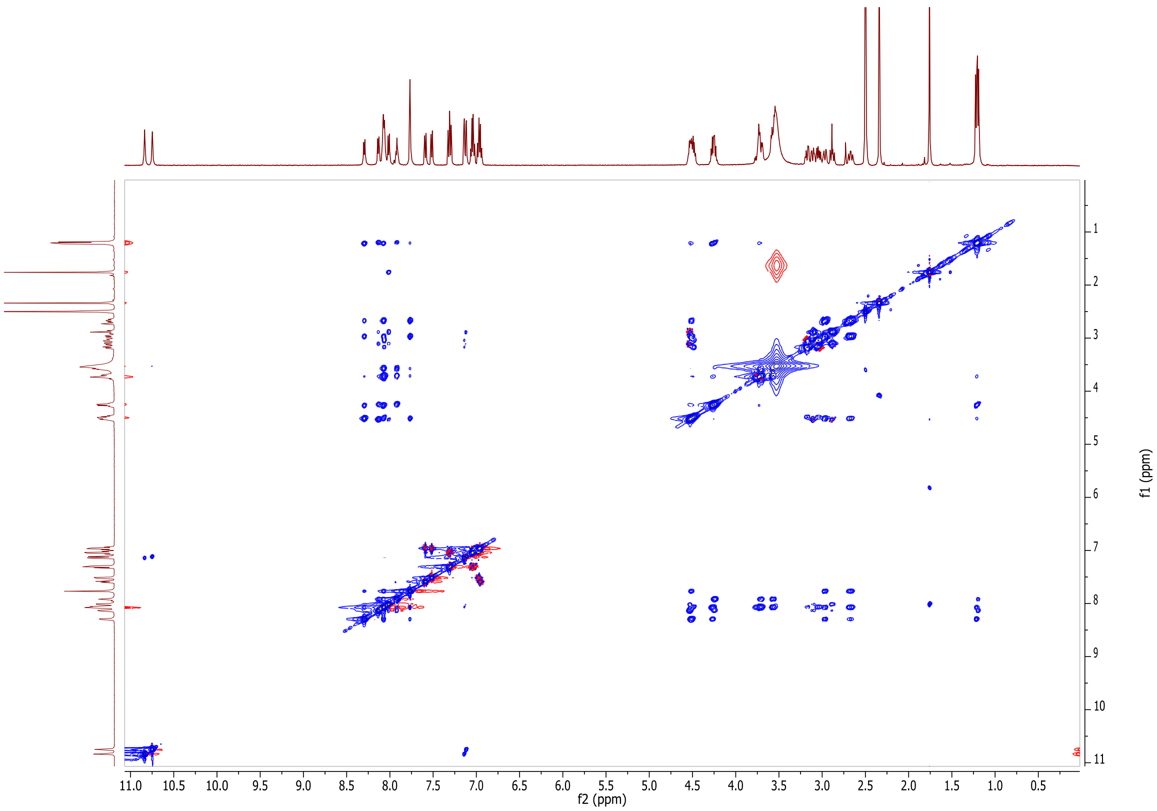


**Supplementary Figure 133 | NOESY NMR spectrum of compound Ac-Trp-Ala-Gly-*3,5*-I,I-Tyr(OAc)-Ala-Gly-Trp-OH (11).**

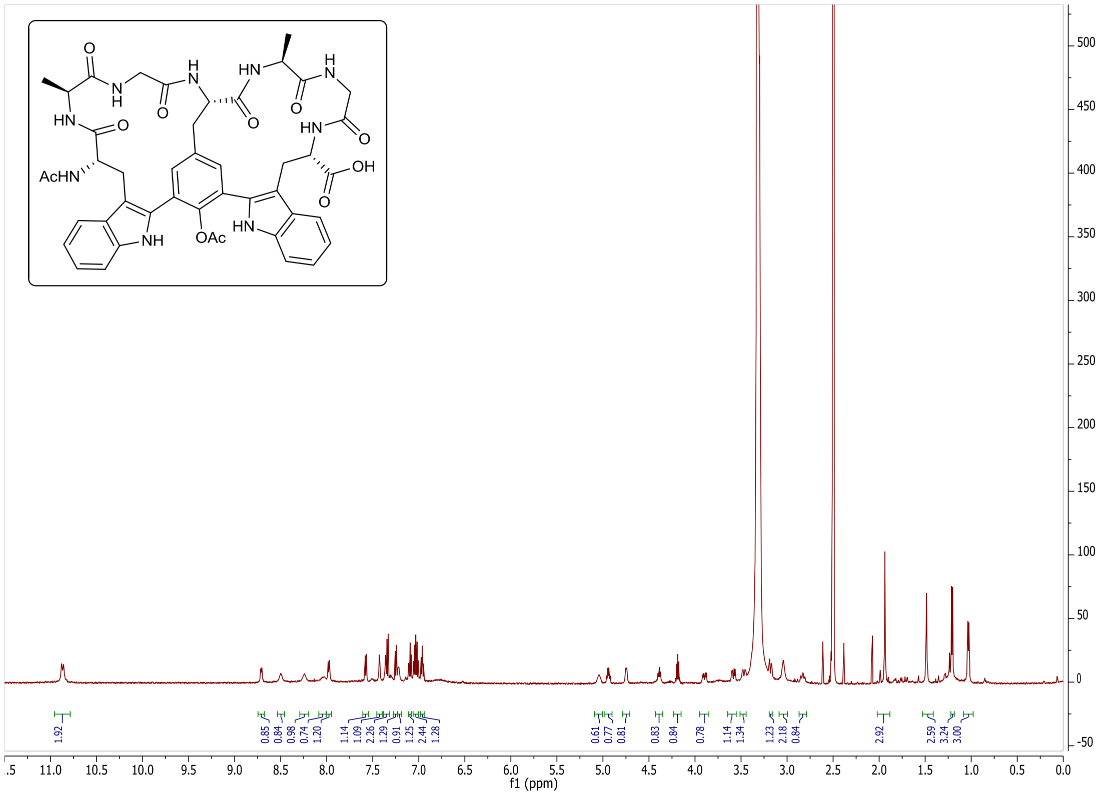

**Supplementary Figure 134 | $^{1}$H NMR spectrum of compound Ac-(bicyclo-*m,m*)-[Trp-Ala-Gly-Tyr(OAc)]-[Tyr(OAc)-Ala-Gly-Trp]-OH (12).**

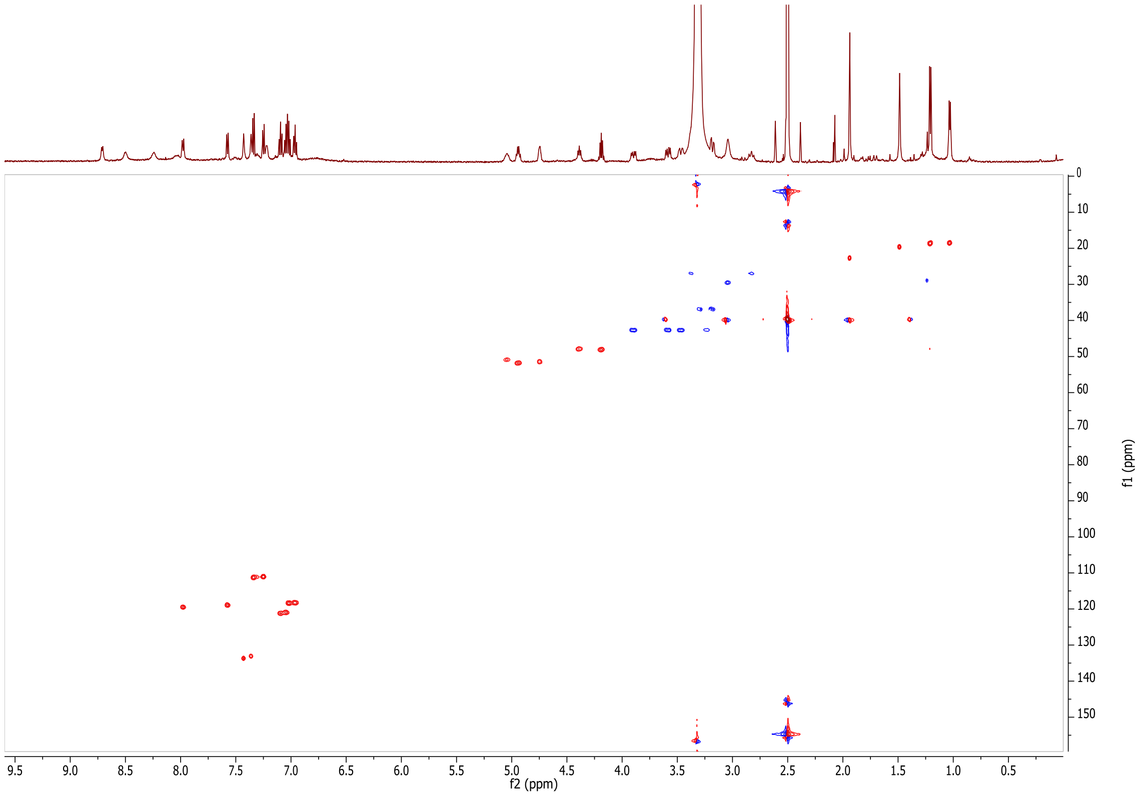

**Supplementary Figure 135 | $^{1}$H-$^{13}$C HSQC NMR spectrum of compound Ac-(bicyclo-*m,m*)-[Trp-Ala-Gly-Tyr(OAc)]-[Tyr(OAc)-Ala-Gly-Trp]-OH (12).**

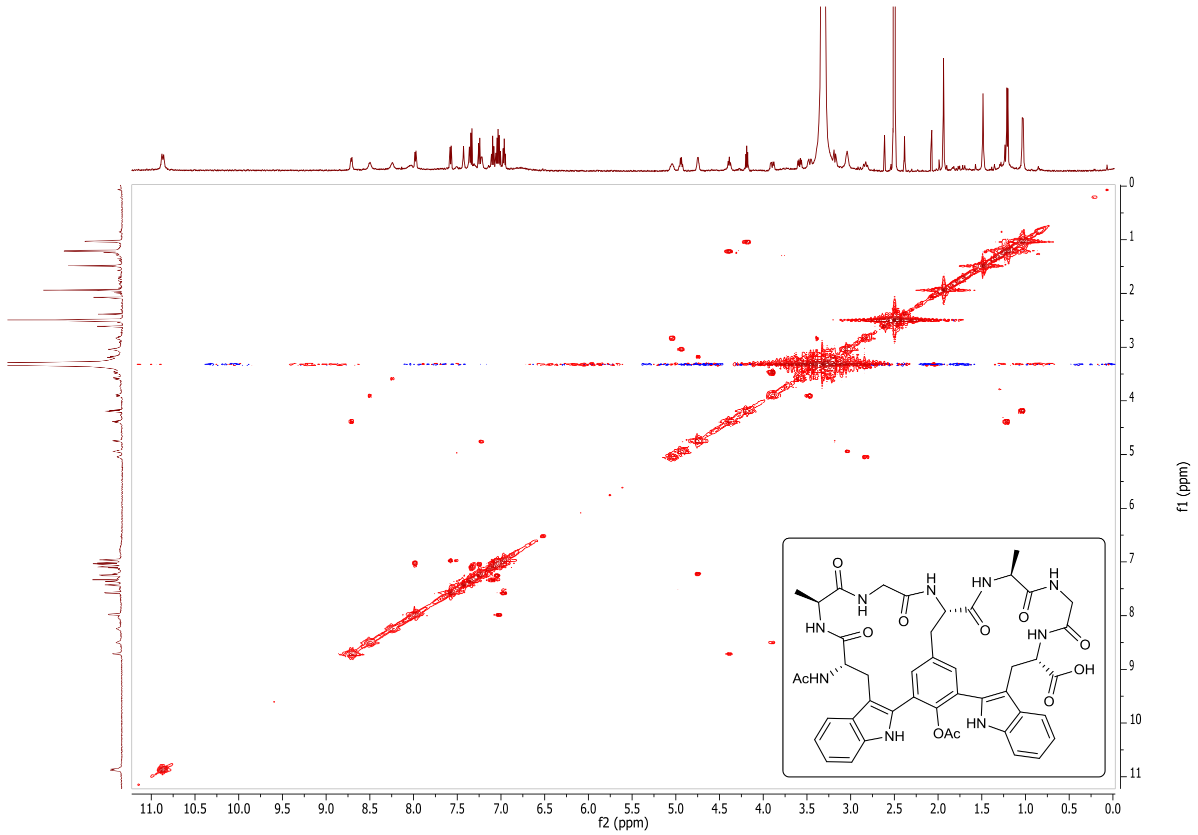


**Supplementary Figure 136 | COSY NMR spectrum of compound Ac-(bicyclo-*m,m*)-[Trp-Ala-Gly-Tyr(OAc)]-[Tyr(OAc)-Ala-Gly-Trp]-OH (12).**

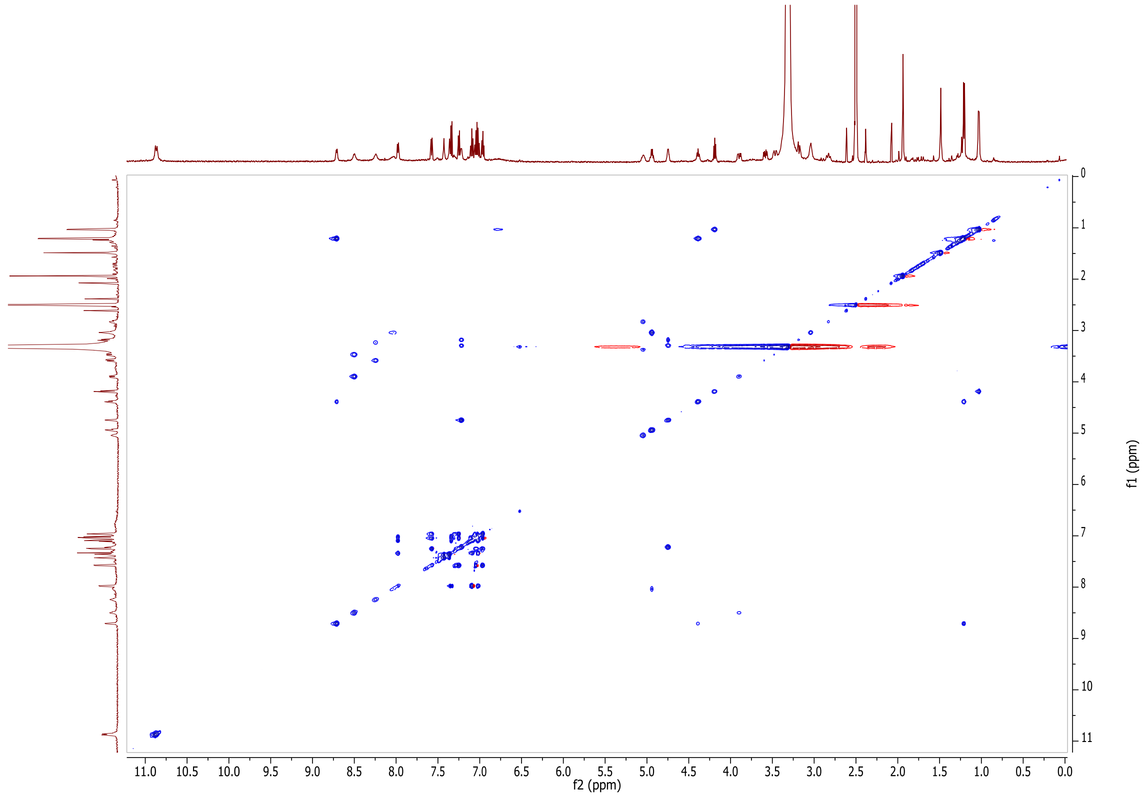


**Supplementary Figure 137 | TOCSY NMR spectrum of compound Ac-(bicyclo-*m,m*)-[Trp-Ala-Gly-Tyr(OAc)]-[Tyr(OAc)-Ala-Gly-Trp]-OH (12).**

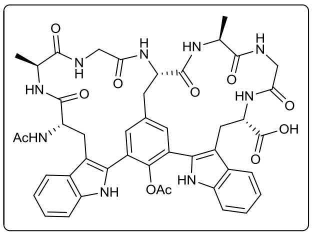


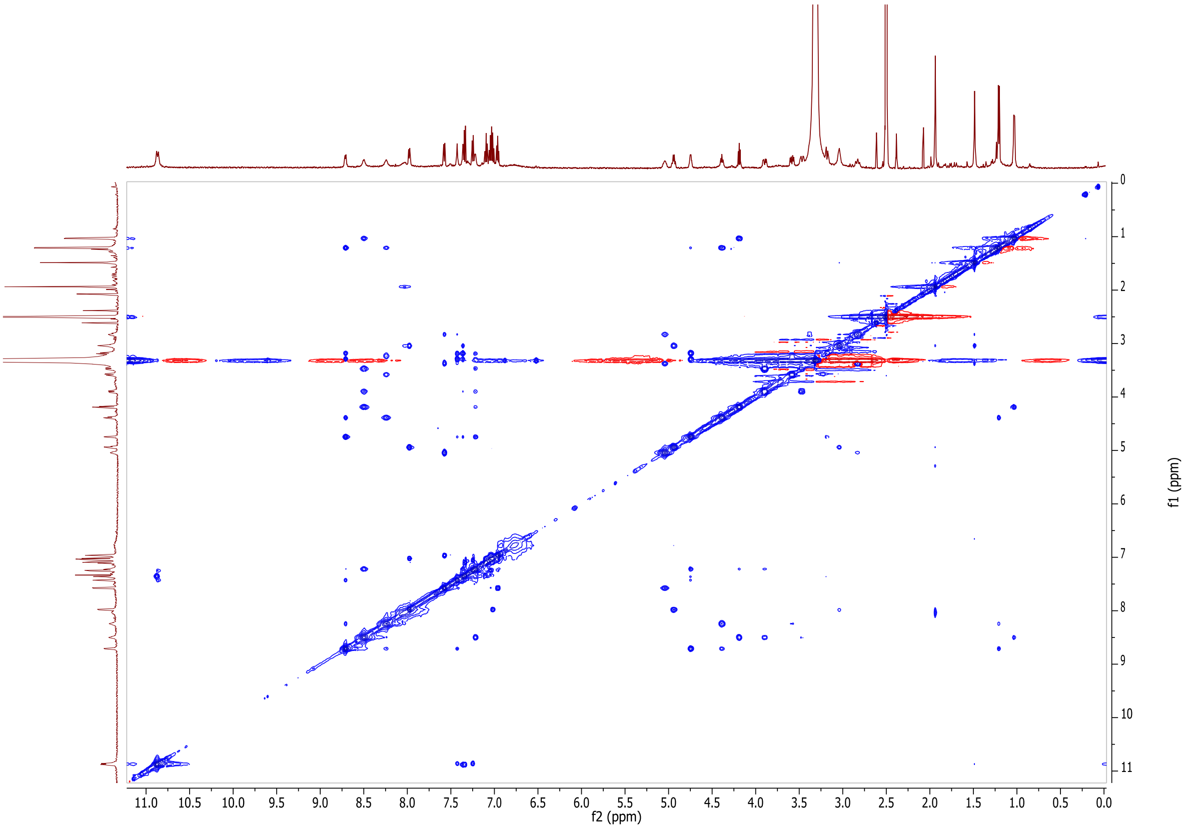


**Supplementary Figure 138 | NOESY NMR spectrum of compound Ac-(bicyclo-*m,m*)-[Trp-Ala-Gly-Tyr(OAc)]-[Tyr(OAc)-Ala-Gly-Trp]-OH (12).**

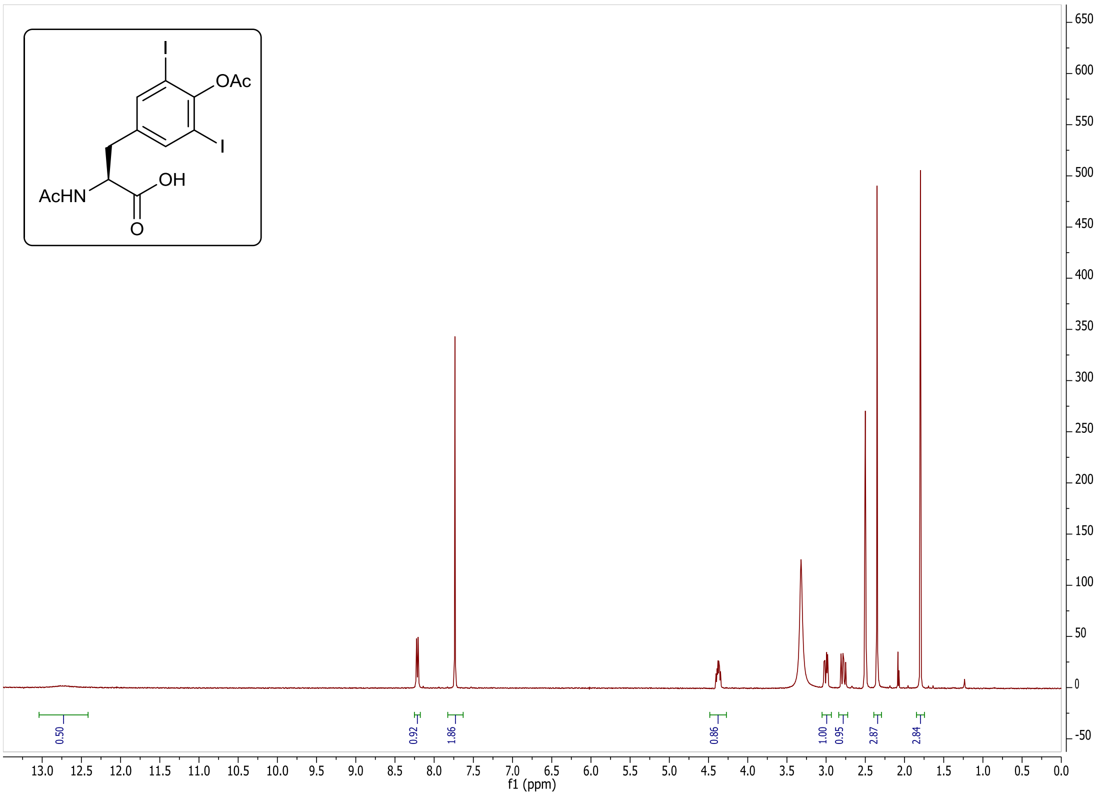


**Supplementary Figure 139 | $^{1}$H NMR spectrum of compound Ac-*m,m'*-I,I-Tyr(OAc)-OH (13).**

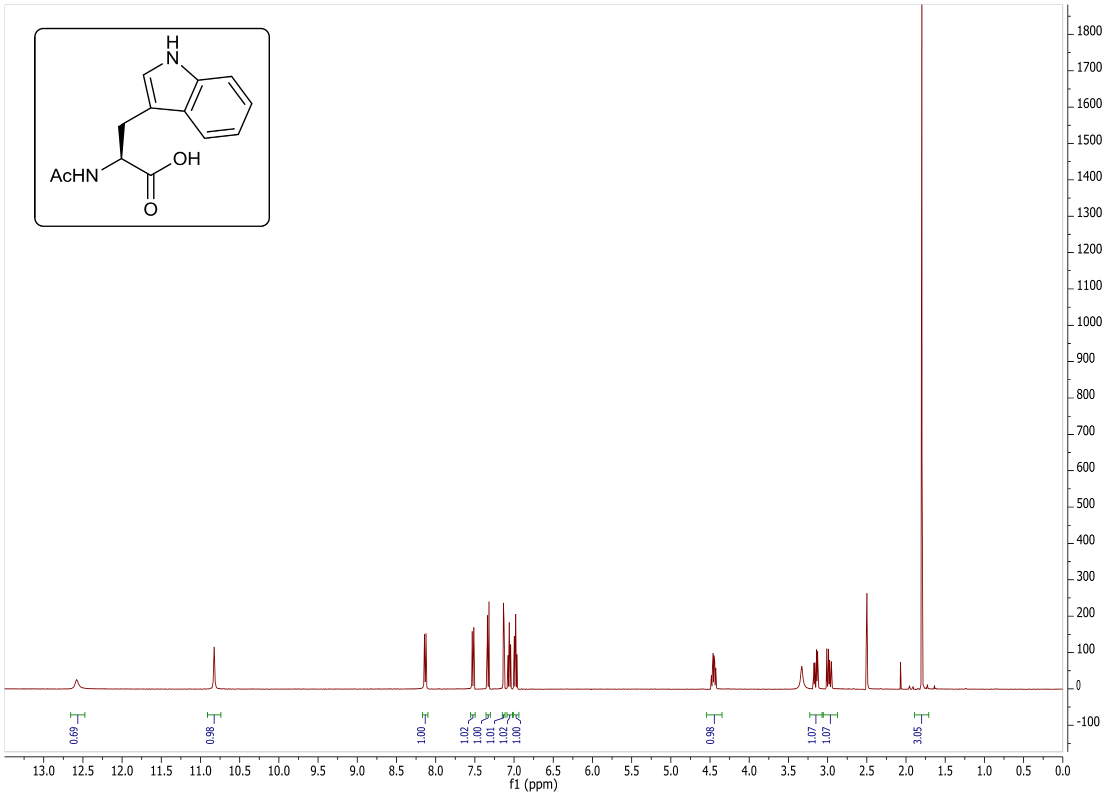


**Supplementary Figure 140 | $^{1}$H NMR spectrum of compound Ac-Trp-OH (14).**

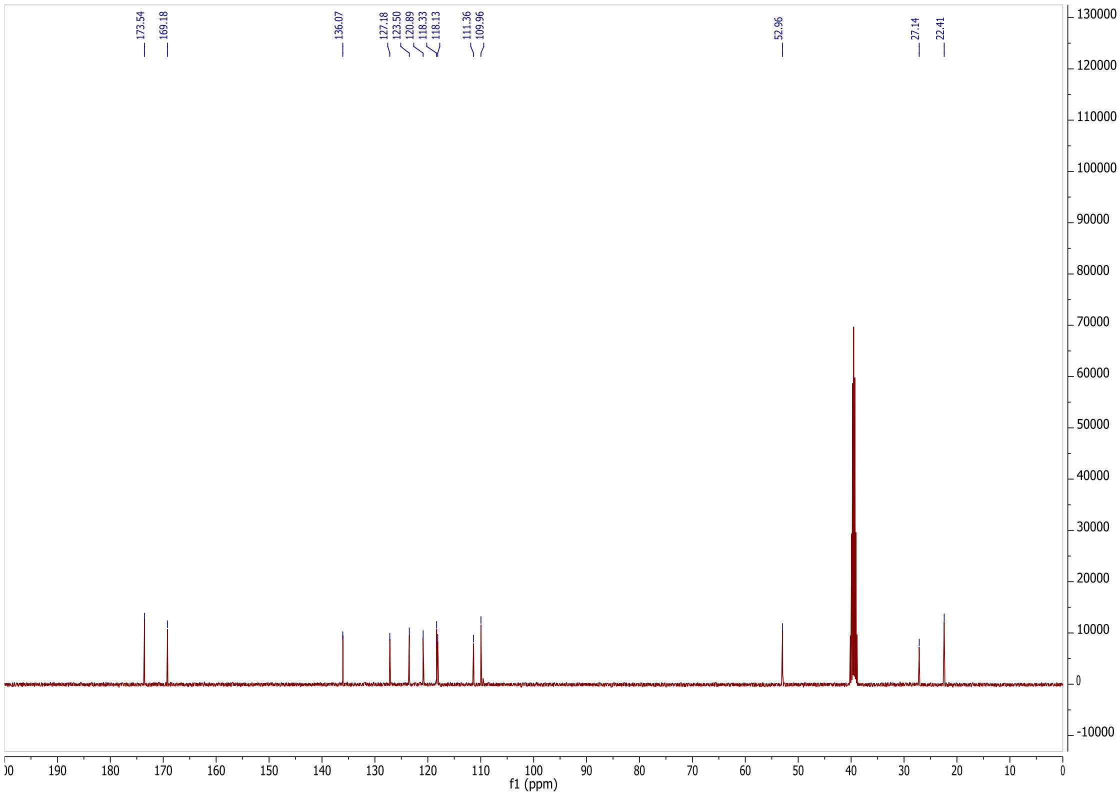


**Supplementary Figure 141 | $^{13}$C NMR spectrum of compound Ac-Trp-OH (14).**

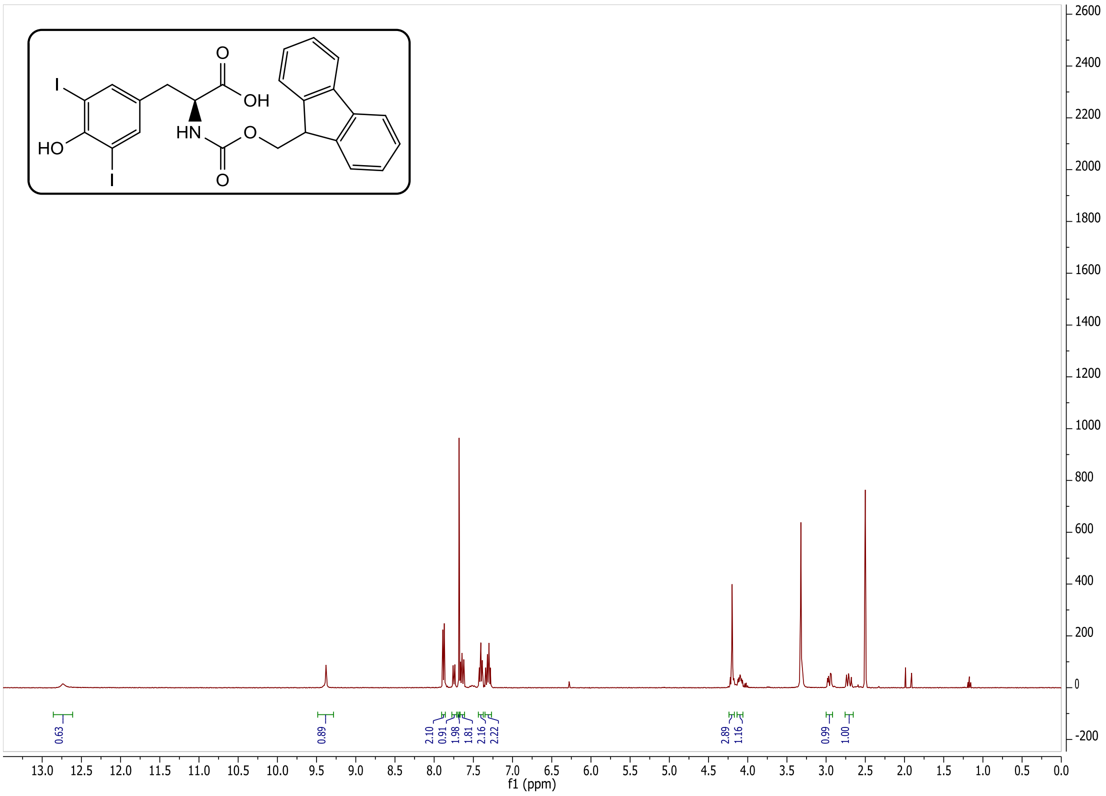

**Supplementary Figure 142 | $^1$H NMR spectrum of compound Fmoc-3,5-diiodo-L-Tyr-OH (15).**

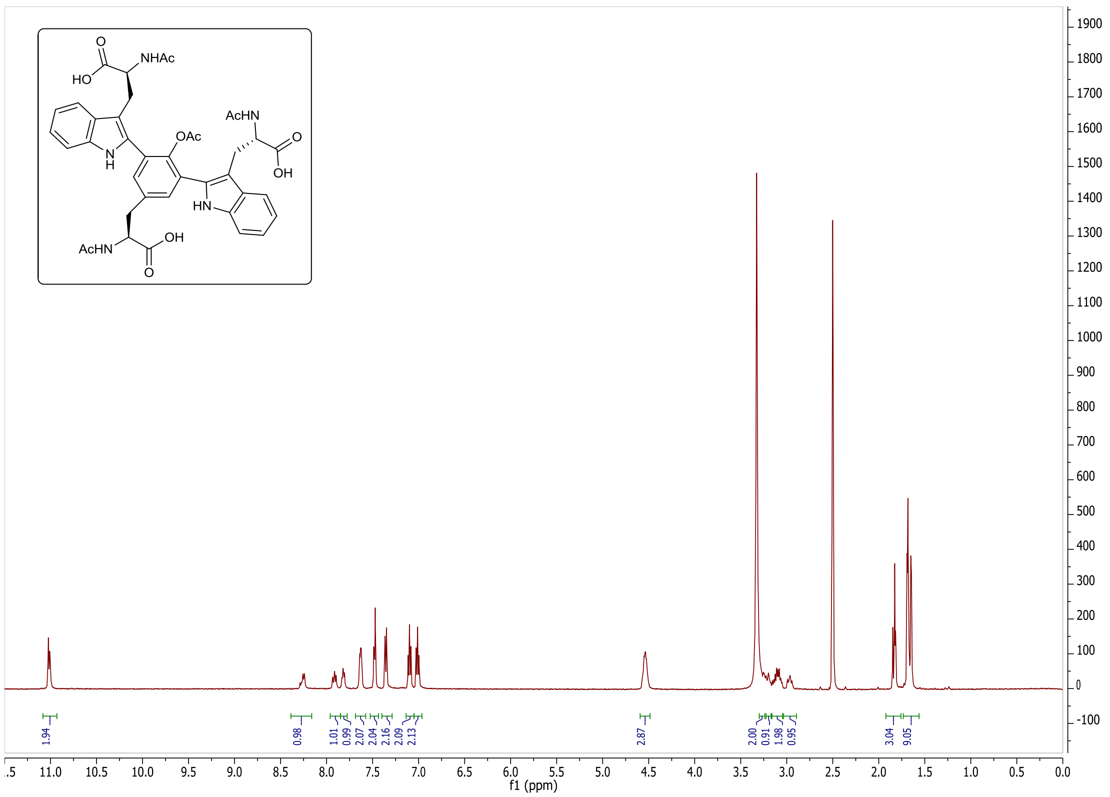

**Supplementary Figure 143 | $^{1}H$ NMR spectrum of compound Ac-*3,5*-di-(Ac-Trp-OH)-Tyr(OAc)-OH (16).**

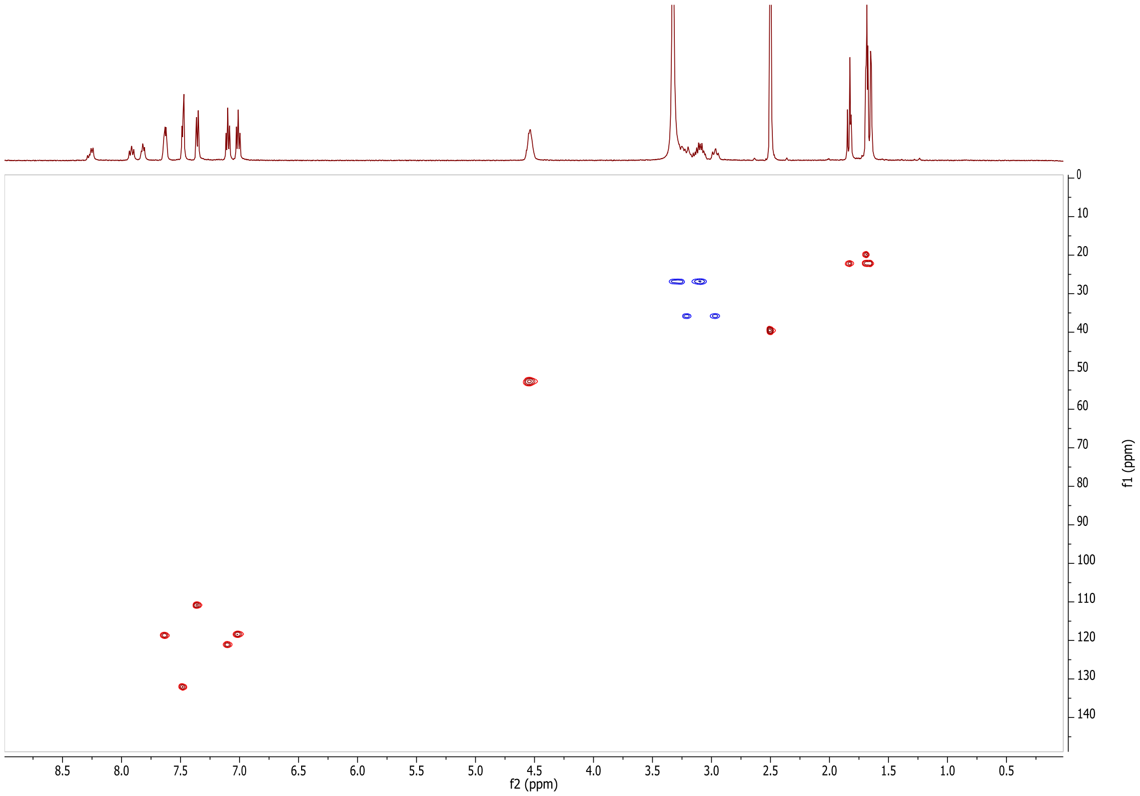

**Supplementary Figure 144 | $^{1}H$-$^{13}C$ HSQC NMR spectrum of compound Ac-*3,5*-di-(Ac-Trp-OH)-Tyr(OAc)-OH (16).**

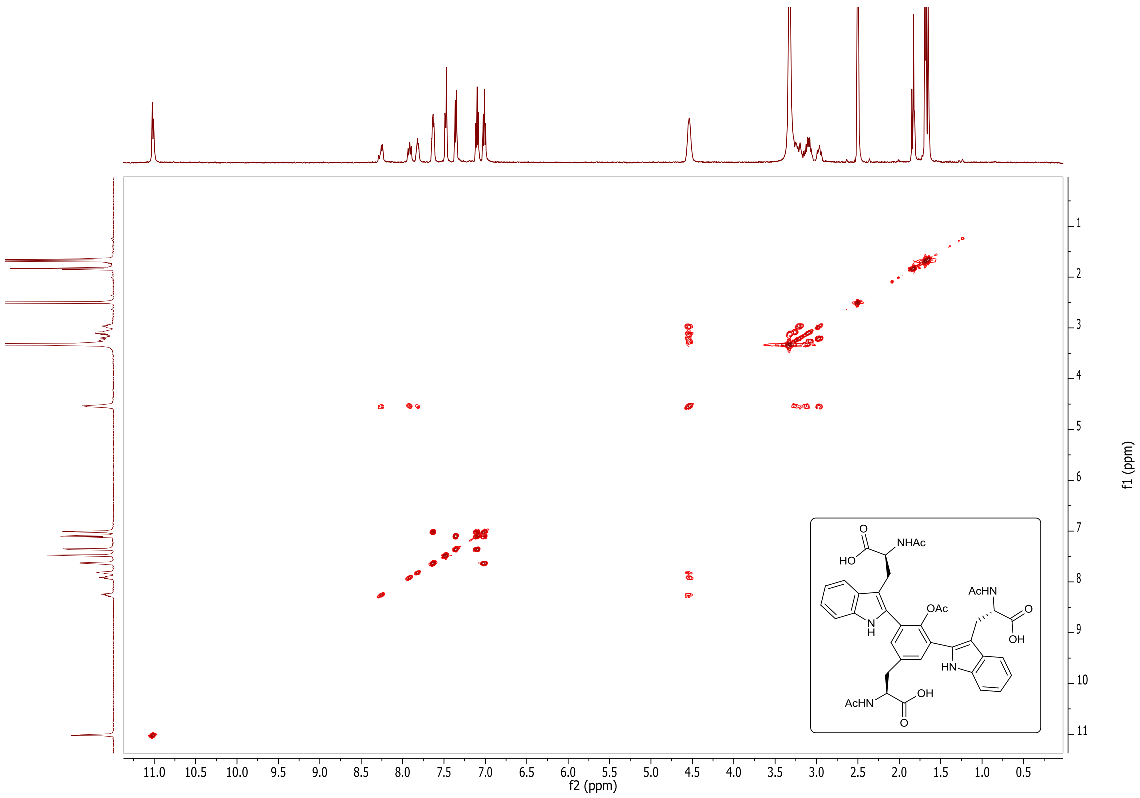


**Supplementary Figure 145 | COSY NMR spectrum of compound Ac-*3,5*-di-(Ac-Trp-OH)-Tyr(OAc)-OH (16).**

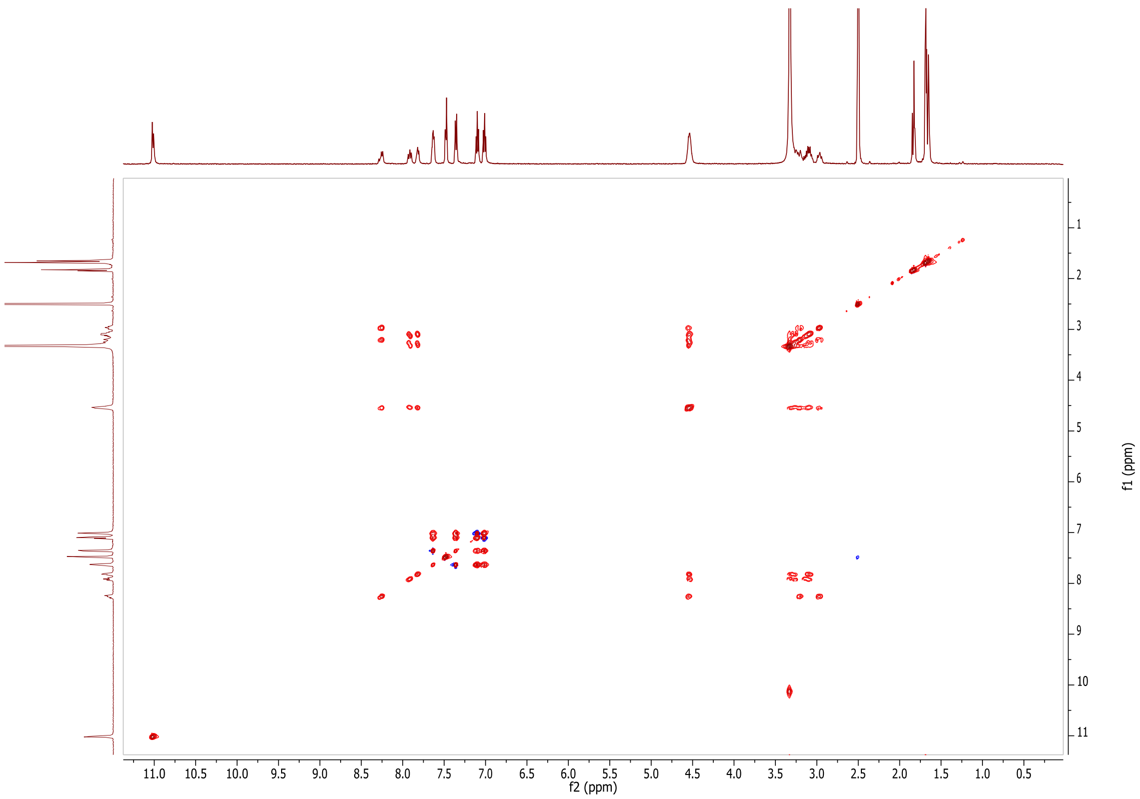


**Supplementary Figure 146 | TOCSY NMR spectrum of compound Ac-*3,5*-di-(Ac-Trp-OH)-Tyr(OAc)-OH (16).**

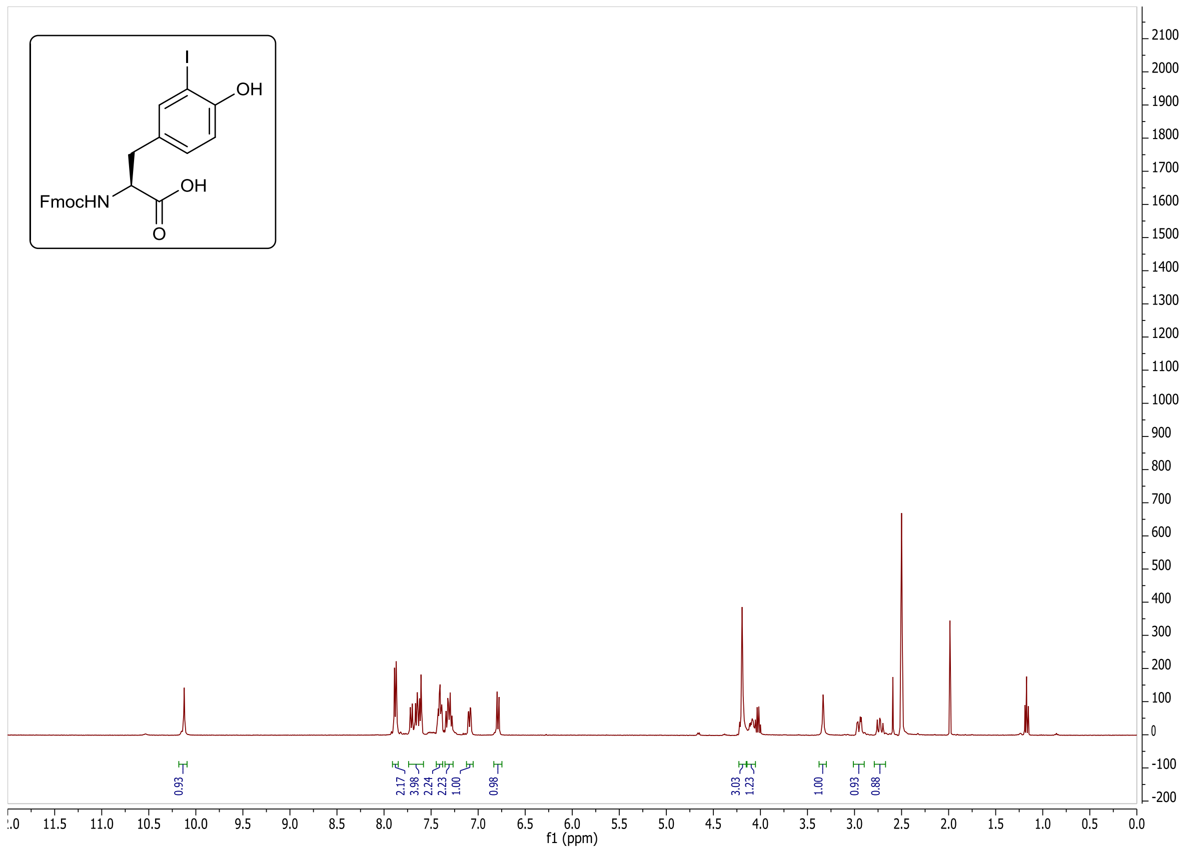


**Supplementary Figure 147 | $^{1}$H NMR spectrum of compound Fmoc-*3*-iodo-Tyr-OH (17).**

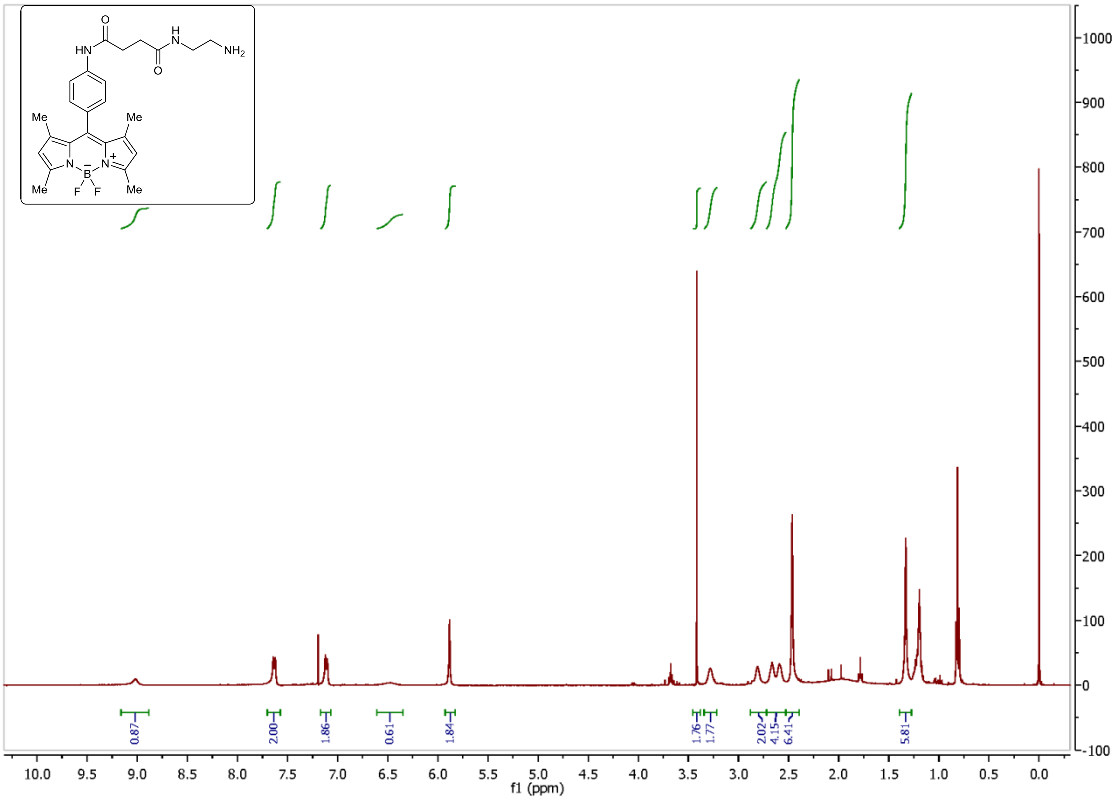

**Supplementary Figure 148 | $^{1}$H NMR spectrum of compound 10-(4-(4-(2-aminoethylamino)-4-oxobutanamido)phenyl)-5,5-difluoro-1,3,7,9-tetramethyl-5*H*-dipyrrolo[1,2-*c*:1',2'-*f*][1,3,2]diazaborinin-4-ium-5-uide (19).**

# Supplementary Tables

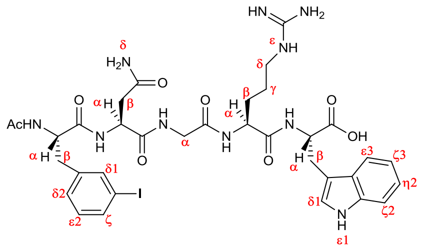

| 1g ($^1$H) | δ (ppm) | | | | | | | | | | | | |
|---|---|---|---|---|---|---|---|---|---|---|---|---|---|
| **AA** | **NH** | **α** | **β** | **γ** | **δ1** | **δ2** | **ε2** | **ζ** | **ε1** | **ζ2** | **η2** | **ζ3** | **ε3** |
| **Phe** | 8.18 | 4.47 | 2.98/ 2.66 | - | 7.66 | 7.25 | 7.05 | 7.54 | - | - | - | - | - |
| **Asn** | 8.38 | 4.51 | 2.57/ 2.50 | - | 7.46/6.96 | - | - | - | - | - | - | - | - |
| **Gly** | 8.03 | 3.76/ 3.66 | - | - | - | - | - | - | - | - | - | - | - |
| **Arg** | 7.92 | 4.36 | 1.68/ 1.54 | 1.46 | 3.07 | - | - | - | 7.46 | - | - | - | - |
| **Trp** | 8.14 | 4.45 | 3.17/ 3.07 | - | 7.16 | - | - | - | 10.83 | 7.32 | 7.05 | 6.97 | 7.51 |

**Supplementary Table 1 | $^1$H chemical shifts assignments of compound Ac-*m*-I-Phe-Asn-Gly-Arg-Trp-OH (1g).**

| 1g ($^{13}$C) | δ (ppm) | | | | | | | | | | |
|---|---|---|---|---|---|---|---|---|---|---|---|
| **AA** | **α** | **β** | **γ** | **δ1** | **δ2** | **ε2** | **ζ** | **ζ2** | **η2** | **ζ3** | **ε3** |
| **Phe** | 53.6 | 36.5 | - | 137.4 | 128.4 | 130.0 | 134.8 | - | - | - | - |
| **Asn** | 49.6 | 36.7 | - | - | - | - | - | - | - | - | - |
| **Gly** | 42.2 | - | - | - | - | - | - | - | - | - | - |
| **Arg** | 51.5 | 28.9 | 24.7 | 40.2 | - | - | - | - | - | - | - |
| **Trp** | 53.0 | 26.9 | - | 123.4 | - | - | - | 111.1 | 120.7 | 118.1 | 117.9 |

**Supplementary Table 2 | $^{13}$C chemical shifts assignments of compound Ac-*m*-I-Phe-Asn-Gly-Arg-Trp-OH (1g).**

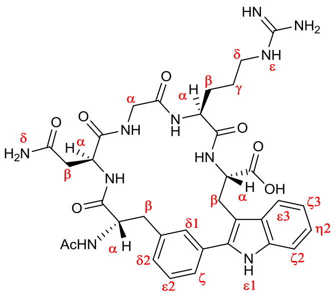

| 2g ($^{1}$H) | δ (ppm) | | | | | | | | | | | | |
|---|---|---|---|---|---|---|---|---|---|---|---|---|---|
| **AA** | **NH** | **α** | **β** | **γ** | **δ1** | **δ2** | **ε2** | **ζ** | **ε1** | **ζ2** | **η2** | **ζ3** | **ε3** |
| **Phe** | 8.36 | 4.68 | 3.09/2.88 | - | 7.70 | 7.20 | 7.37 | 7.49 | - | - | - | - | - |
| **Asn** | 8.42 | 4.60 | 2.62/2.44 | - | 7.38/6.85 | - | - | - | - | - | - | - | - |
| **Gly** | 7.67 | 3.98/3.43 | - | - | - | - | - | - | - | - | - | - | - |
| **Arg** | 7.79 | 4.30 | 1.74/1.55 | 1.41 | 3.04 | - | - | - | - | - | - | - | - |
| **Trp** | 8.33 | 4.48 | 3.42/3.06 | - | - | - | - | - | 11.15 | 7.32 | 7.06 | 6.97 | 7.63 |

**Supplementary Table 3 | $^{1}$H chemical shifts assignments of compound Ac-(Cyclo-*m*)-[Phe-Asn-Gly-Arg-Trp]-OH (2g).**

| 2g ($^{13}$C) | δ (ppm) | | | | | | | | | | |
|---|---|---|---|---|---|---|---|---|---|---|---|
| **AA** | **α** | **β** | **γ** | **δ1** | **δ2** | **ε2** | **ζ** | **ζ2** | **η2** | **ζ3** | **ε3** |
| **Phe** | 53.4 | 37.0 | - | 128.0 | 127.6 | 127.9 | 125.0 | - | - | - | - |
| **Asn** | 49.2 | 35.7 | - | - | - | - | - | - | - | - | - |
| **Gly** | 42.5 | - | - | - | - | - | - | - | - | - | - |
| **Arg** | 51.6 | 28.4 | 23.9 | 39.9 | - | - | - | - | - | - | - |
| **Trp** | 54.2 | 28.2 | - | | - | - | - | 110.6 | 121.1 | 118.3 | 119.0 |

**Supplementary Table 4 | $^{13}$C chemical shifts assignments of compound Ac-(Cyclo-*m*)-[Phe-Asn-Gly-Arg-Trp]-OH (2g).**

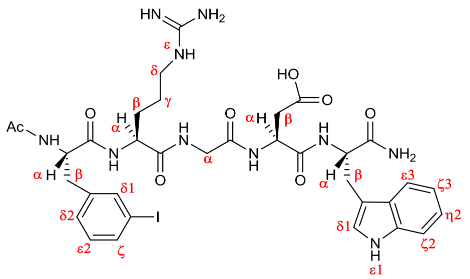

| 1h ($^{1}$H) | δ (ppm) | | | | | | | | | | | | |
|---|---|---|---|---|---|---|---|---|---|---|---|---|---|
| **AA** | **NH** | **α** | **β** | **γ** | **δ1** | **δ2** | **ε2** | **ζ** | **ε1** | **ζ2** | **η2** | **ζ3** | **ε3** |
| **Phe** | 8.10 | 4.51 | 2.98/2.67 | - | 7.69 | 7.27 | 7.05 | 7.55 | - | - | - | - | - |
| **Arg** | 8.23 | 4.31 | 1.72/1.57 | 1.51 | 3.10 | - | - | - | 7.42 | - | - | - | - |
| **Gly** | 8.10 | 3.72 | - | - | - | - | - | - | - | - | - | - | - |
| **Asp** | 8.23 | 4.57 | 2.69/2.46 | - | - | - | - | - | - | - | - | - | - |
| **Trp** | 7.85 | 4.39 | 3.16/2.98 | - | 7.10 | - | - | - | 10.74 | 7.32 | 7.05 | 6.97 | 7.55 |

**Supplementary Table 5 | $^{1}$H chemical shifts assignments of compound Ac-*m*-I-Phe-Arg-Gly-Asp-Trp-OH (1h).**

| 1h ($^{13}$C) | δ (ppm) | | | | | | | | | | |
|---|---|---|---|---|---|---|---|---|---|---|---|
| **AA** | **α** | **β** | **γ** | **δ1** | **δ2** | **ε2** | **ζ** | **ζ2** | **η2** | **ζ3** | **ε3** |
| **Phe** | 53.5 | 36.5 | - | 137.3 | 128.4 | 129.8 | 134.6 | - | - | - | - |
| **Arg** | 52.0 | 28.9 | 24.5 | 40.1 | - | - | - | - | - | - | - |
| **Gly** | 41.5 | - | - | - | - | - | - | - | - | - | - |
| **Asp** | 49.2 | 35.8 | - | - | - | - | - | - | - | - | - |
| **Trp** | 53.1 | 27.1 | - | 123.2 | - | - | - | 110.9 | 120.5 | 117.9 | 118.0 |

**Supplementary Table 6 | $^{13}$C chemical shifts assignments of compound Ac-*m*-I-Phe-Arg-Gly-Asp-Trp-OH (1h).**

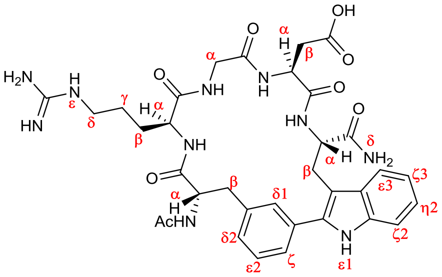


| 2h ($^{1}H$) | | | | | δ (ppm) | | | | | | | | |
|---|---|---|---|---|---|---|---|---|---|---|---|---|---|
| **AA** | **NH** | **α** | **β** | **γ** | **δ1** | **δ2** | **ε2** | **ζ** | **ε1** | **ζ2** | **η2** | **ζ3** | **ε3** |
| **Phe** | 8.32 | 4.66 | 2.97 | - | 7.69 | 7.24 | 7.39 | 7.42 | - | - | - | - | - |
| **Arg** | 7.88 | 4.03 | 1.84/ 1.49 | 1.41/ 1.34 | 3.14/ 2.75 | - | - | - | - | - | - | - | - |
| **Gly** | 8.39 | 3.68/3.43 | - | - | - | - | - | - | - | - | - | - | - |
| **Asp** | 8.06 | 4.25 | 2.67/2.18 | - | - | - | - | - | - | - | - | - | - |
| **Trp** | 7.35 | 4.53 | 3.43/2.97 | - | 7.07/6.89 | - | - | - | 11.00 | 7.31 | 7.07 | 6.99 | 7.59 |

**Supplementary Table 7 | $^{1}H$ chemical shifts assignments of compound Ac-(Cyclo-*m*)-[Phe-Arg-Gly-Asp-Trp]-$NH_2$ (2h).**

| 2h ($^{13}C$) | | | | δ (ppm) | | | | | | | |
|---|---|---|---|---|---|---|---|---|---|---|---|
| **AA** | **α** | **β** | **γ** | **δ1** | **δ2** | **ε2** | **ζ** | **ζ2** | **η2** | **ζ3** | **ε3** |
| **Phe** | 53.5 | 38.4 | - | 128.8 | 127.8 | 128.0 | 126.0 | - | - | - | - |
| **Arg** | 52.5 | 30.3 | 24.6 | 40.4 | - | - | - | - | - | - | - |
| **Gly** | 43.1 | - | - | - | - | - | - | - | - | - | - |
| **Asp** | 48.9 | 37.2 | - | - | - | - | - | - | - | - | - |
| **Trp** | 53.1 | 26.8 | - | - | - | - | - | 110.6 | 120.8 | 118.2 | 118.5 |

**Supplementary Table 8 | $^{13}C$ chemical shifts assignments of compound Ac-(Cyclo-*m*)-[Phe-Arg-Gly-Asp-Trp]-$NH_2$ (2h).**

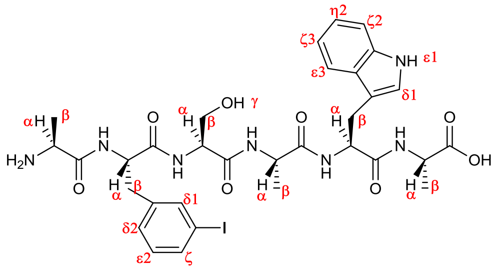

| 1i ($^{1}$H) | | | | | | | δ (ppm) | | | | | | |
|---|---|---|---|---|---|---|---|---|---|---|---|---|---|
| **AA** | **NH** | **α** | **β** | **δ1** | **δ2** | **ε2** | **ζ** | **ε1** | **ζ2** | **η2** | **ζ3** | **ε3** | **γ** |
| **Ala1** | - | 3.70 | 1.31-1.21 | - | - | - | - | - | - | - | - | - | - |
| ***m-I*-Phe** | 8.73 | 4.58 | 3.04/2.76 | 7.71 | 7.3 | 7.06 | 7.57 | - | - | - | - | - | - |
| **Ser** | 8.36 | 4.35 | 3.7/3.59 | - | - | - | - | - | - | - | - | - | 5.39 |
| **Ala2** | 8.15 | 4.17 | 1.12 | - | - | - | - | - | - | - | - | - | - |
| **Trp** | 7.94 | 4.48 | 3.17/2.88 | 7.12 | - | - | - | 10.8 | 7.30 | 7.06 | 6.97 | 7.57 | - |
| **Ala3** | 7.94 | 4.10 | 1.31-1.21 | - | - | - | - | - | - | - | - | - | - |

**Supplementary Table 9 | $^{1}$H chemical shifts assignments of compound H-Ala-*m*-I-Phe-Ser-Ala-Trp-Ala-OH (1i).**

| 1i ($^{13}$C) | | | | | | δ (ppm) | | | | | |
|---|---|---|---|---|---|---|---|---|---|---|---|
| **AA** | **α** | **β** | **δ1** | **δ2** | **ε2** | **ζ** | **ζ2** | **η2** | **ζ3** | **ε3** | **γ** |
| **Ala1** | 48.0 | 17.4 | - | - | - | - | - | - | - | - | - |
| ***m-I*-Phe** | 53.8 | 36.5 | 137.4 | 128.5 | 129.9 | 134.7 | - | - | - | - | - |
| **Ser** | 54.3 | 61.7 | - | - | - | - | - | - | - | - | |
| **Ala2** | 48.6 | 17.5 | - | - | - | - | - | - | - | - | - |
| **Trp** | 53.0 | 27.4 | 123.1 | - | - | - | 110.9 | 120.5 | 117.9 | 118.0 | - |
| **Ala3** | 48.0 | 17.4 | - | - | - | - | - | - | - | - | - |

**Supplementary Table 10 | $^{13}$C chemical shifts assignments of compound H-Ala-*m*-I-Phe-Ser-Ala-Trp-Ala-OH (1i).**

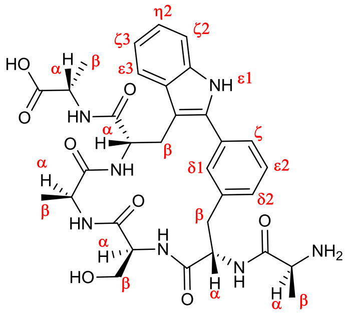

| 2i ($^{1}$H) | | | | | | | δ (ppm) | | | | | |
|---|---|---|---|---|---|---|---|---|---|---|---|---|
| **AA** | **NH** | **α** | **β** | **δ1** | **δ2** | **ε2** | **ζ** | **ε1** | **ζ2** | **η2** | **ζ3** | **ε3** |
| **Ala1** | - | 3.48 | 1.17 | - | - | - | - | - | - | - | - | - |
| **Phe** | 8.46 | 4.97 | 3.18/3.08 | 7.65 | 7.22 | 7.42 | 7.42 | - | - | - | - | - |
| **Ser** | 8.35 | 3.87 | 3.63/3.58 | - | - | - | - | - | - | - | - | - |
| **Ala2** | 7.37 | 3.70 | 0.76 | - | - | - | - | - | - | - | - | - |
| **Trp** | 6.81 | 4.51 | 3.63/3.30 | - | - | - | - | 11.1 | 7.34 | 7.11 | 7.02 | 7.61 |
| **Ala3** | 7.51 | 4.15 | 1.32 | - | - | - | - | - | - | - | - | - |

**Supplementary Table 11 | $^{1}$H chemical shifts assignments of compound H-Ala-(Cyclo-*m)*-[Phe-Ser-Ala-Trp]-Ala-OH (2i).**

| 2i ($^{13}$C) | | | | | | δ (ppm) | | | | |
|---|---|---|---|---|---|---|---|---|---|---|
| **AA** | **α** | **β** | **δ1** | **δ2** | **ε2** | **ζ** | **ζ2** | **η2** | **ζ3** | **ε3** |
| **Ala1** | 49.1 | 19.8 | - | - | - | - | - | - | - | - |
| **Phe** | 51.5 | 36.3 | 128.5 | 128.3 | 128.1 | 125.4 | - | - | - | - |
| **Ser** | 58.0 | 59.9 | - | - | - | - | - | - | - | - |
| **Ala2** | 49.8 | 15.7 | - | - | - | - | - | - | - | - |
| **Trp** | 53.4 | 26.6 | - | - | - | - | 110.8 | 121.3 | 118.4 | 117.8 |
| **Ala3** | 47.6 | 16.6 | - | - | - | - | - | - | - | - |

**Supplementary Table 12 | $^{13}$C chemical shifts assignments of compound H-Ala-(Cyclo-*m)*-[Phe-Ser-Ala-Trp]-Ala-OH (2i).**

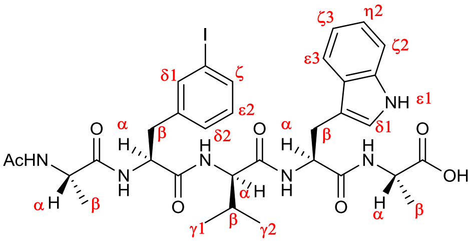


| 1j ($^{1}$H) | δ (ppm) | | | | | | | | | | | | |
|---|---|---|---|---|---|---|---|---|---|---|---|---|---|
| **AA** | **NH** | **α** | **β** | **δ1** | **δ2** | **ε2** | **ζ** | **ε1** | **ζ2** | **η2** | **ζ3** | **ε3** | **γ** |
| **Ala1** | 7.99 | 4.19 | 1.10 | - | - | - | - | - | - | - | - | - | - |
| ***m-I*-Phe** | 7.94 | 4.47 | 2.92/2.71 | 7.60 | 7.20 | 6.96 | 7.50 | - | - | - | - | - | - |
| **Val** | 7.76 | 4.15 | 1.94 | - | - | - | - | - | - | - | - | - | 0.77 |
| **Trp** | 7.99 | 4.60 | 3.13/2.92 | 7.15 | - | - | - | 10.78 | 7.29 | 7.04 | 6.96 | 7.60 | - |
| **Ala2** | 8.17 | 4.20 | 1.25 | - | - | - | - | - | - | - | - | - | - |

**Supplementary Table 13 | $^{1}$H chemical shifts assignments of compound Ac-Ala-*m*-I-Phe-Val-Trp-Ala-OH (1j).**

| 1j ($^{13}$C) | δ (ppm) | | | | | | | | | | |
|---|---|---|---|---|---|---|---|---|---|---|---|
| **AA** | **α** | **β** | **δ1** | **δ2** | **ε2** | **ζ** | **ζ2** | **η2** | **ζ3** | **ε3** | **γ** |
| **Ala1** | 47.5 | 17.9 | - | - | - | - | - | - | - | - | - |
| ***m-I*-Phe** | 53.2 | 36.3 | 137.4 | 128.5 | 129.9 | 134.7 | - | - | - | - | - |
| **Val** | 57.3 | 30.5 | - | - | - | - | - | - | - | - | 18.9/17.7 |
| **Trp** | 52.7 | 27.4 | 123.3 | - | - | - | 110.9 | 120.6 | 117.9 | 118.2 | - |
| **Ala2** | 47.7 | 17.0 | - | - | - | - | - | - | - | - | - |

**Supplementary Table 14 | $^{13}$C chemical shifts assignments of compound Ac-Ala-*m*-I-Phe-Val-Trp-Ala-OH (1j).**

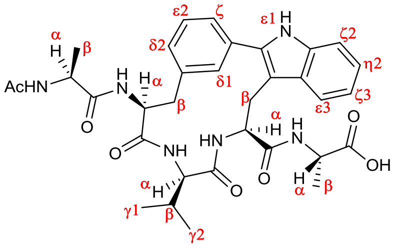


| 2j ($^{1}$H) | δ (ppm) | | | | | | | | | | | | |
|---|---|---|---|---|---|---|---|---|---|---|---|---|---|
| **AA** | **NH** | **α** | **β** | **δ1** | **δ2** | **ε2** | **ζ** | **ε1** | **ζ2** | **η2** | **ζ3** | **ε3** | **γ** |
| **Ala1** | 8.09 | 4.31 | 1.16 | - | - | - | - | - | - | - | - | - | - |
| ***m-I*-Phe** | 7.60 | 4.60 | 2.93 | 7.29 | 7.16 | 7.37 | 7.33 | - | - | - | - | - | - |
| **Val** | 8.09 | 4.07 | 1.75 | - | - | - | - | - | - | - | - | - | 0.72 |
| **Trp** | 7.67 | 4.65 | 3.34/3.20 | - | - | - | - | 11.19 | 7.33 | 7.08 | 6.95 | 7.42 | - |
| **Ala2** | 7.56 | 4.24 | 0.99 | - | - | - | - | - | - | - | - | - | - |

**Supplementary Table 15 | $^{1}$H chemical shifts assignments of compound Ac-Ala-(Cyclo-*m)*-[Phe-Val-Trp]-Ala-OH (2j).**

| 2j ($^{13}$C) | δ (ppm) | | | | | | | | | | |
|---|---|---|---|---|---|---|---|---|---|---|---|
| **AA** | **α** | **β** | **δ1** | **δ2** | **ε2** | **ζ** | **ζ2** | **η2** | **ζ3** | **ε3** | **γ** |
| **Ala1** | 47.7 | 17.7 | - | - | - | - | - | - | - | - | - |
| ***m-I*-Phe** | 53.4 | 38.0 | 129.1 | 128.9 | 128.1 | 126.8 | - | - | - | - | - |
| **Val** | 57.4 | 30.1 | - | - | - | - | - | - | - | - | 18.5 |
| **Trp** | 52.0 | 26.2 | - | - | - | - | 110.5 | 120.9 | 118.0 | 119.2 | - |
| **Ala2** | 47.4 | 16.4 | - | - | - | - | - | - | - | - | - |

**Supplementary Table 16 | $^{13}$C chemical shifts assignments of compound Ac-Ala-(Cyclo-*m)*-[Phe-Val-Trp]-Ala-OH (2j).**

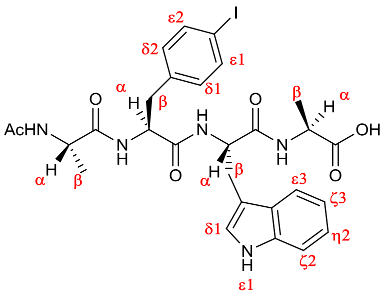


| 1k ($^1$H) | | | | | | δ (ppm) | | | | | |
|---|---|---|---|---|---|---|---|---|---|---|---|
| **AA** | **NH** | **α** | **β** | **δ1** | **δ2** | **ε2** | **ε1** | **ζ2** | **η2** | **ζ3** | **ε3** |
| **Ala1** | 7.96 | 4.19 | 1.07 | - | - | - | - | - | - | - | - |
| ***p-I*-Phe** | 7.83 | 4.40 | 2.90/2.70 | 6.93 | 6.93 | 7.51 | 7.51 | - | - | - | - |
| **Trp** | 8.02 | 4.56 | 3.13/2.95 | 7.16 | - | - | 10.81 | 7.32 | 7.05 | 6.97 | 7.60 |
| **Ala2** | 8.25 | 4.23 | 1.27 | - | - | - | - | - | - | - | - |

**Supplementary Table 17 | $^1$H chemical shifts assignments of compound Ac-Ala-*p*-I-Phe-Trp-Ala-OH (1k).**

| 1k($^{13}$C) | | | | | | δ (ppm) | | | | |
|---|---|---|---|---|---|---|---|---|---|---|
| **AA** | **α** | **β** | **δ1** | **δ2** | **ε2** | **ε1** | **ζ2** | **η2** | **ζ3** | **ε3** |
| **Ala1** | 47.8 | 17.6 | - | - | - | - | - | - | - | - |
| ***p-I*-Phe** | 53.2 | 36.6 | 131.4 | 131.4 | 136.3 | 136.3 | - | - | - | - |
| **Trp** | 52.8 | 27.5 | 123.3 | - | - | - | 111.0 | 120.6 | 117.9 | 118.2 |
| **Ala2** | 47.4 | 17.0 | - | - | - | - | - | - | - | - |

**Supplementary Table 18 | $^{13}$C chemical shifts assignments of compound Ac-Ala-*p*-I-Phe-Trp-Ala-OH (1k).**

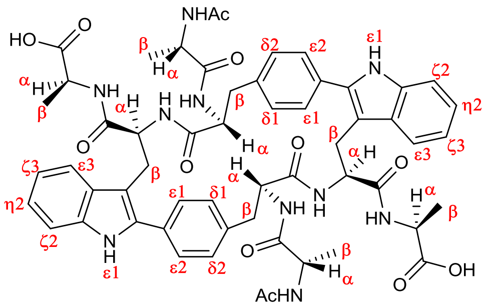


| 2k ($^1$H) | | | | | | δ (ppm) | | | | | |
|---|---|---|---|---|---|---|---|---|---|---|---|
| **AA** | **NH** | **α** | **β** | **δ1** | **δ2** | **ε2** | **ε1** | **ζ2** | **η2** | **ζ3** | **ε3** |
| **Ala1** | 7.93 | 4.18 | 1.08 | - | - | - | - | - | - | - | - |
| ***p-I*-Phe** | 8.13 | 3.96 | 2.64/2.48 | 7.25 | 7.25 | 7.61 | 7.61 | - | - | - | - |
| **Trp** | 6.82 | 4.07 | 3.43/3.43 | - | - | - | 11.20 | 7.34 | 7.11 | 7.03 | 7.67 |
| **Ala2** | 7.90 | 4.21 | 1.28 | - | - | - | - | - | - | - | - |

**Supplementary Table 19 | $^1$H chemical shifts assignments of compound Cyclo-*p,p)*bis-[Phe-Trp]-(Ac-Ala-Phe-Trp-Ala-OH) (2k).**

| 2k($^{13}$C) | | | | | | δ (ppm) | | | | |
|---|---|---|---|---|---|---|---|---|---|---|
| **AA** | **α** | **β** | **δ1** | **δ2** | **ε2** | **ε1** | **ζ2** | **η2** | **ζ3** | **ε3** |
| **Ala1** | 47.7 | 17.8 | - | - | - | - | - | - | - | - |
| ***p-I*-Phe** | 52.9 | 35.2 | 128.8 | 128.8 | 127.9 | 127.9 | - | - | - | - |
| **Trp** | 54.9 | 25.55 | - | - | - | - | 110.9 | 121.4 | 118.5 | 118.3 |
| **Ala2** | 47.5 | 17.0 | - | - | - | - | - | - | - | - |

**Supplementary Table 20 | $^{13}$C chemical shifts assignments of compound Cyclo-*p,p)*bis-[Phe-Trp]-(Ac-Ala-Phe-Trp-Ala-OH) (2k).** Due to the symmetric nature of peptide **2k'**, both tetrapeptide moieties ($A^1$-F-W-$A^2$) are equivalent and have identical NMR signals.

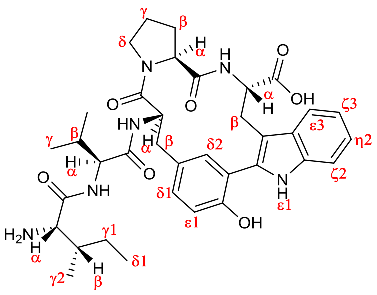


| 2l ($^1$H) | | | | | | | | | | | | |
|---|---|---|---|---|---|---|---|---|---|---|---|---|
| **AA** | **NH** | **α** | **β** | **δ1** | **δ2** | **ε1** | **ζ2** | **η2** | **ζ3** | **ε3** | **γ1** | **γ2** |
| **Ile** | 8.22 | 3.31 | 1.75 | 0.85 | - | - | - | - | - | - | 1.43/1.11 | 0.85 |
| **Val** | 8.15 | 4.33 | 1.97 | - | - | - | - | - | - | - | 0.85 | - |
| ***m-l*-Tyr** | 8.32 | 4.68 | 3.07/2.89 | 7.11 | 7.14 | 6.89 | - | - | - | - | - | - |
| **Pro** | - | 4.49 | 2.12/1.56 | 3.61/3.06 | - | - | - | - | - | - | 1.72/1.47 | - |
| **Trp** | 7.60 | 4.53 | 3.26/2.77 | - | - | 10.79 | 7.32 | 7.06 | 6.99 | 7.60 | - | - |

**Supplementary Table 21 | $^1$H chemical shifts assignments of compound H-Ile-Val-(Cyclo-*m*)-[Tyr-Pro-Trp]-OH (2l).**

| 2l ($^{13}$C) | | | | | | | | | | | |
|---|---|---|---|---|---|---|---|---|---|---|---|
| **AA** | **α** | **β** | **δ1** | **δ2** | **ε1** | **ζ2** | **η2** | **ζ3** | **ε3** | **γ1** | **γ2** |
| **Ile** | 58.1 | 37.3 | - | - | - | - | - | - | - | 23.3 | - |
| **Val** | 56.7 | 30.7 | - | - | - | - | - | - | - | - | - |
| ***m-l*-Tyr** | 51.5 | 35.9 | 130.3 | 131.2 | 115.6 | - | - | - | - | - | - |
| **Pro** | 59.5 | 25.6 | 46.6 | - | - | - | - | - | - | 24.1 | - |
| **Trp** | 54.1 | 28.5 | - | - | - | 110.7 | 120.5 | 117.9 | 117.8 | - | - |

**Supplementary Table 22 | $^{13}$C chemical shifts assignments of compound H-Ile-Val-(Cyclo-*m*)-[Tyr-Pro-Trp]-OH (2l).** Based on NOE interactions and $^{13}$C chemical shift differences of Pro, it was assigned a *trans* type configuration to this amino acid ($^{13}$C $\Delta\delta_{\beta\text{-}\alpha}$ = 1.6 and $^1H_{\alpha}$ – $^1H_{\delta}$ NOE correlation identified).[6,7]

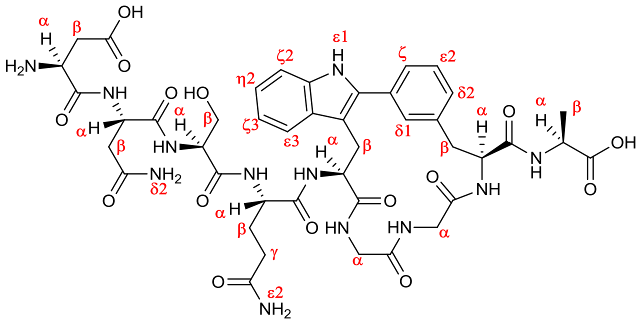

| 2m ($^{1}$H) | | | | | | δ (ppm) | | | | | | |
|---|---|---|---|---|---|---|---|---|---|---|---|---|
| **AA** | **NH** | **α** | **β** | **δ1** | **δ2** | **ε2** | **ε1** | **ζ/ ζ2** | **η2** | **ζ3** | **ε3** | **γ1** |
| **Asp** | - | 3.85 | 2.59 | - | - | - | - | - | - | - | - | - |
| **Asn** | 8.58 | 4.67 | 2.65/2.50 | - | 7.38/6.93 | - | - | - | - | - | - | - |
| **Ser** | 8.23 | 4.18 | 3.71/3.58 | - | - | - | - | - | - | - | - | - |
| **Gln** | 8.01 | 4.27 | 1.91/1.78 | - | - | 7.30/6.74 | - | - | - | - | - | 2.12/2.06 |
| **Trp** | 8.15 | 4.83 | 3.45/3.01 | - | - | - | 11.16 | 7.86 | 7.05 | 7.11 | 7.35 | - |
| **Gly** | 8.15 | 3.79/3.24 | - | - | - | - | - | - | - | - | - | - |
| **Gly** | 8.58 | 3.62/3.32 | - | - | - | - | - | - | - | - | - | - |
| ***m-I*-Phe** | 6.86 | 4.57 | 3.15/2.83 | 7.36 | 7.86 | 7.38 | - | 7.41 | - | - | - | - |
| **Ala** | 8.50 | 4.22 | 1.34 | - | - | - | - | - | - | - | - | - |

**Supplementary Table 23 | $^{1}$H chemical shifts assignments of compound H-Asp-Asn-Ser-Gln-(Cyclo-*m*)-[Trp-Gly-Gly-Phe]-Ala-OH (2m).**

| 2m ($^{13}$C) | | | | | | δ (ppm) | | | | | |
|---|---|---|---|---|---|---|---|---|---|---|---|
| **AA** | **α** | **β** | **δ1** | **δ2** | **ε2** | **ε1** | **ζ/ ζ2** | **η2** | **ζ3** | **ε3** | **γ1** |
| **Asp** | 50.1 | 37.3 | - | - | - | - | - | - | - | - | - |
| **Asn** | 49.8 | 36.5 | - | - | - | - | - | - | - | - | - |
| **Ser** | 56.0 | 61.2 | - | - | - | - | - | - | - | - | - |
| **Gln** | 52.3 | 27.5 | - | - | - | - | - | - | - | - | 31.4 |
| **Trp** | 52.7 | 27.7 | - | - | - | - | 118.9 | 118.3 | 121.1 | 110.7 | - |
| **Gly** | 42.7 | - | - | - | - | - | - | - | - | - | - |
| **Gly** | 42.1 | - | - | - | - | - | - | - | - | - | - |
| ***m-I*-Phe** | 53.8 | 37.5 | 127.2 | 130.1 | 127.9 | - | 125.2 | - | - | - | - |
| **Ala** | 47.5 | 16.76 | - | - | - | - | - | - | - | - | - |

**Supplementary Table 24 | $^{13}$C chemical shifts assignments of compound H-Asp-Asn-Ser-Gln-(Cyclo-*m*)-[Trp-Gly-Gly-Phe]-Ala-OH (2m).**

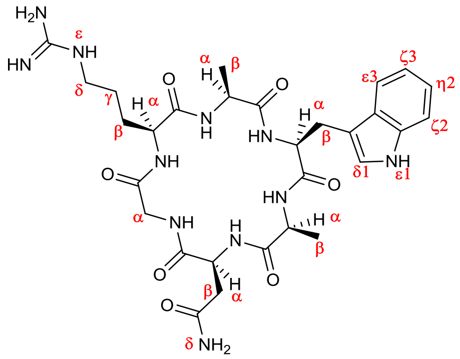

| 3 ($^1$H) | | | | δ (ppm) | | | | | | |
|---|---|---|---|---|---|---|---|---|---|---|
| **AA** | **NH** | **α** | **β** | **δ1** | **ε1** | **ζ2** | **η2** | **ζ3** | **ε3** | **γ** |
| **Trp** | 7.88 | 4.28 | 3.18 | 7.14 | 10.83 | 7.32 | 7.06 | 6.97 | 7.58 | - |
| **Ala1** | 8.09 | 3.97 | 1.21 | - | - | - | - | - | - | - |
| **Asn** | 8.14 | 4.35 | 2.61 | 7.42/6.92 | - | - | - | - | - | - |
| **Gly** | 8.25 | 3.84/3.55 | - | - | - | - | - | - | - | - |
| **Arg** | 8.06 | 4.12 | 1.87/1.60 | 3.08 | 7.47 | - | - | - | 7.47 | 1.47 |
| **Ala2** | 7.99 | 4.08 | 1.21 | - | - | - | - | - | - | - |

**Supplementary Table 25 | $^1$H chemical shifts assignments of compound Cyclo(-Arg-Ala-Trp-Ala-Asn-Gly-) (3).**

| 3 ($^{13}$C) | | | δ (ppm) | | | | | |
|---|---|---|---|---|---|---|---|---|
| **AA** | **α** | **β** | **δ1** | **ζ2** | **η2** | **ζ3** | **ε3** | **γ** |
| **Trp** | 54.3 | 26.6 | 123.5 | 111.0 | 120.6 | 117.9 | 118.1 | - |
| **Ala1** | 49.3 | 17.0 | - | - | - | - | - | - |
| **Asn** | 50.0 | 35.6 | - | - | - | - | - | - |
| **Gly** | 42.6 | - | - | - | - | - | - | - |
| **Arg** | 52.7 | 27.7 | 40.1 | - | - | - | - | 24.8 |
| **Ala2** | 49.2 | 17.0 | - | - | - | - | - | - |

**Supplementary Table 26 | $^{13}$C chemical shifts assignments of compound Cyclo(-Arg-Ala-Trp-Ala-Asn-Gly-) (3).**

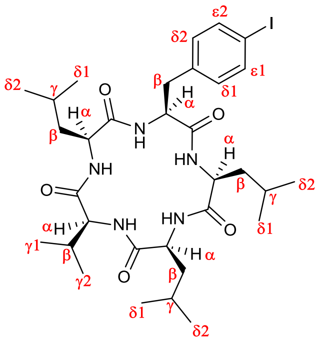

| 4 ($^1$H) | | | δ (ppm) | | | |
|---|---|---|---|---|---|---|
| **AA** | **NH** | **α** | **β** | **γ** | **δ** | **ε** |
| **Leu1** | 8.15 | 3.94 | 1.52/1.35 | 1.28 | 0.83 | - |
| ***p-I*-Phe** | 8.14 | 4.27 | 3.02 | - | 7.03 | 7.62 |
| **Leu2** | 7.98 | 4.05 | 1.72/1.48 | 1.33 | 0.83 | - |
| **Leu3** | 7.95 | 4.12 | 1.68/1.55 | 1.53 | 0.83 | - |
| **Val** | 8.02 | 3.66 | 2.15 | 0.83 | - | - |

**Supplementary Table 27 | $^1$H chemical shifts assignments of compound Cyclo(-Leu-Leu-Val-Leu-*p*-I-Phe-) (4).**

| 4 ($^{13}$C) | | | δ (ppm) | | |
|---|---|---|---|---|---|
| **AA** | **α** | **β** | **γ** | **δ** | **ε** |
| **Leu1** | 53.0 | 39.5 | 24.1 | - | - |
| ***p-I*-Phe** | 55.3 | 35.4 | - | 131.3 | 136.7 |
| **Leu2** | 53.2 | 39.2 | 24.1 | - | - |
| **Leu3** | 53.4 | 39.7 | 24.4 | - | - |
| **Val** | 60.9 | 29.0 | - | - | - |

**Supplementary Table 28 | $^{13}$C chemical shifts assignments of compound Cyclo(-Leu-Leu-Val-Leu-*p*-I-Phe-) (4).**

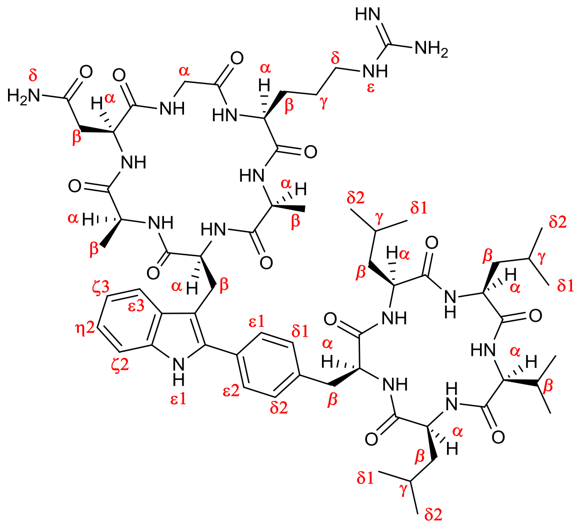


| 5 ($^{1}$H) | | | | δ (ppm) | | | | | | | | |
|---|---|---|---|---|---|---|---|---|---|---|---|---|
| **AA** | **NH** | **α** | **β** | **δ1** | **δ2** | **ε2** | **ε1** | **ζ2** | **η2** | **ζ3** | **ε3** | **γ** |
| **Trp** | 8.07 | 4.25 | 3.40/3.32 | - | - | - | 11.16 | 7.33 | 7.08 | 6.98 | 7.59 | - |
| **Ala1** | 8.00 | 3.92 | 1.11 | - | - | - | - | - | - | - | - | - |
| **Asn** | 8.04 | 4.35 | 2.58 | 7.42/6.91 | - | - | - | - | - | - | - | - |
| **Gly** | 8.45 | 3.86/3.41 | - | - | - | - | - | - | - | - | - | - |
| **Arg** | 7.98 | 4.15 | 1.79/1.59 | 3.08 | - | - | 7.42 | - | - | - | - | 1.47 |
| **Ala2** | 7.94 | 4.02 | 1.11 | - | - | - | - | - | - | - | - | - |
| **Leu1** | 8.18 | 3.97 | 1.63 | 0.85 | 0.85 | - | - | - | - | - | - | 1.37 |
| ***p-I*-Phe** | 8.19 | 4.33 | 3.12 | 7.33 | 7.33 | 7.64 | 7.64 | - | - | - | - | - |
| **Leu2** | 7.98 | 4.13 | 1.56 | 0.85 | 0.85 | - | - | - | - | - | - | 1.47 |
| **Leu3** | 7.98 | 4.13 | 1.71 | 0.85 | 0.85 | - | - | - | - | - | - | 1.54 |
| **Val** | 8.00 | 3.72 | 2.15 | - | - | - | - | - | - | - | - | 0.85 |

**Supplementary Table 29 | $^{1}$H chemical shifts assignments of compound Cyclo(Ala-Asn-Gly-Arg-Ala-C2-Trp-)—Cyclo(C4-Phe-Leu-Leu-Val-Leu-) (5).**

| 5 ($^{13}$C) | | | | δ (ppm) | | | | | | | |
|---|---|---|---|---|---|---|---|---|---|---|---|
| **AA** | **α** | **β** | **δ1** | **δ2** | **ε2** | **ε1** | **ζ2** | **η2** | **ζ3** | **ε3** | **γ** |
| **Trp** | 55.5 | 26.2 | - | - | - | - | 110.7 | 121.2 | 118.3 | 118.8 | - |
| **Ala1** | 48.8 | 17.0 | - | - | - | - | - | - | - | - | - |
| **Asn** | 50.0 | 35.7 | - | - | - | - | - | - | - | - | - |
| **Gly** | 43.0 | - | - | - | - | - | - | - | - | - | - |
| **Arg** | 52.6 | 28.1 | 40.1 | - | - | - | - | - | - | - | 24.9 |
| **Ala2** | 48.8 | 17.0 | - | - | - | - | - | - | - | - | - |
| **Leu1** | 52.9 | 39.4 | - | - | - | - | - | - | - | - | 24.2 |
| ***p-I*-Phe** | 55.9 | 35.9 | 129.0 | 129.0 | 127.1 | 127.1 | - | - | - | - | - |
| **Leu2** | 53.5 | 39.6 | - | - | - | - | - | - | - | - | 24.1 |
| **Leu3** | 53.5 | 39.5 | - | - | - | - | - | - | - | - | 24.4 |
| **Val** | 60.7 | 29.2 | - | - | - | - | - | - | - | - | - |

**Supplementary Table 30 | $^{13}$C chemical shifts assignments of compound Cyclo(Ala-Asn-Gly-Arg-Ala-C2-Trp-)—Cyclo(C4-Phe-Leu-Leu-Val-Leu-) (5).**

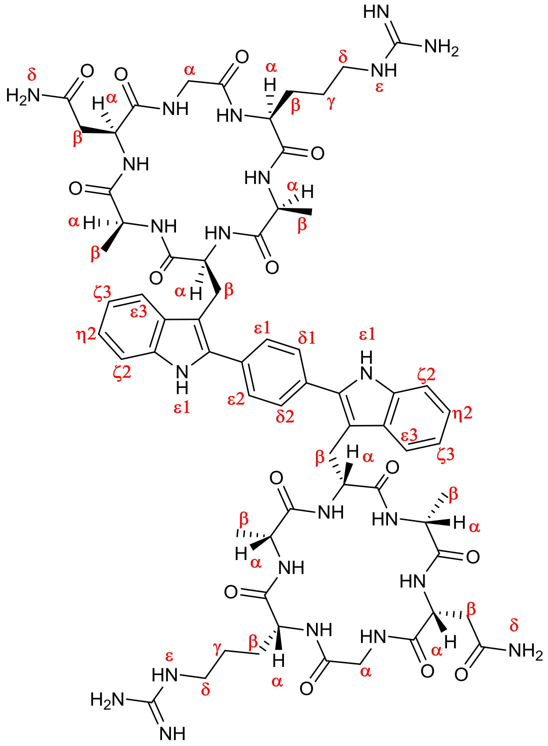


| 6 ($^{1}$H) | | | | | | δ (ppm) | | | | | | |
|---|---|---|---|---|---|---|---|---|---|---|---|---|
| **AA** | **NH** | **α** | **β** | **δ1** | **δ2** | **ε2** | **ε1** | **ζ2** | **η2** | **ζ3** | **ε3** | **γ** |
| **Trp** | 8.17 | 4.36 | 3.51/3.40 | - | - | - | 11.29 | 7.36 | 7.11 | 7.00 | 7.65 | - |
| **Ala1** | 8.06 | 4.04 | 1.11 | - | - | - | - | - | - | - | - | - |
| **Asn** | 8.06 | 4.36 | 2.63-2.56 | 7.42/6.89 | - | - | - | - | - | - | - | - |
| **Gly** | 8.40 | 3.86/3.42 | - | - | - | - | - | - | - | - | - | - |
| **Arg** | 7.97 | 4.18 | 1.81/1.62 | 3.08 | - | - | - | - | - | - | - | 1.47 |
| **Ala2** | 8.06 | 3.98 | 1.11 | - | - | - | - | - | - | - | - | - |
| **Ph** | - | - | - | 7.87 | 7.87 | 7.87 | 7.87 | - | - | - | - | - |

**Supplementary Table 31 | $^{1}$H chemical shifts assignments of compound Bis[cyclo(-Arg-Ala-Trp-Ala-Asn-Gly-)] adduct (6).**

| 6 ($^{13}$C) | | | | | | δ (ppm) | | | | | |
|---|---|---|---|---|---|---|---|---|---|---|---|
| **AA** | **α** | **β** | **δ1** | **δ2** | **ε2** | **ε1** | **ζ2** | **η2** | **ζ3** | **ε3** | **γ** |
| **Trp** | 55.4 | 26.3 | - | - | - | - | 110.7 | 121.3 | 118.3 | 118.1 | - |
| **Ala1** | 49.0 | 17.0 | - | - | - | - | - | - | - | - | - |
| **Asn** | 50.1 | 35.8 | - | - | - | - | - | - | - | - | - |
| **Gly** | 42.8 | - | - | - | - | - | - | - | - | - | - |
| **Arg** | 52.2 | 28.0 | 39.9 | - | - | - | - | - | - | - | 24.6 |
| **Ala2** | 48.8 | 17.0 | - | - | - | - | - | - | - | - | - |
| **Ph** | - | - | 127.3 | 127.3 | 127.3 | 127.3 | - | - | - | - | - |

**Supplementary Table 32 | $^{13}$C chemical shifts assignments of compound Bis[cyclo(-Arg-Ala-Trp-Ala-Asn-Gly-)] adduct (6).**

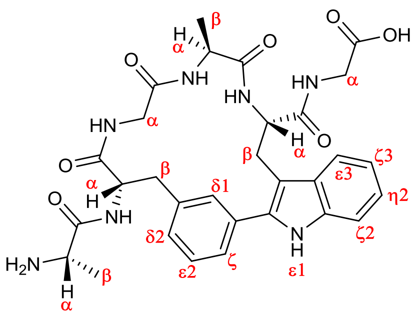


| 9 ($^{1}$H) | | | | δ (ppm) | | | | | | | | |
|---|---|---|---|---|---|---|---|---|---|---|---|---|
| **AA** | **NH** | **α** | **β** | **δ1** | **δ2** | **ε2** | **ζ** | **ε1** | **ζ2** | **η2** | **ζ3** | **ε3** |
| **Ala1** | - | 3.71 | 1.28 | - | - | - | - | - | - | - | - | - |
| ***m-l*-Phe** | 9.14 | 4.79 | 3.14/3.03 | 7.50 | 7.14 | 7.35 | 7.40 | - | - | - | - | - |
| **Gly1** | 8.43 | 3.76/3.57 | - | - | - | - | - | - | - | - | - | - |
| **Ala2** | 7.84 | 3.83 | 0.80 | - | - | - | - | - | - | - | - | - |
| **Trp** | 6.80 | 4.55 | 3.56/3.32 | - | - | - | - | 11.15 | 7.34 | 7.11 | 7.01 | 7.59 |
| **Gly2** | 7.38 | 3.55/3.38 | - | - | - | - | - | - | - | - | - | - |

**Supplementary Table 33 | $^{1}$H chemical shifts assignments of compound H-Ala-(Cyclo-*m*)-[Phe-Gly-Ala-Trp]-Gly-OH (9).**

| 9 ($^{13}$C) | | | | δ (ppm) | | | | | | |
|---|---|---|---|---|---|---|---|---|---|---|
| **AA** | **α** | **β** | **δ1** | **δ2** | **ε2** | **ζ** | **ζ2** | **η2** | **ζ3** | **ε3** |
| **Ala1** | 49.2 | 18.3 | - | - | - | - | - | - | - | - |
| ***m-l*-Phe** | 52.7 | 36.8 | 129.0 | 128.9 | 128.0 | 128.7 | - | - | - | - |
| **Gly1** | 43.1 | - | - | - | - | - | - | - | - | - |
| **Ala2** | 49.3 | 15.8 | - | - | - | - | - | - | - | - |
| **Trp** | 52.4 | 26.6 | - | - | - | - | 110.9 | 121.3 | 118.5 | 118.2 |
| **Gly2** | 42.1 | - | - | - | - | - | - | - | - | - |

**Supplementary Table 34 | $^{13}$C chemical shifts assignments of compound H-Ala-(Cyclo-*m*)-[Phe-Gly-Ala-Trp]-Gly-OH (9).**

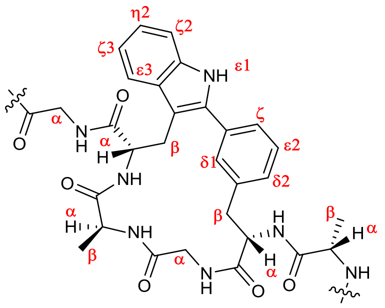

| 10 ($^{1}$H) | | | | | | | δ (ppm) | | | | | |
|---|---|---|---|---|---|---|---|---|---|---|---|---|
| **AA** | **NH** | **α** | **β** | **δ1** | **δ2** | **ε2** | **ζ** | **ε1** | **ζ2** | **η2** | **ζ3** | **ε3** |
| **Ala1** | 8.53 | 4.14 | 1.28 | - | - | - | - | - | - | - | - | - |
| ***m-I*-Phe** | 6.77 | 4.63 | 3.57/2.83 | 7.42 | 7.17 | 7.38 | 7.44 | - | - | - | - | - |
| **Gly1** | 7.39 | 4.09/3.15 | - | - | - | - | - | - | - | - | - | - |
| **Ala2** | 8.19 | 3.47 | 0.34 | - | - | - | - | - | - | - | - | - |
| **Trp** | 6.46 | 4.55 | 3.50 | - | - | - | - | 11.2 | 7.32 | 7.06 | 6.96 | 7.41 |
| **Gly2** | 7.38 | 3.54/3.35 | - | - | - | - | - | - | - | - | - | - |

**Supplementary Table 35 | $^{1}$H chemical shifts assignments of compound Cyclo[-Ala-(Cyclo-*m*)-[Phe-Gly-Ala-Trp]-Gly-] (10).**

| 10 ($^{13}$C) | | | | | | δ (ppm) | | | | |
|---|---|---|---|---|---|---|---|---|---|---|
| **AA** | **α** | **β** | **δ1** | **δ2** | **ε2** | **ζ** | **ζ2** | **η2** | **ζ3** | **ε3** |
| **Ala1** | 49.2 | 16.2 | - | - | - | - | - | - | - | - |
| ***m-I*-Phe** | 51.0 | 35.6 | 129.9 | 129.8 | 128.0 | 126.5 | - | - | - | - |
| **Gly1** | 42.9 | - | - | - | - | - | - | - | - | - |
| **Ala2** | 50.9 | 15.0 | - | - | - | - | - | - | - | - |
| **Trp** | 48.5 | 24.8 | - | - | - | - | 110.8 | 121.0 | 118.2 | 118.2 |
| **Gly2** | 43.1 | - | - | - | - | - | - | - | - | - |

**Supplementary Table 36 | $^{13}$C chemical shifts assignments of compound Cyclo[-Ala-(Cyclo-*m*)-[Phe-Gly-Ala-Trp]-Gly-] (10).**

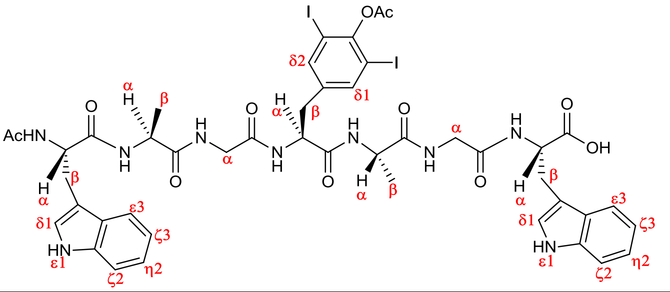


| 11 ($^{1}$H) | | | | δ (ppm) | | | | | | |
|---|---|---|---|---|---|---|---|---|---|---|
| **AA** | **NH** | **α** | **β** | **δ1** | **δ2** | **ε1** | **ζ2** | **η2** | **ζ3** | **ε3** |
| **Trp1** | 8.01 | 4.53 | 3.11/2.89 | 7.12 | - | 10.75 | 7.29 | 7.05 | 6.94 | 7.52 |
| **Ala1** | 8.13 | 4.24 | 1.20 | - | - | - | - | - | - | - |
| **Gly1** | 7.92 | 3.70/3.56 | - | - | - | - | - | - | - | - |
| **Tyr** | 8.07 | 4.56-4.45* | 2.97/2.67 | 7.77 | 7.77 | - | - | - | - | - |
| **Ala2** | 8.30 | 4.27 | 1.21 | - | - | - | - | - | - | - |
| **Gly2** | 8.08 | 3.73 | - | - | - | - | - | - | - | - |
| **Trp2** | 8.06 | 4.48 | 3.18/3.04 | 7.14 | - | 10.84 | 7.32 | 7.07 | 6.97 | 7.59 |

**Supplementary Table 37 | $^{1}$H chemical shifts assignments of compound Ac-Trp-Ala-Gly-*3,5*-I,I-Tyr(OAc)-Ala-Gly-Trp-OH (11).**

| 11 ($^{13}$C) | | | | δ (ppm) | | | | |
|---|---|---|---|---|---|---|---|---|
| **AA** | **α** | **β** | **δ1** | **δ2** | **ζ2** | **η2** | **ζ3** | **ε3** |
| **Trp1** | 53.1 | 27.4 | 123.4 | - | 110.9 | 120.6 | 117.9 | 117.8 |
| **Ala1** | 48.1 | 17.9 | - | - | - | - | - | - |
| **Gly1** | 41.4 | - | - | - | - | - | - | - |
| **Tyr** | 53.1 | 35.7 | 139.4 | 139.4 | - | - | - | - |
| **Ala2** | 48.1 | 17.9 | - | - | - | - | - | - |
| **Gly2** | 41.5 | - | - | - | - | - | - | - |
| **Trp2** | 52.8 | 27.1 | 123.4 | - | 110.9 | 120.6 | 117.9 | 118.4 |

**Supplementary Table 38 | $^{13}$C chemical shifts assignments of compound Ac-Trp-Ala-Gly-*3,5*-I,I-Tyr(OAc)-Ala-Gly-Trp-OH (11).**

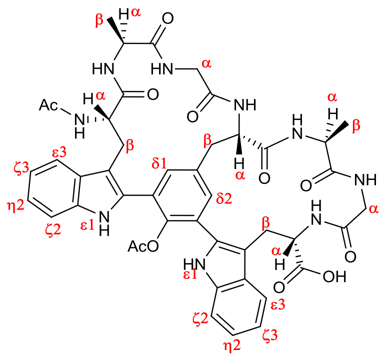


| 12 ($^1$H) | | | | | δ (ppm) | | | | | |
|---|---|---|---|---|---|---|---|---|---|---|
| **AA** | **NH** | **α** | **β** | **δ1** | **δ2** | **ε1** | **ζ2** | **η2** | **ζ3** | **ε3** |
| **Trp1** | 8.03 | 4.94 | 3.04 | - | - | 10.87 | 7.34 | 7.09 | 7.03 | 7.98 |
| **Ala1** | 6.79 | 4.19 | 1.03 | - | - | - | - | - | - | - |
| **Gly1** | 8.50 | 3.90/3.47 | - | - | - | - | - | - | - | - |
| **Tyr** | 7.22 | 4.75 | 3.30/3.19 | 7.43 | 7.36 | - | - | - | - | - |
| **Ala2** | 8.71 | 4.39 | 1.21 | - | - | - | - | - | - | - |
| **Gly2** | 8.24 | 3.58/3.24 | - | - | - | - | - | - | - | - |
| **Trp2** | 6.79 | 5.04 | 3.38/2.83 | - | - | 10.87 | 7.25 | 7.03 | 6.96 | 7.58 |

**Supplementary Table 39 | $^1$H chemical shifts assignments of compound Ac-(bicyclo-*m,m*)-[Trp-Ala-Gly-Tyr(OAc)]-[Tyr(OAc)-Ala-Gly-Trp]-OH (12).**

| 12 ($^{13}$C) | | | | δ (ppm) | | | | |
|---|---|---|---|---|---|---|---|---|
| **AA** | **α** | **β** | **δ1** | **δ2** | **ζ2** | **η2** | **ζ3** | **ε3** |
| **Trp1** | 51.5 | 29.2 | - | - | 111.0 | 120.9 | 118.1 | 119.2 |
| **Ala1** | 47.8 | 18.2 | - | - | - | - | - | - |
| **Gly1** | 42.4 | - | - | - | - | - | - | - |
| **Tyr** | 51.2 | 36.6 | 132.8 | 133.4 | - | - | - | - |
| **Ala2** | 47.6 | 18.4 | - | - | - | - | - | - |
| **Gly2** | 42.4 | - | - | - | - | - | - | - |
| **Trp2** | 50.6 | 26.7 | - | - | 110.8 | 120.7 | 118.0 | 118.7 |

**Supplementary Table 40 | $^{13}$C chemical shifts assignments of compound Ac-(bicyclo-*m,m*)-[Trp-Ala-Gly-Tyr(OAc)]-[Tyr(OAc)-Ala-Gly-Trp]-OH (12).**

# Supplementary Methods

## 1. Abbreviations

Abbreviation used for amino acids and designations of peptides follow the rules of the IUPAC-IUB Commission of Biochemical Nomenclature in *J. Biol. Chem.* 247, 977-983 (1982). The following additional abbreviations are used: ACN: acetonitrile, DMF: *N,N*-dimethylformamide, DCM: dichloromethane, Fmoc: 9*H*-fluorenylmethyloxycarbonyl, TFA: trifluoroacetic acid, PBS: phosphate buffered saline, SPPS: solid phase peptide synthesis, DIEA: *N,N*-diisopropylethylamine, DIPCDI: *N,N*-diisopropylcarbodiimide, HOBt: hydroxybenzotriazole, HBTU: *o*-benzotriazole-*N,N,N',N'*-tetramethyl-uronium-hexafluoro-phosphate, TIS: triisopropylsilane, PyBOP: (benzotriazol-1-yloxy)tripyrrolidinophosphonium hexafluorophosphate, PyAOP: (7-azabenzotriazol-1-yloxy)tripyrrolidinophosphonium hexafluorophosphate, TBTU: *o*-(benzotriazol-1-yl)-*N,N,N′,N′*-tetramethyluronium tetrafluoroborate, TFE: 2,2,2-trifluoroethanol, PivOH: pivalic acid, DMAP: 4-(dimethylamino)pyridine, Trt: trityl, Pbf: 2,2,4,6,7-pentamethyldihydrobenzofuran-5-sulfonyl, AB linker: 2-(4-hidroxymethylphenoxy)-propionic acid, IR: infrared spectroscopy, HPLC-MS: high performance liquid chromatography mass spectrometry, HRMS(ESI): high-resolution mass spectrometry (electrospray ionization), RP-HPLC; reversed phase-high performance liquid chromatography, NMR: nuclear magnetic resonance. HUVEC: human umbilica vein endothelial cell, Fb: fibrinogen, Vn: vitronectin.

## 2. General experimental information

Reactions were monitored by HPLC-MS at 220 nm using a HPLC Waters Alliance HT comprising a pump (Edwards RV12) with degasser, an autosampler and a diode array detector. Flow from the column was split to a MS spectrometer. The MS detector was configured with an eletrospray ionization source (micromass ZQ4000) and nitrogen was used as the nebulizer gas. Data acquisition was performed with MassLynx software. For compounds **2a-2c, 2l, 2m, 1j-BODIPY**, **5**, **6**, and **16**, yields are estimated from the integration of the peak areas in the HPLC-MS crude. Other yields are for the isolated pure compound. All microwave reactions were carried out in 10 mL sealed glass tubes in a focused mono-mode microwave oven ("Discover" by CEM Corporation) featured with a surface sensor for internal temperature determination. Cooling was provided by compressed air ventilating the microwave chamber during the reaction. When stated, the final crude was purified via flash column chromatography Combi Flash ISCO RF provided with dual UV detection.

NMR spectra of peptides in DMSO-$d_6$ were acquired with either a Bruker DMX-500 MHz spectrometer or Bruker Avance III 600 MHz and Bruker Avance 800 MHz spectrometers equipped with TCI cryoprobes. The spectra were referenced relative to the residual DMSO signal ($^1$H, 2.49 ppm; $^{13}$C, 39.5 ppm). $^1$H resonances were unequivocally assigned by two-dimensional NMR

experiments (COSY, TOCSY and NOESY and/or ROESY). Then, the $^{13}C$ resonances were straightforwardly assigned on the basis of the cross-correlations observed in the $^{1}H$-$^{13}C$ HSQC spectra. Mixing times for TOCSY spectra were 70 ms, for NOESY spectra 300-450 ms and for ROESY experiments were 200 ms. The temperature coefficients for the amide protons of each peptide were determined via $^{1}H$ spectra in the range 298-313 K with a step size of 5 K. Chemical shifts (δ) are reported in ppm. Multiplicities are referred by the following abbreviations: s = singlet, d = doublet, t = triplet, dd = double doublet, dt = double triplet, q = quartet, p = pentuplet and m = multiplet. HRMS (ESI positive) were obtained with a LTQ-FT Ultra (Thermo Scientific) mass Spectrometer. IR spectra were obtained on a Thermo Nicolet NEXUS.

CD Spectroscopy. Circular dichroism (CD) measurements were performed using a Jasco J-815 spectrophotometer. The spectra were recorded from 260 to 170 nm using a 1.0 mm path-length quartz cuvette at 2 nm bandwidth, 50 nm/min scan speed, 0.5 s response time, 0.2 nm data pitch and three accumulations. The background signal of the buffer alone was subtracted for each spectrum. CD spectra were converted from raw ellipticity (θ, mdeg) to mean molar ellipticity per residue ([θ], deg $cm^2$ $dmol^{-1}$).

All the samples were dissolved in a buffer of 25 mM $Na_2HPO_4$ (pH 7) at both 100 and 200 μM final peptide concentration. Additionally, new determinations were made in 10% of 2,2,2-trifluoroethanol (TFE) to increase the propensity to form secondary structures. To ensure no interference of peptide aromatic moiety on the spectra profiles, the previously reported 3-(2-Phenyl-1*H*-indol-3-yl)propanoic acid[1] was also analysed at identical conditions as for the tested compounds.

**General procedure for SPPS.**[2] All peptides were manually synthesized in polystyrene syringes fitted with a polyethylene porous disc using Fmoc-based SPPS. Solvents and soluble reagents were removed by suction. The Fmoc group was removed with piperidine-DMF (1:4) (1×1 min, 2×5 min). Peptide synthesis transformations and washes were performed at r.t.

*Resin loading (only for 2-Chlorotrityl resin).* Fmoc-XX-OH (1.0 eq.) was attached to the resin (1.0 eq.) with DIEA (3.0 eq.) in DCM at r.t for 10 min and then DIEA (7.0 eq.) for 40 min. The remaining trityl groups were capped adding 0.8 μL MeOH/mg resin for 10 minutes. After that, the resin was

filtered and washed with DCM (4 x 1 min), DMF (4 x 1 min). The loading of the resin was determined by titration of the Fmoc group.[2]

*Peptide elongation.* After the Fmoc group was eliminated, the resin was washed with DMF (4 x 1 min), DCM (3 x 1 min), DMF (4 x 1 min). The completion of the coupling was monitored with the ninhydrin (free primary amine) or chloranil (free secondary amine) tests.[3] Then, the resin was filtered and washed with DCM (4 x 1 min) and DMF (4 x 1 min) and the Fmoc group was eliminated.

*Acetylation.* When indicated, once the peptide was fully elongated, N-terminal acetylation was performed with acetic anhydride (10 eq.), DIEA (10 eq.) in DMF (30 min).

*Final cleavage.* The resin bound peptide was treated with the corresponding TFA cleavage cocktail. Then, the resin was washed with DCM and the combined eluates were evaporated under vacuum. Then, the residue was washed with $Et_2O$, dissolved in ACN:$H_2O$ and lyophilized furnishing the corresponding peptide.

## 3. Experimental procedures and peptide characterization

### Synthesis and peptide characterization of linear peptides 1a-1f

2-Chlorotrityl resin (1 mmol/g). Amino acid coupling. Fmoc-XX-OH (3.0 eq.) were incorporated with a 5-min pre-activation with DIPCDI (3.0 eq.) and HOBt (3.0 eq.) in DMF for 1h. The N-terminal was acetilated, and then the resin bound peptide was treated with a 5% (v/v) TFA/DCM solution (5 x 1 min).

**Ac-Ala-*m*-Phe-Ala-Trp-Ala-OH (1a). HRMS** (ESI) (m/z): [M] calcd. for $C_{31}H_{37}IN_6O_7$, 732.1768; $[M+H]^+$ found, 733.1836.

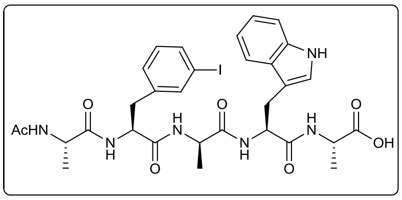


**Ac-Ala-*m*-Phe-Ala-Ala-Trp-Ala-OH (1b). HRMS** (ESI) (m/z): [M] calcd. for $C_{34}H_{42}IN_7O_8$, 803.2140; $[M+H]^+$ found, 804.2215.

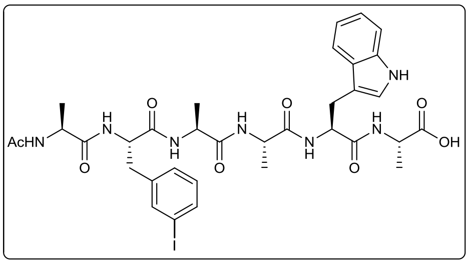

**Ac-Ala-*m*-Phe-Ala-Ala-Ala-Trp-Ala-OH (1c). HRMS** (ESI) (m/z): [M] calcd. for $C_{37}H_{47}IN_8O_9$, 874.2511; $[M+H]^+$ found, 875.2583.

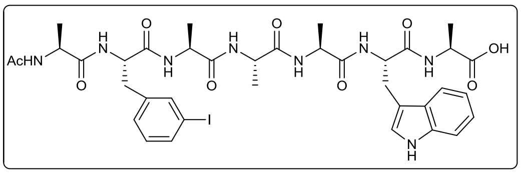

**Ac-Ala-*m*-Tyr(OAc)-Ala-Trp-Ala-OH (1d). HPLC-MS** (m/z): [M] calcd. for $C_{33}H_{39}IN_6O_9$, 790.18; $[M+H]^+$ found, 791.06.

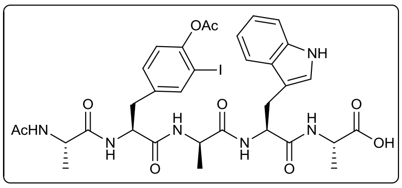

**Ac-Ala-*m*-Tyr(OAc)-Ala-Ala-Trp-Ala-OH (1e). HPLC-MS** (m/z): [M] calcd. for $C_{36}H_{44}IN_7O_{10}$, 861.22; $[M+H]^+$ found, 862.15.

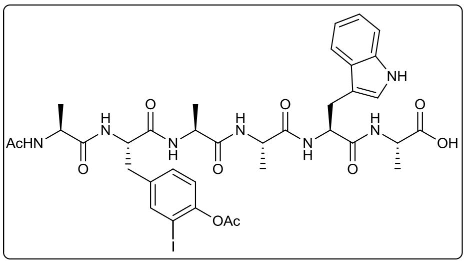

**Ac-Ala-*m*-Tyr(OAc)-Ala-Ala-Ala-Trp-Ala-OH (1f). HPLC-MS** (m/z): [M] calcd. for $C_{39}H_{49}IN_8O_{11}$, 932.26; $[M+H]^+$ found, 933.17.

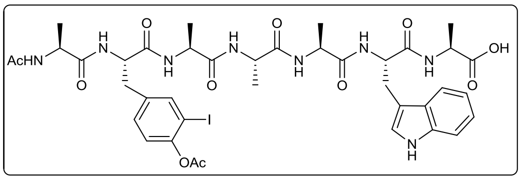

**Synthesis and peptide characterization of linear peptides 1g-1k**

**Ac-*m*-I-Phe-Asn-Gly-Arg-Trp-OH (1g).** 2-Chlorotrityl resin (0.94 mmol/g). Amino acid coupling. Fmoc-XX-OH (3.0 eq.) were incorporated with a 5-min pre-activation with DIPCDI (3.0 eq.) and OxymaPure (3.0 eq.) in DMF for 1h. Fmoc-XX-OH: Fmoc-Trp(Boc)-OH, Fmoc-Arg(Pbf)-OH, Fmoc-Gly-OH, Fmoc-Asn(Trt)-OH. Fmoc-*m*-I-Phe-OH (1.5 eq.) was incorporated with HBTU (1.5 eq.), HOBt (1.5 eq.) and DIEA (3.0 eq.) in DMF for 1h. The resin bound peptide was treated with a 95% TFA, 2.5% TIS, 2.5% $H_2O$ cocktail (1h). Pale solid (90-92% purity, estimated by HPLC-MS). **$^1$H NMR** (500 MHz, DMSO-$d_6$): δ 10.83 (m, 1H), 8.38 (d, $J$ = 7.7 Hz, 1H), 8.18 (d, $J$ = 8.2 Hz, 1H), 8.14 (d, $J$ = 7.5 Hz, 1H), 8.03 (t, $J$ = 5.7 Hz, 1H), 7.92 (d, $J$ = 8.2 Hz, 1H), 7.66 (d, $J$ = 1.7 Hz, 1H), 7.54 (d, $J$ = 7.9 Hz, 1H), 7.51 (d, $J$ = 7.9 Hz, 1H),

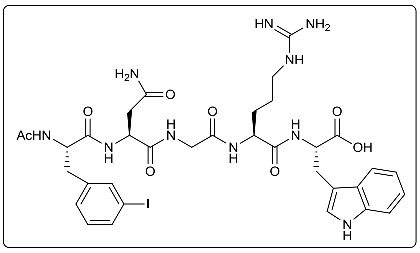

7.46 (m, 2H), 7.32 (d, $J$ = 8.1 Hz, 1H), 7.25 (d, $J$ = 7.7 Hz, 1H), 7.16 (d, $J$ = 2.4 Hz, 1H), 7.05 (t, $J$ = 7.7 Hz, 2H), 7.00 – 6.94 (m, 2H), 4.55 – 4.41 (m, 3H), 4.36 (td, $J$ = 8.4, 5.2 Hz, 1H), 3.76 (dd, $J$ = 16.9, 5.9 Hz, 1H), 3.66 (dd, $J$ = 16.8, 5.5 Hz, 1H), 3.17 (dd, $J$ = 14.7, 5.5 Hz, 1H), 3.07 (m, 3H), 2.98 (dd, $J$ = 13.7, 4.1 Hz, 1H), 2.66 (dd, $J$ = 13.7, 10.3 Hz, 1H), 2.57 (dd, $J$ = 15.5, 6.0 Hz, 1H), 2.50 (1H), 1.75 (s, 3H), 1.68 (m, 1H), 1.58 – 1.40 (m, 3H) ppm. **HPLC-MS** (m/z): $[M+H]^+$ calcd. for $C_{34}H_{43}IN_{10}O_8$, 847.7; found, 847.1.

**Ac-*m*-I-Phe-Arg-Gly-Asp-Trp-OH (1h).** H-Rink-Amide Chemmatrix resin (0.53 mmol/g). Amino acid coupling. Fmoc-XX-OH (3.0 eq.) were incorporated with a 5-min pre-activation with DIPCDI (3.0 eq.) and HOBt (3.0 eq.) in DMF for 1h (2h of coupling were carried out in the case of Fmoc-Trp(Boc)-OH). Fmoc-XX-OH: Fmoc-Trp(Boc)-OH, Fmoc-Asp(O*t*-Bu)-OH, Fmoc-Gly-OH, Fmoc-Arg(Pbf)-OH, Fmoc-*m*-I-Phe-OH. The N-terminal was acetilated, and then the resin bound peptide was treated with a 95% TFA, 2.5% TIS, 2.5% $H_2O$ cocktail (3h). Pale solid (>99% purity, estimated by HPLC-MS). **$^1$H NMR** (600 MHz, DMSO-$d_6$): δ 10.74 (d, $J$ = 2.4 Hz, 1H), 8.23 (dd, $J$ = 7.9, 2.4 Hz, 2H), 8.14 – 8.05 (m, 2H), 7.85 (d, $J$ = 8.0 Hz, 1H), 7.69 (d, $J$ = 1.7 Hz, 1H), 7.60 – 7.51 (m, 2H), 7.42 (m, 1H),7.32 (dt, $J$ = 8.1, 0.9 Hz, 1H), 7.27 (dt, $J$ = 7.6, 1.3 Hz, 1H), 7.12 – 7.03 (m, 4H), 6.97 (ddd, $J$ = 7.9, 6.9, 1.0 Hz, 1H), 4.57 (td, $J$ = 7.7, 6.0 Hz, 1H), 4.51 (ddd, $J$ = 10.5, 8.3, 4.0 Hz, 1H), 4.39 (td, $J$ = 8.1, 5.0 Hz, 1H), 4.31 (td, $J$ = 8.0, 5.6 Hz, 1H), 3.77 – 3.69 (m, 2H), 3.16 (dd, $J$ = 14.7, 5.0 Hz, 1H), 3.10 (dt, $J$ = 11.5, 5.7 Hz, 2H), 2.98 (ddd, $J$ = 14.8, 6.6, 2.7 Hz, 2H), 2.71 – 2.64 (m, 2H), 2.46 (dd, $J$ = 16.6, 7.7 Hz, 1H), 1.76 (s, 3H), 1.72 (q, $J$ = 6.0, 4.3 Hz, 1H), 1.60 – 1.44 (m, 3H) ppm. **HPLC-MS** (m/z): $[M+H]^+$ calcd. for $C_{34}H_{43}IN_{10}O_8$, 847.7; found, 847.2.

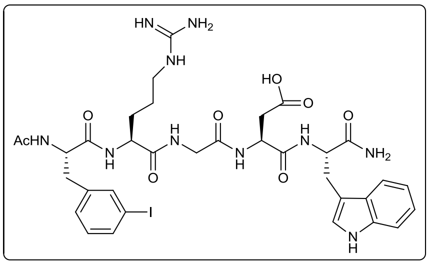

**H-Ala-*m*-I-Phe-Ser-Ala-Trp-Ala-OH (1i).** 2-Chlorotrityl resin (0.94 mmol/g). Amino acid coupling. Fmoc-XX-OH (3.0 eq.) were incorporated with a 5-min pre-activation with DIPCDI (3.0 eq.) and HOBt (3.0 eq.) in DMF for 1h. Fmoc-XX-OH: Fmoc-Ala-OH, Fmoc-

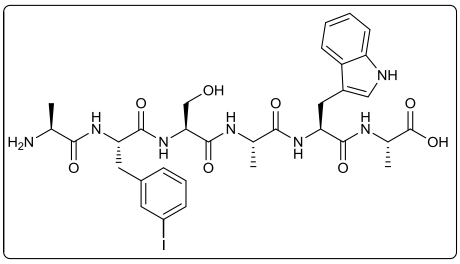

Trp(Boc)-OH, Fmoc-Ser(*t*-Bu)-OH, Fmoc-*m*-I-Phe-OH. The resin bound peptide was treated with a 95% TFA, 2.5% TIS, 2.5% cocktail (1h). Pale solid (>99% purity, estimated by HPLC-MS). **$^1$H NMR** (500 MHz, DMSO-$d_6$): δ 10.80 (d, *J* = 2.8 Hz, 1H), 8.73 (s, 1H), 8.36 (d, *J* = 7.7 Hz, 1H), 8.15 (d, *J* = 6.7 Hz, 1H), 7.94 (dd, *J* = 16.4, 7.4 Hz, 2H), 7.71 (s, 1H), 7.57 (t, *J* = 8.5 Hz, 2H), 7.30 (t, *J* = 8.5 Hz, 2H), 7.12 (d, *J* = 2.6 Hz, 1H), 7.06 (m, 2H), 6.97 (t, *J* = 7.5 Hz, 1H), 5.39 (s, 1H), 4.58 (m, 1H), 4.48 (td, *J* = 9.1, 4.5 Hz, 1H), 4.35 (q, *J* = 6.3 Hz, 1H), 4.17 (q, *J* = 7.0 Hz, 1H), 4.10 (q, *J* = 7.1 Hz, 1H), 3.70 (m, 2H), 3.59 (dd, *J* = 10.6, 5.7 Hz, 1H), 3.17 (dd, *J* = 14.7, 4.3 Hz, 1H), 3.04 (dd, *J* = 14.2, 3.8 Hz, 1H), 2.88 (dd, *J* = 14.8, 9.6 Hz, 1H), 2.76 (dd, *J* = 14.2, 10.7 Hz, 1H), 1.31 – 1.21 (m, 6H), 1.12 (d, *J* = 7.2 Hz, 3H) ppm. **HPLC-MS** (m/z): $[M+H]^+$ calcd. for $C_{32}H_{40}IN_7O_8$, 778.6; found, 778.2.

**Ac-Ala-*m*-I-Phe-Val-Trp-Ala-OH (1j).** 2-Chlorotrityl resin (1.0 mmol/g). Amino acid coupling.

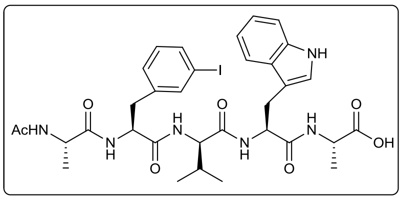


Fmoc-XX-OH (3.0 eq.) were incorporated with a 5-min pre-activation with DIPCDI (3.0 eq.) and HOBt (3.0 eq.) in DMF for 1h. Fmoc-XX-OH: Fmoc-Ala-OH, Fmoc-Trp-OH, Fmoc-Val-OH. Fmoc-*m*-I-Phe-OH (1.5 eq.) was incorporated with PyBOP (1.5 eq.), HOBt (1.5 eq.) and DIEA (3.0 eq.) in DMF for 1h. The N-terminal was acetilated, and then the resin bound peptide was treated with a 5% (v/v) TFA/DCM solution (5 x 1 min). Pale solid (>99% purity, estimated by HPLC-MS). **$^1$H NMR** (500 MHz, DMSO-$d_6$): δ 10.78 (d, *J* = 2.8 Hz, 1H), 8.17 (d, *J* = 7.1 Hz, 1H), 7.99 (t, *J* = 7.5 Hz, 2H), 7.94 (d, *J* = 8.2 Hz, 1H), 7.76 (d, *J* = 8.7 Hz, 1H), 7.60 (m, 2H), 7.50 (d, *J* = 7.9 Hz, 1H), 7.29 (d, *J* = 8.1 Hz, 1H), 7.20 (d, *J* = 7.7 Hz, 1H), 7.15 (d, *J* = 2.5 Hz, 1H), 7.04 (t, *J* = 7.5 Hz, 1H), 6.96 (td, *J* = 7.6, 3.4 Hz, 2H), 4.60 (td, *J* = 8.5, 4.8 Hz, 1H), 4.47 (td, *J* = 9.3, 8.7, 3.9 Hz, 1H), 4.25 – 4.11 (m, 3H), 3.13 (dd, *J* = 14.9, 4.7 Hz, 1H), 2.92 (ddd, *J* = 19.8, 10.3, 5.4 Hz, 2H), 2.76 – 2.67 (m, 1H), 1.94 (h, *J* = 6.8 Hz, 1H), 1.79 (s, 3H), 1.25 (d, *J* = 7.3 Hz, 3H), 1.10 (d, *J* = 7.2 Hz, 3H), 0.77 (dd, *J* = 9.8, 6.6 Hz, 6H) ppm. **HPLC-MS** (m/z): $[M+H]^+$ calcd. for $C_{33}H_{41}IN_6O_7$, 761.6; found, 761.3.

**Ac-Ala-*p*-I-Phe-Trp-Ala-OH (1k).** 2-Chlorotrityl resin (1.0 mmol/g). Amino acid coupling. Fmoc-XX-OH (3.0 eq.) were incorporated with a 5-min pre-activation with DIPCDI (3.0 eq.) and HOBt (3.0 eq.) in DMF for 1h. Fmoc-XX-OH: Fmoc-Ala-OH, Fmoc-Trp-OH. Fmoc-*p*-I-Phe-OH (1.5 eq.) was incorporated with HBTU (1.5 eq.), HOBt (1.5 eq.) and DIEA (3.0 eq.) in DMF for 1h. The N-terminal was acetylated, and the resin bound peptide was treated with a 5% (v/v) TFA/DCM solution (5 x 1 min). Pale solid (>99% purity, estimated by HPLC-MS). **$^1$H NMR** (500 MHz, DMSO-$d_6$): δ 10.81 (d, *J* = 2.5 Hz, 1H), 8.25 (d, *J* = 7.2 Hz, 1H), 8.02 (d, *J* = 8.1 Hz, 1H), 7.96 (d, *J* = 7.4 Hz, 1H), 7.83 (d, *J* = 8.1 Hz, 1H), 7.60 (d, *J* = 7.9 Hz, 1H), 7.56 – 7.45 (m, 2H), 7.32 (d, *J* = 8.1 Hz, 1H), 7.16 (d, *J* = 2.4 Hz, 1H), 7.05 (dd, *J* = 8.2, 6.9 Hz, 1H), 6.97 (t, *J* = 7.4, 6.9 Hz, 1H), 6.95 – 6.90 (m, 2H), 4.56 (td, *J* = 8.5, 4.7 Hz, 1H), 4.40 (td, *J* = 8.5, 4.6 Hz, 1H), 4.21 (dp, *J* = 21.4, 7.1 Hz, 2H), 3.13 (dd, *J* = 14.9, 4.7 Hz, 1H), 3.01 – 2.81 (m, 2H), 2.70 (dd, *J* = 13.9, 8.9 Hz, 1H), 1.79 (s, 3H), 1.27 (d, *J* = 7.3 Hz, 3H), 1.07 (d, *J* = 7.1 Hz, 3H). ppm. **HPLC-MS** (m/z): $[M+H]^+$ calcd. for $C_{28}H_{32}IN_5O_6$, 662.5; found, 662.2.

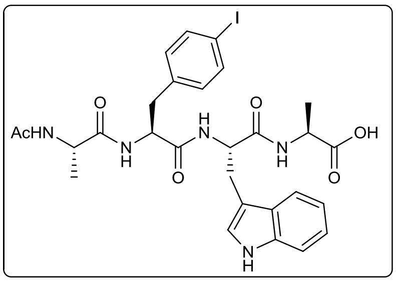


**General procedure for the C-H activation process of peptides 2a-2f**

Unless stated otherwise, the linear peptide (50 mg), $AgBF_4$ (1.0 eq.), 2-nitrobenzoic acid (1.5 eq) and $Pd(OAc)_2$ (0.05 eq) were placed in a microwave reactor vessel in DMF or in a 1:1 mixture of DMF:PBS. The mixture was heated under microwave irradiation (80 W) at 80 °C for 15 min. Water was added and the resulting suspension was filtered through Celite. The filtrate was successively washed with $Et_2O$, and the aqueous phase was lyophilized. The residue was purified by semi-preparative RP-HPLC (XBRIDGE$^{TM}$ Pref C18 5μM 19x100 mm column), using gradients of 25% of B to 60% B [solvent A (0.1% TFA in $H_2O$) and solvent B (0.07% TFA in ACN)], in 20 min, flux: 16 mL·min-1, detection at λ=220 nm.

**Synthesis and peptide characterization of locked peptides 2a-2f**

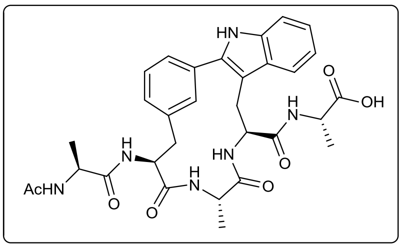

**Ac-Ala-(Cyclo-*m*)-[Phe-Ala-Trp]-Ala-OH (2a).** Starting from peptide **1a** (27 mg, 0.037 mmol) in a 1:1 mixture of DMF:PBS (total volume of 600 μL). Pale solid (38% conversion, estimated by HPLC-MS, 32% yield). **HRMS** (ESI) (m/z): $[M+H]^+$ calcd. for $C_{31}H_{36}N_6O_7$, 605.27182; found, 605.27400.

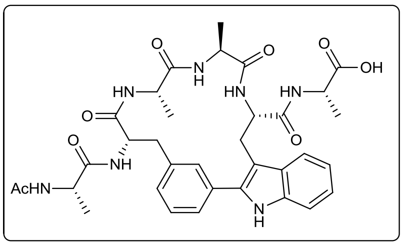

**Ac-Ala-(Cyclo-*m*)-[Phe-Ala-Ala-Trp]-Ala-OH (2b).** Starting from peptide **1b** (50 mg, 0.062 mmol) in a 1:1 mixture of DMF:PBS (total volume of 400 μL). Pale solid (total conversion, estimated by HPLC-MS, 26% yield). **HRMS** (ESI) (m/z): $[M+H]^+$ calcd. for $C_{34}H_{41}N_7O_8$, 676.30894; found, 676.31130. **$^1$H NMR** (500 MHz, $D_2O$): δ 10.58 (s, 1H), 8.49 (d, *J* = 3.2 Hz, 1H), 8.29 (m, 2H), 7.79 (d, *J* = 8.0 Hz, 1H), 7.68 (s, 1H), 7.59 (dd, *J* = 11.3, 5.5 Hz, 3H), 7.48 (m, 2H), 7.44 – 7.39 (m, 1H), 7.29 (t, *J* = 7.5 Hz, 1H), 7.22 (t, *J* = 7.5 Hz, 1H), 6.89 (d, *J* = 9.5 Hz, 1H), 4.89 (1H), 4.82 (1H), 4.37 – 4.22 (m, 2H), 4.02 (dd, *J* = 7.3, 3.0 Hz, 1H), 3.84 (qd, *J* = 7.3, 3.6 Hz, 1H), 3.73 (dd, *J* = 15.2, 3.4 Hz, 1H), 3.47 (dd, *J* = 15.1, 11.5 Hz, 1H), 3.25 (d, *J* = 7.3 Hz, 2H), 1.99 (s, 3H), 1.42 (d, *J* = 7.3 Hz, 3H), 1.34 (m, 6H), 0.82 (d, *J* = 7.4 Hz, 3H) ppm.

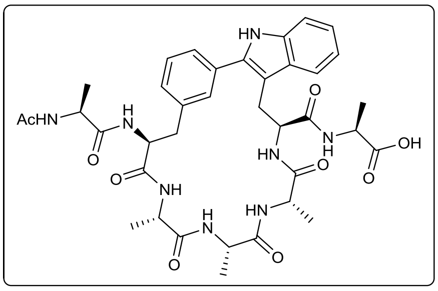

**Ac-Ala-(Cyclo-*m*)-[Phe-Ala-Ala-Ala-Trp]-Ala-OH (2c).** Starting from peptide **1c** (25 mg, 0.029 mmol) in a 1:1 mixture of DMF:PBS (total volume of 600 μL). Pale solid (total conversion, estimated by HPLC-MS, 54% yield). **HRMS** (ESI) (m/z): $[M+H]^+$ calcd. for $C_{37}H_{46}N_8O_9$, 747.34605; found, 747.34752.

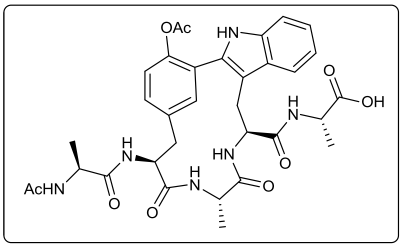

**Ac-Ala-(Cyclo-*m*)-[Tyr(OAc)-Ala-Trp]-Ala-OH (2d).** Starting from peptide **1d** (50 mg, 0.063 mmol) in a 1:1 mixture of DMF:PBS (total volume of 400 μL) (total conversion, estimated by HPLC-MS). **HPLC-MS** (m/z): $[M+H]^+$ calcd. for $C_{33}H_{38}N_6O_9$, 663.69; found, 663.14.

**Ac-Ala-(Cyclo-*m*)-[Tyr(OAc)-Ala-Ala-Trp]-Ala-OH (2e).** Starting from peptide **1e** (50 mg, 0.058

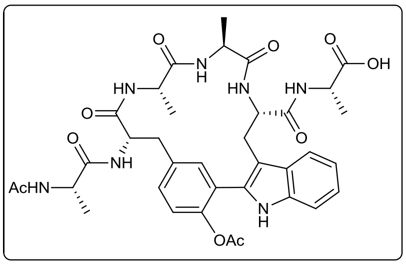

mmol) in a 1:1 mixture of DMF:PBS (total volume of 400 μL). (total conversion, estimated by HPLC-MS). **HRMS** (ESI) (m/z): $[M+H]^+$ calcd. for $C_{36}H_{43}N_7O_{10}$, 734.31442; found, 734.31681.

**Ac-Ala-(Cyclo-*m*)-[Tyr(OAc)-Ala-Ala-Ala-Trp]-Ala-OH (2f).** Starting from peptide **1f** (50 mg,

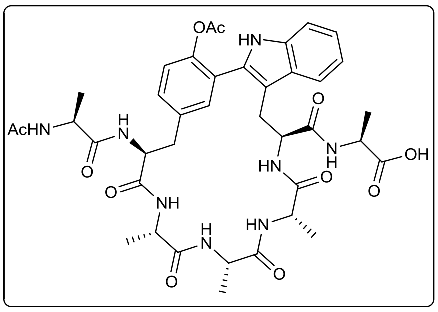

0.053 mmol) in a 1:1 mixture of DMF:PBS (total volume of 400 μL). (total conversion, estimated by HPLC-MS). **HPLC-MS** (m/z): $[M+H]^+$ calcd. for $C_{39}H_{48}N_8O_{11}$, 805.85; found, 805.34.

**General procedure for the C-H activation process of peptides 2g-2k**

Unless stated otherwise, the linear peptide (50 mg), $AgBF_4$ (2.0 eq.), trifluoroacetic acid (1.0 eq.) and $Pd(OAc)_2$ (0.05 eq.) were placed in a microwave reactor vessel in DMF. The mixture was heated under microwave irradiation (250 W) at 90 °C for 20 min. The residue was filtered and purified by semi-preparative RP-HPLC (XBRIDGE™ BEH 130, C18, 5μM OBD 19x50 mm column) [solvent A (0.1% FA in $H_2O$) and solvent B (0.1% FA in ACN)], in 10 min, flux: 20 mL·min-1, detection at λ=220 nm.

**Synthesis and peptide characterization of locked peptides 2g-2m**

**Ac-(Cyclo-*m*)-[Phe-Asn-Gly-Arg-Trp]-OH (2g).** Starting from peptide **1g** (186 mg, 0.220 mmol)

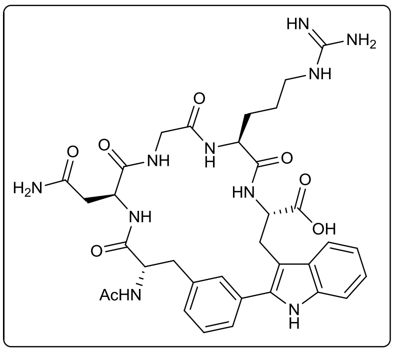

(77% conversion, estimated by HPLC-MS). Semi-preparative RP-HPLC gradient: 15-30% of B. Pale solid (45.3 mg, 29%). **$^1$H NMR** (500 MHz, DMSO-$d_6$): δ 11.15 (s, 1H), 8.42 (s, 1H), 8.35 (m, 2H), 7.79 (d, *J* = 8.2 Hz, 1H), 7.72 – 7.60 (m, 3H), 7.49 (d, *J* = 7.7 Hz, 1H), 7.38 (m, 2H), 7.32 (d, *J* = 8.1 Hz, 1H), 7.20 (d, *J* = 7.7 Hz, 1H), 7.06 (t, *J* = 7.6 Hz, 1H), 6.97 (t, *J* = 7.5 Hz, 1H), 6.85 (m, 1H), 4.68 (m, 1H), 4.60 (m, 1H), 4.48

(m, 1H), 4.30 (m, 1H), 3.98 (dd, $J$ = 16.4, 6.8 Hz, 1H), 3.43 (1H), 3,42 (1H), 3.14-2.99 (4H), 2.88 (t, $J$ = 12.4 Hz, 1H), 2.66 – 2.58 (m, 1H), 2.44 (1H), 1.77 (m, 4H), 1.55 (m, 1H), 1.41 (m, 2H) ppm. **IR** (Film, $cm^{-1}$) ν = 3417.08, 3276.16, 3064.77, 2911.03, 1623.49, 1533.81 $cm^{-1}$. **HRMS** (ESI) (m/z): $[M+H]^+$ calcd. for $C_{34}H_{42}N_{10}O_8$, 719.8; found, 720.1.

**Ac-(Cyclo-*m*)-[Phe-Arg-Gly-Asp-Trp]-NH$_2$ (2h).** Starting from peptide **1h** (190 mg, 0.224 mmol)

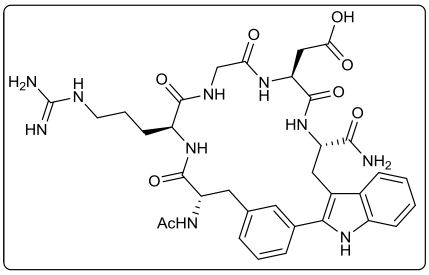


(70% conversion, estimated by HPLC-MS). Semi-preparative RP-HPLC gradient: 15-20% of B. Pale solid (28.9 mg, 18%). **$^1$H NMR** (600 MHz, DMSO-$d_6$): δ 11.00 (s, 1H), 9.88 (s, 1H), 8.39 (s, 1H), 8.32 (d, $J$ = 7.8 Hz, 1H), 8.06 (s, 1H), 7.88 (d, $J$ = 5.5 Hz, 1H), 7.69 (s, 1H), 7.59 (d, $J$ = 8.0 Hz, 1H), 7.42 (dt, $J$ = 7.8, 1.4 Hz, 1H), 7.39 (t, $J$ = 7.5 Hz, 1H), 7.35 (s, 1H), 7.31 (d, $J$ = 8.0 Hz, 1H), 7.24 (d, $J$ = 7.4 Hz), 7.10 – 7.04 (m, 2H), 7.01 – 6.97 (t, $J$ = 7.2 Hz, 1H), 6.89 (s, 1H), 4.66 (td, $J$ = 7.9, 5.4 Hz, 1H), 4.53 (m, 1H), 4.25 (dt, $J$ = 9.4, 4.4 Hz, 1H), 4.06 – 3.99 (m, 1H), 3.68 (dd, $J$ = 16.7, 6.6 Hz, 1H), 3.46 – 3.42 (m, 2H), 3.14 (m, 1H), 3.04 – 2.93 (m, 3H), 2.75 (m, 1H), 2.67 (dd, $J$ = 16.5, 4.0 Hz, 1H), 2.18 (dd, $J$ = 16.9, 5.7 Hz, 1H), 1.88 (s, 3H), 1.86 – 1.82 (m, 1H), 1.49 (m, 1H), 1.41 (m, 1H), 1.34 (m, 1H) ppm. **$^{13}$C NMR** (151 MHz, DMSO-$d_6$): δ 174.82, 173.61, 172.52, 170.87, 170.73, 168.98, 167.68, 157.10, 137.76, 135.84, 135.27, 132.97, 129.15, 128.80, 128.31, 128.07, 126.29, 121.11, 118.73, 118.47, 110.87, 107.50, 53.74, 53.36, 52.83, 49.22, 43.37, 40.69, 38.67, 37.51, 30.53, 27.10, 24.88, 22.53 ppm. **IR** (Film): ν = 3423.49, 3269.75, 2923.84, 1649.11, 1629.89 1540.21 $cm^{-1}$. **HRMS** (ESI) (m/z): $[M+H]^+$ calcd. for $C_{34}H_{42}N_{10}O_8$, 719.32598; found, 719.32718.

**H-Ala-(Cyclo-*m*)-[Phe-Ser-Ala-Trp]-Ala-OH (2i).** Starting from peptide **1i** (145 mg, 0.186 mmol). Additional microwave irradiation cycles were necessary to perform until obtain the desired product as the main peak by HPLC-MS (39% conversion, estimated by HPLC-MS). Semi-preparative RP-HPLC gradient: 12-32% of B. Pale solid (11.5 mg, 10%). **$^1$H NMR** (600 MHz, DMSO-$d_6$): δ 11.09 (s, 1H), 8.46 (m, 1H), 8.35 (t, $J$ = 4.7 Hz, 1H), 7.65 (s, 1H), 7.61 (d, $J$ = 7.9 Hz, 1H), 7.53 – 7.48 (m, 1H), 7.45 – 7.39 (m, 2H), 7.39

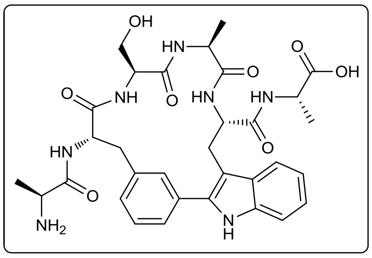

– 7.35 (m, 1H), 7.34 (d, *J* = 8.0 Hz, 1H), 7.22 (dt, *J* = 6.4, 1.9 Hz, 1H), 7.11 (t, *J* = 7.6 Hz, 1H), 7.04 – 7.00 (t, *J* = 7.6 Hz, 1H), 6.81 (d, *J* = 9.5 Hz, 1H), 4.97 (dd, *J* = 10.4, 3.6 Hz, 1H), 4.51 (ddd, *J* = 13.3, 8.5, 2.5 Hz, 1H), 4.15 (p, *J* = 7.3 Hz, 1H), 3.87 (dt, *J* = 5.9, 4.4 Hz, 1H), 3.70 (qd, *J* = 7.3, 4.1 Hz, 1H), 3.65 – 3.58 (m, 3H), 3.48 (1H), 3.30 (1H), 3.18 (dd, *J* = 13.6, 10.3 Hz, 1H), 3.08 (dd, *J* = 13.7, 3.5 Hz, 1H), 1.32 (d, *J* = 7.3 Hz, 3H), 1.17 (d, *J* = 6.9 Hz, 3H), 0.76 (d, *J* = 7.3 Hz, 3H). **$^{13}$C NMR** (151 MHz, DMSO-$d_6$): δ 173.74, 172.09, 171.69, 171.36, 170.54, 137.18, 135.95, 134.68, 132.59, 128.88, 128.73, 128.59, 128.48, 125.80, 121.63, 118.74, 118.18, 111.12, 108.33, 60.30, 58.37, 53.75, 51.87, 50.13, 49.47, 48.00, 36.64, 26.78, 20.08, 17.00, 16.02 ppm. **IR** (Film): ν = 3321.00, 2917.44, 3064.77, 2846.98, 1655.52, 1597.86 cm$^{-1}$. **HRMS** (ESI) (m/z): $[M+H]^+$ calcd. for $C_{32}H_{39}N_7O_8$, 650.29329; found, 650.29471.

**Ac-Ala-(Cyclo-*m*)-[Phe-Val-Trp]-Ala-OH (2j).** Starting from peptide **1j** (207 mg, 0.374 mmol) (71% conversion, estimated by HPLC-MS). Semi-preparative RP-HPLC gradient: 20-40% of B. Pale solid (55.2 mg, 32%). **$^1$H NMR** (600 MHz, DMSO-$d_6$): δ 11.19 (s, 1H), 8.09 (m, 2H), 7.67 (d, *J* = 9.6 Hz, 1H), 7.60 (d, *J* = 7.3 Hz, 1H), 7.56 (d, *J* = 7.0 Hz, 1H), 7.42 (d, *J* = 8.0 Hz, 1H), 7.37 (t, *J* = 7.5 Hz, 1H), 7.33 (dd, *J* = 7.9, 1.3 Hz, 2H), 7.29 (t, *J* = 1.7 Hz, 1H), 7.16 (dt, *J* = 7.5, 1.5 Hz, 1H), 7.08 (ddd, *J* = 8.1, 6.9, 1.1 Hz, 1H), 6.95 (ddd, *J* = 7.9, 6.9, 1.1 Hz, 1H), 4.65 (ddd, *J* = 9.7, 6.4, 3.2 Hz, 1H), 4.60 (ddd, *J* = 8.8, 7.2, 4.4 Hz, 1H), 4.31 (p, *J* = 7.2 Hz, 1H), 4.24 (p, *J* = 7.3 Hz, 1H), 4.07 (t, *J* = 9.5 Hz, 1H), 3,34 (1H), 3.20 (dd, *J* = 14.7, 6.6 Hz, 1H), 3.01 – 2.85 (m, 2H), 1.84 (s, 3H), 1.75 (m, 1H), 1.16 (d, *J* = 7.1 Hz, 3H), 0.99 (d, *J* = 7.3 Hz, 3H), 0.72 (dd, *J* = 6.7, 4.3 Hz, 6H) ppm. **$^{13}$C NMR** (151 MHz, DMSO-$d_6$): δ 173.66, 171.79, 171.26, 169.91, 169.79, 168.93, 137.29, 136.51, 135.78, 132.48, 129.45, 129.29, 128.56, 128.46, 127.18, 121.23, 119.51, 118.40, 110.83, 105.92, 57.69, 53.78, 52.32, 48.02, 47.76, 38.33, 30.44, 26.59, 22.45, 18.87, 18.53, 17.95, 16.67 ppm. **IR** (Film, cm$^{-1}$) ν = 3295.37, 3051.96, 2962.28, 1642.70, 1617.08, 1514.59 cm$^{-1}$. **HRMS** (ESI) (m/z): $[M+H]^+$ calcd. for $C_{33}H_{40}N_6O_7$, 633.30312; found, 633.30487.

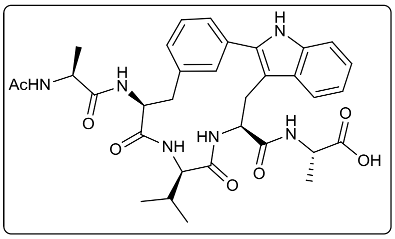

**(Cyclo-*p,p)*bis-[Phe-Trp]-(Ac-Ala-Phe-Trp-Ala-OH) (2k).** Starting from peptide **1k** (600 mg, 0.907 mmol) (60% conversion, estimated by HPLC-MS). Semi-preparative RP-HPLC gradient: 25-30% of B. Pale solid (3.8 mg, 1%). **$^1$H NMR** (800 MHz, DMSO-$d_6$): δ 11.20 (s, 1H), 8.13 (d, *J* = 7.7 Hz, 1H), 7.93 (d, *J* = 7.3 Hz, 1H), 7.90 (d, *J* = 7.0 Hz, 1H), 7.67 (d, *J* = 7.9 Hz, 1H), 7.61 (d, *J* = 7.6 Hz, 2H), 7.34 (d, *J* = 7.9 Hz, 1H), 7.25 (d, *J* = 7.7 Hz, 2H), 7.11 (t, *J* = 7.5 Hz, 1H), 7.03 (t, *J* = 7.5 Hz, 1H), 6.82 (d, *J* = 6.9 Hz, 1H), 4.24 – 4.15 (dp, *J* = 28.2, 7.1 Hz, 2H), 4.07 (ddd, *J* = 10.4, 6.9, 3.8 Hz, 1H), 3.96 (m, 1H), 3.43 (dd, *J* = 15.1, 3.7 Hz, 1H), 3.23 (dd, *J* = 15.2, 9.4 Hz, 1H), 2.64 (dd, *J* = 15.1, 10.6 Hz, 1H), 2.48 (m, 1H), 1.78 (s, 3H), 1.28 (d, *J* = 7.3 Hz, 3H), 1.08 (d, *J* = 7.0 Hz, 3H) ppm. **IR** (Film, $cm^{-1}$) ν = 3404.27, 2911.03, 1655.52 $cm^{-1}$. **HRMS** (ESI) (m/z): $[M+H]^+$ calcd. for $C_{56}H_{62}N_{10}O_{12}$, 1067.4621; found, 1067.4624.

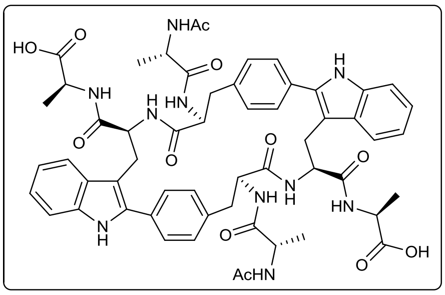

**H-Ile-Val-(Cyclo-*m*)-[Tyr-Pro-Trp]-OH (2l).** *AB linker incorporation for TentaGel S $NH_2$ resin.* AB linker (3.0 eq.) was attached to the resin (1.0 eq.) with DIPCDI (3.0 eq.), OxymaPure (3.0 eq.) in DMF at r.t for 1h. *First amino acid incorporation.* Fmoc-Trp-OH (4.0 eq.) was attached to the resin (1.0 eq.) with DIPCDI (2.0 eq.), DMAP (0.4 eq.) in DCM at r.t (1 x 2h, 1 x 16h). *End-capping of resin to block any remaining unreacted active resin sites.* Anhydride acetic (5.0 eq.) and DIEA (5.0 eq.) in DMF were added for 30 min. Peptide elongation. Fmoc-XX-OH (3.0 eq.) were incorporated with a 5-min pre-activation with DIPCDI (3.0 eq.) and OxymaPure (3.0 eq.) in DMF for 1h. Fmoc-XX-OH: Fmoc-Pro-OH, Fmoc-*3*-iodo-Tyr-OH, Fmoc-Val-OH, Fmoc-Ile-OH. *Stapled bond formation on solid-phase.* The resulting peptide anchored to the resin (67 mg, 0.065 mmol), $AgBF_4$ (13 mg, 0.065 mmol, 1.0 eq.), 2-nitrobenzoic acid (16 mg, 0.098 mmol, 1.5 eq.) and $Pd(OAc)_2$ (0.7 mg, 3.3 μmol, 0.05 eq.) were placed in a microwave reactor vessel in 900 μL of DMF. The mixture was heated under microwave irradiation (250 W) at 90 °C for 20 min. Three more batches were carried out following the same procedure and were combined. The peptide anchored to the resin was treated with 1% DDC in DMF and after removing the Fmoc group it was cleaved from the resin with a 95% TFA, 2.5% TIS, 2.5% $H_2O$ cocktail (1h). Pale solid (76.7 mg, 79% purity estimated by

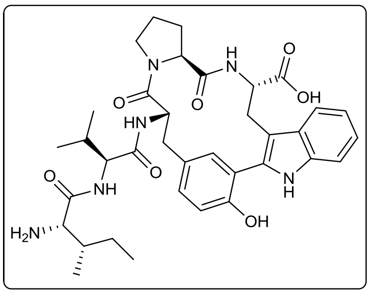

HPLC-MS, 32% yield). A pure fraction was obtained by semi-preparative RP-HPLC (XBRIDGE, PrepC18, 5μM OBD™ 19x150 mm column) [solvent A (0.1% FA in $H_2O$) and solvent B (0.1% FA in ACN)], in 20 min, flux: 16 mL·min-1, detection at λ=220 nm (gradient: 20-25% of B). **$^1$H NMR** (500 MHz, DMSO-$d_6$): δ 10.79 (s, 1H), 8.32 (d, $J$ = 6.7 Hz, 1H), 8.22 (s, 1H), 8.15 (d, $J$ = 8.9 Hz, 1H), 7.66 – 7.56 (m, 2H), 7.33 (d, $J$ = 8.0 Hz, 1H), 7.14 (d, $J$ = 2.2 Hz, 1H), 7.11 (dd, $J$ = 8.3, 2.2 Hz, 1H), 7.07 (t, $J$ = 7.5 Hz, 1H), 6.99 (t, $J$ = 7.4 Hz, 1H), 6.89 (d, $J$ = 8.2 Hz, 1H), 4.68 (td, $J$ = 8.0, 6.4, 3.6 Hz, 1H), 4.58 – 4.46 (m, 2H), 4.33 (dd, $J$ = 8.7, 6.3 Hz, 1H), 3.61 (q, $J$ = 8.5 Hz, 1H), 3.06 (td, $J$ = 12.7, 11.0, 4.9 Hz, 2H), 2.88 (dd, $J$ = 13.6, 3.3 Hz, 1H), 2.76 (dd, $J$ = 14.8, 9.0 Hz, 1H), 2.14 – 2.09 (m, 1H), 1.98 (h, $J$ = 6.7 Hz, 1H), 1.75 (tq, $J$ = 14.0, 5.8, 4.5 Hz, 2H), 1.55 (dtd, $J$ = 16.1, 11.9, 6.2 Hz, 1H), 1.50 – 1.38 (m, 2H), 1.23 (s, 2H), 1.11 (ddd, $J$ = 13.4, 9.5, 7.0 Hz, 1H), 0.91 – 0.79 (m, 12H) ppm. **HRMS** (ESI) (m/z): $[M+H]^+$ calcd. for $C_{36}H_{46}N_6O_7$, 675.35007; found, 675.35096.

**H-Asp-Asn-Ser-Gln-(Cyclo-*m*)-[Trp-Gly-Gly-Phe]-Ala-OH (2m).** *AB linker incorporation for TentaGel S $NH_2$ resin.* AB linker (3.0 eq.) was attached to the resin (1.0 eq.) with DIPCDI (3.0 eq.), OxymaPure (3.0 eq.) in DMF at r.t for 1h. *First amino acid incorporation*. Fmoc-Trp-OH (4.0 eq.) was attached to the resin (1.0 eq.) with DIPCDI (2.0 eq.), DMAP (0.4 eq.) in DCM at r.t (1 x 2h, 1 x 16h). *End-capping of resin to block any remaining unreacted active resin sites.* Anhydride acetic (5.0 eq.) and DIEA (5.0 eq.) in DMF were added for 30 min. *Peptide elongation until fifth amino acid incorporation.* Fmoc-XX-OH (3.0 eq.) were incorporated with a 5-min pre-activation with DIPCDI (3.0 eq.) and OxymaPure (3.0 eq.) in DMF for 1h. Fmoc-XX-OH: Fmoc-Ala-OH, Fmoc-Gly-OH, Fmoc-Trp-OH. Fmoc-*m*-I-Phe-OH (1.5 eq.) was incorporated with HBTU (1.5 eq.), HOBt (1.5 eq.) and DIEA (3.0 eq.) in DMF for 1h. *Stapled bond formation on solid-phase.* The resulting peptide anchored to the resin (64 mg, 0.072 mmol), $AgBF_4$ (14 mg, 0.072 mmol, 1.0 eq.), 2-nitrobenzoic acid (18 mg, 0.108 mmol, 1.5 eq.) and $Pd(OAc)_2$ (0.8 mg, 3.6 μmol, 0.05 eq.) were placed in a microwave reactor vessel in 980 μL of DMF. The mixture was heated under microwave irradiation (250 W) at 90 °C for 20 min. Three more batches

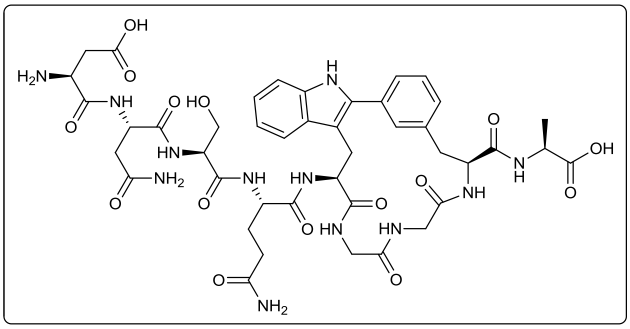

were carried out following the same procedure and were combined. *Peptide elongation until ninth amino acid incorporation.* The peptide anchored to the resin was treated with 1% DDC in DMF and Fmoc-XX-OH (3.0 eq.) were incorporated with a 5-min pre-activation with DIPCDI (3.0 eq.) and OxymaPure (3.0 eq.) in DMF for 1h. Fmoc-XX-OH: Fmoc-Gln(Trt)-OH, Fmoc-Ser(*t*-Bu)-OH, Fmoc-Asn(Trt)-OH, Fmoc-Asp(O*t*-Bu)-OH. After removing the Fmoc group, the final peptide sequence was cleaved from the resin with a 95% TFA, 2.5% TIS, 2.5% $H_2O$ cocktail (1h). Pale solid (86.3 mg, 85% purity estimated by HPLC-MS, 26% yield). A pure fraction was obtained by semi-preparative RP-HPLC (XBRIDGE, PrepC18, 5μM OBD$^{TM}$ 19x150 mm column) [solvent A (0.1% FA in $H_2O$) and solvent B (0.1% FA in ACN)], in 20 min, flux: 16 mL·min-1, detection at λ=220 nm (gradient: 10-25% of B). **$^1$H NMR** (500 MHz, DMSO-*d*$_6$): δ 11.16 (s, 1H), 8.58 (q, *J* = 6.7, 6.2 Hz, 2H), 8.50 (d, *J* = 7.2 Hz, 1H), 8.23 (d, *J* = 7.2 Hz, 1H), 8.15 (m, 2H), 8.01 (d, *J* = 7.9 Hz, 1H), 7.90 – 7.82 (m, 2H), 7.38 (m, 5H), 7.32 – 7.26 (m, 1H), 7.11 (t, *J* = 7.5 Hz, 1H), 7.05 (t, *J* = 7.4 Hz, 1H), 6.97 – 6.90 (m, 1H), 6.86 (d, *J* = 8.4 Hz, 1H), 6.78 – 6.69 (m, 1H), 4.87 – 4.77 (m, 1H), 4.67 (q, *J* = 6.2 Hz, 1H), 4.57 (ddd, *J* = 11.2, 8.3, 2.8 Hz, 1H), 4.27 (td, *J* = 8.8, 5.1 Hz, 1H), 4.22 (t, *J* = 7.3 Hz, 1H), 4.17 (q, *J* = 5.8 Hz, 1H), 3.85 (t, *J* = 5.8 Hz, 1H), 3.79 (dd, *J* = 15.8, 4.6 Hz, 1H), 3.71 (dd, *J* = 11.5, 5.8 Hz, 1H), 3.65 – 3.55 (m, 2H), 3.15 (d, *J* = 13.3 Hz, 1H), 3.01 (dd, *J* = 14.1, 4.5 Hz, 1H), 2.83 (dd, *J* = 13.7, 11.1 Hz, 1H), 2.58 (dd, *J* = 10.6, 5.9 Hz, 2H), 2.17 – 1.98 (m, 2H), 1.91 (ddt, *J* = 15.7, 10.7, 5.4 Hz, 1H), 1.77 (dtd, *J* = 14.8, 9.7, 5.3 Hz, 1H), 1.34 (d, *J* = 7.3 Hz, 3H) ppm. **HRMS** (ESI) (m/z): $[M+H]^+$ calcd. for $C_{43}H_{54}N_{12}O_5$, 979.39044; found, 979.39270.

**Synthesis and peptide characterization of peptides 1j-Bodipy and 2j-Bodipy**

**Ac-Ala-*m*-I-Phe-Val-Trp-Ala-*linker*-BODIPY (1j-Bodipy).** The linear peptide sequence **1j** (16.9 mg, 0.022 mmol, 1.2 eq.), EDC·HCl (5.3 mg, 0.028 mmol, 1.5 eq.) and HOBt·$H_2O$ (4.2 mg, 0.028 mmol, 1.5 eq.) were dissolved in 0.5 mL of DMF. Then, compound **19** (8.9 mg, 0.018 mmol, 1.0 eq.) dissolved in 1 mL of DMF and DIEA (9.7 μL, 0.055 mmol, 3.0 eq.) were added to give a final peptide concentration of 0.01 M and the solution was stirred for 16 h at r.t. Workup was done by removing the

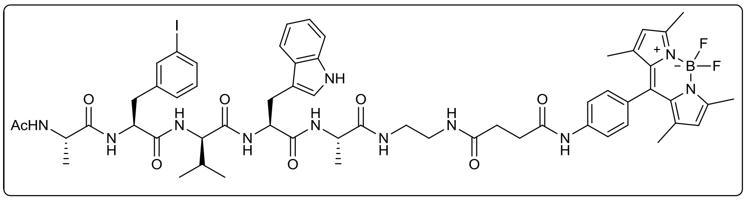

DMF under vacuum, dissolving the crude in EtOAc and extracting with $NaHCO_{3sat}$. Organic layers were combined, dried over sodium sulfate, filtered and concentrated under vacuum to afford 18.0 mg of the crude peptide (58% yield estimated by HPLC-MS conversion). A highly pure fraction of the linear peptide was obtained by purification of 11.0 mg of crude in a PoraPak Rxn RP 60 cc reverse phase column (2 g) [solvent A (0.1% FA in $H_2O$) and solvent B (0.1% FA in ACN)]. Red solid (95% purity by HPLC-MS). **HRMS** (ESI) (m/z): $[M+H]^+$ calcd. for $C_{58}H_{69}BF_2IN_{11}O_8$, 1224.45091; found, 1224.45351.

**Ac-Ala-(Cyclo-*m)*-[Phe-Val-Trp]-Ala-OH-*linker*-BODIPY (2j-Bodipy).** The staple peptide **2j**

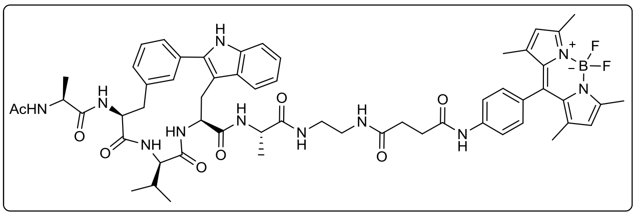

(16.1 mg, 0.021 mmol, 1.2 eq.), EDC·HCl (5.1 mg, 0.026 mmol, 1.5 eq.) and HOBt·$H_2O$ (4.1 mg, 0.026 mmol, 1.5 eq.) were dissolved in 0.6 mL of DMF. Then, compound **19** (8.5 mg, 0.018 mmol, 1.0 eq.) dissolved in 1 mL of DMF and DIEA (9.2 μL, 0.053 mmol, 3.0 eq.) were added to give a final peptide concentration of 0.01 M and the solution was stirred for 16 h at r.t. Workup was done by removing the DMF under vacuum and purifying the crude in a PoraPak Rxn RP 60 cc reverse phase column (2 g) [solvent A (0.1% FA in $H_2O$) and solvent B (0.1% FA in ACN)]. Red solid (10.2 mg, 97% purity by HPLC-MS, 53% yield). **HRMS** (ESI) (m/z): $[M+H]^+$ calcd. for $C_{58}H_{68}BF_2N_{11}O_8$, 1096.53862; found, 1096.54044.

**Synthesis and characterization of compounds 3-19**

**Cyclo(-Arg-Ala-Trp-Ala-Asn-Gly-) (3).** 2-Chlorotrityl resin (1.0 mmol/g). Amino acid coupling.

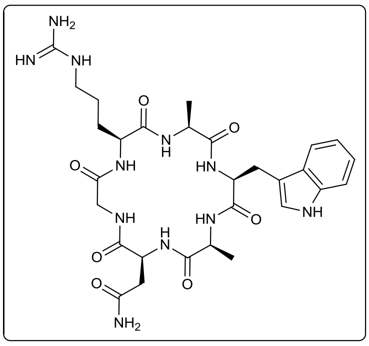

Fmoc-XX-OH (3.0 eq.) were incorporated with a 5-min pre-activation with DIPCDI (3.0 eq.) and OxymaPure (3.0 eq.) in DMF for 1h. Fmoc-XX-OH: Fmoc-Gly-OH, Fmoc-Asn(Trt)-OH, Fmoc-Ala-OH, Fmoc-Trp(Boc)-OH, Fmoc-Arg(Pbf)-OH. The resin bound peptide was treated with a 1% (v/v) TFA/DCM solution (5 x 1 min). Pale solid (94% purity, HPLC). Cyclization in solution. The free-amine free-acid protected linear peptide (1.5 g, 1.18 mmol) was dissolved in 395 mL of ACN/DMF (14:1) solution (0.003 M) and DIEA (6.0 eq.), PyBOP (3.0 eq.) and HOBt (3.0 eq.) were added. The solution was stirred at r.t until

the cyclization was complete (1h). Workup was done by extracting with $NH_4Cl_{sat}$ and $NaHCO_{3sat}$. Organic layers were combined, dried over sodium sulfate, filtered and concentrated under vacuum. Then, the macrocycle was treated with a 95% TFA, 2.5% TIS, 2.5% $H_2O$ cocktail (3h), washed with $Et_2O$, dissolved in ACN:$H_2O$ and lyophilized furnishing the corresponding peptide. Pale solid (436 mg, 33%). **$^1$H NMR** (500 MHz, DMSO-*d*$_6$): δ 10.83 (d, *J* = 2.4 Hz, 1H), 8.25 (t, *J* = 5.6 Hz, 1H), 8.14 (d, *J* = 7.3 Hz, 1H), 8.09 (d, *J* = 6.1 Hz, 1H), 8.06 (d, *J* = 8.1 Hz, 1H), 8.00 – 7.97 (d, *J* = 7.4 Hz, 1H), 7.88 (d, *J* = 6.9 Hz, 1H), 7.60 – 7.56 (d, *J* = 7.9 Hz, 1H), 7.47 (t, *J* = 5.7 Hz, 1H), 7.42 (m, 1H), 7.32 (dt, *J* = 8.1, 0.9 Hz, 1H), 7.14 (d, *J* = 2.4 Hz, 1H), 7.06 (ddd, *J* = 8.2, 6.9, 1.2 Hz, 1H), 6.97 (ddd, *J* = 7.9, 6.9, 1.0 Hz, 1H), 6.92 (m, 1H),4.35 (q, *J* = 6.4 Hz, 1H), 4.32 – 4.24 (m, 1H), 4.15 – 4.02 (m, 2H), 3.97 (p, *J* = 7.0 Hz, 1H), 3.83 (dd, *J* = 16.1, 5.5 Hz, 1H), 3.56 – 3.52 (m, 1H), 3.18 (m, 2H), 3.10 – 3.06 (m, 2H), 2.67 – 2.55 (m, 2H), 1.87 – 1.76 (m, 1H), 1.60 (m, 1H), 1.52 – 1.40 (m, 2H), 1.28 – 1.15 (m, 6H) ppm. **IR** (Film, $cm^{-1}$) ν = 3276.16, 3186.48, 3051.96, 2917.44, 1642.70, 1508.19 $cm^{-1}$. **HRMS** (ESI) (m/z): $[M+H]^+$ calcd. for $C_{29}H_{41}N_{11}O_7$, 656.32632; found, 656.32680.

**Cyclo(-Leu-Leu-Val-Leu-*p*-I-Phe-) (4).** 2-Chlorotrityl resin (0.94 mmol/g). Amino acid coupling.

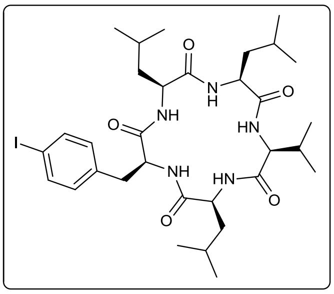


Fmoc-XX-OH (3.0 eq.) were incorporated with a 5-min pre-activation with DIPCDI (3.0 eq.) and OxymaPure (3.0 eq.) in DMF for 1h. Fmoc-XX-OH: Fmoc-*p*-I-Phe-OH, Fmoc-Leu-OH, Fmoc-Val-OH. The resin bound peptide was treated with a 5% (v/v) TFA/DCM solution (5 x 2 min). Pale solid (85% purity, HPLC). Cyclization in solution. The free-amine free-acid linear peptide (119 mg, 0.163 mmol) was dissolved in 55 mL of ACN/DCM (1:9) solution (0.003 M) and DIEA (6.0 eq.), HATU (1.5 eq.) and TBTU (1.5 eq.) were added. The solution was stirred at r.t until the cyclization was complete (3h). Workup was done by extracting with $NH_4Cl_{sat}$ and $NaHCO_{3sat}$. Organic layers were combined, dried over sodium sulfate, filtered and concentrated under vacuum. Then, the macrocycle was purified via flash column chromatography using and EtOAc/hexane gradient on silica gel furnishing the corresponding peptide. Pale solid (28.1 mg, 24%). **$^1$H NMR** (500 MHz, DMSO-*d*$_6$): δ 8.15 (t, *J* = 7.7 Hz, 2H), 8.02 (d, *J* = 8.4 Hz, 1H), 8.00 – 7.89 (m, 2H), 7.62 (d, *J* = 7.7 Hz, 2H), 7.03 (d, *J* = 7.9 Hz, 2H), 4.27 (q, *J* = 8.0 Hz, 1H), 4.12 (q, *J* = 8.4, 6.2 Hz, 1H), 4.05 (m, 1H), 3.94 (q, *J* =

8.4 Hz, 1H), 3.66 (t, *J* = 9.5 Hz, 1H), 3.07 – 2.95 (m, 2H), 2.15 (m, 1H), 1.68 (m, 2H), 1.58 – 1.43 (m, 4H), 1.38 – 1.24 (m, 3H), 0.92 – 0.74 (m, 24H) ppm. **IR** (Film, $cm^{-1}$) ν = 3295.37, 3077.58, 2962.28, 1655.52, 1540.21 $cm^{-1}$. **HRMS** (ESI) (m/z): $[M+H]^+$ calcd. for $C_{32}H_{50}IN_5O_5$, 712.29294; found, 712.29392.

**Cyclo(Ala-Asn-Gly-Arg-Ala-C2-Trp-)—Cyclo(C4-Phe-Leu-Leu-Val-Leu-) (5).** Macrocycle

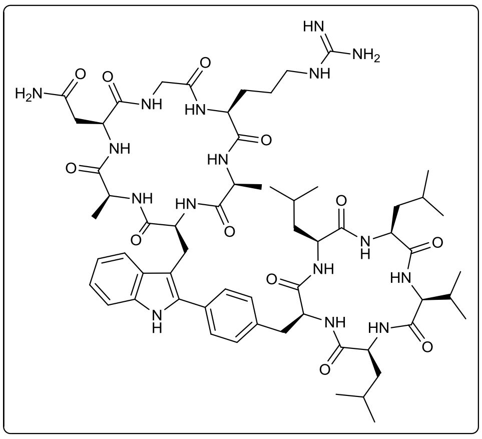

**4** (40.0 mg, 0.056 mmol), macrocycle **3** (55.3 mg, 0.084 mmol, 1.5 eq.), $AgBF_4$ (43.8 mg, 0.225 mmol, 4.0 eq.), pivalic acid (5.7 mg, 0.056 mmol, 1.0 eq.) and $Pd(OAc)_2$ (1.4 mg, 0.077 mmol, 0.1 eq.) were placed in a microwave reactor vessel in 2 mL of PBS:DMF (1:1). The mixture was heated under microwave irradiation (250 W) at 90 °C for 20 min. The irradiation cycle was repeated by adding a new portion of $Pd(OAc)_2$ and $AgBF_4$. The residue was filtered and partially purified in a PoraPak Rxn RP 60 cc reverse phase column (5 g) [solvent A (0.1% FA in $H_2O$) and solvent B (0.1% FA in ACN)]. Pale solid (1.63 mg, 2% yield estimated by HPLC-MS conversion). A pure fraction was obtained by analytic RP-HPLC (XBRIDGE™ BEH 130, C18, 5μM 10x100 mm column) [solvent A (0.045% TFA in $H_2O$) and solvent B (0.036% TFA in ACN)], in 30 min, flux: 3 mL·min-1, detection at λ=220 nm (gradient: 30-50% of B). **$^1$H NMR** (600 MHz, DMSO-*d*$_6$): δ 11.16 (s, 1H), 8.45 (t, *J* = 5.9 Hz, 1H), 8.19-8.18 (t, *J* = 7.9 Hz, 2H), 8.11 – 7.90 (m, 8H), 7.64 (d, *J* = 8.1 Hz, 2H), 7.59 (d, *J* = 8.0 Hz, 1H), 7.44 – 7.40 (m, 2H), 7.35 – 7.31 (m, 3H), 7.11 – 7.06 (m, 1H), 7.00 – 6.96 (m, 1H), 6.91 (m, 1H), 4.39 – 4.29 (m, 2H), 4.25 (q, *J* = 7.0 Hz, 1H), 4.19 – 4.09 (m, 3H), 4.02 (m, 1H), 3.97 (q, *J* = 8.7 Hz, 1H), 3.92 (q, *J* = 6.6, 6.1 Hz, 1H), 3.86 (dd, *J* = 15.8, 5.8 Hz, 1H), 3.72 (t, *J* = 9.0 Hz, 1H), 3.41 (1H), 3.40-3.32 (2H) 3.12 (m, 2H), 3.10 – 3.04 (m, 2H), 2.58 (d, *J* = 6.2 Hz, 2H), 2.19 – 2.11 (m, 1H), 1.79 (m, 1H), 1.73 – 1.41 (m, 11H), 1.39 – 1.34 (m, 1H), 1.11 (dd, *J* = 7.1, 3.2 Hz, 6H), 0.92 (d, *J* = 6.3 Hz, 3H), 0.90 – 0.81 (m, 18H), 0.77 (d, *J* = 6.3 Hz, 3H) ppm. **IR** (Film, $cm^{-1}$) ν = 3321.00, 3199.29, 2962.28, 2923.84, 1655.52, 1533.81 $cm^{-1}$. **HRMS** (ESI) (m/z): $[M+H]^+$ calcd. for $C_{61}H_{91}N_{16}O_{12}$, 1239.69969; found, 1239.70241.

**Bis[cyclo(-Arg-Ala-Trp-Ala-Asn-Gly-)] adduct (6).** 1,4-diiodobenzene (35 mg, 0.106 mmol),

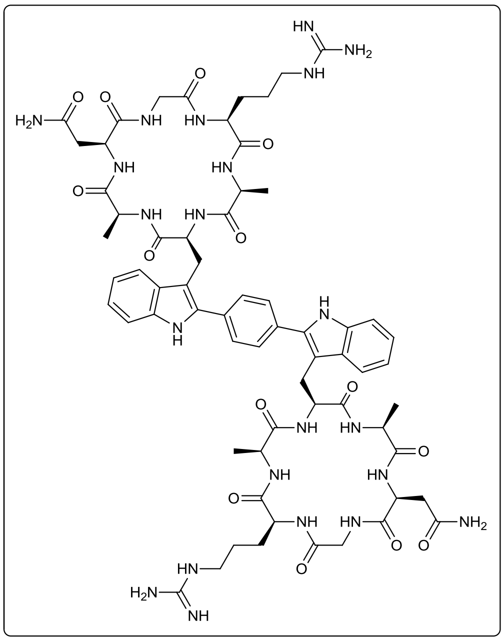

macrocycle **3** (209 mg, 0.318 mmol, 3.0 eq.), $AgBF_4$ (124 mg, 0.637 mmol, 6.0 eq.), pivalic acid (16.3 mg, 0.159 mmol, 1.5 eq.) and $Pd(OAc)_2$ (9.5 mg, 0.042 mmol, 0.4 eq.) were placed in a microwave reactor vessel in 2 mL of PBS:DMF (1:1). The mixture was heated under microwave irradiation (250 W) at 90 °C for 20 min. The crude was filtered and the workup was done by washing with AcOEt and then precipitating by adding ACN to the aqueous phase. The precipitated was washed with ACN, decanted and dried, obtaining 159 mg of crude (pale solid, 42%, yield estimated by HPLC-MS conversion). A pure fraction was obtained by semi-preparative RP-HPLC (Phenomenex Jupiter, C18, 10µM, 21.20x100 mm column, [solvent A (0.1% FA in $H_2O$) and solvent B (0.05% FA in ACN)], in 20 min, flux: 20 mL·min-1, detection at λ=220 nm (gradient: 15-20% of B). **$^1$H NMR** (600 MHz, DMSO-$d_6$): δ 11.29 (s, 2H), 8.40 (s, 2H), 8.17 (s, 2H), 8.06 (m, 6H), 7.97 (d, $J$ = 8.7 Hz, 2H), 7.87 (s, 4H), 7.65 (m, 2H), 7.42 (s, 2H), 7.36 (d, $J$ = 8.0 Hz, 2H), 7.32 – 7.16 (m, 6H), 7.13 – 7.08 (m, 2H), 7.00 (t, $J$ = 7.3 Hz, 2H), 6.89 (s, 2H), 4.36 (m, 4H), 4.18 (m, 2H), 4.09 – 4.02 (m, 2H), 3.98 (m, 2H), 3.86 (m, 2H), 3.56 – 3.48 (m, 2H), 3.44 – 3.39 (m, 4H), 3.08 (q, $J$ = 6.4 Hz, 4H), 2.63 – 2.56 (m, 4H), 1.81 (dd, $J$ = 13.4, 6.9 Hz, 2H), 1.62 (m, 2H), 1.47 (m, 4H), 1.11 (m, 12H) ppm. **IR** (Film, $cm^{-1}$) ν = 3314.59, 3199.29, 3051.96, 2911.03, 1661.92, 1533.81 $cm^{-1}$. **HRMS** (ESI) (m/z): [M] calcd. for $C_{64}H_{84}N_{22}O_{14}$, 1384.65373; found, 1384.65505.

**H-Ala-(Cyclo-*m*)-[Phe-Gly-Ala-Trp]-Gly-OH (9).** *AB linker incorporation for TentaGel S $NH_2$ resin*. AB linker (3.0 eq.) was attached to the resin (1.0 eq.) with DIPCDI (3.0 eq.), OxymaPure (3.0 eq.) in DMF at r.t for 1h. *First amino acid incorporation*. Fmoc-Gly-OH (4.0 eq.) was attached to the resin (1.0 eq.) with DIPCDI (2.0 eq.), DMAP (0.4 eq.) in DCM at r.t

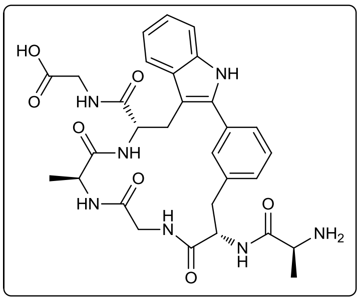

(1 x 2h, 1 x 16h). *End-capping of resin to block any remaining unreacted active resin sites.*Anhydride acetic (5.0 eq.) and DIEA (5.0 eq.) in DMF were added for 30 min. *Peptide elongation.* Fmoc-XX-OH (3.0 eq.) were incorporated with a 5-min pre-activation with DIPCDI (3.0 eq.) and OxymaPure (3.0 eq.) in DMF for 1h. Fmoc-XX-OH: Fmoc-Trp-OH, Fmoc-Ala-OH, Fmoc-Gly-OH. Fmoc-*m*-I-Phe-OH (1.5 eq.) was incorporated with HBTU (1.5 eq.), HOBt (1.5 eq.) and DIEA (3.0 eq.) in DMF for 1h. *Stapled bond formation on solid-phase.* The resulting peptide **7** anchored to the resin (139 mg, 0.145 mmol), $AgBF_4$ (28 mg, 0.144 mmol, 1.0 eq.), 2-nitrobenzoic acid (36 mg, 0.215 mmol, 1.5 eq.) and $Pd(OAc)_2$ (1.6 mg, 7.1 μmol, 0.05 eq.) were placed in a microwave reactor vessel in 2 mL of DMF. The mixture was heated under microwave irradiation (250 W) at 90 °C for 20 min. Eight more batches were carried out following the same procedure and were combined. The peptide **8** anchored to the resin was treated with 1% DDC in DMF and after removing the Fmoc group it was cleaved from the resin with a 95% TFA, 2.5% TIS, 2.5% $H_2O$ cocktail (1h). (85% purity, estimated by HPLC-MS). **$^1$H NMR** (500 MHz, DMSO-$d_6$): δ 11.15 (s, 1H), 8.43 (t, *J* = 5.1 Hz, 1H), 7.84 (s, 1H), 7.59 (d, *J* = 7.9 Hz, 1H), 7.50 (s, 1H), 7.41-7.35 (m, 4H), 7.17 – 7.08 (m, 2H), 7.01 (t, *J* = 7.4 Hz, 1H), 6.80 (d, *J* = 8.4 Hz, 1H), 4.83 – 4.74 (m, 1H), 4.55 (q, *J* = 8.2 Hz, 1H), 3.83 (m, 1H), 3.76 (m, 1H), 3.71 (m, 1H), 3.57 – 3.53 (m, 3H), 3.38-3.32 (m, 2H), 3.14 – 3.11 (m, 1H), 3.03 (dd, *J* = 13.5, 8.8 Hz, 1H), 1.28 (d, *J* = 6.9 Hz, 3H), 0.80 (d, *J* = 7.2 Hz, 3H) ppm. **IR** (Film, $cm^{-1}$) ν = 3288.97, 3051.96, 2923.84, 1649.11, 1527.40 $cm^{-1}$. **HRMS** (ESI) (m/z): $[M+H]^+$ calcd. for $C_{30}H_{35}N_7O_7$, 606.26707; found, 606.26754.

**Cyclo[-Ala-(Cyclo-*m*)-[Phe-Gly-Ala-Trp]-Gly-] (10).** The free-amine free-acid stapled peptide **9**

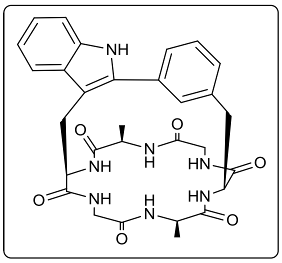


(92.2 mg, 0.152 mmol) was dissolved in 152 mL of DMF (0.001 M) and DIEA (6.0 eq.) and PyAOP (2.0 eq.) were added. The solution was stirred at r.t until the cyclization was complete (1.5h). DMF was removed under vacuum, and the crude was dissolved in EtOAc and extracted with $NH_4Cl_{sat}$ and $NaHCO_{3sat}$. Organic layers were combined, dried over sodium sulfate, filtered and concentrated under vacuum. The crude was purified by semi-preparative RP-HPLC (XBRIDGE™ BEH 130, C18, 5μM OBD 19x50 mm column) [solvent A (0.1% FA in $H_2O$) and solvent B (0.1% FA in ACN)], in 10 min, flux: 20 mL·min-1, detection at λ=220 nm (gradient: 20-30% of B). Pale solid (16.0 mg, 18%).

**$^{1}$H NMR** (600 MHz, DMSO-$d_6$): δ 11.23 (s, 1H), 8.53 (s, 1H), 8.19 (s, 1H), 7.49 – 7.35 (m, 6H), 7.32 (d, *J* = 8.0 Hz, 1H), 7.17 (dt, *J* = 7.7, 1.4 Hz, 1H), 7.06 (ddd, *J* = 8.0, 6.9, 1.2 Hz, 1H), 6.96 (ddd, *J* = 8.0, 6.8, 1.0 Hz, 1H), 6.77 (d, *J* = 8.9 Hz, 1H), 6.46 (s, 1H), 4.72 – 4.60 (m, 1H), 4.55 (dt, *J* = 8.6, 6.7 Hz, 1H), 4.14 (m, 1H), 4.11 – 4.05 (m, 1H), 3.57 (dd, *J* = 13.4, 1.9 Hz, 1H), 3.54 (d, *J* = 4.9 Hz, 1H), 3.50 (m, 2H), 3.49 – 3.43 (m, 1H), 3.37 – 3.34 (m, 1H), 3.15 (dd, *J* = 14.1, 2.6 Hz, 1H), 2.83 (dd, *J* = 13.7, 6.7 Hz, 1H), 1.28 (d, *J* = 7.4 Hz, 3H), 0.34 (d, *J* = 7.3 Hz, 3H) ppm. **$^{13}$C NMR** (151 MHz, DMSO-$d_6$): δ 171.42, 171.41, 170.82, 170.71, 169.08, 168.56, 136.02, 135.69, 135.66, 133.76, 130.16, 130.11, 129.65, 128.24, 126.79, 121.22, 118.51, 118.45, 111.02, 105.66, 51.27, 51.18, 49.44, 48.72, 43.31, 43.18, 35.90, 25.07, 16.43, 15.23 ppm. **IR** (Film, cm$^{-1}$) ν = 3378.65, 3301.78, 3051.96, 2930.25, 1649.11, 1533.81 cm$^{-1}$. **HRMS** (ESI) (m/z): [M+H]$^+$ calcd. for $C_{30}H_{33}N_7O_6$, 588.25651; found, 588.25770.

**Ac-Trp-Ala-Gly-*3,5*-I,I-Tyr(OAc)-Ala-Gly-Trp-OH (11).** 2-Chlorotrityl resin (0.8 mmol/g).

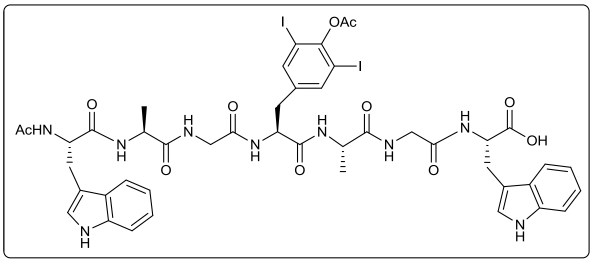


Amino acid coupling. Fmoc-XX-OH (3.0 eq.) were incorporated with a 5-min pre-activation with DIPCDI (3.0 eq.) and OxymaPure (3.0 eq.) in DMF for 1h. Fmoc-XX-OH: Fmoc-Gly-OH, Fmoc-Ala-OH, Fmoc-*m.m*-I,I-Tyr-OH, Fmoc-Trp-OH. The N-terminal was acetilated, and the resin bound peptide was treated with a 5% (v/v) TFA/DCM solution (5 x 2 min). Pale solid (>99% purity, estimated by HPLC-MS). **$^{1}$H NMR** (500 MHz, DMSO-$d_6$): δ 10.84 (d, *J* = 2.5 Hz, 1H), 10.75 (d, *J* = 2.5 Hz, 1H), 8.30 (d, *J* = 7.2 Hz, 1H), 8.13 (d, *J* = 7.2 Hz, 1H), 8.10 – 8.03 (m, 3H), 8.01 (d, *J* = 8.0 Hz, 1H), 7.92 (t, *J* = 5.7 Hz, 1H), 7.77 (s, 2H), 7.59 (d, *J* = 7.9 Hz, 1H), 7.52 (d, *J* = 7.9 Hz, 1H), 7.31 (dd, *J* = 9.7, 8.3 Hz, 2H), 7.13 (dd, *J* = 12.0, 2.4 Hz, 2H), 7.09 – 7.00 (m, 2H), 6.96 (dddd, *J* = 8.9, 7.8, 6.9, 1.1 Hz, 2H), 4.56 – 4.50 (m, 2H), 4.48 (dt, *J* = 7.8, 3.8 Hz, 1H), 4.33 – 4.18 (m, 2H), 3.79 – 3.68 (m, 3H), 3.56 (1H), 3.18 (dd, *J* = 14.7, 5.3 Hz, 1H), 3.11 (dd, *J* = 14.7, 4.7 Hz, 1H), 3.04 (dd, *J* = 14.6, 8.0 Hz, 1H), 2.97 (dd, *J* = 14.1, 3.8 Hz, 1H), 2.93 – 2.84 (m, 1H), 2.67 (dd, *J* = 13.9, 10.0 Hz, 1H), 2.34 (s, 3H), 1.76 (s, 3H), 1.21 (dd, *J* = 10.6, 7.0 Hz, 6H) ppm. **HPLC-MS** (m/z): [M+H]$^+$ calcd. for $C_{45}H_{49}I_2N_9O_{11}$,1146.7; found, 1146.9.

**Ac-(bicyclo-*m,m*)-[Trp-Ala-Gly-Tyr(OAc)]-[Tyr(OAc)-Ala-Gly-Trp]-OH (12).** The linear

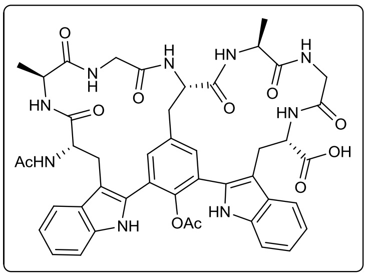


peptide **11** (50 mg, 0.044 mmol), $AgBF_4$ (51 mg, 0.262 mmol, 6.0 eq.), pivalic acid (6.7 mg, 0.066 mmol, 1.5 eq.) and $Pd(OAc)_2$ (3.9 mg, 0.018 mmol, 0.4 eq.) were placed in a microwave reactor vessel in 500 μL of DMF. The mixture was heated under microwave irradiation (250 W) at 90 °C for 20 min. Three more batches were carried out following the same procedure. All the crudes were filtered and combined (25% conversion, estimated by HPLC-MS). The crude was purified by semi-preparative RP-HPLC (XBRIDGE$^{TM}$ BEH 130, C18, 5μM OBD 19x50 mm column, [solvent A (0.1% FA in $H_2O$) and solvent B (0.1% FA in ACN)], in 10 min, flux: 20 mL·min-1, detection at λ=220 nm, gradient: 25-30% of B. **$^1$H NMR** (600 MHz, DMSO-$d_6$): δ 10.87 (d, *J* = 12.6 Hz, 2H), 8.71 (d, *J* = 8.0 Hz, 1H), 8.50 (s, 1H), 8.24 (s, 1H), 8.03 (m, 1H), 7.98 (d, *J* = 8.0 Hz, 1H), 7.58 (d, *J* = 8.0 Hz, 1H), 7.43 (s, 1H), 7.38 – 7.32 (m, 2H), 7.25 (d, *J* = 8.2 Hz, 1H), 7.22 (d, *J* = 7.0 Hz, 1H), 7.09 (ddd, *J* = 8.1, 6.9, 1.1 Hz, 1H), 7.03 (dt, *J* = 15.7, 7.6 Hz, 2H), 6.96 (t, *J* = 7.8 Hz, 1H), 6.79 (2H), 5.04 (m, 1H), 4.94 (q, *J* = 7.4 Hz, 1H), 4.75 (m, 1H), 4.43 – 4.35 (m, 1H), 4.19 (p, *J* = 6.9 Hz, 1H), 3.90 (dd, *J* = 17.0, 7.1 Hz, 1H), 3.58 (dd, *J* = 16.7, 7.0 Hz, 1H), 3.51 – 3.44 (m, 1H), 3.38 (1H), 3.30 (1H), 3.24 (1H), 3.19 (m, 1H), 3.04 (s, 2H), 2.83 (t, *J* = 13.2 Hz, 1H), 1.94 (s, 3H), 1.49 (s, 3H), 1.21 (d, *J* = 7.2 Hz, 3H), 1.03 (d, *J* = 7.0 Hz, 3H) ppm. **HRMS** (ESI) (m/z): $[M+H]^+$ calcd. for $C_{45}H_{47}N_9O_{11}$, 890.34678; found, 890.34796.

**Ac-*m,m'*-I,I-Tyr(OAc)-OH (13).** To a vigorously stirred suspension of commercially available H-

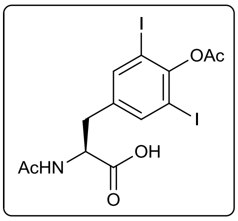


3,5-*I,I*-Tyr-OH·$2H_2O$ (2.17 g, 4.62 rnmol) in $H_2O$ (14.2 rnL) at 0°C was added $Et_3N$ (1.29 mL, 9.24 rnmol). Acetic anhydride (1.05 mL, 11.1 rnmol) was added dropwise at 0°C and $Et_3N$ was added to maintain the pH between 6 and 8. After the addition was complete, the reaction was warmed to rt and stirred vigorously. After 5 min of reaction, a new portion of acetic anhydride and $Et_3N$ was added. Once the reaction was completed (30 min), The solution was then carefully acidified to pH 2 with 1.2M $HCl_{(aq)}$. The white precipitate formed was dissolved in EtOAc and subsequent extractions with $H_2O$ afforded the desired product as a white solid (2.41 g, 90%). **$^1$H NMR** (400 MHz, DMSO-$d_6$): δ 12.76 (s, 1H), 8.21 (d, *J* = 8.1 Hz, 1H),

7.73 (s, 2H), 4.38 (ddd, $J$ = 9.5, 8.0, 4.9 Hz, 1H), 3.00 (dd, $J$ = 13.9, 4.9 Hz, 1H), 2.78 (dd, $J$ = 13.9, 9.5 Hz, 1H), 2.35 (s, 3H), 1.80 (s, 3H) ppm.

**Ac-Trp-OH (14).**[4] To a solution of tryptophan (2.5 g, 12.2 mmol) and NaOH (0.588 g, 14.7 mmol) in 75 mL of $H_2O$ was added acetic anhydride (15 mL, 159 mmol) and was stirred for 2 h, resulting in a white solid precipitate. The precipitate was filtered from the reaction mixture and rinsed with cold water. The residue was suspended in 50 mL of 0.2 M HCl, cooled, filtered and washed with cold water and dried to give a white solid (1.46 g, 48%). **$^1$H NMR** (400 MHz, DMSO-$d_6$): δ 12.58 (s, 1H), 10.91 – 10.74 (m, 1H), 8.13 (d, $J$ = 7.8 Hz, 1H), 7.52 (d, $J$ = 7.9 Hz, 1H), 7.33 (d, $J$ = 8.1 Hz, 1H), 7.13 (d, $J$ = 2.4 Hz, 1H), 7.06 (ddd, $J$ = 8.2, 7.0, 1.2 Hz, 1H), 6.98 (ddd, $J$ = 7.9, 7.0, 1.1 Hz, 1H), 4.45 (ddd, $J$ = 8.6, 7.8, 5.1 Hz, 1H), 3.15 (ddd, $J$ = 14.6, 5.1, 0.9 Hz, 1H), 3.06 – 2.87 (m, 1H), 1.80 (s, 3H) ppm. **$^{13}$C NMR** (101 MHz): δ 173.54, 169.18, 136.07, 127.18, 123.50, 120.89, 118.33, 118.13, 111.36, 109.96, 52.96, 27.14, 22.41 ppm.

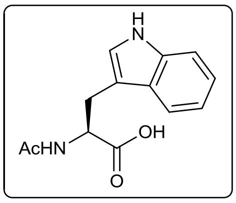


**Fmoc-*3,5*-diiodo-Tyr-OH (15).** To a suspension of 9.0 g (20.8 mmol) of H-*3,5*-I,I-Tyr-OH in 31.2 mL of 10% $NaHCO_3$ was added 7.7 g (22.9 mmol) of Fmoc-OSu in 63 mL of acetone. The resulting mixture was stirred at room temperature for 23 h and acetone was removed by rotary evaporation. Upon extraction with ether, the sodium salt of Fmoc-diiodotyrosine precipitated and was collected by filtration; the solid was washed thoroughly with $H_2O$ then EtOAc and dried. This sodium salt was suspended in 60 mL of $H_2O$ and acidified with 12M HC1. The free acid was extracted with EtOAc. The organic layers were combined, dried over sodium sulfate, filtered and concentrated under vacuum. White solid (11.4 g, 84%). **$^1$H NMR** (400 MHz, DMSO-$d_6$): δ 12.73 (s, 1H), 9.38 (s, 1H), 7.88 (d, $J$ = 7.8 Hz, 2H), 7.75 (d, $J$ = 8.5 Hz, 1H), 7.68 (s, 2H), 7.67 – 7.61 (m, 2H), 7.41 (tdd, $J$ = 7.5, 2.6, 1.1 Hz, 2H), 7.31 (dtd, $J$ = 9.7, 7.5, 1.2 Hz, 2H), 4.20 (m, 3H), 4.10 (ddd, $J$ = 10.6, 8.4, 4.3 Hz, 1H), 2.96 (dd, $J$ = 13.9, 4.4 Hz, 1H), 2.71 (dd, $J$ = 13.8, 10.6 Hz, 1H) ppm.

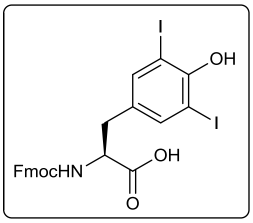

**Ac-*3,5*-di-(Ac-Trp-OH)-Tyr(OAc)-OH (16).** Ac-3,5-diiodo-Tyr(OAc)-OH (100 mg, 0.087 mmol),

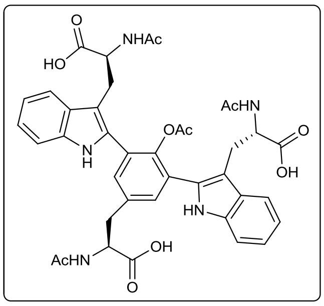

Ac-Trp-OH (286 mg, 1.16 mmol, 6.0 eq.), $AgBF_4$ (226 mg, 1.16 mmol, 6.0 eq.), pivalic acid (30 mg, 0.290 mmol, 1.5 eq.) and $Pd(OAc)_2$ (17 mg, 0.077 mmol, 0.4 eq.) were placed in a microwave reactor vessel in 1200 µL of DMF. The mixture was heated under microwave irradiation (250 W) at 90 °C for 20 min. The residue was filtered and partially purified in a PoraPak Rxn RP 60 cc reverse phase column (5 g) [solvent A (0.1% FA in $H_2O$) and solvent B (0.1% FA in ACN)]. Pale solid (28.5 mg, 20% yield estimated by HPLC-MS conversion). A pure fraction was obtained by semi-preparative RP-HPLC (XBRIDGE™, C18, 5µM OBD 19x100 mm column, [solvent A (0.1% FA in $H_2O$) and solvent B (0.05% FA in ACN)], in 20 min, flux: 16 mL·min-1, detection at λ=220 nm (gradient: 25-30% of B). **$^1$H NMR** (500 MHz, DMSO-*d*$_6$): δ 12.62 (s, 3H), 11.08 – 10.93 (m, 2H), 8.26 (dd, *J* = 15.5, 7.9 Hz, 1H), 7.91 (dd, *J* = 10.8, 8.0 Hz, 1H), 7.82 (t, *J* = 6.9 Hz, 1H), 7.63 (dt, *J* = 7.8, 3.8 Hz, 2H), 7.53 – 7.44 (m, 2H), 7.36 (d, *J* = 8.0 Hz, 2H), 7.10 (t, *J* = 7.5 Hz, 2H), 7.01 (t, *J* = 7.5 Hz, 2H), 4.54 (m, 3H), 3.26 (dd, *J* = 9.2, 6.2 Hz, 2H), 3.22 – 3.17 (m, 1H), 3.16 – 3.05 (m, 2H), 2.96 (m, 1H), 1.92 – 1.76 (m, 3H), 1.73 – 1.56 (m, 9H) ppm. **IR** (Film, $cm^{-1}$) ν = 3353.02, 3051.96, 2923.84, 1732.38, 1649.11, 1527.40 $cm^{-1}$. **HRMS** (ESI) (m/z): $[M+H]^+$ calcd. for $C_{39}H_{39}N_5O_{11}$, 754.27188; found, 754.27292.

**Fmoc-*3*-iodo-Tyr-OH (17).** To a suspension of 2.0 g (6.5 mmol) of H-*3*-I-Tyr-OH in 10.0 mL of

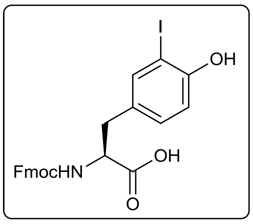

10% $NaHCO_3$ was added 2.4 g (7.2 mmol) of Fmoc-OSu in 20 mL of acetone. The resulting mixture was stirred at room temperature for 20 h and acetone was removed by rotary evaporation. Upon extraction with ether, the sodium salt of Fmoc-diiodotyrosine precipitated and was collected by filtration; the solid was washed thoroughly with $H_2O$ and dried. This sodium salt was suspended in 20 mL of $H_2O$ and acidified with 12M HC1. The free acid was extracted with EtOAc. The organic layers were combined, dried over sodium sulfate, filtered and concentrated under vacuum. The residue was recrystallized from EtOAc/hexane to give a white solid (2.8 g, 81%). **$^1$H NMR** (400 MHz, DMSO-*d*$_6$): δ 10.12 (s, 1H), 7.88 (d, *J* = 7.5 Hz, 2H), 7.74 – 7.58 (m, 4H), 7.41 (dddd, *J* = 7.7, 5.0, 3.8, 2.0 Hz, 2H), 7.31 (dtd, *J* = 10.6, 7.4, 1.2

Hz, 2H), 7.09 (dd, $J$ = 8.3, 2.1 Hz, 1H), 6.79 (d, $J$ = 8.2 Hz, 1H), 4.19 (d, $J$ = 4.3 Hz, 3H), 4.14 – 4.05 (m, 1H), 3.33 (s, 1H), 2.95 (dd, $J$ = 13.8, 4.4 Hz, 1H), 2.73 (dd, $J$ = 13.9, 10.5 Hz, 1H) ppm.

**10-(4-(3-carboxypropanamido)phenyl)-5,5-difluoro-1,3,7,9-tetramethyl-5*H*-dipyrrolo[1,2-*c*:1',2'-*f*][1,3,2]diazaborinin-4-ium-5-uide (18).** Compound **18** was prepared as described in the literature.[5]

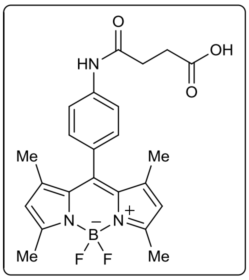


**10-(4-(4-(2-aminoethylamino)-4-oxobutanamido)phenyl)-5,5-difluoro-1,3,7,9-tetramethyl-5*H*-dipyrrolo[1,2-*c*:1',2'-*f*][1,3,2]diazaborinin-4-ium-5-uide (19).** Bodipy derivative **18** (100 mg, 0.228 mmol) and ethylenediamine (76 µL, 1.14 mmol, 5.0 eq.) were dissolved in DCM (5 mL), EDC-HCl (52 mg, 0.271 mmol, 1.2 eq.) and HOBt (37 mg, 0.274 mmol, 1.2 eq.) were added, and the mixture was stirred for 16 h. Another portion of EDC-HCl (52 mg, 0.271 mmol, 1.2 eq.) and HOBt (37 mg, 0.274 mmol, 1.2 eq.) were added, and the reaction further stirred for 24 h. 10 ml of DCM were added, and the mixture was extracted $Na_2CO_{3aq.}$ (3 x 15 mL). The organic layers were combined, dried over $Na_2SO_4$, filtered and evaporated under vacuum. The residue was dissolved in MeOH and loaded into a SCX-2 isolute (500 mg) column, washed with MeOH (30 mL) and released with $NH_3$ solution (4M) in MeOH (10 mL). After removal of the solvents, amino bodipy derivative **19** was recovered as an orange solid (67 mg, 62%). **$^1$H NMR** (400 MHz, $CDCl_3$): δ 9.02 (s, 1H), 7.63 (d, $J$ = 8.1 Hz, 2H), 7.11 (d, $J$ = 8.2 Hz, 2H), 6.48 (s, 1H), 5.88 (s, 2H), 3.42 (s, 2H), 3.28 (s, 2H), 2.85 – 2.53 (m, 6H), 2.46 (s, 6H), 1.33 (s, 6H) ppm. **HPLC-MS** (m/z): [M] calcd. for $C_{25}H_{30}BF_2N_5O_2$, 481.25; [M] found, 481.78.

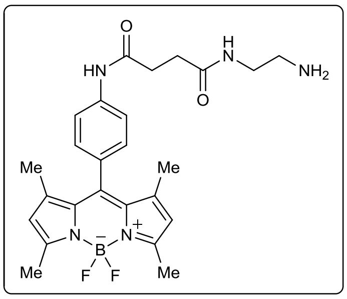

## 4. Biochemical and cellular studies

### Proteolytic degradation assay

Stock solutions of α-chymotrypsin (from bovine pancreas type II, ≥ 40 U/mg) were freshly prepared in 1 mM HCl (placed on ice), and stock solutions of peptides (500 μM) were dissolved in DMSO. Reactions were conducted in glass vials by dilution of α-chymotrypsin solution into assay buffer (56 mMTris pH 7.8, 560 μM $CaCl_2$, 0.1 %*v* Tween-80) and subsequent addition of the peptide stock solution to give a final substrate concentration of 50 μM, 50 μg/mL α-chymotrypsin, and 10 %*v* DMSO. Aliquots were removed at different times and diluted 1:1 with $H_2O$ containing 1 %*v* TFA, which resulted in a pH < 2. The rate of peptide degradation was monitorized by HPLC-MS analysis at the corresponding wavelength were the UV absorption was maximum. No loss was observed in control reactions which contained BSA instead of α-chymotrypsin.

### Cell adhesion assays of RGD-containing compound 2h

As the signaling peptides specifically bind to integrins (transmembrane receptors, directly involved in tumor metastasis and tumor-induced angiogenesis) acting as mild antagonists,[9] we tested wether the rigidified analog conserved this property. The inhibition of the cellular adhesion of the RGD-containing compound **2h** and its linear precursor **1h** were evaluated against Human Umbilical Vein Endothelial Cell cancer cell line, with coating of fibrinogen (Fb) and vitronectin (Vn) as integrin ligands and using cilengitide® as a positive control. The tested compounds selectively block αvβ3-mediated cell adhesion with Fb but do not have blocking activity of Vn against integrin αvβ5.

Non-tissue culture treated ELISA plates [NUNC, Maxisorp 442404] were coated ON at 4 ºC with the specific concentration of the ligand. Coating solution was discarded and wells were blocked with blocking solution (PBS + BSA 1.5%; 60 minutes at 37 ºC). Blocking solution was discarded by flicking and serial dilutions of the compounds were plated in quadruplicates. Immediately, harvested cells are plated at a given concentration (20000-25000 / well for HUVEC and DAOY and 50000 for HT-29) to the same plate. Plates were incubated for 90 minutes at 37 ºC/5%$CO_2$ to allow cell adhesion on the ligand. After then, non-adhered cells were removed and hexosaminidase substrate (N-acetyl-β-

D-glucosaminide) was added to each well and incubated for 3 hours (37ºC/5%$CO_2$ for VN and O/N for de FB). Optical density was read at 405 nm. The proliferation inhibition EC50 was calculated using the Prism-4 software based on the sigmoidal dose-response (variable slope) equation.

Each plate contains positive and negative controls and peptides are tested as duplicates. Each assay has been repeated at least twice and the adhesion inhibition $EC_{50}$ is calculated, when possible, using the Prism-4 software based on the sigmoidal dose-response (variable slope) equation.

Cell adhesion inhibition curves for compounds **1h** and **2h** using Vn and Fb as ligands in HUVEC cell line.

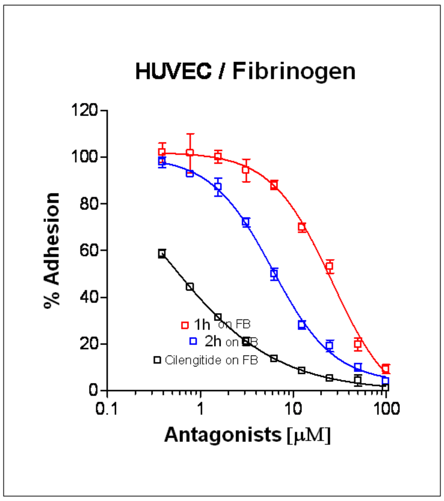


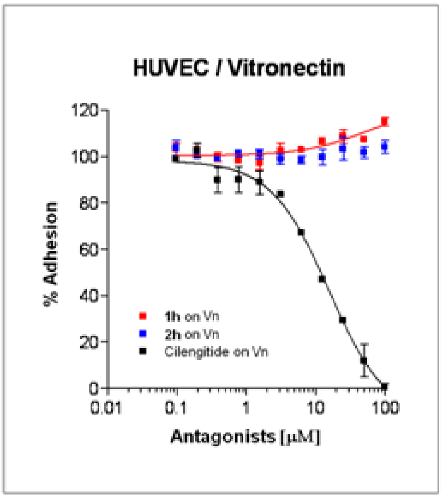


| HUVEC / Fb | $EC_{50}$ (µM) |
|---|---|
| **1h** | **26** |
| **2h** | **6** |
| **Cilengitide** | **0.08** |

| HUVEC / Vn | $EC_{50}$ (µM) |
|---|---|
| **1h** | _ |
| **2h** | _ |
| **Cilengitide** | **16** |

**Cytotoxicity determination of RGD-containing compound 2h**

The citotoxicity of the conjugated peptide **5** and its macrocyclic precursors **3** and **4** were evaluated against three human cancer cell lines: lung carcinoma A549, breast cancer MCF-7 and MCF-10A cell lines. Citotoxicity experiments were performed following the methods indicated elsewhere.[10] Only the macrocycle **4** showed activity ($IC_{50}$ <10 µM) in line with the expected activity for the parent compound (sansalvamide).[11]

**Live imaging of SH-SY5Y cells upon incubation with 1j-Bodipy and 2j-Bodipy**

For confocal microscopy analysis, cells were seeded ($4 \div 12 \times 10^3$ per $cm^2$) on glass chamber coverslips and cultured for 24 h before being incubated for 30 min in fresh medium containing **1j-Bodipy** (750 nM) or **2j-Bodipy** (750 nM). Cells were washed, and fresh medium was introduced to perform experiments with living cells in $CO_2$ and temperature-controlled conditions. Images were collected with a Leica SP5 Spectral confocal microscope attached to an inverted DMI 6000, using a 63×/1.3 Glyc HCX PL APO objective.

## Supplementary References


1. Preciado, S., Mendive-Tapia, L., Albericio, F. & Lavilla, R. Synthesis of C-2 arylated tryptophan amino acids and related compounds through palladium-catalyzed C-H activation. *J. Org. Chem.* **78,** 8129–8135 (2013).
2. Chan, W. C. & White, P. D. *Fmoc solid phase peptide synthesis.* 376 (Oxford University Press, New York, 2000).
3. E. Kaiser, R. L. Colescott, C. D. Bossinger, P. I. C. Color test for detection of free terminal amino groups in the solid-phase synthesis of peptides. *Anal. Biochem.* **34,** 595–598 (1970).
4. Seim, K. L., Obermeyer, A. C. & Francis, M. B. Oxidative modification of native protein residues using cerium (IV) ammonium nitrate. *J. Am. Chem. Soc.* **133,** 16970–16976 (2011).
5. Cui, A. *et al.* Synthesis, spectral properties and photostability of novel boron–dipyrromethene dyes. *J. Photoch. Photobio. A.* **186,** 85–92 (2007).
6. Schubert, M., Labudde, D., Oschkinat, H. & Schmieder, P. A software tool for the prediction of Xaa-Pro peptide bond conformations in proteins based on 13C chemical shift statistics. *J. Biomol. NMR.* **24,** 149–154 (2002).
7. Shen, Y. & Bax, A. Prediction of Xaa-Pro peptide bond conformation from sequence and chemical shifts. *J. Biomol. NMR.* **46,** 199–204 (2010).
8. Spartan'14 for Windows, Macintosh and Linux, version 1.1.4, wavefunction, inc. www.wavefun.com.
9. Manzoni, L. *et al.* Cyclic RGD-containing functionalized azabicycloalkane peptides as potent integrin antagonists for tumor targeting. *Chem. Med. Chem.* **4,** 615–632 (2009).
10. Preciado, S. *et al.* Synthesis and biological evaluation of a post-synthetically modified Trp-based diketopiperazine. *Med. Chem. Comm.* **4,** 1171–1174 (2013).
11. Pan, P.-S. *et al.* A comprehensive study of Sansalvamide A derivatives: The structure-activity relationships of 78 derivatives in two pancreatic cancer cell lines. *Bioorg. Med. Chem.* **17,** 5806–5825 (2009).
12. Zhou, L., Budge, S. M., Ghaly, A. E., Brooks, M. S. & Dave, D. Extraction, purification and characterization of fish chymotrypsin: a review. **7,** 104–123 (2011).
13. Von Kleist, L. *et al.* Role of the clathrin terminal domain in regulating coated pit dynamics revealed by small molecule inhibition. *Cell* **146,** 471–484 (2011).
